# Administration of statins is correlated with favourable prognosis in lung cancer patients receiving immune checkpoint inhibitors

**DOI:** 10.3389/fimmu.2025.1638677

**Published:** 2025-10-06

**Authors:** Jingjing Yang, Jie Lin, Huaijuan Guo, Wenjuan Wu, Jiaxin Wang, Jingxian Mao, Wenbin Fan, Yang Lu, Ying Wang, Xuebing Yan

**Affiliations:** ^1^ Department of Oncology, The Affiliated Hospital of Yangzhou University, Yangzhou University, Yangzhou, Jiangsu, China; ^2^ Department of Hepatobiliary and Pancreatic Surgery, Jilin University Second Hospital, Changchun, Jilin, China; ^3^ Department of Oncology, Northern Jiangsu People’s Hospital affiliated to Yangzhou University, Yangzhou University, Yangzhou, Jiangsu, China; ^4^ Department of Oncology, Baoying Traditional Chinese Medicine Hospital, Yangzhou University, Yangzhou, Jiangsu, China; ^5^ Department of Plastic Surgery, The Affiliated Hospital of Yangzhou University, Yangzhou University, Yangzhou, Jiangsu, China

**Keywords:** lung cancer, immune checkpoint inhibitor, statin, biomarker, prognosis

## Abstract

**Objective:**

Statins are commonly used for cardiovascular diseases and recent studies have supported their anti-cancer role in numerous human malignancies. This study aims to investigate their prognostic impact in lung cancer patients receiving immune checkpoint inhibitors (ICIs).

**Methods:**

A retrospective analysis was performed based on the clinical data from 235 lung cancer patients who received ICI therapy between 2019 and 2024 in three hospitals. The correlation of statin use with overall survival (OS) or progression-free survival (PFS) was analyzed. Then, a comprehensive bioinformatics analysis was used to identify prognostic target genes of statins and investigate their correlation with immune infiltration, followed by validation in an independent cohort and cellular experiments.

**Results:**

In the whole cohort, 80 patients (34.0%) received statins. The statin users had a significantly better OS and PFS than the non-statin users. Statin use was an independent favorable prognostic factor for ICI-treated lung cancer patients. Transcription factor RAR-related orphan receptor alpha (RORA) was identified as a favorable prognostic target gene of statins. RORA was found to be downregulated in lung cancer tissues and correlated with infiltration of some immune cells. In the validation cohort, RORA expression was positively correlated with CD8^+^ T cell infiltration in lung cancer tissues, and improved prognosis in lung cancer patients receiving ICIs. Atorvastatin treatment increased RORA expression and RORA knockdown partly antagonized the inhibitory role of Atorvastatin on the malignant characteristics of lung cancer cells *in vitro*.

**Conclusion:**

Statin use was significantly correlated with improved prognosis in lung cancer patients receiving ICIs. Statins may enhance ICI efficacy partly through RORA. Due to study limitations, the actual role of statins and their target genes in anti-cancer immunity needs further investigations.

## 1 Introduction

Lung cancer ranks the first in the incidence and mortality among all the human malignancies worldwide (1). Despite great advances in screening and targeted therapy, the overall five-year survival rate of lung cancer is low, ranging from 19.7% to 32.9% (2). The introduction of immunotherapy has dramatically extended the overall survival (OS) of patients with unresectable or metastatic disease, with its representative drug known as immune checkpoint inhibitors (ICIs) (3). The pharmacological mechanism of ICIs is inhibition of Cytotoxic T-lymphocyte-associated protein 4 (CTLA-4) or Programmed cell death protein 1 (PD-1) or its ligand (PD-L1) to enhance the anti-cancer function of T cells. The actual efficacy of ICIs is affected by various inherent factors such as microsatellite instability status, PD-L1 expression, tumor mutational burden and host microbiome (4–6). Previously, our team has found some concomitant medications are able to enhance or diminish ICI efficacy such as antibiotics, proton pump inhibitors and opioids (7–9). Since these medications are inevitably used in most cancer patients, a further understanding about their specific roles in cancer immunotherapy is crucial for developing individualized anti-cancer strategies.

Statins, as 3-hydroxy-3-methyl-glutaryl-coenzyme A (HMG-Co-A) reductase inhibitors, are commonly used drugs for cardiovascular diseases through reducing cholesterol (10). Emerging studies have suggested administration of statins may also act as an effective adjuvant anticancer therapy. For instance, ovarian cancer patients who received niraparib (a Poly (ADP-ribose) polymerase inhibitor) and statins had an improved progression free survival (PFS) as compared with those who only used niraparib (11). Statin use is also correlated with improved outcome in patients with esophageal squamous cell carcinoma who received concurrent chemoradiotherapy (12). Recent mechanism investigations have revealed statin can inhibit the PD-L1 expression in cancer cells, implying its potential in activating anti-cancer immunity (13, 14). In a retrospective work based on SEER-Medicare database, statin use was associated with reduced cancer-specific mortality in non-small cell lung cancer (NSCLC) patients who received ICI therapy (15). In patients with advanced NSCLC who received PD-1 inhibitors, statin use was associated with improved objective response rate (ORR) and PFS instead of OS (16). In contrast, another study demonstrated statin use was associated with prolonged OS instead of PFS in NSCLC patients receiving anti-PD-1 monotherapy (17). In addition, there are several studies reporting negative results (18, 19). Therefore, the prognostic impact of statin use in ICI-treated patients with lung cancer remains controversial, suggesting the necessity of further investigations.

In this study, a multicenter retrospective cohort enrolling 235 patients was utilized to clarify the prognostic impact of statin use. Then, a bioinformatics method was used to identify statin target genes (STGs) that were potentially correlated with clinical outcome and immune infiltration, followed by clinical and cellular validations. This study provides novel insights into the role of statins in cancer immunotherapy, contributing to more precise management of concomitant medications in clinical practice.

## 2 Materials and methods

### 2.1 Patient information

**Figure 1:**
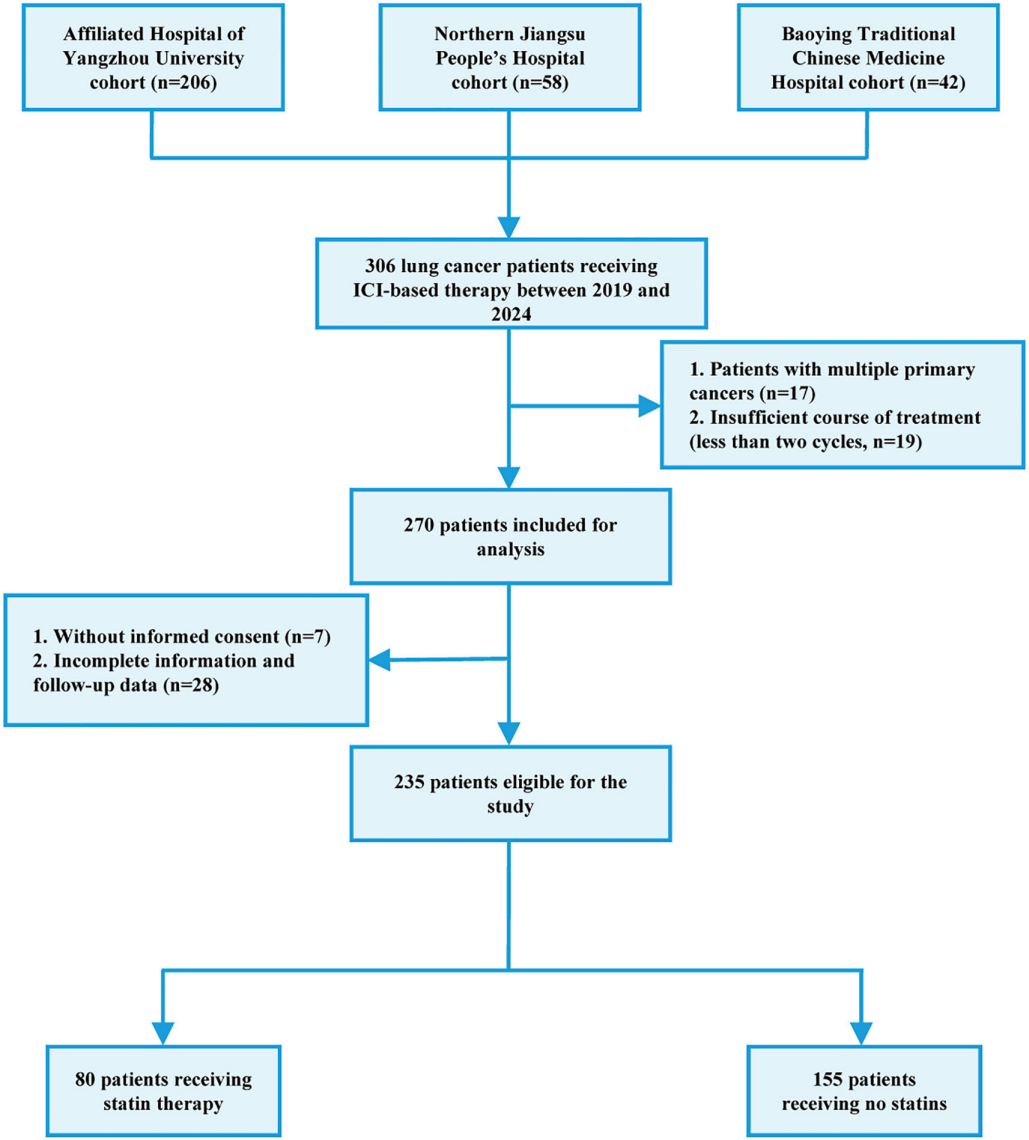
Flowchart of patient recruitment in the retrospective study.

The flow chart of patient recruitment was shown in **Figure 1**. Between January 2019 and November 2024, 306 patients who received anti-therapies at Department of Oncology, the Affiliated Hospital of Yangzhou University (n=206), Nothern Jiangsu People’s Hospital Affiliated Yangzhou University (n=58) and Baoying Traditional Chinese Medicine Hospital (n=42) were initially included. The inclusion criteria were as follows: 1) patients aged over 18; 2) pathologically diagnosed as lung cancer; 3) patients receiving ICIs or combined with other anti-cancer therapies including chemotherapy, radiotherapy, and targeted therapy. The exclusion criteria were as follows: 1) multiple existing primary tumors; 2) incomplete medical records; 3) data missing in the follow-up; 4) insufficient ICI therapy (less than two cycles); 5) hyperprogression. Hyperprogression was defined as Evaluation Criteria in Solid Tumors (RECIST) version 1.1 progressive disease at the first computed tomography (CT) scan during ICI therapy and an absolute increase in the tumor growth rate exceeding 50% per month (20). As a result, a total of 235 patients were finally included. This study was approved by the local ethics committee (No. 2022-YKL11-class 05). The informed consents were acquired from patients for using their tissue samples and medical information in scientific researches.

### 2.2 Therapeutic regimens and oncology evaluation

For ICI therapy, the following drugs were used: sintilimab (n=84), tirelizumab (n=60), camrelizumab (n=29), pembrolizumab (n=13), durvalumab (n=12), serplulimab (n=11), toripalimab (n=11), atezolizumab (n=8), nivolumab (n=4), adebrelimab (n=2) and envafolimab (n=1). The management of drug toxicities was carried out according to National Comprehensive Cancer Network (NCCN) guidelines (21). Corticosteroids could be used for treating most high-grade toxicities and permanent discontinuation of ICI drugs was suggested in case of severe toxicities. For monitoring drug toxicities, patients received physical examination and laboratory detection every two or three weeks. In case of pulmonary or cardiovascular toxicity, CT or ultrasound examination are recommended. For chemotherapy, the following drugs were used: platinum (n=175), etoposide (n=55), paclitaxel (n=48), pemetrexed (n=46), nab-paclitaxel (n=37), docetaxel (n=7) and gemcitabine (n=5). In case that severe toxicities were observed, the drug dose were reduced by 25-50% or another regimen was recommended. For targeted therapy, the following drugs were used: bevacizumab (n=14), anlotinib (n=11) and apatinib (n=2). A total of 22 patients received radiotherapy. Chemotherapy combined with immunotherapy was the most commonly used, the representative chemotherapeutic agents were pemetrexed (500 mg/m^2^) plus cisplatin (75 mg/m^2^) or carboplatin (area under the curve=5 mg/mL/min) once every 3 weeks.

The therapy response was evaluated every two or three cycles, based on serum tumor biomarkers and radiological examination. The RECIST version 1.1 was used to determine Complete Response (CR), Partial Response (PR), Progressive Disease (PD) and Stable Disease (SD). The clinical outcome was determined using OS and PFS. The OS was defined as the period from ICI initiation to death from any cause, while PFS was defined as the period from ICI initiation to PD.

### 2.3 Identification of statin target genes in online databases

The STGs were obtained from the DrugBank (https://go.drugbank.com) (22), Comparative Toxicogenomics Database (https://ctdbase.org/) (23), Swiss Target Prediction (http://www.swisstargetprediction.ch/) (24) and TargetNet (http://targetnet.scbdd.com/calcnet/index/) (25) databases.

### 2.4 Prognostic analysis of statin target genes in online databases

The transcriptome data of Lung Adenocarcinoma (LUAD) and Lung Squamous Cell Carcinoma (LUSC) from The Cancer Genome Atlas (TCGA, https://portal.gdc.cancer.gov/v1/) database were downloaded. The favorable prognostic genes were identified using the univariate cox regression method with the following inclusion criteria: HR<1 and p<0.05. Then, the shared genes between the favorable prognostic genes and STGs were identified and their prognostic significance was validated using the Kaplan-Meier survival model (p<0.01). The associations between the identified genes and clinical features were determined using UALCAN database (https://ualcan.path.uab.edu/index.html) (26).

### 2.5 Immune infiltration analysis

For quantifying immune cell infiltration, the following algorithms were used: EPIC, MCP-COUNTER, TIMER, XCELL, QUANTISEQ, CIBERSORT and CIBERSORT-ABS. The immune subtypes were classified as follows: C1 (Wound healing), C2 (IFN-gamma dominant), C3 (Inflammation), C4 (lymphocyte depletion), C5 (Immunologically Quiet), and C6 (TGF-beta dominant). The tumor immune micro-environment cell composition database (TIMEDB) was used to investigate the correlation of STGs with immune cells (https://timedb.deepomics.org/) and the details were provided in **Supplementary Table S1** (27). For further confirming the correlation, the cellular distribution of STGs were analyzed using single-cell sequencing data from TISCH database (http://tisch.comp-genomics.org/home/).

### 2.6 Immunohistochemical staining

The tumor and adjacent normal tissues were collected from 42 NSCLC patients who received ICI-based therapy at Department of Oncology, the Affiliated Hospital of Yangzhou University. The paraffin embedded tissue samples were cut into 4μm-thick sections, then dewaxed using xylene and rehydrated using gradient alcohol. After antigen retrieval and blocking endogenous peroxidase activity, the sections were incubated with the primary antibody against RAR-related orphan receptor alpha (RORA) (Proteintech, USA, 1:200) or CD8 (Cell Signaling Technology, USA, 1:1000) overnight. The sections were then incubated with the secondary antibody (Cell Signaling Technology, 1:1000) for 30 min and staining was visualized using Diaminobenzidine Kit (Thermo Fisher Scientific, USA). For staining assessment for RORA expression, the scoring system was calculated based on staining Intensity (SI) and Percentage of Positive stained cells (PP). SI was classified as follows: negative (score 0), weak (score 1), moderate (score 2) and strong (score 3). PP was classified as follows: ≤5% (score 0), 6-25% (score 1), 26-50% (score 2), 51-75% (score 3) and ≥76% (score 4). A final score was calculated by multiplying the scores of SI and PP. High RORA expression was defined as a final score more than 6. For staining assessment for CD8 expression, more than 30 positively stained cells per field (×400) in the tumor stroma was defined as CD8^+^ T cell rich tumor tissues, while the opposite case was defined as CD8^+^ T cell deficient tumor tissues.

### 2.7 Cell culture, plasmid construction and lentiviral packaging

A549 cells were obtained from the Type Culture Collection of the Chinese Academy of Sciences (Shanghai, China) and were cultured in complete F-12K medium (Procell, USA) supplemented with 10% fetal bovine serum (FBS) (Thermo Fisher Scientific), 1% L-alanyl-L-glutamine and 1% penicillin/streptomycin. The cells were maintained at 37°C in a humidified incubator with 5% CO_2_.

### 2.8 RNA interference and plasmid construction

The sequences for RORA knockdown (KD) and negative control (NC) were as follows: KD: (Forward: 5’-GCUUCUACCUGGACAUACA-3’, Reverse: 5’-UGUAUGUCCAGGUAGAAGC-3’); NC: (Forward: 5’-UUCUCCGAACGU GUCACGU-3’, Reverse: 5’-ACGUGACACGUUCGGAGAA-3’). The plasmid construction, lentiviral packaging and transfection was performed according to our previous study (28).

### 2.9 Western blot analysis

Total protein from A549 cells was extracted using cell lysis buffer (Servicebio, China). Equal amounts of protein were separated by 10% SDS–polyacrylamide gel electrophoresis and transferred onto a nitrocellulose membrane. The membrane was blocked at room temperature for 30 min, followed by incubation with a primary antibody against RORA (Proteintech, 1:1000) at 4°C overnight. Then, the membrane was incubated with horseradish peroxidase-conjugated goat anti-rabbit IgG secondary antibody (Beyotime, China, 1:1000) at room temperature for 1h. Finally, the membrane was visualized using an enhanced chemiluminescence detection kit (Beyotime).

### 2.10 Cell counting kit-8 assay

A549 cells were seeded in a 96-well plate and incubated for 24 hours. The cells were then treated with different concentrations of Atorvastatin (MedChemExpress, USA) and incubated for 24, 48, 72, or 96 hours. After washing with PBS, 10 µL of CCK-8 solution (Beyotime) was added. After 1h incubation, absorbance at 450 nm was measured using a microplate reader.

### 2.11 Colony formation assay

A549 cells were plated into six-well culture dishes and maintained under humidified conditions at 37°C with 5% CO_2_ for two weeks. After incubation, the colonies were fixed in 4% paraformaldehyde for 20 minutes, followed by staining with crystal violet solution (Beyotime) for 20 minutes and counted.

### 2.12 Invasion and migration assay

The invasive and migratory capabilities of A549 cells were evaluated using a transwell chamber assay. Briefly, A549 cells were seeded into the upper chamber with serum-free medium, with the lower chamber filled with complete medium. After 24h incubation, the cells were fixed with paraformaldehyde (Beyotime), stained with crystal violet and counted under a microscope. For the invasion assay, a matrix gel was added to the upper chamber before the experiment.

### 2.13 Statistical analysis

The statistical analysis was performed using SPSS software (version 25.0), GraphPad prism (version 8.0) and R project (version 4.3.0). The clinical correlation analysis was performed using the chi-square test. The survival curves were plotted using the Kaplan-Meier model and the survival difference was compared using the log-rank test. The factors affecting OS or PFS were identified using the univariate and multivariate analysis based on cox proportional hazards regression model. Comparisons between two groups were evaluated using t-test, while differences among more than two groups were assessed by one-way analysis of variance (ANOVA). Statistical significance was defined as a p-value < 0.05.

## 3 Results

### 3.1 General description of patient characteristics

The baseline clinical characteristics of included patients were shown in **Table 1**. 202 patients (86.0%) were males. The median age at initial diagnose was 69 years, ranging from 48 to 88 years. 199 patients (84.7%) were pathologically diagnosed as NSCLC, while the rest (15.3%) were diagnosed as small cell lung cancer (SCLC). 36 patients (15.3%) have previously received surgical treatment. 123 patients (52.3%) had a history of smoking. 216 patients (91.9%) received ICI drugs combined other anti-therapies, while the rest (8.1%) received ICI monotherapy. There were 13 patients (5.5%) with EGFR mutation site, 40 patients (17.0%) with other mutation sites, and 182 patients (77.4%) without sequencing information. There were 64 patients (27.2%) with negative PD-L1 expression, 94 patients (40.0%) with positive PD-L1 expression, and 77 cases (32.8%) without information about PD-L1 expression. 80 patients (34.0%) received statins, and the most commonly used drug type was Rosuvastatin (n=58), followed by Atorvastatin (n=12) and Simvastatin (n=10). The correlation analysis demonstrated that statin use was significantly correlated with coronary artery disease(p<0.001), while no significant difference was observed in the result clinical features between the statin users and non-statin users (all p>0.05).

**Table 1:** Baseline characteristics of the entire cohort.

Clinical features	Non-statin (n=155)	Statin (n=80)	P-value
Age			0.253
<70 years old	75(48.4)	45(56.3)	
≥70 years old	80(51.6)	35(43.8)	
Gender			0.200
Female	25(16.1)	8(10.0)	
Male	130(83.9)	72(90.0)	
Smoking			0.810
Never	73(47.1)	39(48.8)	
Current/former	82(52.9)	41(51.3)	
Pathological type			0.384
NSCLC	129(83.2)	70(87.5)	
SCLC	26(16.8)	10(12.5)	
Stage			0.336
III	46(29.7)	19(23.8)	
IV	109(70.3)	61(76.3)	
ECOG			0.051
0-1	82(52.9)	53(66.3)	
≥2	73(47.1)	27(33.8)	
Treatment			0.788
Monotherapy	12(7.7)	7(8.8)	
Combination	143(92.3)	73(91.3)	
Surgery History			0.922
No	131(84.5)	68(85.0)	
Yes	24(15.5)	12(15.0)	
Therapy Lines			0.500
1–2 lines	137(88.4)	73(91.3)	
3^th^ line or more	18(11.6)	7(8.8)	
PD-L1 expression			0.157
Negative	48(31.0)	16(20.0)	
Positive	61(39.4)	33(41.3)	
NA	46(29.7)	31(38.8)	
Hypertension			0.085
No	112(72.3)	49(61.3)	
Yes	43(27.7)	31(38.8)	
Coronary artery disease			<0.001
No	128(82.6)	31(38.8)	
Yes	27(17.4)	49(61.3)	
Gene mutation			0.519
EGFR mutation	8(5.2)	5(6.3)	
Other mutation	25(16.1)	15(18.8)	
NA	122(78.7)	60(75.0)	

SCLC, small cell lung cancer; NSCLC, non-small cell lung cancer; ECOG, Eastern Cooperative Oncology Group; NA, not available.

### 3.2 Prognostic impact of statin use in lung cancer patients receiving ICIs

**Figure 2:**
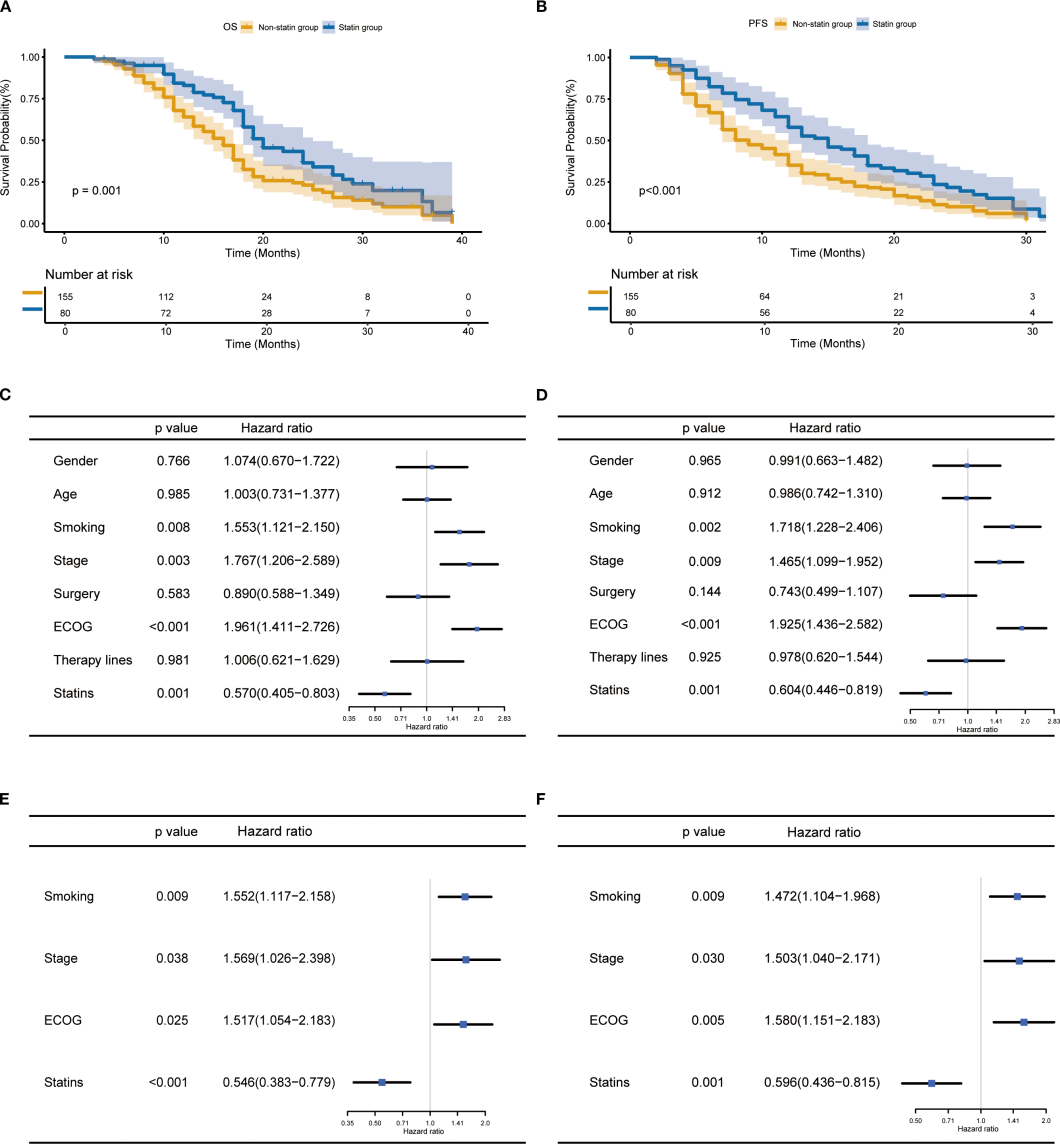
Prognostic impact of statin use in lung cancer patients receiving immune checkpoint inhibitor therapy. **(A, B)** Kaplan-Meier curves for the correlation of statin use with overall survival (OS) **(A)** and progression-free survival (PFS) **(B)**. **(C, D)** Univariate analysis for OS **(C)** and PFS **(D)**. **(E, F)** Multivariate analysis for OS **(E)** and PFS **(F)**.

In the entire cohort, the statin users had a significantly better OS (p=0.001, **Figure 2A**) and PFS (p<0.001, **Figure 2B**) than the non-statin users. The univariate analysis revealed smoking history, ECOG, tumor stage and statin use were significantly correlated with both OS and PFS (all p<0.05, **Figures 2C, D**). The multivariate analysis confirmed statin use together with smoking history, ECOG and tumor stage were independent factors affecting OS and PFS (all p<0.05, **Figures 2E, F**). In terms of treatment response (**Supplementary Figure S1**). After the first evaluation, the statin users had significantly higher objective response rate (ORR) and disease control rate (DCR) than the non-statin users.

In the subgroup analysis, the statin use was associated with improved OS and PFS in patients aged below 70 years (**Supplementary Figures S2A, B**). In contrast, among patients aged 70 years or older, the statin use was associated with improved PFS, whereas no statistically significant difference was observed in OS (**Supplementary Figures S2C, D**). Regarding smoking status, statin use was linked to improved OS and PFS in smokers (**Supplementary Figures S3A, B**). In the non-smokers, statin use was significantly associated with improved PFS instead of OS (**Supplementary Figures S3C, D**). In patients with NSCLC, the statin use was associated with improved OS and PFS (**Supplementary Figures S4A, B**), and similar results were observed in patients with SCLC (**Supplementary Figures S4C, D**). In patients with stage III, no significant differences in outcome were observed between the statin and non-statin group (**Supplementary Figures S5A, B**). In patients with IV stage, the statin group had significantly better prognosis than the non-statin group (**Supplementary Figures S5C, D**). Finally, the use of rosuvastatin was found to associate with improved OS and PFS (**Supplementary Figures S6A, B**).

### 3.3 Prognostic significance of selected statin target genes in patients with lung cancer

**Figure 3:**
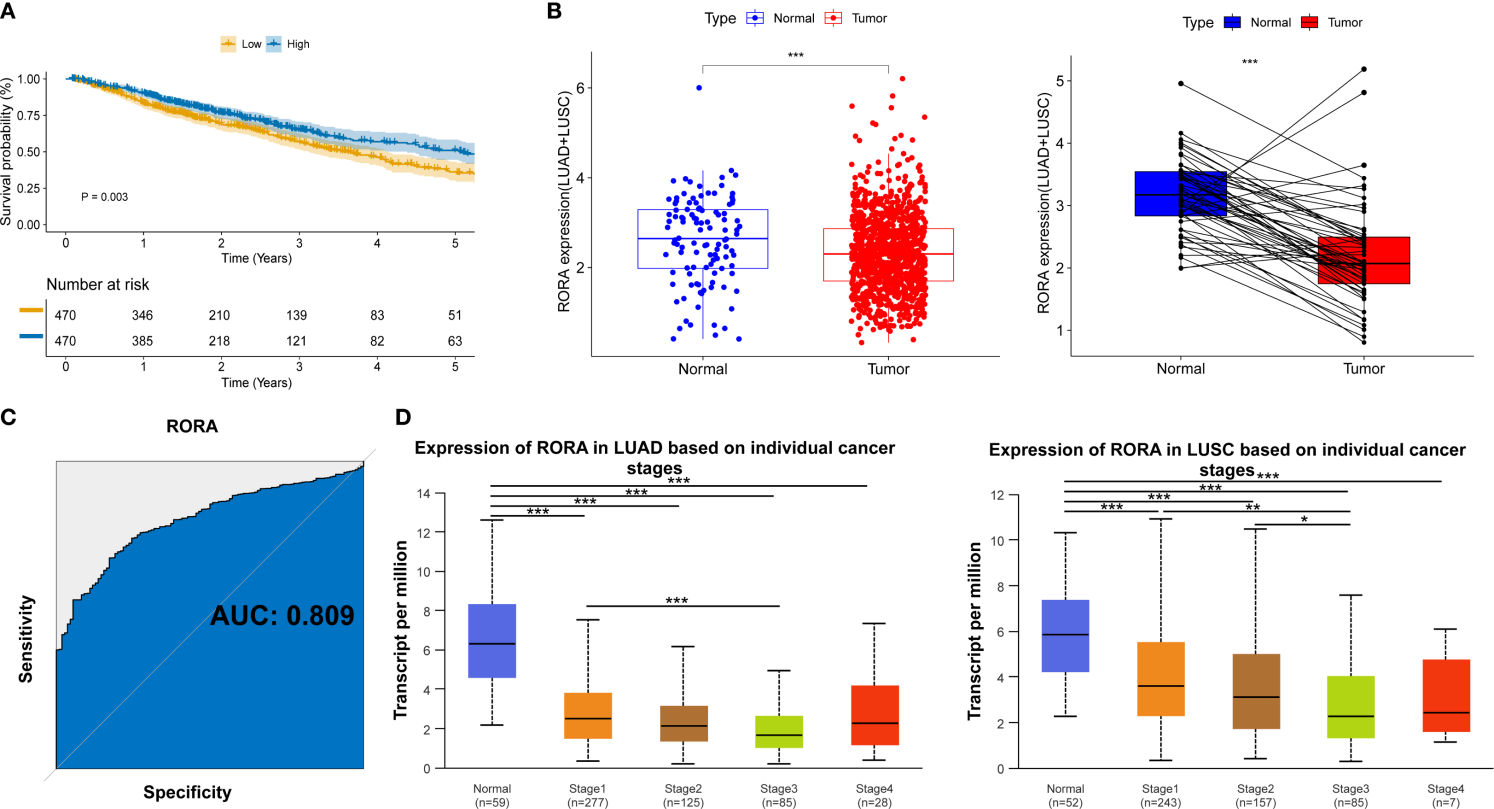
Clinical significance of RORA in NSCLC patients from online databases. **(A)** Kaplan-Meier curves for the correlation of RORA expression with overall survival. **(B)** RORA expression in the tumor and normal lung tissues of NSCLC patients. **(C)** Receiver operator characteristic curve for the diagnostic performance of RORA in distinguishing lung cancer tissues from normal tissues. **(D)** RORA expression in normal lung tissues and tumor tissues from lung cancer patients within different TNM stages. *p<0.05, **p<0.01, ***p<0.001.

For clarifying the mechanisms underlying the impact of statins on ICI drugs, we focused on STGs. As shown in **Supplementary Figure S7A**, target genes of three representative statin drugs (Atorvastatin, n=718; Rosuvastatin, n=550; Simvastatin, n=1058) were initially obtained from four online drug databases. Then, a total of 504 shared target genes were identified (**Supplementary Figure S7B**) and the details were provided in **Supplementary Table S2**. Meanwhile, a total of 457 favorable prognostic genes were identified through the univariate cox regression using the TCGA database and the details were provided in **Supplementary Table S3**. As shown in **Supplementary Figure S7C**, a total of 11 overlap genes were finally determined between 504 STGs and 457 favorable prognostic genes. For further confirming the prognostic significance of these 11 genes, the Kaplan-Meier survival model was utilized. As a result, high expression of transcription factor RORA was found to significantly associate with better survival of patients with lung cancer (p=0.003, **Figure 3A** and **Supplementary Figure S8**). The expression of RORA was also found to be significantly reduced in tumor tissues as compared with normal lung tissues from patients with lung cancer (**Figure 3B**). The receiver operator characteristic (ROC) curve analysis revealed the diagnostic area under the curve (AUC) for distinguish tumors from normal tissues is 0.809 (**Figure 3C**). In addition, the analysis based on the UALCAN database confirmed that RORA expression was significantly reduced in tumor tissues from lung cancer patients within TNM stage I-IV as compared with that in normal lung tissues (**Figure 3D**). The cellular assay demonstrated RORA overexpression (**Supplementary Figure S9A**) significantly inhibited the proliferation (**Supplementary Figure S9B**), colony formation (**Supplementary Figure S9C**), invasion (**Supplementary Figure S9D**), and migration (**Supplementary Figure S9E**) of lung cancer cells.

### 3.4 Correlation of RORA with immune cell infiltration

**Figure 4:**
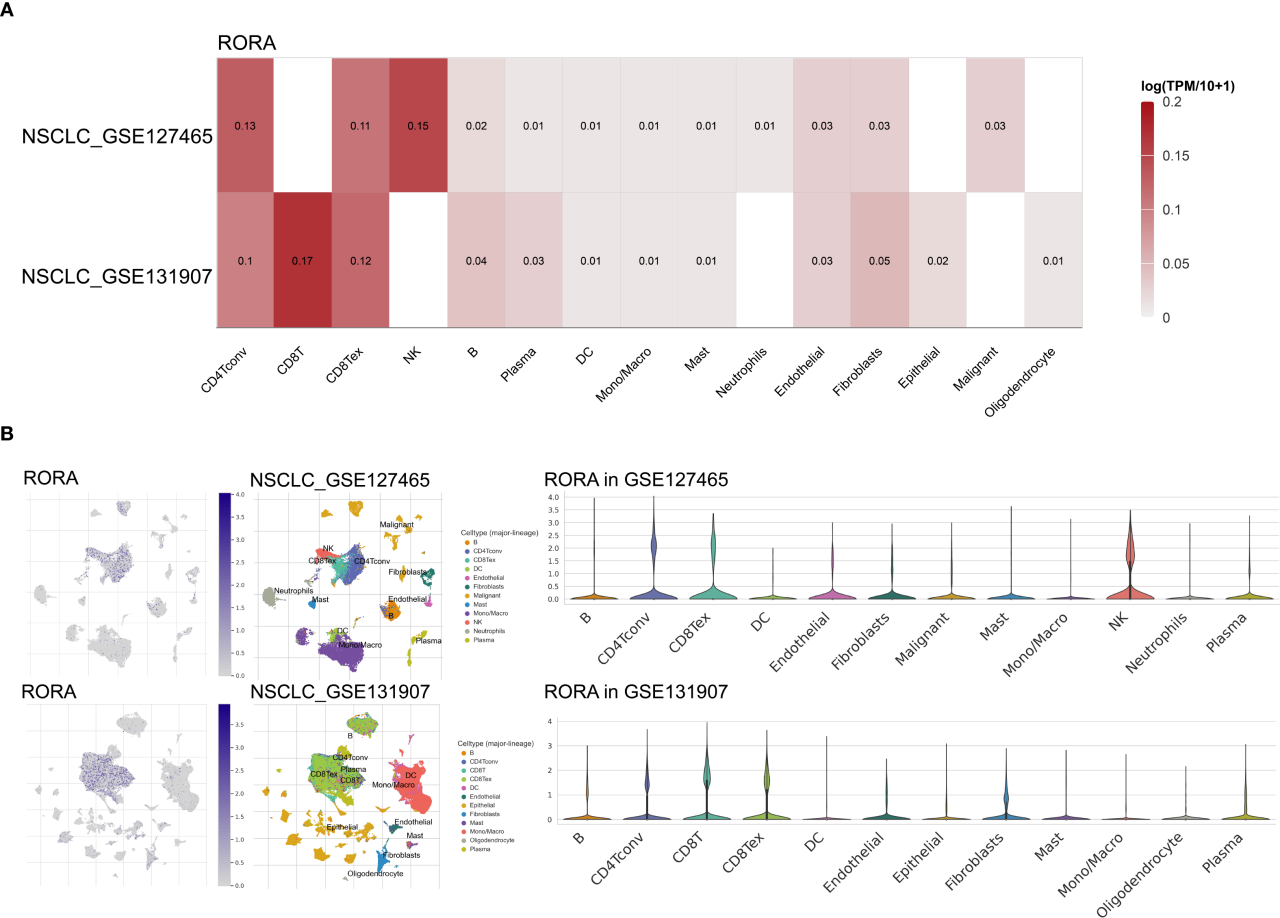
Correlations of RORA with immune infiltration in NSCLC patients. **(A)** Correlation of RORA with different immune cells at the single-cell level in the GSE127465 and GSE131907 NSCLC cohorts. **(B)** Localization of RORA in different immune cells in the GSE127465 and GSE131907 NSCLC cohorts.

As shown in **Supplementary Figure S10A**, the RORA expression was positively correlated with infiltration of numerous immune cells such as B cells, CD4^+^ and CD8^+^ T cells. Using the TCGA cohort including 867 lung cancer patients, high RORA expression was predominantly correlated with immune C1 type (Wound Healing), followed by immune C2 type (IFN-gamma Dominant) and immune C3 type (Inflammatory) (**Supplementary Figure S10B**). For further confirming the correlation between RORA and immune cells, two NSCLC single-cell datasets were utilized. As shown in **Figure 4A**, RORA expression was positively correlated with NK cells in NSCLC_GSE127465 dataset and CD8^+^ T cells in NSCLC_GSE131907 dataset. This result was also confirmed by gene localization analysis in **Figure 4B**.

### 3.5 Validation of the correlation between RORA and CD8^+^ T cells

**Figure 5:**
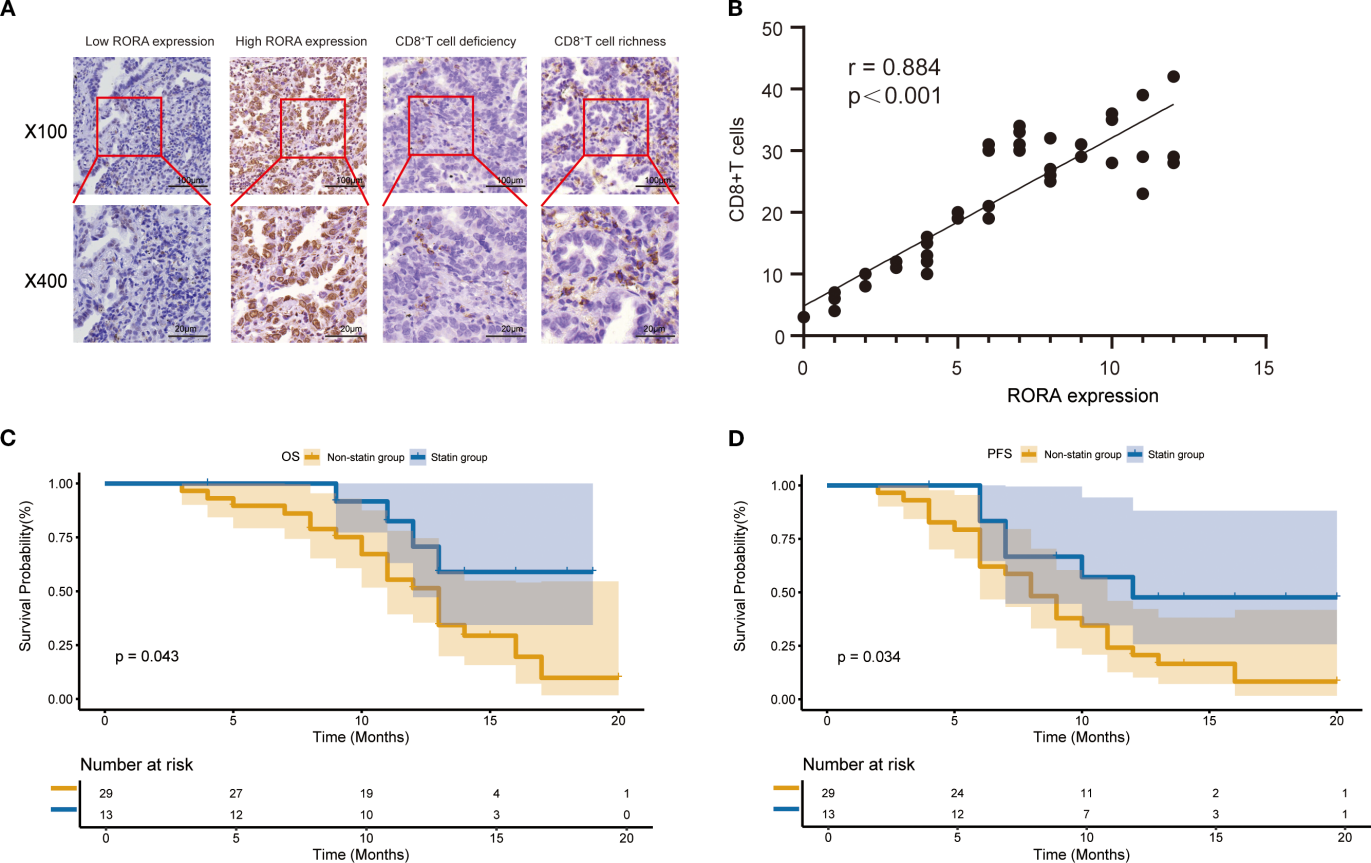
Expression of RORA and its correlation with CD8^+^ T cells in a validation cohort. **(A)** Representative staining images for RORA expression and CD8^+^ T cells in the tumor tissues from patients with lung cancer. **(B)** Correlation of RORA expression with proportion of CD8^+^ T cells in tumor tissues. **(C, D)** Kaplan-Meier curves for the correlation of RORA expression with overall survival **(C)** and progression-free survival **(D)** of lung cancer patients receiving immune checkpoint inhibitors.

Since the bioinformatics analysis has closely linked RORA expression with CD8^+^ T cells, we next validated this correlation in tissue samples from a cohort including 42 ICI-treated patients with lung cancer and patient information was provided in **Supplementary Table S4**. The expression of RORA and CD8 in normal and tumor tissues were detected using IHC and representative staining images were shown **Figure 5A**. The positive expression of RORA were mainly detected in the cell nucleus while that of CD8 were mainly found in the tumor stroma. According to the evaluation criterion, 16 and 26 cases were defined as high and low RORA expression respectively, while 13 and 29 cases defined as CD8^+^ T cell richness and deficiency. The correlation analysis demonstrated that RORA expression was positively correlated with CD8^+^ T cell abundance (r=0.884, p<0.001, **Figure 5B**). In addition, a significant positive correlation was also observed between statin use and CD8^+^T cells richness (p=0.032). Finally, high RORA expression in tumor tissues was found to associate with better OS (**Figure 5C**) and PFS (**Figure 5D**) in lung cancer patients receiving ICI based therapy.

### 3.6 RORA knockdown partly rescued the inhibitory role of Atorvastatin in the malignant characteristics of lung cancer cells

**Figure 6:**
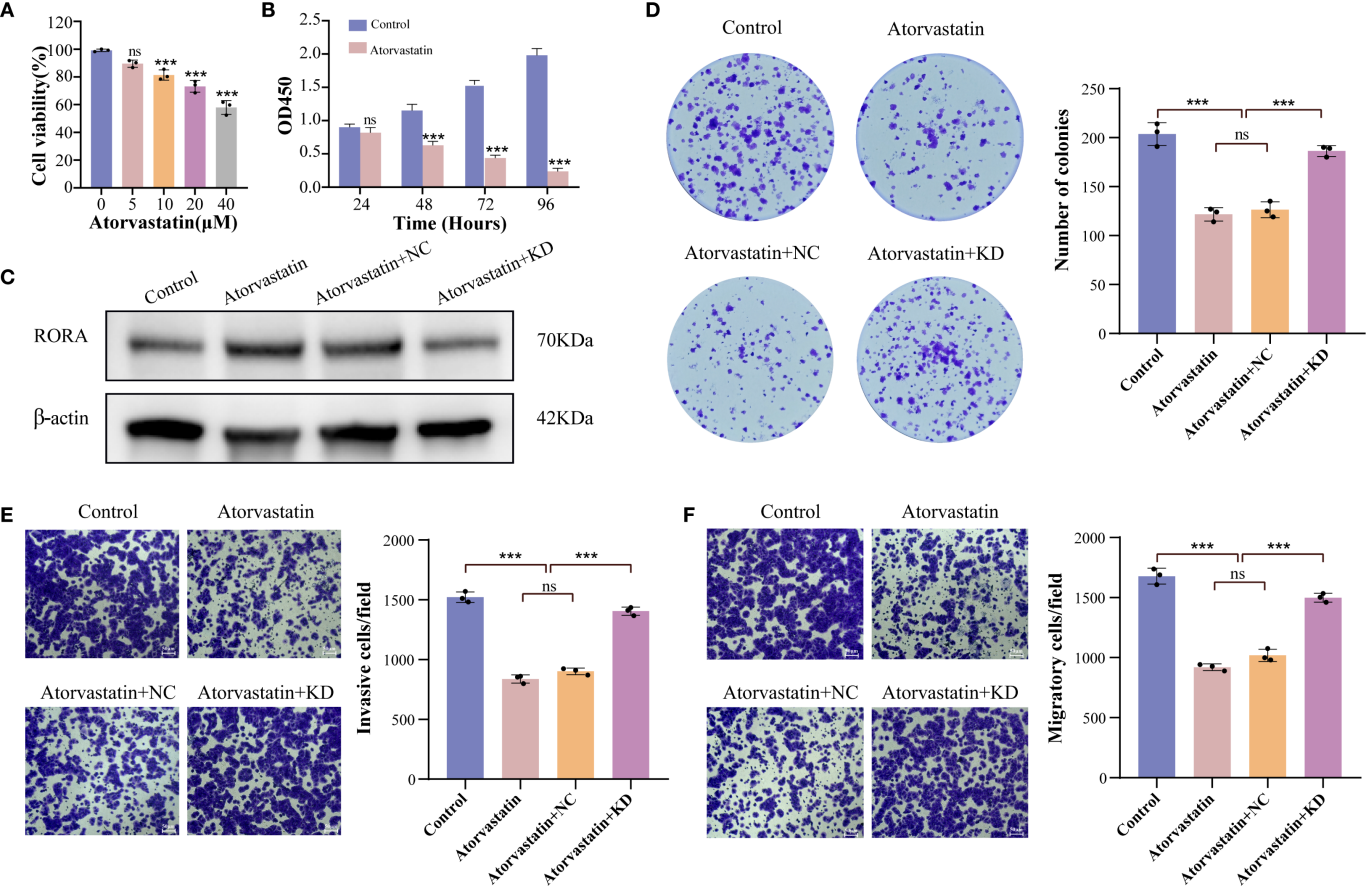
RORA knockdown partly rescues the inhibitory impact of Atorvastatin on the malignant characteristics of lung cancer cells. **(A)** Impact of gradient concentrations of Atorvastatin on the viability of lung cancer cells. **(B)** CCK-8 detecting the impact of Atorvastatin on the proliferation of lung cancer cells within 96h. **(C)** Western blot detecting the protein expression of RORA in Atorvastatin- treated lung cancer cells with or without RORA knockdown. **(D)** Colony formation assay detecting the formed clones of Atorvastatin-treated lung cancer cells with or without RORA knockdown. **(E, F)** Transwell assay detecting the invasion **(E)** and migration **(F)** of Atorvastatin-treated lung cancer cells with or without RORA knockdown. ***p < 0.001. ns, not significant.

For further confirming the correlation between statins and RORA, we selected Atorvastatin for cellular assays. The CCK-8 assay determined that the optimum inhibitory concentration of Atorvastatin was 40 μM (**Figure 6A**). Using this concentration, Atorvastatin treatment effectively impaired the viability of lung cancer cells within 96h (**Figure 6B**). The western blot demonstrated that Atorvastatin treatment significantly increased the protein expression of RORA (**Figure 6C**). The function assays demonstrated Atorvastatin treatment effectively inhibited the colony formation (**Figure 6D**), invasion (**Figure 6E**), and migration (**Figure 6F**) of lung cancer cells, while RORA knockdown could partly rescue this inhibitory impact.

## 4 Discussion

With the rapid development of cardio-oncology, several cardiovascular drugs such as β-blockers and statins have gained increasing attention in cancer prevention and treatment (29). For instance, statin use was found to effectively prevent lung cancer developing from idiopathic pulmonary fibrosis (30). A SEER database analysis demonstrated statin use after cancer diagnosis was correlated with reduced cancer-specific mortality of patients with lung cancer or pancreatic cancer (31). Concurrent chemoradiotherapy combined with statin use has been proved to improve OS and cancer-specific survival of patients with unresectable stage III lung squamous cell carcinoma (32). A recent meta-analysis has suggested concomitant statin use was associated with improved OS and PFS of cancer patients receiving ICIs, implying its potential to enhance ICI efficacy (33). However, in the subgroup analysis of this meta-analysis, the association was only statistically significant in patients with renal cell carcinoma instead of those with NSCLC or melanoma, adding complexity into the prognostic impact of statin use. In this study, using a multicenter retrospective cohort enrolling 235 patients with lung cancer, we aimed to clarify the impact of statins on ICI efficacy, which may benefit the precise management of concomitant medications in anti-cancer immunotherapy.

In the survival analysis, the statin users were found to have a significantly better OS and PFS than the non-statin users. In addition, statin use was identified as an independent favorable prognostic factor. The subgroup analysis further confirmed its beneficial impact in patients with different clinical features. These findings collectively suggest statin use may enhance the efficacy of ICI therapy, which is accordance with some previous studies (13, 16, 34). In other cancers, similar results are observed. For instance, statin use was significantly correlated with prolonged OS and PFS in patients with metastatic renal cell carcinoma who received nivolumab (35). Statin use was identified as an independent favorable predictor for objective response to ICI therapy in patients with recurrent or metastatic head and neck cancer (36). However, there also several studies reporting negative results (18, 19, 37). In the retrospective work, various intrinsic factors such as patient selection, therapy strategy and other concomitant drugs may collectively result in inconsistent findings, suggesting the necessity of well-designed clinical trials to further validate the actual role of statins in immunotherapy. Previous studies have demonstrated statin users were found to have a higher risk of immune-related adverse events (irAEs) such as anemia, arthralgia, colitis and gastrointestinal toxicity than non-statin users (38, 39). One putative explanation is that statins may activate cytotoxic CD8^+^ T cells to directly damage healthy tissues or activate CD4^+^ T cells mediated pro-inflammatory signaling pathways (40). It is reasonable that additional monitoring should be made on statin users during ICI therapy, which may benefit the early diagnosis and prevention of irAEs.

There are some mechanism investigations to support the role of statins in enhancing ICI therapy. Firstly, increased cholesterol not only directly supports tumor cell metabolism, but also impairs tumor antigen presentation as well as T cell proliferation and activation (41). Statins as inhibitors of cholesterol synthesis have been proved to shape the immune tumor microenvironment. For instance, Simvastatin inhibits lncRNA SNHG29 level in tumors and then inactivates YAP, resulting in decreased PD-L1 expression and enhanced cytotoxic T lymphocyte infiltration (42). Lovastatin represses PD-L1 expression through inactivating its transcription factor such as NF-κB, STAT1 and STAT3, increases CD4^+^ and CD8^+^ T cells in the tumors from the mice receiving anti-PD-1 therapy (13). Both Simvastatin and Lovastatin contributed to T cell-induced killing of tumor cells *in vitro*, and enhanced response to anti-PD-1 therapy in mice bearing mouse oral cancer cells through activating T cells (43). However, this study also found both the drugs were able to inhibit T cell proliferation at a high dose, implying importance of determining their optimal doses in combination with ICIs. Secondly, ICI drugs was commonly used in combination with chemotherapeutic drugs, which not only directly kills tumor cells but may also exerts a detrimental impact on immune cells and therefore weakens the anti-cancer immune response (44). Recent studies have found statins could improve the immune suppressive microenvironment induced by chemotherapeutic drugs. For instance, Lovastatin inhibited the PD-L1 expression induced by paclitaxel, and enhance anti-cancer efficacy of chemoimmunotherapy through promoting infiltration of CD8^+^ T cells (45). Simvastatin combined with cisplatin increased the proportion of CD86^+^ maturated dendritic cells and CD8^+^ T cells, creating a favorable immune microenvironment for ICI therapy (46). Finally, statin use prevented cardiovascular irAEs through immune modulation and endothelial protection, which benefits the overall prognosis of patients receiving ICI therapy (47).

For further clarifying the mechanisms underlying clinical findings, the network pharmacological method was utilized. As a result, numerous molecular targets of statin drugs were identified and we focused on RORA due to its downregulation in lung cancer tissues and significant prognostic impact on patients with lung cancer. RORA, known as a circadian clock molecule, has been proved as a tumor suppressor in various cancers such as esophageal, prostate and gastric cancer (48–50). In lung cancer, RORA was downregulated in the tumor tissues from patients within early-stage and proved to inhibit the proliferation and migration of cancer cells (51). A mechanism investigation revealed RORA repressed hypoxia-inducible factor 1-alpha and its downstream genes to inhibit the malignant phenotype of lung adenocarcinoma cells (52). In accordance with these studies, our cellular assays demonstrated RORA overexpression dramatically inhibited the malignant characteristics of lung cancer cells. Moreover, RORA knockdown was found to partly antagonize the anti-cancer role of atorvastatin *in vitro.* These findings strongly suggest RORA may be a crucial target gene for statin-mediated anti-cancer effects. Our further analysis revealed RORA expression was correlated with infiltration of some immune cells such as B cells, CD4^+^ and CD8^+^ T cells, implying its potential impact on anti-cancer immunity. A recent bioinformatics analysis revealed RORA expression was correlated with infiltration of CD8^+^ T cells in patients with diffuse large B cell lymphoma (53). The melanoma patients with high RORA expression were found to have an improved prognosis after immunotherapy, and a RORA agonist combined anti-CTLA4 therapy enhanced T-cell-mediated anti-cancer immune response in animal experiments (54). Therefore, we speculate RORA may enhance ICI therapy partly through promoting infiltration of CD8^+^ T cells. For validating the speculation, the RORA expression and CD8^+^ T cell proportion were detected in the tumor tissues from patients with lung cancer who received ICI-based therapy. As a result, high RORA expression was significantly correlated with better OS and PFS in ICI-treated patients. More importantly, RORA expression and CD8^+^ T cell proportion were positively correlated in tumor tissues. These findings collectively suggest that statins enhance ICI therapy partly through their target genes, which may contribute to infiltration of immune cells.

Despite our novel findings, there are several limitations in our study. Firstly, the sample size of the retrospective cohort is limited. Secondly, the types of ICI drugs vary dramatically among patients. Thirdly, information of some crucial predictive biomarkers such as PD-L1, TMB and gene mutation is missing in some cases. Fourthly, statins are known to reduce blood lipid levels and prevent cardiovascular diseases. Actually, patients with serious coronary artery disease or hypertension who did not use statins may more likely to undergo non-cancerous deaths during anti-cancer therapy, which needs to be further investigated. Finally, despite the cellular validations, the impact of statin or RORA on immune cells remains poorly investigated, which is hoped to clarified based on well-designed animal experiments and single-cell sequencing data.

In conclusion, we found that statin use was significantly correlated with better prognosis in patients with lung cancer who received ICI therapy. RORA as a target gene of statins was correlated with infiltration of CD8^+^ T cells, and patients with high RORA expression had a better prognosis than those with low RORA expression after ICI therapy. These findings collectively support the beneficial role of statins in combination with ICI drugs in treating lung cancer.

## Data Availability

The original contributions presented in the study are included in the article/**Supplementary Material**. Further inquiries can be directed to the corresponding author.

## Supplementary material

Supplementary Figure 1 Comparison of therapy response between statin users and non-statin users in the first evaluation.

Supplementary Figure 2 Prognostic impact of statin use in the subgroups stratified by age<70 years **(A, B)** and age ≥70 years **(C, D)**.

Supplementary Figure 3 Prognostic impact of statin use in the subgroups stratified by smokers **(A, B)** and non-smokers **(C, D)**.

Supplementary Figure 4 Prognostic impact of statin use in the subgroups stratified by non-small cell lung cancer **(A, B)** and small cell lung cancer **(C, D)**.

Supplementary Figure 5 Prognostic impact of statin use in the subgroups stratified by stage III **(A, B)** and stage IV **(C, D)**.

Supplementary Figure 6 Impact of statin use on the overall survival **(A)** and progression-free survival **(B)** in the subgroups stratified by Rosuvastatin.

Supplementary Figure 7 Identification of prognostic statin target genes (STGs) in non-small cell lung cancer (NSCLC) patients. **(A)** Venn plot for the numbers of target genes of Atorvastatin, Rosuvastatin and Simvastatin in the online databases. **(B)** Venn plot of shared target genes of Atorvastatin, Rosuvastatin and Simvastatin. **(C)** Venn plot for identifying prognostic STGs for NSCLC using univariate cox analysis (HR<1 and p<0.05).

Supplementary Figure 8 Kaplan-Meier curves for the correlation of other statin target genes with overall survival in patients with lung cancer.

Supplementary Figure 9 RORA overexpression inhibits the malignant characteristics of lung cancer cells. **(A)** Western blot confirming the efficacy of RORA overexpression in lung cancer cells. **(B)** CCK-8 assay detecting the proliferation of lung cancer cells with RORA overexpression. **(C)** Colony formation assay detecting the formed clones of lung cancer cells with RORA overexpression. **(D, E)** Transwell assay detecting the invasion **(D)** and migration **(E)** of lung cancer cells with RORA overexpression. **p < 0.01, ***p < 0.001.

Supplementary Figure 10 Correlations of RORA with immune infiltration in non-small cell lung cancer patients. **(A)** Correlations between RORA expression and tumor-infiltrating immune cells. **(B)** Differences of immune subtypes between patients with high and low RORA expression.
