# Supplementary material for: Administration of statins is correlated with favourable prognosis in lung cancer patients receiving immune checkpoint inhibitors
Source: Front Immunol. 2025 Oct 6;16:1638677. doi: 10.3389/fimmu.2025.1638677 (PMC12535986; doi:10.3389/fimmu.2025.1638677)
Supplement: Supplementary Figure 1 — Comparison of therapy response between statin users and non-statin users in the first evaluation. [file DataSheet1.zip › Supplementary Table1.docx]

Supplementary Table 1. Details of immune subtypes in each sample.

| Sample | Subtype_Immune_Model_Based |
| --- | --- |
| TCGA-A5-A0GI-01 | Wound Healing (Immune C1) |
| TCGA-S9-A7J2-01 | Immunologically Quiet (Immune C5) |
| TCGA-EK-A2RE-01 | IFN-gamma Dominant (Immune C2) |
| TCGA-D5-5538-01 | IFN-gamma Dominant (Immune C2) |
| TCGA-F4-6854-01 | Wound Healing (Immune C1) |
| TCGA-AX-A2H7-01 | Wound Healing (Immune C1) |
| TCGA-C8-A1HL-01 | Wound Healing (Immune C1) |
| TCGA-EW-A2FS-01 | IFN-gamma Dominant (Immune C2) |
| TCGA-IR-A3L7-01 | Wound Healing (Immune C1) |
| TCGA-05-4420-01 | Wound Healing (Immune C1) |
| TCGA-2G-AAFZ-01 | Wound Healing (Immune C1) |
| TCGA-DX-A3U8-01 | Lymphocyte Depleted (Immune C4) |
| TCGA-GL-8500-01 | Lymphocyte Depleted (Immune C4) |
| TCGA-CC-A3MA-01 | Wound Healing (Immune C1) |
| TCGA-IN-7808-01 | IFN-gamma Dominant (Immune C2) |
| TCGA-CC-A9FW-01 | Inflammatory (Immune C3) |
| TCGA-AA-3982-01 | IFN-gamma Dominant (Immune C2) |
| TCGA-B9-A8YH-01 | Inflammatory (Immune C3) |
| TCGA-E1-A7YI-01 | Lymphocyte Depleted (Immune C4) |
| TCGA-B5-A1MY-01 | IFN-gamma Dominant (Immune C2) |
| TCGA-CH-5751-01 | IFN-gamma Dominant (Immune C2) |
| TCGA-A2-A3XX-01 | Wound Healing (Immune C1) |
| TCGA-DQ-5624-01 | IFN-gamma Dominant (Immune C2) |
| TCGA-KK-A7B3-01 | Inflammatory (Immune C3) |
| TCGA-HG-A2PA-01 | IFN-gamma Dominant (Immune C2) |
| TCGA-06-5412-01 | Lymphocyte Depleted (Immune C4) |
| TCGA-BP-4765-01 | Inflammatory (Immune C3) |
| TCGA-EJ-8468-01 | Inflammatory (Immune C3) |
| TCGA-CZ-5459-01 | Inflammatory (Immune C3) |
| TCGA-KK-A8I5-01 | Inflammatory (Immune C3) |
| TCGA-KP-A3W3-01 | IFN-gamma Dominant (Immune C2) |
| TCGA-IQ-A61O-01 | Wound Healing (Immune C1) |
| TCGA-BH-A18K-01 | Wound Healing (Immune C1) |
| TCGA-DD-AAW0-01 | Inflammatory (Immune C3) |
| TCGA-AG-A011-01 | Wound Healing (Immune C1) |
| TCGA-AA-3511-01 | IFN-gamma Dominant (Immune C2) |
| TCGA-Z7-A8R5-01 | Wound Healing (Immune C1) |
| TCGA-39-5031-01 | Wound Healing (Immune C1) |
| TCGA-ZG-A8QW-01 | Inflammatory (Immune C3) |
| TCGA-DU-7302-01 | Immunologically Quiet (Immune C5) |
| TCGA-DJ-A3UX-01 | Inflammatory (Immune C3) |
| TCGA-ZN-A9VQ-01 | Wound Healing (Immune C1) |
| TCGA-EI-6512-01 | Wound Healing (Immune C1) |
| TCGA-J4-A83K-01 | Inflammatory (Immune C3) |
| TCGA-2H-A9GF-01 | Wound Healing (Immune C1) |
| TCGA-HZ-7922-01 | TGF-beta Dominant (Immune C6) |
| TCGA-BJ-A28S-01 | Inflammatory (Immune C3) |
| TCGA-AG-3578-01 | Wound Healing (Immune C1) |
| TCGA-QG-A5YX-01 | Wound Healing (Immune C1) |
| TCGA-AA-3553-01 | Wound Healing (Immune C1) |
| TCGA-ZG-A9MC-01 | IFN-gamma Dominant (Immune C2) |
| TCGA-FE-A238-01 | IFN-gamma Dominant (Immune C2) |
| TCGA-DU-A7TI-01 | Lymphocyte Depleted (Immune C4) |
| TCGA-BR-8372-01 | IFN-gamma Dominant (Immune C2) |
| TCGA-HC-7742-01 | Inflammatory (Immune C3) |
| TCGA-64-1679-01 | Wound Healing (Immune C1) |
| TCGA-CV-5976-01 | IFN-gamma Dominant (Immune C2) |
| TCGA-25-1315-01 | IFN-gamma Dominant (Immune C2) |
| TCGA-R6-A8WC-01 | Wound Healing (Immune C1) |
| TCGA-GE-A2C6-01 | Inflammatory (Immune C3) |
| TCGA-CV-6948-01 | Wound Healing (Immune C1) |
| TCGA-D1-A1NW-01 | IFN-gamma Dominant (Immune C2) |
| TCGA-CJ-6028-01 | Inflammatory (Immune C3) |
| TCGA-24-2297-01 | IFN-gamma Dominant (Immune C2) |
| TCGA-CZ-5456-01 | Inflammatory (Immune C3) |
| TCGA-AA-3693-01 | Wound Healing (Immune C1) |
| TCGA-EM-A22Q-01 | Inflammatory (Immune C3) |
| TCGA-49-6744-01 | IFN-gamma Dominant (Immune C2) |
| TCGA-L5-A4ON-01 | Lymphocyte Depleted (Immune C4) |
| TCGA-IB-7891-01 | TGF-beta Dominant (Immune C6) |
| TCGA-ZN-A9VU-01 | IFN-gamma Dominant (Immune C2) |
| TCGA-Q3-A5QY-01 | Inflammatory (Immune C3) |
| TCGA-66-2794-01 | IFN-gamma Dominant (Immune C2) |
| TCGA-AJ-A3BI-01 | IFN-gamma Dominant (Immune C2) |
| TCGA-W2-A7UY-01 | Inflammatory (Immune C3) |
| TCGA-E3-A3E2-01 | Inflammatory (Immune C3) |
| TCGA-FB-A545-01 | IFN-gamma Dominant (Immune C2) |
| TCGA-G4-6320-01 | Wound Healing (Immune C1) |
| TCGA-EB-A41A-01 | Wound Healing (Immune C1) |
| TCGA-55-6982-01 | IFN-gamma Dominant (Immune C2) |
| TCGA-RC-A7SH-01 | Lymphocyte Depleted (Immune C4) |
| TCGA-CD-8528-01 | Lymphocyte Depleted (Immune C4) |
| TCGA-CM-6164-01 | Wound Healing (Immune C1) |
| TCGA-C5-A1ME-01 | Wound Healing (Immune C1) |
| TCGA-09-0369-01 | Wound Healing (Immune C1) |
| TCGA-E2-A2P6-01 | Inflammatory (Immune C3) |
| TCGA-VD-A8KF-01 | Lymphocyte Depleted (Immune C4) |
| TCGA-AX-A0IZ-01 | IFN-gamma Dominant (Immune C2) |
| TCGA-EL-A4JZ-01 | Inflammatory (Immune C3) |
| TCGA-M7-A722-01 | Lymphocyte Depleted (Immune C4) |
| TCGA-CV-A6K0-01 | IFN-gamma Dominant (Immune C2) |
| TCGA-13-1512-01 | IFN-gamma Dominant (Immune C2) |
| TCGA-BH-A1EN-01 | Lymphocyte Depleted (Immune C4) |
| TCGA-D1-A17D-01 | Inflammatory (Immune C3) |
| TCGA-AO-A1KT-01 | Wound Healing (Immune C1) |
| TCGA-N9-A4Q1-01 | Wound Healing (Immune C1) |
| TCGA-12-5299-01 | Lymphocyte Depleted (Immune C4) |
| TCGA-EM-A3AN-01 | Inflammatory (Immune C3) |
| TCGA-29-1761-01 | IFN-gamma Dominant (Immune C2) |
| TCGA-BJ-A0ZJ-01 | Inflammatory (Immune C3) |
| TCGA-P5-A5EX-01 | Inflammatory (Immune C3) |
| TCGA-DJ-A4V2-01 | Inflammatory (Immune C3) |
| TCGA-HT-8563-01 | Lymphocyte Depleted (Immune C4) |
| TCGA-J8-A3O2-01 | Inflammatory (Immune C3) |
| TCGA-E9-A3HO-01 | IFN-gamma Dominant (Immune C2) |
| TCGA-AX-A1CA-01 | IFN-gamma Dominant (Immune C2) |
| TCGA-A6-A567-01 | Wound Healing (Immune C1) |
| TCGA-58-A46N-01 | Wound Healing (Immune C1) |
| TCGA-B5-A1MR-01 | IFN-gamma Dominant (Immune C2) |
| TCGA-A3-3383-01 | IFN-gamma Dominant (Immune C2) |
| TCGA-NJ-A4YI-01 | Inflammatory (Immune C3) |
| TCGA-D8-A1XJ-01 | Wound Healing (Immune C1) |
| TCGA-VT-A80J-01 | IFN-gamma Dominant (Immune C2) |
| TCGA-Q1-A73Q-01 | IFN-gamma Dominant (Immune C2) |
| TCGA-AG-3587-01 | IFN-gamma Dominant (Immune C2) |
| TCGA-BQ-5880-01 | Inflammatory (Immune C3) |
| TCGA-75-7027-01 | Wound Healing (Immune C1) |
| TCGA-BA-6870-01 | IFN-gamma Dominant (Immune C2) |
| TCGA-73-4666-01 | IFN-gamma Dominant (Immune C2) |
| TCGA-MV-A51V-01 | Wound Healing (Immune C1) |
| TCGA-77-A5G6-01 | Wound Healing (Immune C1) |
| TCGA-5P-A9K4-01 | Inflammatory (Immune C3) |
| TCGA-VD-A8KE-01 | Lymphocyte Depleted (Immune C4) |
| TCGA-56-A4BX-01 | Wound Healing (Immune C1) |
| TCGA-E9-A3QA-01 | IFN-gamma Dominant (Immune C2) |
| TCGA-BR-A4J9-01 | Inflammatory (Immune C3) |
| TCGA-CV-6955-01 | IFN-gamma Dominant (Immune C2) |
| TCGA-P3-A5Q6-01 | Wound Healing (Immune C1) |
| TCGA-FG-A60K-01 | Immunologically Quiet (Immune C5) |
| TCGA-D7-A4Z0-01 | Inflammatory (Immune C3) |
| TCGA-QK-A64Z-01 | IFN-gamma Dominant (Immune C2) |
| TCGA-2J-AABI-01 | Wound Healing (Immune C1) |
| TCGA-AC-A2FK-01 | Inflammatory (Immune C3) |
| TCGA-VP-A879-01 | Inflammatory (Immune C3) |
| TCGA-29-A5NZ-01 | Wound Healing (Immune C1) |
| TCGA-B5-A1MV-01 | Wound Healing (Immune C1) |
| TCGA-DV-A4W0-01 | Inflammatory (Immune C3) |
| TCGA-37-4129-01 | Lymphocyte Depleted (Immune C4) |
| TCGA-DD-AAVZ-01 | Lymphocyte Depleted (Immune C4) |
| TCGA-D5-6534-01 | TGF-beta Dominant (Immune C6) |
| TCGA-DJ-A13R-01 | Inflammatory (Immune C3) |
| TCGA-E9-A1R4-01 | IFN-gamma Dominant (Immune C2) |
| TCGA-3U-A98F-01 | Lymphocyte Depleted (Immune C4) |
| TCGA-77-8145-01 | IFN-gamma Dominant (Immune C2) |
| TCGA-LL-A740-01 | Wound Healing (Immune C1) |
| TCGA-F5-6863-01 | Wound Healing (Immune C1) |
| TCGA-06-2565-01 | Lymphocyte Depleted (Immune C4) |
| TCGA-33-AASD-01 | Wound Healing (Immune C1) |
| TCGA-ET-A3BS-01 | Inflammatory (Immune C3) |
| TCGA-DM-A1D4-01 | Wound Healing (Immune C1) |
| TCGA-22-5478-01 | Wound Healing (Immune C1) |
| TCGA-MN-A4N1-01 | Wound Healing (Immune C1) |
| TCGA-LN-A4A8-01 | IFN-gamma Dominant (Immune C2) |
| TCGA-55-7911-01 | IFN-gamma Dominant (Immune C2) |
| TCGA-EY-A1GC-01 | IFN-gamma Dominant (Immune C2) |
| TCGA-ED-A627-01 | Inflammatory (Immune C3) |
| TCGA-N6-A4VC-01 | Wound Healing (Immune C1) |
| TCGA-16-0846-01 | Lymphocyte Depleted (Immune C4) |
| TCGA-C8-A3M8-01 | Wound Healing (Immune C1) |
| TCGA-BH-A0BM-01 | Inflammatory (Immune C3) |
| TCGA-DM-A0X9-01 | Wound Healing (Immune C1) |
| TCGA-AY-A69D-01 | Wound Healing (Immune C1) |
| TCGA-AN-A0XW-01 | Wound Healing (Immune C1) |
| TCGA-55-A4DG-01 | Inflammatory (Immune C3) |
| TCGA-RW-A684-01 | Lymphocyte Depleted (Immune C4) |
| TCGA-XD-AAUL-01 | Wound Healing (Immune C1) |
| TCGA-AA-3667-01 | Wound Healing (Immune C1) |
| TCGA-ET-A2N4-01 | Inflammatory (Immune C3) |
| TCGA-58-A46J-01 | IFN-gamma Dominant (Immune C2) |
| TCGA-24-1551-01 | IFN-gamma Dominant (Immune C2) |
| TCGA-06-2561-01 | Lymphocyte Depleted (Immune C4) |
| TCGA-WC-A88A-01 | Lymphocyte Depleted (Immune C4) |
| TCGA-DD-AAVV-01 | Inflammatory (Immune C3) |
| TCGA-ZJ-A8QQ-01 | IFN-gamma Dominant (Immune C2) |
| TCGA-37-4133-01 | IFN-gamma Dominant (Immune C2) |
| TCGA-77-8146-01 | Wound Healing (Immune C1) |
| TCGA-2G-AAHN-01 | IFN-gamma Dominant (Immune C2) |
| TCGA-L9-A5IP-01 | Wound Healing (Immune C1) |
| TCGA-CJ-5672-01 | Inflammatory (Immune C3) |
| TCGA-73-4662-01 | Inflammatory (Immune C3) |
| TCGA-3U-A98I-01 | TGF-beta Dominant (Immune C6) |
| TCGA-CQ-5327-01 | IFN-gamma Dominant (Immune C2) |
| TCGA-FI-A2F4-01 | IFN-gamma Dominant (Immune C2) |
| TCGA-E2-A1LL-01 | Wound Healing (Immune C1) |
| TCGA-FD-A3SR-01 | IFN-gamma Dominant (Immune C2) |
| TCGA-KK-A8II-01 | Lymphocyte Depleted (Immune C4) |
| TCGA-BH-A28O-01 | Inflammatory (Immune C3) |
| TCGA-XV-AAZW-01 | Wound Healing (Immune C1) |
| TCGA-BJ-A0Z2-01 | Inflammatory (Immune C3) |
| TCGA-DK-A6B0-01 | Lymphocyte Depleted (Immune C4) |
| TCGA-97-7553-01 | Inflammatory (Immune C3) |
| TCGA-ET-A2N0-01 | Inflammatory (Immune C3) |
| TCGA-YB-A89D-01 | Wound Healing (Immune C1) |
| TCGA-CH-5752-01 | Inflammatory (Immune C3) |
| TCGA-77-7142-01 | IFN-gamma Dominant (Immune C2) |
| TCGA-CR-7374-01 | IFN-gamma Dominant (Immune C2) |
| TCGA-KS-A4I1-01 | Inflammatory (Immune C3) |
| TCGA-60-2719-01 | IFN-gamma Dominant (Immune C2) |
| TCGA-VR-A8Q7-01 | Wound Healing (Immune C1) |
| TCGA-A7-A4SC-01 | Inflammatory (Immune C3) |
| TCGA-YU-A90Y-01 | IFN-gamma Dominant (Immune C2) |
| TCGA-FD-A5BS-01 | IFN-gamma Dominant (Immune C2) |
| TCGA-IG-A3Y9-01 | IFN-gamma Dominant (Immune C2) |
| TCGA-VR-A8EY-01 | Wound Healing (Immune C1) |
| TCGA-J7-6720-01 | Inflammatory (Immune C3) |
| TCGA-E9-A24A-01 | Lymphocyte Depleted (Immune C4) |
| TCGA-49-AARE-01 | Wound Healing (Immune C1) |
| TCGA-BC-4073-01 | IFN-gamma Dominant (Immune C2) |
| TCGA-A6-2671-01 | IFN-gamma Dominant (Immune C2) |
| TCGA-DK-A1AD-01 | IFN-gamma Dominant (Immune C2) |
| TCGA-B6-A400-01 | IFN-gamma Dominant (Immune C2) |
| TCGA-EM-A2OW-01 | Inflammatory (Immune C3) |
| TCGA-XF-AAMQ-01 | IFN-gamma Dominant (Immune C2) |
| TCGA-BB-A5HY-01 | IFN-gamma Dominant (Immune C2) |
| TCGA-AG-3742-01 | Wound Healing (Immune C1) |
| TCGA-WB-A816-01 | Inflammatory (Immune C3) |
| TCGA-EA-A410-01 | Wound Healing (Immune C1) |
| TCGA-CN-4722-01 | Wound Healing (Immune C1) |
| TCGA-AN-A041-01 | Wound Healing (Immune C1) |
| TCGA-13-0899-01 | Wound Healing (Immune C1) |
| TCGA-AY-A8YK-01 | Wound Healing (Immune C1) |
| TCGA-QA-A7B7-01 | Lymphocyte Depleted (Immune C4) |
| TCGA-A5-AB3J-01 | Inflammatory (Immune C3) |
| TCGA-50-8460-01 | Inflammatory (Immune C3) |
| TCGA-PG-A917-01 | IFN-gamma Dominant (Immune C2) |
| TCGA-IB-7889-01 | TGF-beta Dominant (Immune C6) |
| TCGA-EM-A3SY-01 | Lymphocyte Depleted (Immune C4) |
| TCGA-ZG-A9L1-01 | Inflammatory (Immune C3) |
| TCGA-CN-5361-01 | IFN-gamma Dominant (Immune C2) |
| TCGA-EX-A3L1-01 | Wound Healing (Immune C1) |
| TCGA-50-5933-01 | TGF-beta Dominant (Immune C6) |
| TCGA-EM-A3O7-01 | Inflammatory (Immune C3) |
| TCGA-IE-A4EH-01 | Wound Healing (Immune C1) |
| TCGA-A2-A04Y-01 | IFN-gamma Dominant (Immune C2) |
| TCGA-43-3394-01 | Wound Healing (Immune C1) |
| TCGA-ZF-AA4T-01 | IFN-gamma Dominant (Immune C2) |
| TCGA-MQ-A6BQ-01 | Lymphocyte Depleted (Immune C4) |
| TCGA-E2-A1LH-01 | IFN-gamma Dominant (Immune C2) |
| TCGA-ET-A39P-01 | Inflammatory (Immune C3) |
| TCGA-78-7155-01 | Wound Healing (Immune C1) |
| TCGA-2G-AAM4-01 | Wound Healing (Immune C1) |
| TCGA-AG-3727-01 | Wound Healing (Immune C1) |
| TCGA-E7-A3X6-01 | IFN-gamma Dominant (Immune C2) |
| TCGA-5M-AAT4-01 | Wound Healing (Immune C1) |
| TCGA-24-1474-01 | Lymphocyte Depleted (Immune C4) |
| TCGA-3H-AB3U-01 | Wound Healing (Immune C1) |
| TCGA-CM-6167-01 | Wound Healing (Immune C1) |
| TCGA-IZ-8196-01 | Inflammatory (Immune C3) |
| TCGA-CR-7370-01 | Wound Healing (Immune C1) |
| TCGA-BQ-5888-01 | Inflammatory (Immune C3) |
| TCGA-B6-A0RH-01 | IFN-gamma Dominant (Immune C2) |
| TCGA-EL-A3ZG-01 | Inflammatory (Immune C3) |
| TCGA-EL-A3CR-01 | Inflammatory (Immune C3) |
| TCGA-AA-3520-01 | Wound Healing (Immune C1) |
| TCGA-A8-A07W-01 | Wound Healing (Immune C1) |
| TCGA-QR-A6GY-01 | Lymphocyte Depleted (Immune C4) |
| TCGA-VD-A8KA-01 | Lymphocyte Depleted (Immune C4) |
| TCGA-YL-A8HJ-01 | Inflammatory (Immune C3) |
| TCGA-64-1677-01 | IFN-gamma Dominant (Immune C2) |
| TCGA-AU-6004-01 | IFN-gamma Dominant (Immune C2) |
| TCGA-GU-A762-01 | IFN-gamma Dominant (Immune C2) |
| TCGA-2Z-A9JK-01 | Inflammatory (Immune C3) |
| TCGA-CS-5397-01 | Lymphocyte Depleted (Immune C4) |
| TCGA-AX-A3G8-01 | Wound Healing (Immune C1) |
| TCGA-B5-A11Q-01 | Inflammatory (Immune C3) |
| TCGA-AA-3812-01 | Inflammatory (Immune C3) |
| TCGA-AR-A1AS-01 | Lymphocyte Depleted (Immune C4) |
| TCGA-CZ-4860-01 | Lymphocyte Depleted (Immune C4) |
| TCGA-DD-AACA-01 | Inflammatory (Immune C3) |
| TCGA-09-2048-01 | Wound Healing (Immune C1) |
| TCGA-HU-A4GQ-01 | IFN-gamma Dominant (Immune C2) |
| TCGA-E1-5322-01 | Immunologically Quiet (Immune C5) |
| TCGA-73-4670-01 | Wound Healing (Immune C1) |
| TCGA-ZG-A9L5-01 | Inflammatory (Immune C3) |
| TCGA-CN-5365-01 | IFN-gamma Dominant (Immune C2) |
| TCGA-MT-A7BN-01 | Wound Healing (Immune C1) |
| TCGA-BR-4371-01 | IFN-gamma Dominant (Immune C2) |
| TCGA-B0-5700-01 | Inflammatory (Immune C3) |
| TCGA-97-7552-01 | Inflammatory (Immune C3) |
| TCGA-BF-A5ER-01 | Lymphocyte Depleted (Immune C4) |
| TCGA-JW-A5VI-01 | IFN-gamma Dominant (Immune C2) |
| TCGA-XK-AAJT-01 | Lymphocyte Depleted (Immune C4) |
| TCGA-56-A4BW-01 | IFN-gamma Dominant (Immune C2) |
| TCGA-56-8308-01 | Wound Healing (Immune C1) |
| TCGA-D1-A102-01 | Wound Healing (Immune C1) |
| TCGA-CV-6956-01 | IFN-gamma Dominant (Immune C2) |
| TCGA-AA-3854-01 | Wound Healing (Immune C1) |
| TCGA-AP-A056-01 | Wound Healing (Immune C1) |
| TCGA-DQ-5631-01 | Wound Healing (Immune C1) |
| TCGA-85-8353-01 | Wound Healing (Immune C1) |
| TCGA-36-1580-01 | Wound Healing (Immune C1) |
| TCGA-OL-A66O-01 | IFN-gamma Dominant (Immune C2) |
| TCGA-HP-A5N0-01 | Inflammatory (Immune C3) |
| TCGA-CR-7369-01 | IFN-gamma Dominant (Immune C2) |
| TCGA-55-A48Z-01 | Wound Healing (Immune C1) |
| TCGA-AP-A1E3-01 | Wound Healing (Immune C1) |
| TCGA-SC-A6LP-01 | Wound Healing (Immune C1) |
| TCGA-HT-8558-01 | Immunologically Quiet (Immune C5) |
| TCGA-FD-A43S-01 | Inflammatory (Immune C3) |
| TCGA-5B-A90C-01 | IFN-gamma Dominant (Immune C2) |
| TCGA-EW-A1P0-01 | Lymphocyte Depleted (Immune C4) |
| TCGA-A5-A0GX-01 | Inflammatory (Immune C3) |
| TCGA-QK-A6IG-01 | IFN-gamma Dominant (Immune C2) |
| TCGA-22-4604-01 | Wound Healing (Immune C1) |
| TCGA-BT-A42F-01 | IFN-gamma Dominant (Immune C2) |
| TCGA-61-1900-01 | Wound Healing (Immune C1) |
| TCGA-Y8-A894-01 | Inflammatory (Immune C3) |
| TCGA-S9-A6U8-01 | Immunologically Quiet (Immune C5) |
| TCGA-DC-5337-01 | Wound Healing (Immune C1) |
| TCGA-CV-7261-01 | Wound Healing (Immune C1) |
| TCGA-DJ-A3V7-01 | Inflammatory (Immune C3) |
| TCGA-KL-8345-01 | Immunologically Quiet (Immune C5) |
| TCGA-BP-4998-01 | Inflammatory (Immune C3) |
| TCGA-ZH-A8Y5-01 | Inflammatory (Immune C3) |
| TCGA-EL-A3T9-01 | TGF-beta Dominant (Immune C6) |
| TCGA-OR-A5J2-01 | Inflammatory (Immune C3) |
| TCGA-BR-8363-01 | IFN-gamma Dominant (Immune C2) |
| TCGA-T2-A6X2-01 | IFN-gamma Dominant (Immune C2) |
| TCGA-CR-6492-01 | IFN-gamma Dominant (Immune C2) |
| TCGA-G2-A3IB-01 | IFN-gamma Dominant (Immune C2) |
| TCGA-EW-A1PC-01 | IFN-gamma Dominant (Immune C2) |
| TCGA-BG-A0M3-01 | Wound Healing (Immune C1) |
| TCGA-GM-A2DA-01 | Wound Healing (Immune C1) |
| TCGA-CJ-5675-01 | Inflammatory (Immune C3) |
| TCGA-CU-A3KJ-01 | Wound Healing (Immune C1) |
| TCGA-A7-A0CH-01 | Wound Healing (Immune C1) |
| TCGA-CM-6168-01 | Wound Healing (Immune C1) |
| TCGA-E9-A2JS-01 | IFN-gamma Dominant (Immune C2) |
| TCGA-EA-A1QT-01 | IFN-gamma Dominant (Immune C2) |
| TCGA-L6-A4EP-01 | Inflammatory (Immune C3) |
| TCGA-W4-A7U4-01 | IFN-gamma Dominant (Immune C2) |
| TCGA-4E-A92E-01 | IFN-gamma Dominant (Immune C2) |
| TCGA-HQ-A5ND-01 | IFN-gamma Dominant (Immune C2) |
| TCGA-EL-A3ZL-01 | Inflammatory (Immune C3) |
| TCGA-XY-A8S3-01 | IFN-gamma Dominant (Immune C2) |
| TCGA-75-7031-01 | IFN-gamma Dominant (Immune C2) |
| TCGA-EW-A6SB-01 | IFN-gamma Dominant (Immune C2) |
| TCGA-A5-A1OG-01 | IFN-gamma Dominant (Immune C2) |
| TCGA-44-A47A-01 | IFN-gamma Dominant (Immune C2) |
| TCGA-B6-A0WV-01 | Wound Healing (Immune C1) |
| TCGA-WB-A81J-01 | Inflammatory (Immune C3) |
| TCGA-2G-AAL5-01 | Wound Healing (Immune C1) |
| TCGA-HE-7129-01 | Inflammatory (Immune C3) |
| TCGA-05-4398-01 | IFN-gamma Dominant (Immune C2) |
| TCGA-HD-7753-01 | IFN-gamma Dominant (Immune C2) |
| TCGA-FI-A2EU-01 | IFN-gamma Dominant (Immune C2) |
| TCGA-BA-5559-01 | IFN-gamma Dominant (Immune C2) |
| TCGA-KK-A59X-01 | Lymphocyte Depleted (Immune C4) |
| TCGA-LN-A4MQ-01 | Wound Healing (Immune C1) |
| TCGA-UL-AAZ6-01 | IFN-gamma Dominant (Immune C2) |
| TCGA-B6-A0RG-01 | IFN-gamma Dominant (Immune C2) |
| TCGA-SP-A6QC-01 | Lymphocyte Depleted (Immune C4) |
| TCGA-SJ-A6ZI-01 | IFN-gamma Dominant (Immune C2) |
| TCGA-67-3773-01 | Inflammatory (Immune C3) |
| TCGA-24-1562-01 | Wound Healing (Immune C1) |
| TCGA-VS-AA62-01 | IFN-gamma Dominant (Immune C2) |
| TCGA-EM-A3SU-01 | Inflammatory (Immune C3) |
| TCGA-HT-7874-01 | Immunologically Quiet (Immune C5) |
| TCGA-77-8007-01 | Wound Healing (Immune C1) |
| TCGA-D1-A179-01 | Wound Healing (Immune C1) |
| TCGA-FK-A3SG-01 | Inflammatory (Immune C3) |
| TCGA-77-7463-01 | Wound Healing (Immune C1) |
| TCGA-S9-A6TV-01 | Lymphocyte Depleted (Immune C4) |
| TCGA-BH-A6R9-01 | Wound Healing (Immune C1) |
| TCGA-39-5039-01 | Wound Healing (Immune C1) |
| TCGA-UC-A7PG-01 | Wound Healing (Immune C1) |
| TCGA-DK-A3IS-01 | Wound Healing (Immune C1) |
| TCGA-BR-4363-01 | IFN-gamma Dominant (Immune C2) |
| TCGA-CV-6943-01 | IFN-gamma Dominant (Immune C2) |
| TCGA-64-5781-01 | Wound Healing (Immune C1) |
| TCGA-L5-A4OX-01 | IFN-gamma Dominant (Immune C2) |
| TCGA-UY-A8OD-01 | Wound Healing (Immune C1) |
| TCGA-EO-A2CH-01 | IFN-gamma Dominant (Immune C2) |
| TCGA-CQ-7068-01 | IFN-gamma Dominant (Immune C2) |
| TCGA-VM-A8CD-01 | Lymphocyte Depleted (Immune C4) |
| TCGA-D8-A1JN-01 | Inflammatory (Immune C3) |
| TCGA-CV-5444-01 | IFN-gamma Dominant (Immune C2) |
| TCGA-AR-A24P-01 | Wound Healing (Immune C1) |
| TCGA-EA-A97N-01 | IFN-gamma Dominant (Immune C2) |
| TCGA-LN-A49K-01 | IFN-gamma Dominant (Immune C2) |
| TCGA-SX-A7SM-01 | Lymphocyte Depleted (Immune C4) |
| TCGA-DJ-A4V5-01 | Inflammatory (Immune C3) |
| TCGA-DD-AAEI-01 | Lymphocyte Depleted (Immune C4) |
| TCGA-AZ-4615-01 | IFN-gamma Dominant (Immune C2) |
| TCGA-06-0132-01 | Lymphocyte Depleted (Immune C4) |
| TCGA-VR-A8EO-01 | Wound Healing (Immune C1) |
| TCGA-HT-7616-01 | Lymphocyte Depleted (Immune C4) |
| TCGA-CR-7395-01 | IFN-gamma Dominant (Immune C2) |
| TCGA-DJ-A2PS-01 | Inflammatory (Immune C3) |
| TCGA-BR-7196-01 | IFN-gamma Dominant (Immune C2) |
| TCGA-AA-A02H-01 | Wound Healing (Immune C1) |
| TCGA-AP-A0LL-01 | Inflammatory (Immune C3) |
| TCGA-BP-4777-01 | Inflammatory (Immune C3) |
| TCGA-24-2290-01 | Lymphocyte Depleted (Immune C4) |
| TCGA-ET-A39J-01 | Lymphocyte Depleted (Immune C4) |
| TCGA-E9-A1N9-01 | Wound Healing (Immune C1) |
| TCGA-B6-A0I9-01 | Wound Healing (Immune C1) |
| TCGA-CN-6022-01 | Wound Healing (Immune C1) |
| TCGA-E8-A242-01 | Inflammatory (Immune C3) |
| TCGA-DJ-A2Q5-01 | Inflammatory (Immune C3) |
| TCGA-DW-7963-01 | Inflammatory (Immune C3) |
| TCGA-E2-A1L9-01 | IFN-gamma Dominant (Immune C2) |
| TCGA-NC-A5HE-01 | IFN-gamma Dominant (Immune C2) |
| TCGA-BP-4797-01 | Inflammatory (Immune C3) |
| TCGA-E5-A4U1-01 | Wound Healing (Immune C1) |
| TCGA-OE-A75W-01 | Wound Healing (Immune C1) |
| TCGA-DX-AB3C-01 | Wound Healing (Immune C1) |
| TCGA-BP-4762-01 | Inflammatory (Immune C3) |
| TCGA-AR-A1AP-01 | IFN-gamma Dominant (Immune C2) |
| TCGA-KT-A7W1-01 | Lymphocyte Depleted (Immune C4) |
| TCGA-44-3919-01 | IFN-gamma Dominant (Immune C2) |
| TCGA-39-5035-01 | IFN-gamma Dominant (Immune C2) |
| TCGA-CV-6952-01 | IFN-gamma Dominant (Immune C2) |
| TCGA-BR-4367-01 | IFN-gamma Dominant (Immune C2) |
| TCGA-CN-A63T-01 | IFN-gamma Dominant (Immune C2) |
| TCGA-BH-A0H5-01 | TGF-beta Dominant (Immune C6) |
| TCGA-OK-A5Q2-01 | Inflammatory (Immune C3) |
| TCGA-B5-A0K9-01 | Wound Healing (Immune C1) |
| TCGA-CV-A45P-01 | IFN-gamma Dominant (Immune C2) |
| TCGA-BH-A0E6-01 | Wound Healing (Immune C1) |
| TCGA-D8-A1JA-01 | Lymphocyte Depleted (Immune C4) |
| TCGA-56-7221-01 | IFN-gamma Dominant (Immune C2) |
| TCGA-4G-AAZT-01 | Inflammatory (Immune C3) |
| TCGA-VR-A8EX-01 | IFN-gamma Dominant (Immune C2) |
| TCGA-B0-4844-01 | IFN-gamma Dominant (Immune C2) |
| TCGA-P5-A5F2-01 | Immunologically Quiet (Immune C5) |
| TCGA-BP-4352-01 | Wound Healing (Immune C1) |
| TCGA-99-8032-01 | Wound Healing (Immune C1) |
| TCGA-BH-A0BW-01 | IFN-gamma Dominant (Immune C2) |
| TCGA-DW-7841-01 | Inflammatory (Immune C3) |
| TCGA-H7-8502-01 | IFN-gamma Dominant (Immune C2) |
| TCGA-CV-A6JN-01 | IFN-gamma Dominant (Immune C2) |
| TCGA-OR-A5LN-01 | Inflammatory (Immune C3) |
| TCGA-BH-A0HB-01 | IFN-gamma Dominant (Immune C2) |
| TCGA-AG-3609-01 | IFN-gamma Dominant (Immune C2) |
| TCGA-29-1695-01 | Lymphocyte Depleted (Immune C4) |
| TCGA-H2-A2K9-01 | Inflammatory (Immune C3) |
| TCGA-G9-7509-01 | Inflammatory (Immune C3) |
| TCGA-FV-A2QR-01 | Inflammatory (Immune C3) |
| TCGA-19-2629-01 | Lymphocyte Depleted (Immune C4) |
| TCGA-B2-4098-01 | Inflammatory (Immune C3) |
| TCGA-D7-8575-01 | Inflammatory (Immune C3) |
| TCGA-HT-7607-01 | Immunologically Quiet (Immune C5) |
| TCGA-CR-7380-01 | IFN-gamma Dominant (Immune C2) |
| TCGA-43-2576-01 | Wound Healing (Immune C1) |
| TCGA-DJ-A2PW-01 | IFN-gamma Dominant (Immune C2) |
| TCGA-N8-A56S-01 | Wound Healing (Immune C1) |
| TCGA-BR-8367-01 | Wound Healing (Immune C1) |
| TCGA-ET-A3BW-01 | Inflammatory (Immune C3) |
| TCGA-06-0238-01 | Lymphocyte Depleted (Immune C4) |
| TCGA-FB-AAQ3-01 | IFN-gamma Dominant (Immune C2) |
| TCGA-CJ-5679-01 | Inflammatory (Immune C3) |
| TCGA-C5-A8YR-01 | IFN-gamma Dominant (Immune C2) |
| TCGA-AQ-A54O-01 | Wound Healing (Immune C1) |
| TCGA-V1-A9Z7-01 | Wound Healing (Immune C1) |
| TCGA-KL-8346-01 | Inflammatory (Immune C3) |
| TCGA-FE-A239-01 | Inflammatory (Immune C3) |
| TCGA-DQ-7594-01 | IFN-gamma Dominant (Immune C2) |
| TCGA-XF-A8HF-01 | Wound Healing (Immune C1) |
| TCGA-KQ-A41P-01 | Wound Healing (Immune C1) |
| TCGA-DX-A8BH-01 | IFN-gamma Dominant (Immune C2) |
| TCGA-DD-AACT-01 | Inflammatory (Immune C3) |
| TCGA-BL-A13I-01 | Lymphocyte Depleted (Immune C4) |
| TCGA-DX-A7EU-01 | Inflammatory (Immune C3) |
| TCGA-B6-A0WZ-01 | IFN-gamma Dominant (Immune C2) |
| TCGA-C8-A135-01 | Wound Healing (Immune C1) |
| TCGA-39-5027-01 | Wound Healing (Immune C1) |
| TCGA-HD-7917-01 | IFN-gamma Dominant (Immune C2) |
| TCGA-A5-A0VQ-01 | Wound Healing (Immune C1) |
| TCGA-22-5485-01 | Lymphocyte Depleted (Immune C4) |
| TCGA-EL-A4K1-01 | Inflammatory (Immune C3) |
| TCGA-H4-A2HQ-01 | IFN-gamma Dominant (Immune C2) |
| TCGA-AK-3427-01 | Lymphocyte Depleted (Immune C4) |
| TCGA-DJ-A2PX-01 | Inflammatory (Immune C3) |
| TCGA-BH-A0BJ-01 | Inflammatory (Immune C3) |
| TCGA-BH-A203-01 | Wound Healing (Immune C1) |
| TCGA-BG-A0M0-01 | Wound Healing (Immune C1) |
| TCGA-24-1419-01 | IFN-gamma Dominant (Immune C2) |
| TCGA-AG-3583-01 | Wound Healing (Immune C1) |
| TCGA-V4-A9F7-01 | Lymphocyte Depleted (Immune C4) |
| TCGA-GL-A9DD-01 | Inflammatory (Immune C3) |
| TCGA-13-A5FT-01 | IFN-gamma Dominant (Immune C2) |
| TCGA-WB-A81M-01 | Lymphocyte Depleted (Immune C4) |
| TCGA-HT-7873-01 | Immunologically Quiet (Immune C5) |
| TCGA-55-A491-01 | IFN-gamma Dominant (Immune C2) |
| TCGA-FY-A3NM-01 | Inflammatory (Immune C3) |
| TCGA-06-0749-01 | Lymphocyte Depleted (Immune C4) |
| TCGA-ND-A4WF-01 | Wound Healing (Immune C1) |
| TCGA-AZ-4681-01 | Wound Healing (Immune C1) |
| TCGA-46-6025-01 | IFN-gamma Dominant (Immune C2) |
| TCGA-FE-A236-01 | Lymphocyte Depleted (Immune C4) |
| TCGA-ZF-AA4W-01 | IFN-gamma Dominant (Immune C2) |
| TCGA-UY-A9PA-01 | IFN-gamma Dominant (Immune C2) |
| TCGA-AA-3968-01 | Wound Healing (Immune C1) |
| TCGA-BG-A0MO-01 | IFN-gamma Dominant (Immune C2) |
| TCGA-HT-7603-01 | Immunologically Quiet (Immune C5) |
| TCGA-AJ-A2QN-01 | Wound Healing (Immune C1) |
| TCGA-AJ-A3NF-01 | IFN-gamma Dominant (Immune C2) |
| TCGA-FU-A40J-01 | IFN-gamma Dominant (Immune C2) |
| TCGA-B9-4116-01 | Inflammatory (Immune C3) |
| TCGA-BH-A0C7-01 | IFN-gamma Dominant (Immune C2) |
| TCGA-50-6595-01 | IFN-gamma Dominant (Immune C2) |
| TCGA-DB-A64P-01 | Immunologically Quiet (Immune C5) |
| TCGA-BP-4981-01 | Inflammatory (Immune C3) |
| TCGA-VS-A9V5-01 | Wound Healing (Immune C1) |
| TCGA-AK-3428-01 | Inflammatory (Immune C3) |
| TCGA-CR-7373-01 | IFN-gamma Dominant (Immune C2) |
| TCGA-G4-6311-01 | Wound Healing (Immune C1) |
| TCGA-BH-A8FZ-01 | IFN-gamma Dominant (Immune C2) |
| TCGA-S3-AA17-01 | IFN-gamma Dominant (Immune C2) |
| TCGA-DF-A2L0-01 | IFN-gamma Dominant (Immune C2) |
| TCGA-CI-6619-01 | Wound Healing (Immune C1) |
| TCGA-B7-A5TI-01 | Wound Healing (Immune C1) |
| TCGA-D8-A13Z-01 | IFN-gamma Dominant (Immune C2) |
| TCGA-BA-5152-01 | IFN-gamma Dominant (Immune C2) |
| TCGA-DD-AAEE-01 | Lymphocyte Depleted (Immune C4) |
| TCGA-A4-7997-01 | Lymphocyte Depleted (Immune C4) |
| TCGA-DX-A48O-01 | Inflammatory (Immune C3) |
| TCGA-A7-A6VX-01 | IFN-gamma Dominant (Immune C2) |
| TCGA-VS-A8Q9-01 | IFN-gamma Dominant (Immune C2) |
| TCGA-60-2713-01 | Wound Healing (Immune C1) |
| TCGA-GU-A42Q-01 | Wound Healing (Immune C1) |
| TCGA-95-A4VP-01 | Inflammatory (Immune C3) |
| TCGA-BP-4340-01 | Inflammatory (Immune C3) |
| TCGA-HC-8261-01 | Inflammatory (Immune C3) |
| TCGA-85-A512-01 | Wound Healing (Immune C1) |
| TCGA-ET-A25P-01 | Inflammatory (Immune C3) |
| TCGA-DJ-A3VI-01 | Inflammatory (Immune C3) |
| TCGA-55-8302-01 | IFN-gamma Dominant (Immune C2) |
| TCGA-44-4112-01 | Wound Healing (Immune C1) |
| TCGA-EJ-A65E-01 | Inflammatory (Immune C3) |
| TCGA-AF-3911-01 | Wound Healing (Immune C1) |
| TCGA-UF-A7JJ-01 | IFN-gamma Dominant (Immune C2) |
| TCGA-05-4430-01 | IFN-gamma Dominant (Immune C2) |
| TCGA-AR-A255-01 | IFN-gamma Dominant (Immune C2) |
| TCGA-WB-A81F-01 | Inflammatory (Immune C3) |
| TCGA-V4-A9EQ-01 | Lymphocyte Depleted (Immune C4) |
| TCGA-HZ-7919-01 | Wound Healing (Immune C1) |
| TCGA-CN-5366-01 | IFN-gamma Dominant (Immune C2) |
| TCGA-AR-A1AT-01 | IFN-gamma Dominant (Immune C2) |
| TCGA-E5-A2PC-01 | IFN-gamma Dominant (Immune C2) |
| TCGA-AD-6899-01 | Wound Healing (Immune C1) |
| TCGA-YL-A8HM-01 | IFN-gamma Dominant (Immune C2) |
| TCGA-CD-A486-01 | IFN-gamma Dominant (Immune C2) |
| TCGA-UY-A9PE-01 | Wound Healing (Immune C1) |
| TCGA-CQ-7071-01 | IFN-gamma Dominant (Immune C2) |
| TCGA-D1-A3DG-01 | Wound Healing (Immune C1) |
| TCGA-VQ-A8PX-01 | IFN-gamma Dominant (Immune C2) |
| TCGA-XF-A9SV-01 | Wound Healing (Immune C1) |
| TCGA-24-1924-01 | IFN-gamma Dominant (Immune C2) |
| TCGA-DX-A6BG-01 | Wound Healing (Immune C1) |
| TCGA-E7-A6MF-01 | Inflammatory (Immune C3) |
| TCGA-MM-A84U-01 | Inflammatory (Immune C3) |
| TCGA-CV-6933-01 | Wound Healing (Immune C1) |
| TCGA-E2-A1B0-01 | Wound Healing (Immune C1) |
| TCGA-DS-A0VL-01 | IFN-gamma Dominant (Immune C2) |
| TCGA-63-6202-01 | IFN-gamma Dominant (Immune C2) |
| TCGA-BF-AAP2-01 | IFN-gamma Dominant (Immune C2) |
| TCGA-46-3767-01 | Wound Healing (Immune C1) |
| TCGA-2G-AALO-01 | Inflammatory (Immune C3) |
| TCGA-EM-A4FQ-01 | Inflammatory (Immune C3) |
| TCGA-B0-5095-01 | Inflammatory (Immune C3) |
| TCGA-CI-6622-01 | Wound Healing (Immune C1) |
| TCGA-BP-4167-01 | Inflammatory (Immune C3) |
| TCGA-AG-3601-01 | Wound Healing (Immune C1) |
| TCGA-ZP-A9D2-01 | IFN-gamma Dominant (Immune C2) |
| TCGA-BH-A18R-01 | Wound Healing (Immune C1) |
| TCGA-BA-7269-01 | Wound Healing (Immune C1) |
| TCGA-CG-4466-01 | Wound Healing (Immune C1) |
| TCGA-HU-A4G3-01 | Wound Healing (Immune C1) |
| TCGA-OR-A5KY-01 | Lymphocyte Depleted (Immune C4) |
| TCGA-NH-A6GC-01 | Wound Healing (Immune C1) |
| TCGA-DX-A48K-01 | Wound Healing (Immune C1) |
| TCGA-DD-A73B-01 | Lymphocyte Depleted (Immune C4) |
| TCGA-AC-A4ZE-01 | Inflammatory (Immune C3) |
| TCGA-P5-A5F1-01 | Lymphocyte Depleted (Immune C4) |
| TCGA-DK-AA6X-01 | IFN-gamma Dominant (Immune C2) |
| TCGA-EO-A22T-01 | Wound Healing (Immune C1) |
| TCGA-BR-7722-01 | Inflammatory (Immune C3) |
| TCGA-BI-A0VR-01 | IFN-gamma Dominant (Immune C2) |
| TCGA-A6-3808-01 | Wound Healing (Immune C1) |
| TCGA-33-AAS8-01 | Wound Healing (Immune C1) |
| TCGA-AR-A24H-01 | IFN-gamma Dominant (Immune C2) |
| TCGA-F4-6570-01 | Wound Healing (Immune C1) |
| TCGA-E8-A432-01 | Inflammatory (Immune C3) |
| TCGA-MZ-A5BI-01 | IFN-gamma Dominant (Immune C2) |
| TCGA-EJ-8474-01 | Inflammatory (Immune C3) |
| TCGA-DU-7011-01 | Immunologically Quiet (Immune C5) |
| TCGA-06-5414-01 | Lymphocyte Depleted (Immune C4) |
| TCGA-J9-A8CP-01 | Inflammatory (Immune C3) |
| TCGA-ZF-A9R9-01 | Inflammatory (Immune C3) |
| TCGA-P8-A6RX-01 | Inflammatory (Immune C3) |
| TCGA-JY-A6FE-01 | IFN-gamma Dominant (Immune C2) |
| TCGA-A8-A099-01 | Inflammatory (Immune C3) |
| TCGA-CM-6679-01 | Wound Healing (Immune C1) |
| TCGA-KL-8342-01 | Inflammatory (Immune C3) |
| TCGA-XF-A8HB-01 | TGF-beta Dominant (Immune C6) |
| TCGA-HT-8019-01 | Immunologically Quiet (Immune C5) |
| TCGA-HV-A5A4-01 | IFN-gamma Dominant (Immune C2) |
| TCGA-DC-6157-01 | Wound Healing (Immune C1) |
| TCGA-NQ-A638-01 | Wound Healing (Immune C1) |
| TCGA-VS-A9UU-01 | IFN-gamma Dominant (Immune C2) |
| TCGA-66-2790-01 | IFN-gamma Dominant (Immune C2) |
| TCGA-V1-A8WS-01 | Wound Healing (Immune C1) |
| TCGA-CE-A3MD-01 | Inflammatory (Immune C3) |
| TCGA-HU-A4GD-01 | IFN-gamma Dominant (Immune C2) |
| TCGA-94-7033-01 | IFN-gamma Dominant (Immune C2) |
| TCGA-VQ-A94U-01 | Wound Healing (Immune C1) |
| TCGA-LK-A4O0-01 | Wound Healing (Immune C1) |
| TCGA-XD-AAUG-01 | Wound Healing (Immune C1) |
| TCGA-FV-A4ZP-01 | Lymphocyte Depleted (Immune C4) |
| TCGA-KN-8435-01 | Inflammatory (Immune C3) |
| TCGA-P5-A77X-01 | Immunologically Quiet (Immune C5) |
| TCGA-NC-A5HI-01 | Wound Healing (Immune C1) |
| TCGA-ZJ-AAXN-01 | IFN-gamma Dominant (Immune C2) |
| TCGA-BP-4781-01 | Inflammatory (Immune C3) |
| TCGA-CV-6937-01 | Wound Healing (Immune C1) |
| TCGA-AJ-A3QS-01 | IFN-gamma Dominant (Immune C2) |
| TCGA-DD-AACS-01 | Lymphocyte Depleted (Immune C4) |
| TCGA-MT-A67F-01 | IFN-gamma Dominant (Immune C2) |
| TCGA-VS-A9U6-01 | IFN-gamma Dominant (Immune C2) |
| TCGA-EB-A6QZ-01 | Lymphocyte Depleted (Immune C4) |
| TCGA-5P-A9KF-01 | Inflammatory (Immune C3) |
| TCGA-EB-A1NK-01 | Wound Healing (Immune C1) |
| TCGA-RY-A843-01 | Immunologically Quiet (Immune C5) |
| TCGA-A8-A09R-01 | Wound Healing (Immune C1) |
| TCGA-DK-AA6S-01 | Wound Healing (Immune C1) |
| TCGA-V1-A9Z8-01 | Inflammatory (Immune C3) |
| TCGA-DD-A4NK-01 | Inflammatory (Immune C3) |
| TCGA-CM-4750-01 | IFN-gamma Dominant (Immune C2) |
| TCGA-VQ-A8PJ-01 | Wound Healing (Immune C1) |
| TCGA-KQ-A41S-01 | Wound Healing (Immune C1) |
| TCGA-4Z-AA7O-01 | Wound Healing (Immune C1) |
| TCGA-55-8507-01 | Wound Healing (Immune C1) |
| TCGA-DJ-A3UN-01 | Inflammatory (Immune C3) |
| TCGA-HW-7493-01 | Immunologically Quiet (Immune C5) |
| TCGA-BG-A0MC-01 | Inflammatory (Immune C3) |
| TCGA-D6-6516-01 | IFN-gamma Dominant (Immune C2) |
| TCGA-C5-A2LX-01 | IFN-gamma Dominant (Immune C2) |
| TCGA-CR-6471-01 | IFN-gamma Dominant (Immune C2) |
| TCGA-VM-A8CA-01 | Immunologically Quiet (Immune C5) |
| TCGA-D1-A17M-01 | IFN-gamma Dominant (Immune C2) |
| TCGA-D8-A27G-01 | Wound Healing (Immune C1) |
| TCGA-B0-5699-01 | Inflammatory (Immune C3) |
| TCGA-AA-3845-01 | IFN-gamma Dominant (Immune C2) |
| TCGA-A5-A0GH-01 | Wound Healing (Immune C1) |
| TCGA-XQ-A8TB-01 | Inflammatory (Immune C3) |
| TCGA-37-5819-01 | Wound Healing (Immune C1) |
| TCGA-DU-7015-01 | Immunologically Quiet (Immune C5) |
| TCGA-MQ-A4LJ-01 | Wound Healing (Immune C1) |
| TCGA-C5-A1M8-01 | IFN-gamma Dominant (Immune C2) |
| TCGA-C5-A7CJ-01 | IFN-gamma Dominant (Immune C2) |
| TCGA-AA-3980-01 | IFN-gamma Dominant (Immune C2) |
| TCGA-22-A5C4-01 | Wound Healing (Immune C1) |
| TCGA-HB-A2OT-01 | Lymphocyte Depleted (Immune C4) |
| TCGA-85-A4CL-01 | Wound Healing (Immune C1) |
| TCGA-TM-A7C5-01 | Immunologically Quiet (Immune C5) |
| TCGA-06-0184-01 | Lymphocyte Depleted (Immune C4) |
| TCGA-ZF-AA56-01 | IFN-gamma Dominant (Immune C2) |
| TCGA-BP-4801-01 | Inflammatory (Immune C3) |
| TCGA-D8-A1XC-01 | Inflammatory (Immune C3) |
| TCGA-DU-8162-01 | Immunologically Quiet (Immune C5) |
| TCGA-TM-A84O-01 | Immunologically Quiet (Immune C5) |
| TCGA-06-5410-01 | Lymphocyte Depleted (Immune C4) |
| TCGA-DC-6158-01 | IFN-gamma Dominant (Immune C2) |
| TCGA-HT-A4DS-01 | Lymphocyte Depleted (Immune C4) |
| TCGA-69-8254-01 | Inflammatory (Immune C3) |
| TCGA-AA-A00N-01 | Wound Healing (Immune C1) |
| TCGA-EM-A3FM-01 | Inflammatory (Immune C3) |
| TCGA-GH-A9DA-01 | Wound Healing (Immune C1) |
| TCGA-TN-A7HL-01 | IFN-gamma Dominant (Immune C2) |
| TCGA-3B-A9HT-01 | IFN-gamma Dominant (Immune C2) |
| TCGA-CN-4742-01 | IFN-gamma Dominant (Immune C2) |
| TCGA-AA-3984-01 | IFN-gamma Dominant (Immune C2) |
| TCGA-FY-A3R7-01 | Inflammatory (Immune C3) |
| TCGA-BH-A1FJ-01 | IFN-gamma Dominant (Immune C2) |
| TCGA-DD-AACX-01 | Lymphocyte Depleted (Immune C4) |
| TCGA-78-8640-01 | IFN-gamma Dominant (Immune C2) |
| TCGA-DJ-A3UY-01 | Inflammatory (Immune C3) |
| TCGA-EW-A1PE-01 | IFN-gamma Dominant (Immune C2) |
| TCGA-E2-A1II-01 | IFN-gamma Dominant (Immune C2) |
| TCGA-S3-AA14-01 | Wound Healing (Immune C1) |
| TCGA-CR-7392-01 | IFN-gamma Dominant (Immune C2) |
| TCGA-CV-7100-01 | IFN-gamma Dominant (Immune C2) |
| TCGA-60-2714-01 | IFN-gamma Dominant (Immune C2) |
| TCGA-2G-AAFO-01 | IFN-gamma Dominant (Immune C2) |
| TCGA-AR-A5QP-01 | Wound Healing (Immune C1) |
| TCGA-NF-A4WX-01 | Wound Healing (Immune C1) |
| TCGA-K6-A3WQ-01 | IFN-gamma Dominant (Immune C2) |
| TCGA-E9-A6HE-01 | IFN-gamma Dominant (Immune C2) |
| TCGA-BR-8291-01 | Inflammatory (Immune C3) |
| TCGA-DD-AADA-01 | Inflammatory (Immune C3) |
| TCGA-TQ-A7RN-01 | Immunologically Quiet (Immune C5) |
| TCGA-ZM-AA0B-01 | IFN-gamma Dominant (Immune C2) |
| TCGA-GL-A9DE-01 | Lymphocyte Depleted (Immune C4) |
| TCGA-QR-A70O-01 | Inflammatory (Immune C3) |
| TCGA-BH-A0HI-01 | Wound Healing (Immune C1) |
| TCGA-XE-AANI-01 | Wound Healing (Immune C1) |
| TCGA-ET-A3DT-01 | Inflammatory (Immune C3) |
| TCGA-F4-6806-01 | Wound Healing (Immune C1) |
| TCGA-DD-A1EI-01 | Wound Healing (Immune C1) |
| TCGA-AP-A0LG-01 | Wound Healing (Immune C1) |
| TCGA-NH-A50U-01 | IFN-gamma Dominant (Immune C2) |
| TCGA-BP-4790-01 | Inflammatory (Immune C3) |
| TCGA-AX-A1C8-01 | IFN-gamma Dominant (Immune C2) |
| TCGA-BP-5008-01 | Inflammatory (Immune C3) |
| TCGA-P8-A6RY-01 | Lymphocyte Depleted (Immune C4) |
| TCGA-AJ-A3BK-01 | IFN-gamma Dominant (Immune C2) |
| TCGA-CR-6480-01 | IFN-gamma Dominant (Immune C2) |
| TCGA-AA-3692-01 | Wound Healing (Immune C1) |
| TCGA-AF-2693-01 | Wound Healing (Immune C1) |
| TCGA-D5-6924-01 | Wound Healing (Immune C1) |
| TCGA-28-2513-01 | Lymphocyte Depleted (Immune C4) |
| TCGA-EW-A1P1-01 | Wound Healing (Immune C1) |
| TCGA-AA-A01R-01 | IFN-gamma Dominant (Immune C2) |
| TCGA-V4-A9EF-01 | Lymphocyte Depleted (Immune C4) |
| TCGA-JU-AAVI-01 | IFN-gamma Dominant (Immune C2) |
| TCGA-QR-A70R-01 | Inflammatory (Immune C3) |
| TCGA-OL-A66P-01 | IFN-gamma Dominant (Immune C2) |
| TCGA-NG-A4VW-01 | Wound Healing (Immune C1) |
| TCGA-CG-4462-01 | TGF-beta Dominant (Immune C6) |
| TCGA-A2-A1G4-01 | Wound Healing (Immune C1) |
| TCGA-E2-A105-01 | Wound Healing (Immune C1) |
| TCGA-55-7573-01 | Inflammatory (Immune C3) |
| TCGA-N5-A4RA-01 | Wound Healing (Immune C1) |
| TCGA-E6-A1LZ-01 | Wound Healing (Immune C1) |
| TCGA-L5-A4OR-01 | Wound Healing (Immune C1) |
| TCGA-EY-A2OM-01 | Wound Healing (Immune C1) |
| TCGA-CV-A45W-01 | IFN-gamma Dominant (Immune C2) |
| TCGA-EJ-5502-01 | Inflammatory (Immune C3) |
| TCGA-B0-5085-01 | Inflammatory (Immune C3) |
| TCGA-2L-AAQL-01 | TGF-beta Dominant (Immune C6) |
| TCGA-D1-A17F-01 | IFN-gamma Dominant (Immune C2) |
| TCGA-HC-8265-01 | Wound Healing (Immune C1) |
| TCGA-AD-6965-01 | Wound Healing (Immune C1) |
| TCGA-BJ-A3PR-01 | IFN-gamma Dominant (Immune C2) |
| TCGA-SH-A7BD-01 | IFN-gamma Dominant (Immune C2) |
| TCGA-H2-A422-01 | Inflammatory (Immune C3) |
| TCGA-EY-A549-01 | Inflammatory (Immune C3) |
| TCGA-DX-A6YU-01 | TGF-beta Dominant (Immune C6) |
| TCGA-P5-A735-01 | Immunologically Quiet (Immune C5) |
| TCGA-VQ-A928-01 | Wound Healing (Immune C1) |
| TCGA-D5-6931-01 | Wound Healing (Immune C1) |
| TCGA-DX-A6Z2-01 | Inflammatory (Immune C3) |
| TCGA-BS-A0V6-01 | IFN-gamma Dominant (Immune C2) |
| TCGA-BC-A10T-01 | Wound Healing (Immune C1) |
| TCGA-AA-3872-01 | Wound Healing (Immune C1) |
| TCGA-43-5668-01 | IFN-gamma Dominant (Immune C2) |
| TCGA-R8-A6ML-01 | Immunologically Quiet (Immune C5) |
| TCGA-FB-AAQ0-01 | IFN-gamma Dominant (Immune C2) |
| TCGA-BF-AAP1-01 | Lymphocyte Depleted (Immune C4) |
| TCGA-A2-A0EV-01 | IFN-gamma Dominant (Immune C2) |
| TCGA-HT-7691-01 | Immunologically Quiet (Immune C5) |
| TCGA-18-4083-01 | Wound Healing (Immune C1) |
| TCGA-FG-A6J1-01 | Lymphocyte Depleted (Immune C4) |
| TCGA-CV-7104-01 | IFN-gamma Dominant (Immune C2) |
| TCGA-EO-A22S-01 | Wound Healing (Immune C1) |
| TCGA-A8-A08A-01 | Inflammatory (Immune C3) |
| TCGA-D1-A17B-01 | Inflammatory (Immune C3) |
| TCGA-CD-8526-01 | IFN-gamma Dominant (Immune C2) |
| TCGA-BR-7703-01 | IFN-gamma Dominant (Immune C2) |
| TCGA-W8-A86G-01 | Inflammatory (Immune C3) |
| TCGA-AL-3471-01 | Inflammatory (Immune C3) |
| TCGA-MH-A854-01 | Wound Healing (Immune C1) |
| TCGA-YF-AA3M-01 | Wound Healing (Immune C1) |
| TCGA-76-4929-01 | Lymphocyte Depleted (Immune C4) |
| TCGA-2Y-A9H6-01 | Inflammatory (Immune C3) |
| TCGA-44-2656-01 | IFN-gamma Dominant (Immune C2) |
| TCGA-A6-5656-01 | Wound Healing (Immune C1) |
| TCGA-43-8115-01 | Wound Healing (Immune C1) |
| TCGA-3A-A9IX-01 | Inflammatory (Immune C3) |
| TCGA-EI-6884-01 | Wound Healing (Immune C1) |
| TCGA-DU-6402-01 | Lymphocyte Depleted (Immune C4) |
| TCGA-AG-3890-01 | Wound Healing (Immune C1) |
| TCGA-Q1-A73P-01 | IFN-gamma Dominant (Immune C2) |
| TCGA-39-5016-01 | IFN-gamma Dominant (Immune C2) |
| TCGA-HT-7684-01 | Immunologically Quiet (Immune C5) |
| TCGA-D8-A1XL-01 | IFN-gamma Dominant (Immune C2) |
| TCGA-E8-A413-01 | Inflammatory (Immune C3) |
| TCGA-BA-6871-01 | IFN-gamma Dominant (Immune C2) |
| TCGA-HT-8104-01 | Lymphocyte Depleted (Immune C4) |
| TCGA-CN-6017-01 | IFN-gamma Dominant (Immune C2) |
| TCGA-CC-A7IH-01 | Inflammatory (Immune C3) |
| TCGA-D7-A747-01 | IFN-gamma Dominant (Immune C2) |
| TCGA-A4-7585-01 | Inflammatory (Immune C3) |
| TCGA-TS-A7P6-01 | Wound Healing (Immune C1) |
| TCGA-BG-A0W2-01 | Wound Healing (Immune C1) |
| TCGA-DD-A4NG-01 | Inflammatory (Immune C3) |
| TCGA-BP-5170-01 | Inflammatory (Immune C3) |
| TCGA-FN-7833-01 | Lymphocyte Depleted (Immune C4) |
| TCGA-KN-8436-01 | Inflammatory (Immune C3) |
| TCGA-C5-A8XK-01 | IFN-gamma Dominant (Immune C2) |
| TCGA-43-A474-01 | Wound Healing (Immune C1) |
| TCGA-HT-7473-01 | Lymphocyte Depleted (Immune C4) |
| TCGA-06-0645-01 | Lymphocyte Depleted (Immune C4) |
| TCGA-33-AASI-01 | Wound Healing (Immune C1) |
| TCGA-DM-A1DA-01 | Wound Healing (Immune C1) |
| TCGA-IB-7647-01 | Wound Healing (Immune C1) |
| TCGA-BJ-A2N9-01 | Inflammatory (Immune C3) |
| TCGA-41-2572-01 | Lymphocyte Depleted (Immune C4) |
| TCGA-AG-3885-01 | Inflammatory (Immune C3) |
| TCGA-CF-A7I0-01 | Wound Healing (Immune C1) |
| TCGA-EK-A3GN-01 | IFN-gamma Dominant (Immune C2) |
| TCGA-WJ-A86L-01 | Lymphocyte Depleted (Immune C4) |
| TCGA-DX-AB2X-01 | Lymphocyte Depleted (Immune C4) |
| TCGA-MJ-A850-01 | Wound Healing (Immune C1) |
| TCGA-C5-A907-01 | IFN-gamma Dominant (Immune C2) |
| TCGA-49-4506-01 | IFN-gamma Dominant (Immune C2) |
| TCGA-B0-5713-01 | Inflammatory (Immune C3) |
| TCGA-BQ-5877-01 | Inflammatory (Immune C3) |
| TCGA-D1-A160-01 | Wound Healing (Immune C1) |
| TCGA-BR-6564-01 | IFN-gamma Dominant (Immune C2) |
| TCGA-B2-5633-01 | Inflammatory (Immune C3) |
| TCGA-BH-A0BZ-01 | IFN-gamma Dominant (Immune C2) |
| TCGA-XF-AAMX-01 | Wound Healing (Immune C1) |
| TCGA-OR-A5LR-01 | Inflammatory (Immune C3) |
| TCGA-A2-A0ER-01 | Wound Healing (Immune C1) |
| TCGA-IG-A8O2-01 | IFN-gamma Dominant (Immune C2) |
| TCGA-AX-A1C7-01 | IFN-gamma Dominant (Immune C2) |
| TCGA-TQ-A7RU-01 | Immunologically Quiet (Immune C5) |
| TCGA-BP-4982-01 | Inflammatory (Immune C3) |
| TCGA-JY-A938-01 | IFN-gamma Dominant (Immune C2) |
| TCGA-XF-A9T8-01 | IFN-gamma Dominant (Immune C2) |
| TCGA-2G-AAG0-01 | IFN-gamma Dominant (Immune C2) |
| TCGA-EO-A22X-01 | Wound Healing (Immune C1) |
| TCGA-DD-A4ND-01 | Inflammatory (Immune C3) |
| TCGA-B8-5162-01 | Inflammatory (Immune C3) |
| TCGA-66-2785-01 | IFN-gamma Dominant (Immune C2) |
| TCGA-25-1630-01 | Lymphocyte Depleted (Immune C4) |
| TCGA-OL-A66K-01 | Inflammatory (Immune C3) |
| TCGA-P4-A5E8-01 | Inflammatory (Immune C3) |
| TCGA-Q9-A6FW-01 | Wound Healing (Immune C1) |
| TCGA-IN-7806-01 | Inflammatory (Immune C3) |
| TCGA-36-1574-01 | IFN-gamma Dominant (Immune C2) |
| TCGA-QK-A8Z8-01 | IFN-gamma Dominant (Immune C2) |
| TCGA-CQ-5326-01 | IFN-gamma Dominant (Immune C2) |
| TCGA-DB-A4XF-01 | Immunologically Quiet (Immune C5) |
| TCGA-2Y-A9H2-01 | Inflammatory (Immune C3) |
| TCGA-C5-A7CM-01 | Wound Healing (Immune C1) |
| TCGA-4K-AAAL-01 | IFN-gamma Dominant (Immune C2) |
| TCGA-A4-A5Y1-01 | Inflammatory (Immune C3) |
| TCGA-56-8201-01 | Wound Healing (Immune C1) |
| TCGA-G9-6342-01 | Inflammatory (Immune C3) |
| TCGA-KU-A66T-01 | IFN-gamma Dominant (Immune C2) |
| TCGA-DD-AAVW-01 | Inflammatory (Immune C3) |
| TCGA-CJ-4905-01 | Inflammatory (Immune C3) |
| TCGA-EP-A2KC-01 | Lymphocyte Depleted (Immune C4) |
| TCGA-MT-A51W-01 | IFN-gamma Dominant (Immune C2) |
| TCGA-LN-A7HY-01 | Wound Healing (Immune C1) |
| TCGA-FY-A3R8-01 | Inflammatory (Immune C3) |
| TCGA-WB-A81H-01 | Inflammatory (Immune C3) |
| TCGA-G7-6790-01 | Inflammatory (Immune C3) |
| TCGA-XY-A89B-01 | IFN-gamma Dominant (Immune C2) |
| TCGA-CC-A7IL-01 | Lymphocyte Depleted (Immune C4) |
| TCGA-EW-A1OX-01 | Lymphocyte Depleted (Immune C4) |
| TCGA-EL-A3T1-01 | IFN-gamma Dominant (Immune C2) |
| TCGA-55-A48X-01 | Inflammatory (Immune C3) |
| TCGA-YZ-A984-01 | Inflammatory (Immune C3) |
| TCGA-BP-5174-01 | Inflammatory (Immune C3) |
| TCGA-KN-8427-01 | Wound Healing (Immune C1) |
| TCGA-QR-A705-01 | Inflammatory (Immune C3) |
| TCGA-20-1684-01 | IFN-gamma Dominant (Immune C2) |
| TCGA-N8-A4PI-01 | Lymphocyte Depleted (Immune C4) |
| TCGA-05-4402-01 | IFN-gamma Dominant (Immune C2) |
| TCGA-2G-AAH4-01 | Wound Healing (Immune C1) |
| TCGA-CX-7086-01 | IFN-gamma Dominant (Immune C2) |
| TCGA-ET-A25L-01 | Inflammatory (Immune C3) |
| TCGA-UZ-A9PZ-01 | Inflammatory (Immune C3) |
| TCGA-AG-3881-01 | Wound Healing (Immune C1) |
| TCGA-A8-A0A9-01 | IFN-gamma Dominant (Immune C2) |
| TCGA-DE-A2OL-01 | Inflammatory (Immune C3) |
| TCGA-G7-A4TM-01 | Inflammatory (Immune C3) |
| TCGA-BQ-7060-01 | Inflammatory (Immune C3) |
| TCGA-DD-A4NS-01 | Inflammatory (Immune C3) |
| TCGA-B0-4697-01 | Inflammatory (Immune C3) |
| TCGA-QR-A6GO-01 | Lymphocyte Depleted (Immune C4) |
| TCGA-AX-A3FS-01 | IFN-gamma Dominant (Immune C2) |
| TCGA-S9-A6WP-01 | Immunologically Quiet (Immune C5) |
| TCGA-BH-A1FB-01 | Inflammatory (Immune C3) |
| TCGA-44-8119-01 | Wound Healing (Immune C1) |
| TCGA-BP-4759-01 | TGF-beta Dominant (Immune C6) |
| TCGA-FG-A713-01 | Immunologically Quiet (Immune C5) |
| TCGA-DI-A1BY-01 | Wound Healing (Immune C1) |
| TCGA-V4-A9EZ-01 | Lymphocyte Depleted (Immune C4) |
| TCGA-VS-A9V2-01 | IFN-gamma Dominant (Immune C2) |
| TCGA-VS-A94Y-01 | Wound Healing (Immune C1) |
| TCGA-F4-6461-01 | Wound Healing (Immune C1) |
| TCGA-90-A4EE-01 | IFN-gamma Dominant (Immune C2) |
| TCGA-C5-A8ZZ-01 | IFN-gamma Dominant (Immune C2) |
| TCGA-MQ-A6BS-01 | Inflammatory (Immune C3) |
| TCGA-B5-A11G-01 | IFN-gamma Dominant (Immune C2) |
| TCGA-HT-A5RA-01 | Lymphocyte Depleted (Immune C4) |
| TCGA-E1-A7YL-01 | Lymphocyte Depleted (Immune C4) |
| TCGA-AO-A0JC-01 | IFN-gamma Dominant (Immune C2) |
| TCGA-JW-A852-01 | IFN-gamma Dominant (Immune C2) |
| TCGA-BB-4224-01 | IFN-gamma Dominant (Immune C2) |
| TCGA-CJ-6030-01 | Inflammatory (Immune C3) |
| TCGA-A3-3319-01 | Inflammatory (Immune C3) |
| TCGA-VQ-AA68-01 | IFN-gamma Dominant (Immune C2) |
| TCGA-UD-AAC5-01 | Wound Healing (Immune C1) |
| TCGA-DD-AADI-01 | IFN-gamma Dominant (Immune C2) |
| TCGA-V4-A9F0-01 | Inflammatory (Immune C3) |
| TCGA-FC-7961-01 | Inflammatory (Immune C3) |
| TCGA-CC-A7IG-01 | Wound Healing (Immune C1) |
| TCGA-MR-A8JO-01 | Inflammatory (Immune C3) |
| TCGA-GC-A3YS-01 | Wound Healing (Immune C1) |
| TCGA-OR-A5LG-01 | Lymphocyte Depleted (Immune C4) |
| TCGA-VD-A8KH-01 | Lymphocyte Depleted (Immune C4) |
| TCGA-CR-7379-01 | Wound Healing (Immune C1) |
| TCGA-CJ-4918-01 | Inflammatory (Immune C3) |
| TCGA-A8-A07J-01 | TGF-beta Dominant (Immune C6) |
| TCGA-E9-A229-01 | IFN-gamma Dominant (Immune C2) |
| TCGA-L5-A4OU-01 | IFN-gamma Dominant (Immune C2) |
| TCGA-X9-A971-01 | Lymphocyte Depleted (Immune C4) |
| TCGA-D6-6826-01 | Wound Healing (Immune C1) |
| TCGA-A2-A0SX-01 | IFN-gamma Dominant (Immune C2) |
| TCGA-CU-A0YO-01 | Wound Healing (Immune C1) |
| TCGA-KK-A8I4-01 | Inflammatory (Immune C3) |
| TCGA-UZ-A9PM-01 | Inflammatory (Immune C3) |
| TCGA-B8-5545-01 | Inflammatory (Immune C3) |
| TCGA-US-A776-01 | Wound Healing (Immune C1) |
| TCGA-BR-8484-01 | IFN-gamma Dominant (Immune C2) |
| TCGA-55-7724-01 | IFN-gamma Dominant (Immune C2) |
| TCGA-F4-6703-01 | Wound Healing (Immune C1) |
| TCGA-HU-A4G8-01 | IFN-gamma Dominant (Immune C2) |
| TCGA-BC-A10W-01 | Lymphocyte Depleted (Immune C4) |
| TCGA-DX-AB2Q-01 | Wound Healing (Immune C1) |
| TCGA-44-6776-01 | Inflammatory (Immune C3) |
| TCGA-49-4514-01 | Wound Healing (Immune C1) |
| TCGA-05-4396-01 | Inflammatory (Immune C3) |
| TCGA-AA-3864-01 | Wound Healing (Immune C1) |
| TCGA-EJ-7789-01 | Lymphocyte Depleted (Immune C4) |
| TCGA-85-A50Z-01 | Wound Healing (Immune C1) |
| TCGA-B2-3923-01 | Inflammatory (Immune C3) |
| TCGA-55-8204-01 | Wound Healing (Immune C1) |
| TCGA-BP-5183-01 | Inflammatory (Immune C3) |
| TCGA-V4-A9EA-01 | Lymphocyte Depleted (Immune C4) |
| TCGA-QS-A5YR-01 | IFN-gamma Dominant (Immune C2) |
| TCGA-L4-A4E6-01 | Inflammatory (Immune C3) |
| TCGA-L5-A8NJ-01 | IFN-gamma Dominant (Immune C2) |
| TCGA-DX-A1L0-01 | TGF-beta Dominant (Immune C6) |
| TCGA-BH-A0AW-01 | IFN-gamma Dominant (Immune C2) |
| TCGA-VS-A9UJ-01 | Wound Healing (Immune C1) |
| TCGA-2G-AAF6-01 | IFN-gamma Dominant (Immune C2) |
| TCGA-B5-A11P-01 | Wound Healing (Immune C1) |
| TCGA-XD-AAUH-01 | Inflammatory (Immune C3) |
| TCGA-AX-A3FW-01 | IFN-gamma Dominant (Immune C2) |
| TCGA-BH-A2L8-01 | IFN-gamma Dominant (Immune C2) |
| TCGA-HU-A4GC-01 | IFN-gamma Dominant (Immune C2) |
| TCGA-EB-A5SE-01 | Wound Healing (Immune C1) |
| TCGA-HT-A5R9-01 | Lymphocyte Depleted (Immune C4) |
| TCGA-AA-3492-01 | Wound Healing (Immune C1) |
| TCGA-A3-3362-01 | Inflammatory (Immune C3) |
| TCGA-CS-5396-01 | Immunologically Quiet (Immune C5) |
| TCGA-DX-AB32-01 | IFN-gamma Dominant (Immune C2) |
| TCGA-BB-7866-01 | IFN-gamma Dominant (Immune C2) |
| TCGA-HD-7832-01 | IFN-gamma Dominant (Immune C2) |
| TCGA-Y8-A895-01 | Inflammatory (Immune C3) |
| TCGA-A8-A08I-01 | IFN-gamma Dominant (Immune C2) |
| TCGA-BH-A0HP-01 | Wound Healing (Immune C1) |
| TCGA-14-0790-01 | Lymphocyte Depleted (Immune C4) |
| TCGA-BH-A0BS-01 | Lymphocyte Depleted (Immune C4) |
| TCGA-DJ-A4UL-01 | Inflammatory (Immune C3) |
| TCGA-CD-A48C-01 | Wound Healing (Immune C1) |
| TCGA-AA-3544-01 | IFN-gamma Dominant (Immune C2) |
| TCGA-TS-A7P1-01 | Wound Healing (Immune C1) |
| TCGA-75-6205-01 | IFN-gamma Dominant (Immune C2) |
| TCGA-FP-8099-01 | IFN-gamma Dominant (Immune C2) |
| TCGA-ZJ-AAXI-01 | IFN-gamma Dominant (Immune C2) |
| TCGA-B6-A0IG-01 | IFN-gamma Dominant (Immune C2) |
| TCGA-XN-A8T5-01 | Inflammatory (Immune C3) |
| TCGA-ZG-A9M4-01 | Inflammatory (Immune C3) |
| TCGA-EJ-7793-01 | Inflammatory (Immune C3) |
| TCGA-AA-3496-01 | Wound Healing (Immune C1) |
| TCGA-QR-A7IN-01 | Inflammatory (Immune C3) |
| TCGA-ZF-AA4N-01 | Wound Healing (Immune C1) |
| TCGA-XF-AAMG-01 | Wound Healing (Immune C1) |
| TCGA-ZH-A8Y1-01 | Inflammatory (Immune C3) |
| TCGA-DJ-A13T-01 | Inflammatory (Immune C3) |
| TCGA-QR-A6GT-01 | TGF-beta Dominant (Immune C6) |
| TCGA-AK-3451-01 | IFN-gamma Dominant (Immune C2) |
| TCGA-CJ-4923-01 | IFN-gamma Dominant (Immune C2) |
| TCGA-FV-A3R2-01 | Lymphocyte Depleted (Immune C4) |
| TCGA-CZ-5987-01 | Inflammatory (Immune C3) |
| TCGA-98-A538-01 | Wound Healing (Immune C1) |
| TCGA-ET-A3BO-01 | Inflammatory (Immune C3) |
| TCGA-KP-A3W0-01 | Wound Healing (Immune C1) |
| TCGA-CV-6436-01 | IFN-gamma Dominant (Immune C2) |
| TCGA-EJ-7782-01 | Inflammatory (Immune C3) |
| TCGA-HW-8322-01 | Immunologically Quiet (Immune C5) |
| TCGA-BP-4159-01 | TGF-beta Dominant (Immune C6) |
| TCGA-Q1-A6DT-01 | Wound Healing (Immune C1) |
| TCGA-85-6561-01 | Wound Healing (Immune C1) |
| TCGA-L5-A8NN-01 | IFN-gamma Dominant (Immune C2) |
| TCGA-ZF-A9RL-01 | Lymphocyte Depleted (Immune C4) |
| TCGA-25-1633-01 | IFN-gamma Dominant (Immune C2) |
| TCGA-25-2396-01 | IFN-gamma Dominant (Immune C2) |
| TCGA-BR-7715-01 | Wound Healing (Immune C1) |
| TCGA-BH-A0DO-01 | Inflammatory (Immune C3) |
| TCGA-HC-A8CY-01 | Lymphocyte Depleted (Immune C4) |
| TCGA-X6-A8C5-01 | Inflammatory (Immune C3) |
| TCGA-DY-A0XA-01 | Wound Healing (Immune C1) |
| TCGA-DM-A28E-01 | Wound Healing (Immune C1) |
| TCGA-S9-A6U9-01 | Immunologically Quiet (Immune C5) |
| TCGA-5R-AA1C-01 | Lymphocyte Depleted (Immune C4) |
| TCGA-CK-6747-01 | Wound Healing (Immune C1) |
| TCGA-WB-A819-01 | Lymphocyte Depleted (Immune C4) |
| TCGA-Y8-A8S1-01 | TGF-beta Dominant (Immune C6) |
| TCGA-G9-7523-01 | Inflammatory (Immune C3) |
| TCGA-CJ-5680-01 | Inflammatory (Immune C3) |
| TCGA-34-5234-01 | IFN-gamma Dominant (Immune C2) |
| TCGA-A6-5667-01 | Wound Healing (Immune C1) |
| TCGA-HT-7676-01 | Immunologically Quiet (Immune C5) |
| TCGA-LN-A4A4-01 | Wound Healing (Immune C1) |
| TCGA-XF-A9T0-01 | Wound Healing (Immune C1) |
| TCGA-FW-A5DX-01 | Lymphocyte Depleted (Immune C4) |
| TCGA-62-A46O-01 | IFN-gamma Dominant (Immune C2) |
| TCGA-G3-AAV1-01 | Lymphocyte Depleted (Immune C4) |
| TCGA-HT-7469-01 | Lymphocyte Depleted (Immune C4) |
| TCGA-56-7822-01 | Wound Healing (Immune C1) |
| TCGA-56-A62T-01 | IFN-gamma Dominant (Immune C2) |
| TCGA-LL-A73Y-01 | Inflammatory (Immune C3) |
| TCGA-BC-A10X-01 | Inflammatory (Immune C3) |
| TCGA-EA-A3QE-01 | IFN-gamma Dominant (Immune C2) |
| TCGA-BH-A0DK-01 | IFN-gamma Dominant (Immune C2) |
| TCGA-GC-A4ZW-01 | Wound Healing (Immune C1) |
| TCGA-K4-A4AC-01 | IFN-gamma Dominant (Immune C2) |
| TCGA-85-A4JB-01 | IFN-gamma Dominant (Immune C2) |
| TCGA-62-A472-01 | IFN-gamma Dominant (Immune C2) |
| TCGA-NJ-A55R-01 | Inflammatory (Immune C3) |
| TCGA-23-1026-01 | IFN-gamma Dominant (Immune C2) |
| TCGA-VQ-A8E3-01 | IFN-gamma Dominant (Immune C2) |
| TCGA-F5-6702-01 | Wound Healing (Immune C1) |
| TCGA-VS-A9UQ-01 | Wound Healing (Immune C1) |
| TCGA-BH-A0BG-01 | IFN-gamma Dominant (Immune C2) |
| TCGA-AJ-A23N-01 | Wound Healing (Immune C1) |
| TCGA-W5-AA34-01 | Inflammatory (Immune C3) |
| TCGA-CA-5255-01 | Wound Healing (Immune C1) |
| TCGA-93-A4JP-01 | Lymphocyte Depleted (Immune C4) |
| TCGA-CK-4947-01 | Wound Healing (Immune C1) |
| TCGA-FD-A3B3-01 | IFN-gamma Dominant (Immune C2) |
| TCGA-IE-A6BZ-01 | Inflammatory (Immune C3) |
| TCGA-FU-A3HY-01 | Wound Healing (Immune C1) |
| TCGA-2G-AAGS-01 | Wound Healing (Immune C1) |
| TCGA-V1-A8MU-01 | Inflammatory (Immune C3) |
| TCGA-G6-A8L8-01 | Inflammatory (Immune C3) |
| TCGA-KP-A3W1-01 | IFN-gamma Dominant (Immune C2) |
| TCGA-CV-A6JZ-01 | IFN-gamma Dominant (Immune C2) |
| TCGA-32-2634-01 | Lymphocyte Depleted (Immune C4) |
| TCGA-BS-A0U7-01 | Wound Healing (Immune C1) |
| TCGA-62-8395-01 | Inflammatory (Immune C3) |
| TCGA-24-2261-01 | IFN-gamma Dominant (Immune C2) |
| TCGA-AN-A0AT-01 | Wound Healing (Immune C1) |
| TCGA-13-0800-01 | IFN-gamma Dominant (Immune C2) |
| TCGA-3U-A98H-01 | TGF-beta Dominant (Immune C6) |
| TCGA-77-8136-01 | IFN-gamma Dominant (Immune C2) |
| TCGA-D1-A2G0-01 | Wound Healing (Immune C1) |
| TCGA-ET-A40P-01 | Inflammatory (Immune C3) |
| TCGA-CW-5589-01 | Inflammatory (Immune C3) |
| TCGA-VQ-A8E0-01 | Wound Healing (Immune C1) |
| TCGA-66-2756-01 | Wound Healing (Immune C1) |
| TCGA-61-1998-01 | Lymphocyte Depleted (Immune C4) |
| TCGA-23-1029-01 | IFN-gamma Dominant (Immune C2) |
| TCGA-95-7567-01 | IFN-gamma Dominant (Immune C2) |
| TCGA-E2-A14U-01 | Inflammatory (Immune C3) |
| TCGA-AD-6888-01 | Wound Healing (Immune C1) |
| TCGA-KB-A93G-01 | Wound Healing (Immune C1) |
| TCGA-F4-6809-01 | Wound Healing (Immune C1) |
| TCGA-A3-3385-01 | Inflammatory (Immune C3) |
| TCGA-VS-A9UV-01 | Wound Healing (Immune C1) |
| TCGA-50-5051-01 | Wound Healing (Immune C1) |
| TCGA-BP-5000-01 | Inflammatory (Immune C3) |
| TCGA-CN-A63U-01 | IFN-gamma Dominant (Immune C2) |
| TCGA-AA-3502-01 | Wound Healing (Immune C1) |
| TCGA-HT-7478-01 | Lymphocyte Depleted (Immune C4) |
| TCGA-5P-A9K6-01 | Inflammatory (Immune C3) |
| TCGA-VS-A8EG-01 | IFN-gamma Dominant (Immune C2) |
| TCGA-IZ-8195-01 | Inflammatory (Immune C3) |
| TCGA-43-8116-01 | Wound Healing (Immune C1) |
| TCGA-MW-A4EC-01 | Inflammatory (Immune C3) |
| TCGA-P3-A6T2-01 | IFN-gamma Dominant (Immune C2) |
| TCGA-A3-3343-01 | Inflammatory (Immune C3) |
| TCGA-L5-A4OM-01 | Wound Healing (Immune C1) |
| TCGA-WB-A80Q-01 | Inflammatory (Immune C3) |
| TCGA-BK-A13B-01 | Wound Healing (Immune C1) |
| TCGA-EX-A69L-01 | IFN-gamma Dominant (Immune C2) |
| TCGA-FJ-A871-01 | Wound Healing (Immune C1) |
| TCGA-WB-A817-01 | Inflammatory (Immune C3) |
| TCGA-55-A48Y-01 | Wound Healing (Immune C1) |
| TCGA-BJ-A45F-01 | Inflammatory (Immune C3) |
| TCGA-AA-3525-01 | Wound Healing (Immune C1) |
| TCGA-AK-3447-01 | Lymphocyte Depleted (Immune C4) |
| TCGA-Q1-A73O-01 | IFN-gamma Dominant (Immune C2) |
| TCGA-D5-6532-01 | Wound Healing (Immune C1) |
| TCGA-QR-A70J-01 | IFN-gamma Dominant (Immune C2) |
| TCGA-CC-A9FU-01 | Wound Healing (Immune C1) |
| TCGA-VS-A8QC-01 | IFN-gamma Dominant (Immune C2) |
| TCGA-86-A456-01 | IFN-gamma Dominant (Immune C2) |
| TCGA-33-AASB-01 | Wound Healing (Immune C1) |
| TCGA-EA-A5ZE-01 | IFN-gamma Dominant (Immune C2) |
| TCGA-XT-AASU-01 | Wound Healing (Immune C1) |
| TCGA-FE-A22Z-01 | Inflammatory (Immune C3) |
| TCGA-CV-7429-01 | IFN-gamma Dominant (Immune C2) |
| TCGA-RW-A8AZ-01 | Lymphocyte Depleted (Immune C4) |
| TCGA-50-5068-01 | IFN-gamma Dominant (Immune C2) |
| TCGA-AG-A00Y-01 | IFN-gamma Dominant (Immune C2) |
| TCGA-NJ-A55A-01 | Inflammatory (Immune C3) |
| TCGA-ZS-A9CF-01 | Lymphocyte Depleted (Immune C4) |
| TCGA-A4-8516-01 | Lymphocyte Depleted (Immune C4) |
| TCGA-56-7580-01 | Wound Healing (Immune C1) |
| TCGA-E8-A416-01 | Inflammatory (Immune C3) |
| TCGA-S4-A8RM-01 | Inflammatory (Immune C3) |
| TCGA-DU-A5TW-01 | Immunologically Quiet (Immune C5) |
| TCGA-DX-AB2S-01 | IFN-gamma Dominant (Immune C2) |
| TCGA-A5-A0R9-01 | Wound Healing (Immune C1) |
| TCGA-IB-7890-01 | Wound Healing (Immune C1) |
| TCGA-GD-A3OP-01 | Inflammatory (Immune C3) |
| TCGA-90-7769-01 | Wound Healing (Immune C1) |
| TCGA-CR-6467-01 | IFN-gamma Dominant (Immune C2) |
| TCGA-S9-A6TX-01 | Immunologically Quiet (Immune C5) |
| TCGA-B5-A0K8-01 | IFN-gamma Dominant (Immune C2) |
| TCGA-IG-A97H-01 | IFN-gamma Dominant (Immune C2) |
| TCGA-37-4135-01 | IFN-gamma Dominant (Immune C2) |
| TCGA-RY-A83Z-01 | Lymphocyte Depleted (Immune C4) |
| TCGA-IG-A5B8-01 | IFN-gamma Dominant (Immune C2) |
| TCGA-JX-A5QV-01 | IFN-gamma Dominant (Immune C2) |
| TCGA-AA-3930-01 | IFN-gamma Dominant (Immune C2) |
| TCGA-BR-8683-01 | IFN-gamma Dominant (Immune C2) |
| TCGA-DX-A8BQ-01 | IFN-gamma Dominant (Immune C2) |
| TCGA-EW-A1P5-01 | IFN-gamma Dominant (Immune C2) |
| TCGA-DJ-A13W-01 | Lymphocyte Depleted (Immune C4) |
| TCGA-QR-A6GS-01 | Inflammatory (Immune C3) |
| TCGA-FD-A3SL-01 | Wound Healing (Immune C1) |
| TCGA-ET-A39L-01 | Inflammatory (Immune C3) |
| TCGA-BH-A1FN-01 | IFN-gamma Dominant (Immune C2) |
| TCGA-TS-A8AF-01 | Wound Healing (Immune C1) |
| TCGA-EK-A2GZ-01 | Wound Healing (Immune C1) |
| TCGA-N5-A4RM-01 | Wound Healing (Immune C1) |
| TCGA-AZ-6600-01 | Wound Healing (Immune C1) |
| TCGA-MM-A564-01 | Inflammatory (Immune C3) |
| TCGA-E9-A3X8-01 | Wound Healing (Immune C1) |
| TCGA-J4-A6G3-01 | Inflammatory (Immune C3) |
| TCGA-51-4079-01 | Wound Healing (Immune C1) |
| TCGA-DQ-7596-01 | IFN-gamma Dominant (Immune C2) |
| TCGA-HZ-8002-01 | Inflammatory (Immune C3) |
| TCGA-3A-A9IH-01 | IFN-gamma Dominant (Immune C2) |
| TCGA-B5-A3FC-01 | IFN-gamma Dominant (Immune C2) |
| TCGA-BJ-A0ZC-01 | Inflammatory (Immune C3) |
| TCGA-98-8020-01 | Wound Healing (Immune C1) |
| TCGA-CV-5970-01 | IFN-gamma Dominant (Immune C2) |
| TCGA-DX-AB2G-01 | TGF-beta Dominant (Immune C6) |
| TCGA-A7-A4SE-01 | IFN-gamma Dominant (Immune C2) |
| TCGA-A1-A0SJ-01 | Wound Healing (Immune C1) |
| TCGA-J2-A4AG-01 | Inflammatory (Immune C3) |
| TCGA-EI-6510-01 | Wound Healing (Immune C1) |
| TCGA-G4-6588-01 | Wound Healing (Immune C1) |
| TCGA-RC-A7S9-01 | Lymphocyte Depleted (Immune C4) |
| TCGA-22-5471-01 | IFN-gamma Dominant (Immune C2) |
| TCGA-E2-A570-01 | Wound Healing (Immune C1) |
| TCGA-CZ-5466-01 | Inflammatory (Immune C3) |
| TCGA-CV-7425-01 | IFN-gamma Dominant (Immune C2) |
| TCGA-EK-A2R9-01 | IFN-gamma Dominant (Immune C2) |
| TCGA-QR-A6H4-01 | Lymphocyte Depleted (Immune C4) |
| TCGA-BH-A201-01 | Wound Healing (Immune C1) |
| TCGA-66-2744-01 | IFN-gamma Dominant (Immune C2) |
| TCGA-61-2016-01 | IFN-gamma Dominant (Immune C2) |
| TCGA-CN-4733-01 | IFN-gamma Dominant (Immune C2) |
| TCGA-IR-A3LL-01 | IFN-gamma Dominant (Immune C2) |
| TCGA-05-4405-01 | Inflammatory (Immune C3) |
| TCGA-06-0125-01 | Lymphocyte Depleted (Immune C4) |
| TCGA-V4-A9E5-01 | Lymphocyte Depleted (Immune C4) |
| TCGA-DD-AA3A-01 | Wound Healing (Immune C1) |
| TCGA-RW-A68A-01 | Lymphocyte Depleted (Immune C4) |
| TCGA-AG-A036-01 | Wound Healing (Immune C1) |
| TCGA-D1-A103-01 | IFN-gamma Dominant (Immune C2) |
| TCGA-A5-A0R6-01 | IFN-gamma Dominant (Immune C2) |
| TCGA-Y8-A8RZ-01 | Lymphocyte Depleted (Immune C4) |
| TCGA-CR-5243-01 | IFN-gamma Dominant (Immune C2) |
| TCGA-CZ-5469-01 | Wound Healing (Immune C1) |
| TCGA-B6-A0RP-01 | Inflammatory (Immune C3) |
| TCGA-D8-A27V-01 | Inflammatory (Immune C3) |
| TCGA-ZF-AA4V-01 | IFN-gamma Dominant (Immune C2) |
| TCGA-50-5944-01 | Inflammatory (Immune C3) |
| TCGA-RD-A8N1-01 | IFN-gamma Dominant (Immune C2) |
| TCGA-BR-8687-01 | Wound Healing (Immune C1) |
| TCGA-OR-A5JV-01 | Immunologically Quiet (Immune C5) |
| TCGA-ED-A7PY-01 | Inflammatory (Immune C3) |
| TCGA-EM-A2CO-01 | Inflammatory (Immune C3) |
| TCGA-BR-A4IY-01 | IFN-gamma Dominant (Immune C2) |
| TCGA-50-6673-01 | TGF-beta Dominant (Immune C6) |
| TCGA-V1-A9OQ-01 | Inflammatory (Immune C3) |
| TCGA-FG-A6IZ-01 | Immunologically Quiet (Immune C5) |
| TCGA-C5-A1M5-01 | IFN-gamma Dominant (Immune C2) |
| TCGA-DQ-7592-01 | IFN-gamma Dominant (Immune C2) |
| TCGA-CR-7372-01 | IFN-gamma Dominant (Immune C2) |
| TCGA-B5-A1N2-01 | IFN-gamma Dominant (Immune C2) |
| TCGA-YL-A9WI-01 | Lymphocyte Depleted (Immune C4) |
| TCGA-EM-A2OZ-01 | Inflammatory (Immune C3) |
| TCGA-55-7727-01 | IFN-gamma Dominant (Immune C2) |
| TCGA-QR-A6H0-01 | Inflammatory (Immune C3) |
| TCGA-CS-4941-01 | Lymphocyte Depleted (Immune C4) |
| TCGA-A1-A0SE-01 | Wound Healing (Immune C1) |
| TCGA-OU-A5PI-01 | Lymphocyte Depleted (Immune C4) |
| TCGA-A4-7996-01 | Inflammatory (Immune C3) |
| TCGA-77-8153-01 | Wound Healing (Immune C1) |
| TCGA-MS-A51U-01 | TGF-beta Dominant (Immune C6) |
| TCGA-L5-A88Y-01 | Inflammatory (Immune C3) |
| TCGA-FY-A3WA-01 | Inflammatory (Immune C3) |
| TCGA-QS-A8F1-01 | Inflammatory (Immune C3) |
| TCGA-G3-A25S-01 | Lymphocyte Depleted (Immune C4) |
| TCGA-ER-A19K-01 | Lymphocyte Depleted (Immune C4) |
| TCGA-EM-A1CS-01 | Inflammatory (Immune C3) |
| TCGA-EL-A3ZM-01 | IFN-gamma Dominant (Immune C2) |
| TCGA-DJ-A13X-01 | Inflammatory (Immune C3) |
| TCGA-DX-A1L3-01 | Inflammatory (Immune C3) |
| TCGA-2Z-A9JN-01 | Inflammatory (Immune C3) |
| TCGA-AR-A1AM-01 | Wound Healing (Immune C1) |
| TCGA-HT-8113-01 | Lymphocyte Depleted (Immune C4) |
| TCGA-13-0890-01 | IFN-gamma Dominant (Immune C2) |
| TCGA-VF-A8AE-01 | IFN-gamma Dominant (Immune C2) |
| TCGA-DH-A7UT-01 | Immunologically Quiet (Immune C5) |
| TCGA-D1-A16F-01 | Wound Healing (Immune C1) |
| TCGA-CK-6751-01 | Wound Healing (Immune C1) |
| TCGA-CV-7238-01 | IFN-gamma Dominant (Immune C2) |
| TCGA-EL-A3T7-01 | Inflammatory (Immune C3) |
| TCGA-SN-A6IS-01 | IFN-gamma Dominant (Immune C2) |
| TCGA-B0-5075-01 | Inflammatory (Immune C3) |
| TCGA-DU-5851-01 | Immunologically Quiet (Immune C5) |
| TCGA-CN-5367-01 | IFN-gamma Dominant (Immune C2) |
| TCGA-DX-A3LS-01 | TGF-beta Dominant (Immune C6) |
| TCGA-86-8279-01 | Wound Healing (Immune C1) |
| TCGA-W2-A7HB-01 | Immunologically Quiet (Immune C5) |
| TCGA-E7-A6MD-01 | Wound Healing (Immune C1) |
| TCGA-CR-5250-01 | Inflammatory (Immune C3) |
| TCGA-B0-5100-01 | Inflammatory (Immune C3) |
| TCGA-EJ-7218-01 | Inflammatory (Immune C3) |
| TCGA-XK-AAJR-01 | Inflammatory (Immune C3) |
| TCGA-55-A494-01 | Inflammatory (Immune C3) |
| TCGA-BP-4775-01 | Inflammatory (Immune C3) |
| TCGA-2G-AALF-01 | Wound Healing (Immune C1) |
| TCGA-ET-A3BT-01 | Inflammatory (Immune C3) |
| TCGA-61-2000-01 | IFN-gamma Dominant (Immune C2) |
| TCGA-A4-7288-01 | Inflammatory (Immune C3) |
| TCGA-BR-4369-01 | Wound Healing (Immune C1) |
| TCGA-B0-5400-01 | Inflammatory (Immune C3) |
| TCGA-G4-6317-01 | Wound Healing (Immune C1) |
| TCGA-25-2404-01 | IFN-gamma Dominant (Immune C2) |
| TCGA-CV-7437-01 | Wound Healing (Immune C1) |
| TCGA-55-7913-01 | Wound Healing (Immune C1) |
| TCGA-K7-A5RG-01 | IFN-gamma Dominant (Immune C2) |
| TCGA-QR-A707-01 | Lymphocyte Depleted (Immune C4) |
| TCGA-EY-A1GW-01 | Lymphocyte Depleted (Immune C4) |
| TCGA-C5-A7X8-01 | Wound Healing (Immune C1) |
| TCGA-B0-5120-01 | Inflammatory (Immune C3) |
| TCGA-2G-AAKM-01 | Wound Healing (Immune C1) |
| TCGA-SG-A6Z4-01 | Lymphocyte Depleted (Immune C4) |
| TCGA-VD-AA8R-01 | Immunologically Quiet (Immune C5) |
| TCGA-3B-A9HZ-01 | Inflammatory (Immune C3) |
| TCGA-CJ-4873-01 | Inflammatory (Immune C3) |
| TCGA-SX-A7SL-01 | Lymphocyte Depleted (Immune C4) |
| TCGA-BH-A1EX-01 | TGF-beta Dominant (Immune C6) |
| TCGA-HW-8321-01 | Immunologically Quiet (Immune C5) |
| TCGA-G4-6309-01 | Wound Healing (Immune C1) |
| TCGA-KL-8340-01 | Inflammatory (Immune C3) |
| TCGA-EZ-7264-01 | Immunologically Quiet (Immune C5) |
| TCGA-E8-A433-01 | Inflammatory (Immune C3) |
| TCGA-EJ-7791-01 | Inflammatory (Immune C3) |
| TCGA-IN-A6RR-01 | IFN-gamma Dominant (Immune C2) |
| TCGA-55-8615-01 | Inflammatory (Immune C3) |
| TCGA-HZ-A4BK-01 | TGF-beta Dominant (Immune C6) |
| TCGA-EL-A3GQ-01 | Inflammatory (Immune C3) |
| TCGA-BK-A26L-01 | Wound Healing (Immune C1) |
| TCGA-VF-A8AA-01 | IFN-gamma Dominant (Immune C2) |
| TCGA-G2-A2EO-01 | Wound Healing (Immune C1) |
| TCGA-D6-A6EO-01 | IFN-gamma Dominant (Immune C2) |
| TCGA-RC-A7SF-01 | Inflammatory (Immune C3) |
| TCGA-EB-A24C-01 | Lymphocyte Depleted (Immune C4) |
| TCGA-F1-A448-01 | Wound Healing (Immune C1) |
| TCGA-A7-A0CJ-01 | IFN-gamma Dominant (Immune C2) |
| TCGA-CS-6666-01 | Lymphocyte Depleted (Immune C4) |
| TCGA-BP-4992-01 | Inflammatory (Immune C3) |
| TCGA-CM-5868-01 | Wound Healing (Immune C1) |
| TCGA-WC-A885-01 | Lymphocyte Depleted (Immune C4) |
| TCGA-BR-A4IV-01 | Inflammatory (Immune C3) |
| TCGA-HD-A4C1-01 | IFN-gamma Dominant (Immune C2) |
| TCGA-P4-AAVO-01 | Lymphocyte Depleted (Immune C4) |
| TCGA-NC-A5HK-01 | IFN-gamma Dominant (Immune C2) |
| TCGA-VQ-A91Z-01 | Wound Healing (Immune C1) |
| TCGA-DX-A8BR-01 | Lymphocyte Depleted (Immune C4) |
| TCGA-W5-AA2X-01 | Wound Healing (Immune C1) |
| TCGA-DH-A66D-01 | Immunologically Quiet (Immune C5) |
| TCGA-IR-A3LH-01 | IFN-gamma Dominant (Immune C2) |
| TCGA-KO-8410-01 | Inflammatory (Immune C3) |
| TCGA-D1-A16R-01 | Wound Healing (Immune C1) |
| TCGA-LN-A4A9-01 | IFN-gamma Dominant (Immune C2) |
| TCGA-LL-A5YO-01 | IFN-gamma Dominant (Immune C2) |
| TCGA-86-A4JF-01 | IFN-gamma Dominant (Immune C2) |
| TCGA-DC-6156-01 | TGF-beta Dominant (Immune C6) |
| TCGA-QU-A6IP-01 | Inflammatory (Immune C3) |
| TCGA-FI-A3PV-01 | Wound Healing (Immune C1) |
| TCGA-V1-A9O9-01 | Inflammatory (Immune C3) |
| TCGA-EO-A2CG-01 | Wound Healing (Immune C1) |
| TCGA-AN-A0FW-01 | Wound Healing (Immune C1) |
| TCGA-VQ-A8PH-01 | Wound Healing (Immune C1) |
| TCGA-AC-A6IX-01 | Wound Healing (Immune C1) |
| TCGA-A5-A0GU-01 | IFN-gamma Dominant (Immune C2) |
| TCGA-EB-A551-01 | IFN-gamma Dominant (Immune C2) |
| TCGA-P5-A77W-01 | Immunologically Quiet (Immune C5) |
| TCGA-HW-7491-01 | Immunologically Quiet (Immune C5) |
| TCGA-EY-A1GS-01 | Lymphocyte Depleted (Immune C4) |
| TCGA-AA-A010-01 | Wound Healing (Immune C1) |
| TCGA-A3-3320-01 | Inflammatory (Immune C3) |
| TCGA-FT-A3EE-01 | Wound Healing (Immune C1) |
| TCGA-AA-3837-01 | Wound Healing (Immune C1) |
| TCGA-L9-A443-01 | Inflammatory (Immune C3) |
| TCGA-DJ-A2PN-01 | Inflammatory (Immune C3) |
| TCGA-G3-A7M6-01 | Inflammatory (Immune C3) |
| TCGA-E9-A1RG-01 | Wound Healing (Immune C1) |
| TCGA-66-2767-01 | IFN-gamma Dominant (Immune C2) |
| TCGA-MR-A520-01 | Lymphocyte Depleted (Immune C4) |
| TCGA-FD-A3B7-01 | IFN-gamma Dominant (Immune C2) |
| TCGA-E1-5307-01 | Immunologically Quiet (Immune C5) |
| TCGA-78-8660-01 | IFN-gamma Dominant (Immune C2) |
| TCGA-C5-A1M9-01 | IFN-gamma Dominant (Immune C2) |
| TCGA-KL-8325-01 | Immunologically Quiet (Immune C5) |
| TCGA-HW-7495-01 | Immunologically Quiet (Immune C5) |
| TCGA-K4-A3WU-01 | Inflammatory (Immune C3) |
| TCGA-AG-4021-01 | Wound Healing (Immune C1) |
| TCGA-E2-A156-01 | Inflammatory (Immune C3) |
| TCGA-CS-6669-01 | Immunologically Quiet (Immune C5) |
| TCGA-EL-A3GW-01 | Inflammatory (Immune C3) |
| TCGA-EM-A2CL-01 | Inflammatory (Immune C3) |
| TCGA-CF-A47V-01 | Lymphocyte Depleted (Immune C4) |
| TCGA-AR-A24R-01 | IFN-gamma Dominant (Immune C2) |
| TCGA-BS-A0TE-01 | IFN-gamma Dominant (Immune C2) |
| TCGA-HT-7481-01 | Immunologically Quiet (Immune C5) |
| TCGA-BG-A0MU-01 | Inflammatory (Immune C3) |
| TCGA-NC-A5HO-01 | Wound Healing (Immune C1) |
| TCGA-EY-A1GD-01 | Wound Healing (Immune C1) |
| TCGA-BF-A1PZ-01 | IFN-gamma Dominant (Immune C2) |
| TCGA-XQ-A8TA-01 | Lymphocyte Depleted (Immune C4) |
| TCGA-78-7148-01 | Wound Healing (Immune C1) |
| TCGA-DU-8163-01 | Immunologically Quiet (Immune C5) |
| TCGA-D8-A1JP-01 | Wound Healing (Immune C1) |
| TCGA-T2-A6WZ-01 | IFN-gamma Dominant (Immune C2) |
| TCGA-AP-A0LN-01 | Wound Healing (Immune C1) |
| TCGA-AX-A06L-01 | Wound Healing (Immune C1) |
| TCGA-AN-A0FS-01 | Wound Healing (Immune C1) |
| TCGA-77-8154-01 | Wound Healing (Immune C1) |
| TCGA-2G-AAFM-01 | IFN-gamma Dominant (Immune C2) |
| TCGA-DJ-A3V5-01 | Inflammatory (Immune C3) |
| TCGA-AC-A3TM-01 | Wound Healing (Immune C1) |
| TCGA-DJ-A2Q3-01 | Inflammatory (Immune C3) |
| TCGA-D6-8568-01 | IFN-gamma Dominant (Immune C2) |
| TCGA-CV-7446-01 | IFN-gamma Dominant (Immune C2) |
| TCGA-A5-A0GQ-01 | IFN-gamma Dominant (Immune C2) |
| TCGA-DJ-A2PY-01 | Inflammatory (Immune C3) |
| TCGA-DK-A3X1-01 | Wound Healing (Immune C1) |
| TCGA-BG-A0MQ-01 | Wound Healing (Immune C1) |
| TCGA-B9-A5W7-01 | Inflammatory (Immune C3) |
| TCGA-C8-A12K-01 | IFN-gamma Dominant (Immune C2) |
| TCGA-AR-A2LE-01 | Inflammatory (Immune C3) |
| TCGA-AD-6964-01 | IFN-gamma Dominant (Immune C2) |
| TCGA-37-A5EL-01 | Wound Healing (Immune C1) |
| TCGA-26-1442-01 | Immunologically Quiet (Immune C5) |
| TCGA-B0-4688-01 | Lymphocyte Depleted (Immune C4) |
| TCGA-AA-3848-01 | Wound Healing (Immune C1) |
| TCGA-BH-A0HK-01 | Wound Healing (Immune C1) |
| TCGA-CJ-4899-01 | Inflammatory (Immune C3) |
| TCGA-G4-6295-01 | Wound Healing (Immune C1) |
| TCGA-RD-A8NB-01 | IFN-gamma Dominant (Immune C2) |
| TCGA-B2-4101-01 | Inflammatory (Immune C3) |
| TCGA-BQ-7048-01 | Inflammatory (Immune C3) |
| TCGA-DG-A2KJ-01 | IFN-gamma Dominant (Immune C2) |
| TCGA-55-A490-01 | Wound Healing (Immune C1) |
| TCGA-4Z-AA81-01 | IFN-gamma Dominant (Immune C2) |
| TCGA-D1-A16E-01 | IFN-gamma Dominant (Immune C2) |
| TCGA-CF-A3MG-01 | Wound Healing (Immune C1) |
| TCGA-QR-A708-01 | Lymphocyte Depleted (Immune C4) |
| TCGA-CS-4942-01 | Lymphocyte Depleted (Immune C4) |
| TCGA-BR-A4PE-01 | IFN-gamma Dominant (Immune C2) |
| TCGA-NH-A50T-01 | Wound Healing (Immune C1) |
| TCGA-FE-A237-01 | Inflammatory (Immune C3) |
| TCGA-2G-AAFI-01 | IFN-gamma Dominant (Immune C2) |
| TCGA-ZS-A9CE-01 | Lymphocyte Depleted (Immune C4) |
| TCGA-AR-A250-01 | Wound Healing (Immune C1) |
| TCGA-HC-7230-01 | Inflammatory (Immune C3) |
| TCGA-S5-AA26-01 | Wound Healing (Immune C1) |
| TCGA-HT-8110-01 | Lymphocyte Depleted (Immune C4) |
| TCGA-D8-A1JG-01 | IFN-gamma Dominant (Immune C2) |
| TCGA-KL-8337-01 | Immunologically Quiet (Immune C5) |
| TCGA-ZF-A9R4-01 | Lymphocyte Depleted (Immune C4) |
| TCGA-E2-A152-01 | Wound Healing (Immune C1) |
| TCGA-GN-A26C-01 | IFN-gamma Dominant (Immune C2) |
| TCGA-AR-A24V-01 | IFN-gamma Dominant (Immune C2) |
| TCGA-85-8070-01 | Wound Healing (Immune C1) |
| TCGA-BS-A0TA-01 | IFN-gamma Dominant (Immune C2) |
| TCGA-A2-A0CR-01 | TGF-beta Dominant (Immune C6) |
| TCGA-AK-3429-01 | Inflammatory (Immune C3) |
| TCGA-PC-A5DL-01 | TGF-beta Dominant (Immune C6) |
| TCGA-DX-A6BB-01 | Lymphocyte Depleted (Immune C4) |
| TCGA-S9-A6U1-01 | Immunologically Quiet (Immune C5) |
| TCGA-YC-A9TC-01 | Wound Healing (Immune C1) |
| TCGA-BS-A0V4-01 | Inflammatory (Immune C3) |
| TCGA-AN-A0FX-01 | Wound Healing (Immune C1) |
| TCGA-Z6-A9VB-01 | IFN-gamma Dominant (Immune C2) |
| TCGA-Q1-A5R3-01 | IFN-gamma Dominant (Immune C2) |
| TCGA-AC-A62V-01 | Wound Healing (Immune C1) |
| TCGA-CN-6988-01 | Wound Healing (Immune C1) |
| TCGA-ET-A39O-01 | Inflammatory (Immune C3) |
| TCGA-AP-A0LJ-01 | Inflammatory (Immune C3) |
| TCGA-UB-AA0U-01 | Inflammatory (Immune C3) |
| TCGA-A6-4107-01 | Wound Healing (Immune C1) |
| TCGA-FK-A3S3-01 | Inflammatory (Immune C3) |
| TCGA-FB-AAQ1-01 | TGF-beta Dominant (Immune C6) |
| TCGA-AK-3455-01 | IFN-gamma Dominant (Immune C2) |
| TCGA-HC-7211-01 | Inflammatory (Immune C3) |
| TCGA-2Z-A9J3-01 | Inflammatory (Immune C3) |
| TCGA-B2-3924-01 | Inflammatory (Immune C3) |
| TCGA-GM-A3XN-01 | Wound Healing (Immune C1) |
| TCGA-C8-A275-01 | IFN-gamma Dominant (Immune C2) |
| TCGA-UD-AAC7-01 | Wound Healing (Immune C1) |
| TCGA-4L-AA1F-01 | Wound Healing (Immune C1) |
| TCGA-AK-3433-01 | Inflammatory (Immune C3) |
| TCGA-DD-AAD8-01 | Lymphocyte Depleted (Immune C4) |
| TCGA-AO-A129-01 | IFN-gamma Dominant (Immune C2) |
| TCGA-P3-A6T3-01 | IFN-gamma Dominant (Immune C2) |
| TCGA-R5-A7ZR-01 | IFN-gamma Dominant (Immune C2) |
| TCGA-CV-A468-01 | IFN-gamma Dominant (Immune C2) |
| TCGA-EJ-5516-01 | Inflammatory (Immune C3) |
| TCGA-DX-A8BV-01 | Lymphocyte Depleted (Immune C4) |
| TCGA-AO-A12E-01 | Lymphocyte Depleted (Immune C4) |
| TCGA-VQ-A927-01 | Wound Healing (Immune C1) |
| TCGA-YF-AA3L-01 | Wound Healing (Immune C1) |
| TCGA-BP-4354-01 | Lymphocyte Depleted (Immune C4) |
| TCGA-BR-8059-01 | Wound Healing (Immune C1) |
| TCGA-EL-A3MZ-01 | Inflammatory (Immune C3) |
| TCGA-BS-A0V8-01 | Wound Healing (Immune C1) |
| TCGA-BH-A1F8-01 | IFN-gamma Dominant (Immune C2) |
| TCGA-G3-AAV3-01 | Inflammatory (Immune C3) |
| TCGA-QK-A6V9-01 | IFN-gamma Dominant (Immune C2) |
| TCGA-A3-3306-01 | Inflammatory (Immune C3) |
| TCGA-CC-A8HV-01 | Lymphocyte Depleted (Immune C4) |
| TCGA-D8-A1JK-01 | IFN-gamma Dominant (Immune C2) |
| TCGA-HC-A8D1-01 | Inflammatory (Immune C3) |
| TCGA-ZF-A9R0-01 | Wound Healing (Immune C1) |
| TCGA-RY-A840-01 | Immunologically Quiet (Immune C5) |
| TCGA-A8-A08X-01 | Wound Healing (Immune C1) |
| TCGA-EI-6882-01 | Wound Healing (Immune C1) |
| TCGA-BP-4975-01 | Inflammatory (Immune C3) |
| TCGA-AZ-4315-01 | IFN-gamma Dominant (Immune C2) |
| TCGA-DD-AADO-01 | IFN-gamma Dominant (Immune C2) |
| TCGA-3M-AB46-01 | IFN-gamma Dominant (Immune C2) |
| TCGA-DX-A48R-01 | Lymphocyte Depleted (Immune C4) |
| TCGA-VM-A8C9-01 | Lymphocyte Depleted (Immune C4) |
| TCGA-EB-A85I-01 | IFN-gamma Dominant (Immune C2) |
| TCGA-D6-A6EK-01 | IFN-gamma Dominant (Immune C2) |
| TCGA-CJ-4869-01 | Inflammatory (Immune C3) |
| TCGA-AA-3506-01 | Wound Healing (Immune C1) |
| TCGA-3A-A9I9-01 | Inflammatory (Immune C3) |
| TCGA-A4-8098-01 | Lymphocyte Depleted (Immune C4) |
| TCGA-BR-4255-01 | Wound Healing (Immune C1) |
| TCGA-F2-6879-01 | IFN-gamma Dominant (Immune C2) |
| TCGA-IN-A7NT-01 | IFN-gamma Dominant (Immune C2) |
| TCGA-29-1784-01 | IFN-gamma Dominant (Immune C2) |
| TCGA-SR-A6MU-01 | Lymphocyte Depleted (Immune C4) |
| TCGA-EB-A4OZ-01 | IFN-gamma Dominant (Immune C2) |
| TCGA-J8-A3YD-01 | Inflammatory (Immune C3) |
| TCGA-06-0743-01 | Lymphocyte Depleted (Immune C4) |
| TCGA-D1-A16J-01 | IFN-gamma Dominant (Immune C2) |
| TCGA-E9-A1R6-01 | Wound Healing (Immune C1) |
| TCGA-R5-A7ZI-01 | IFN-gamma Dominant (Immune C2) |
| TCGA-AJ-A3NC-01 | Wound Healing (Immune C1) |
| TCGA-QR-A70K-01 | Lymphocyte Depleted (Immune C4) |
| TCGA-G3-A25X-01 | IFN-gamma Dominant (Immune C2) |
| TCGA-AP-A1DV-01 | Wound Healing (Immune C1) |
| TCGA-BP-4983-01 | IFN-gamma Dominant (Immune C2) |
| TCGA-B4-5378-01 | Lymphocyte Depleted (Immune C4) |
| TCGA-BR-A4J6-01 | Wound Healing (Immune C1) |
| TCGA-DQ-7591-01 | IFN-gamma Dominant (Immune C2) |
| TCGA-A8-A09A-01 | Inflammatory (Immune C3) |
| TCGA-G3-A3CK-01 | Lymphocyte Depleted (Immune C4) |
| TCGA-BR-6801-01 | Wound Healing (Immune C1) |
| TCGA-F9-A4JJ-01 | Inflammatory (Immune C3) |
| TCGA-IQ-A61I-01 | IFN-gamma Dominant (Immune C2) |
| TCGA-5P-A9JZ-01 | Lymphocyte Depleted (Immune C4) |
| TCGA-HD-A6I0-01 | IFN-gamma Dominant (Immune C2) |
| TCGA-WC-A87W-01 | Inflammatory (Immune C3) |
| TCGA-EM-A3AR-01 | Inflammatory (Immune C3) |
| TCGA-A4-A5Y0-01 | Lymphocyte Depleted (Immune C4) |
| TCGA-57-1582-01 | Wound Healing (Immune C1) |
| TCGA-HB-A3L4-01 | Wound Healing (Immune C1) |
| TCGA-3A-A9IB-01 | Wound Healing (Immune C1) |
| TCGA-G7-6792-01 | Inflammatory (Immune C3) |
| TCGA-BD-A2L6-01 | Lymphocyte Depleted (Immune C4) |
| TCGA-B2-5639-01 | Inflammatory (Immune C3) |
| TCGA-GK-A6C7-01 | Inflammatory (Immune C3) |
| TCGA-EL-A4K7-01 | Inflammatory (Immune C3) |
| TCGA-ZG-A9LY-01 | Inflammatory (Immune C3) |
| TCGA-DJ-A1QI-01 | Inflammatory (Immune C3) |
| TCGA-B5-A1MW-01 | IFN-gamma Dominant (Immune C2) |
| TCGA-DJ-A2PQ-01 | Inflammatory (Immune C3) |
| TCGA-OR-A5KO-01 | Lymphocyte Depleted (Immune C4) |
| TCGA-A8-A08O-01 | Wound Healing (Immune C1) |
| TCGA-EM-A22M-01 | Inflammatory (Immune C3) |
| TCGA-AA-A01C-01 | Wound Healing (Immune C1) |
| TCGA-V4-A9ES-01 | Lymphocyte Depleted (Immune C4) |
| TCGA-CV-7422-01 | IFN-gamma Dominant (Immune C2) |
| TCGA-HT-7482-01 | Immunologically Quiet (Immune C5) |
| TCGA-CV-6962-01 | Wound Healing (Immune C1) |
| TCGA-EA-A50E-01 | IFN-gamma Dominant (Immune C2) |
| TCGA-21-1082-01 | Wound Healing (Immune C1) |
| TCGA-A2-A4RY-01 | Inflammatory (Immune C3) |
| TCGA-QH-A6CW-01 | Immunologically Quiet (Immune C5) |
| TCGA-UF-A7JT-01 | IFN-gamma Dominant (Immune C2) |
| TCGA-HC-7737-01 | Inflammatory (Immune C3) |
| TCGA-AL-3466-01 | Inflammatory (Immune C3) |
| TCGA-R5-A805-01 | IFN-gamma Dominant (Immune C2) |
| TCGA-A2-A0SY-01 | Inflammatory (Immune C3) |
| TCGA-D1-A0ZQ-01 | Wound Healing (Immune C1) |
| TCGA-AC-A8OP-01 | Inflammatory (Immune C3) |
| TCGA-98-A53J-01 | Wound Healing (Immune C1) |
| TCGA-VP-A87B-01 | Inflammatory (Immune C3) |
| TCGA-DU-A7T8-01 | Lymphocyte Depleted (Immune C4) |
| TCGA-AO-A126-01 | Wound Healing (Immune C1) |
| TCGA-EM-A4FF-01 | Inflammatory (Immune C3) |
| TCGA-CJ-4878-01 | Inflammatory (Immune C3) |
| TCGA-CH-5792-01 | Inflammatory (Immune C3) |
| TCGA-DD-A11D-01 | Lymphocyte Depleted (Immune C4) |
| TCGA-AO-A0J6-01 | IFN-gamma Dominant (Immune C2) |
| TCGA-DU-5870-01 | Immunologically Quiet (Immune C5) |
| TCGA-24-2033-01 | Lymphocyte Depleted (Immune C4) |
| TCGA-D5-5539-01 | Wound Healing (Immune C1) |
| TCGA-DD-AACK-01 | Lymphocyte Depleted (Immune C4) |
| TCGA-D8-A27F-01 | Wound Healing (Immune C1) |
| TCGA-AP-A1DR-01 | Wound Healing (Immune C1) |
| TCGA-56-7579-01 | Lymphocyte Depleted (Immune C4) |
| TCGA-EA-A556-01 | IFN-gamma Dominant (Immune C2) |
| TCGA-MJ-A68J-01 | Inflammatory (Immune C3) |
| TCGA-DD-A118-01 | Inflammatory (Immune C3) |
| TCGA-VS-A9V3-01 | IFN-gamma Dominant (Immune C2) |
| TCGA-44-6774-01 | Wound Healing (Immune C1) |
| TCGA-KD-A5QS-01 | Lymphocyte Depleted (Immune C4) |
| TCGA-BQ-7061-01 | Inflammatory (Immune C3) |
| TCGA-A5-A3LP-01 | Wound Healing (Immune C1) |
| TCGA-25-1627-01 | Wound Healing (Immune C1) |
| TCGA-63-A5MJ-01 | IFN-gamma Dominant (Immune C2) |
| TCGA-EW-A2FV-01 | IFN-gamma Dominant (Immune C2) |
| TCGA-V4-A9E8-01 | Inflammatory (Immune C3) |
| TCGA-TP-A8TV-01 | Inflammatory (Immune C3) |
| TCGA-AY-6386-01 | Wound Healing (Immune C1) |
| TCGA-D7-A74A-01 | Wound Healing (Immune C1) |
| TCGA-TM-A84S-01 | Immunologically Quiet (Immune C5) |
| TCGA-B0-4701-01 | Inflammatory (Immune C3) |
| TCGA-33-4538-01 | Wound Healing (Immune C1) |
| TCGA-26-5136-01 | Lymphocyte Depleted (Immune C4) |
| TCGA-GU-A42P-01 | Wound Healing (Immune C1) |
| TCGA-B0-4698-01 | IFN-gamma Dominant (Immune C2) |
| TCGA-CS-4943-01 | Lymphocyte Depleted (Immune C4) |
| TCGA-G7-6796-01 | Inflammatory (Immune C3) |
| TCGA-AA-3855-01 | Wound Healing (Immune C1) |
| TCGA-E9-A54X-01 | Lymphocyte Depleted (Immune C4) |
| TCGA-AX-A2HJ-01 | Wound Healing (Immune C1) |
| TCGA-AO-A0JB-01 | Wound Healing (Immune C1) |
| TCGA-2F-A9KP-01 | Wound Healing (Immune C1) |
| TCGA-A2-A1G1-01 | IFN-gamma Dominant (Immune C2) |
| TCGA-AC-A3W5-01 | Wound Healing (Immune C1) |
| TCGA-A6-5661-01 | Wound Healing (Immune C1) |
| TCGA-DJ-A1QF-01 | Inflammatory (Immune C3) |
| TCGA-N8-A4PO-01 | Wound Healing (Immune C1) |
| TCGA-KL-8338-01 | Inflammatory (Immune C3) |
| TCGA-LN-A4A2-01 | Wound Healing (Immune C1) |
| TCGA-J4-A83N-01 | Inflammatory (Immune C3) |
| TCGA-WB-A80K-01 | Inflammatory (Immune C3) |
| TCGA-CA-5797-01 | Wound Healing (Immune C1) |
| TCGA-B5-A11M-01 | Inflammatory (Immune C3) |
| TCGA-A7-A13G-01 | Inflammatory (Immune C3) |
| TCGA-AA-3514-01 | Wound Healing (Immune C1) |
| TCGA-V4-A9EW-01 | Inflammatory (Immune C3) |
| TCGA-2G-AALY-01 | Wound Healing (Immune C1) |
| TCGA-14-1034-01 | Lymphocyte Depleted (Immune C4) |
| TCGA-E2-A1IH-01 | Wound Healing (Immune C1) |
| TCGA-BR-A4IU-01 | Inflammatory (Immune C3) |
| TCGA-VS-A9UH-01 | IFN-gamma Dominant (Immune C2) |
| TCGA-B0-4691-01 | IFN-gamma Dominant (Immune C2) |
| TCGA-CN-A6V3-01 | IFN-gamma Dominant (Immune C2) |
| TCGA-G3-AAV7-01 | Lymphocyte Depleted (Immune C4) |
| TCGA-CU-A3QU-01 | Lymphocyte Depleted (Immune C4) |
| TCGA-D1-A17U-01 | Wound Healing (Immune C1) |
| TCGA-BG-A0VW-01 | Wound Healing (Immune C1) |
| TCGA-AC-A3EH-01 | Wound Healing (Immune C1) |
| TCGA-12-3652-01 | Lymphocyte Depleted (Immune C4) |
| TCGA-A3-A6NN-01 | Inflammatory (Immune C3) |
| TCGA-D8-A1Y1-01 | Lymphocyte Depleted (Immune C4) |
| TCGA-UD-AAC1-01 | Wound Healing (Immune C1) |
| TCGA-BR-8677-01 | IFN-gamma Dominant (Immune C2) |
| TCGA-AO-A03V-01 | Wound Healing (Immune C1) |
| TCGA-RY-A845-01 | Immunologically Quiet (Immune C5) |
| TCGA-CJ-5689-01 | Inflammatory (Immune C3) |
| TCGA-DK-AA6Q-01 | IFN-gamma Dominant (Immune C2) |
| TCGA-DD-A4NI-01 | Inflammatory (Immune C3) |
| TCGA-EY-A1GP-01 | IFN-gamma Dominant (Immune C2) |
| TCGA-EJ-5515-01 | Inflammatory (Immune C3) |
| TCGA-76-4927-01 | Lymphocyte Depleted (Immune C4) |
| TCGA-FG-8182-01 | Immunologically Quiet (Immune C5) |
| TCGA-UF-A7JC-01 | IFN-gamma Dominant (Immune C2) |
| TCGA-CN-5373-01 | IFN-gamma Dominant (Immune C2) |
| TCGA-E3-A3DZ-01 | Inflammatory (Immune C3) |
| TCGA-26-5132-01 | Lymphocyte Depleted (Immune C4) |
| TCGA-A3-3363-01 | Lymphocyte Depleted (Immune C4) |
| TCGA-BH-A0DD-01 | IFN-gamma Dominant (Immune C2) |
| TCGA-X6-A8C4-01 | Lymphocyte Depleted (Immune C4) |
| TCGA-VS-A9UD-01 | IFN-gamma Dominant (Immune C2) |
| TCGA-JX-A3PZ-01 | Wound Healing (Immune C1) |
| TCGA-BP-4325-01 | Inflammatory (Immune C3) |
| TCGA-DD-AADL-01 | Lymphocyte Depleted (Immune C4) |
| TCGA-BT-A20P-01 | Wound Healing (Immune C1) |
| TCGA-FB-AAPZ-01 | Inflammatory (Immune C3) |
| TCGA-98-A53A-01 | Wound Healing (Immune C1) |
| TCGA-EU-5905-01 | Inflammatory (Immune C3) |
| TCGA-CZ-5985-01 | Inflammatory (Immune C3) |
| TCGA-N9-A4Q4-01 | Wound Healing (Immune C1) |
| TCGA-WR-A838-01 | IFN-gamma Dominant (Immune C2) |
| TCGA-B5-A11I-01 | IFN-gamma Dominant (Immune C2) |
| TCGA-V1-A8WV-01 | Inflammatory (Immune C3) |
| TCGA-D7-6525-01 | IFN-gamma Dominant (Immune C2) |
| TCGA-91-8497-01 | Inflammatory (Immune C3) |
| TCGA-A5-A7WJ-01 | IFN-gamma Dominant (Immune C2) |
| TCGA-ZF-A9RN-01 | IFN-gamma Dominant (Immune C2) |
| TCGA-NC-A5HL-01 | IFN-gamma Dominant (Immune C2) |
| TCGA-L3-A524-01 | Wound Healing (Immune C1) |
| TCGA-C5-A1MP-01 | IFN-gamma Dominant (Immune C2) |
| TCGA-BR-A4J1-01 | Wound Healing (Immune C1) |
| TCGA-CC-A5UD-01 | Lymphocyte Depleted (Immune C4) |
| TCGA-IN-A6RO-01 | IFN-gamma Dominant (Immune C2) |
| TCGA-B7-A5TK-01 | IFN-gamma Dominant (Immune C2) |
| TCGA-E6-A2P8-01 | Wound Healing (Immune C1) |
| TCGA-AJ-A23O-01 | IFN-gamma Dominant (Immune C2) |
| TCGA-X6-A7WD-01 | Lymphocyte Depleted (Immune C4) |
| TCGA-56-8307-01 | IFN-gamma Dominant (Immune C2) |
| TCGA-A2-A0EN-01 | Inflammatory (Immune C3) |
| TCGA-GV-A3QI-01 | Lymphocyte Depleted (Immune C4) |
| TCGA-W5-AA2H-01 | IFN-gamma Dominant (Immune C2) |
| TCGA-DX-A6YX-01 | Lymphocyte Depleted (Immune C4) |
| TCGA-3X-AAVB-01 | Wound Healing (Immune C1) |
| TCGA-FB-AAPU-01 | Wound Healing (Immune C1) |
| TCGA-VQ-A8PO-01 | IFN-gamma Dominant (Immune C2) |
| TCGA-DD-AAE6-01 | Lymphocyte Depleted (Immune C4) |
| TCGA-J1-A4AH-01 | Wound Healing (Immune C1) |
| TCGA-GU-A42R-01 | Wound Healing (Immune C1) |
| TCGA-B0-5081-01 | Inflammatory (Immune C3) |
| TCGA-33-AASL-01 | IFN-gamma Dominant (Immune C2) |
| TCGA-CW-5587-01 | Inflammatory (Immune C3) |
| TCGA-CG-4301-01 | IFN-gamma Dominant (Immune C2) |
| TCGA-BA-5149-01 | IFN-gamma Dominant (Immune C2) |
| TCGA-FG-6688-01 | Lymphocyte Depleted (Immune C4) |
| TCGA-H9-7775-01 | Inflammatory (Immune C3) |
| TCGA-E9-A1R2-01 | IFN-gamma Dominant (Immune C2) |
| TCGA-62-A470-01 | Lymphocyte Depleted (Immune C4) |
| TCGA-A8-A08L-01 | Wound Healing (Immune C1) |
| TCGA-A7-A3RF-01 | Lymphocyte Depleted (Immune C4) |
| TCGA-53-A4EZ-01 | IFN-gamma Dominant (Immune C2) |
| TCGA-78-7162-01 | Inflammatory (Immune C3) |
| TCGA-IG-A51D-01 | IFN-gamma Dominant (Immune C2) |
| TCGA-FD-A5BU-01 | IFN-gamma Dominant (Immune C2) |
| TCGA-68-8251-01 | Wound Healing (Immune C1) |
| TCGA-BG-A2AD-01 | Wound Healing (Immune C1) |
| TCGA-B0-4847-01 | Inflammatory (Immune C3) |
| TCGA-RS-A6TO-01 | IFN-gamma Dominant (Immune C2) |
| TCGA-MQ-A4LC-01 | IFN-gamma Dominant (Immune C2) |
| TCGA-F4-6808-01 | Wound Healing (Immune C1) |
| TCGA-76-4932-01 | Lymphocyte Depleted (Immune C4) |
| TCGA-A2-A0D4-01 | Wound Healing (Immune C1) |
| TCGA-DI-A1NN-01 | IFN-gamma Dominant (Immune C2) |
| TCGA-HT-7689-01 | Immunologically Quiet (Immune C5) |
| TCGA-EW-A1IW-01 | Wound Healing (Immune C1) |
| TCGA-VQ-A94P-01 | Inflammatory (Immune C3) |
| TCGA-CV-6003-01 | IFN-gamma Dominant (Immune C2) |
| TCGA-AG-3909-01 | Wound Healing (Immune C1) |
| TCGA-21-1081-01 | Wound Healing (Immune C1) |
| TCGA-AO-A03M-01 | IFN-gamma Dominant (Immune C2) |
| TCGA-D7-6521-01 | Wound Healing (Immune C1) |
| TCGA-58-8391-01 | Wound Healing (Immune C1) |
| TCGA-HF-A5NB-01 | IFN-gamma Dominant (Immune C2) |
| TCGA-B6-A0IQ-01 | Wound Healing (Immune C1) |
| TCGA-62-8398-01 | Wound Healing (Immune C1) |
| TCGA-BH-A1FG-01 | IFN-gamma Dominant (Immune C2) |
| TCGA-AR-A0TS-01 | IFN-gamma Dominant (Immune C2) |
| TCGA-66-2754-01 | Wound Healing (Immune C1) |
| TCGA-CJ-5686-01 | Inflammatory (Immune C3) |
| TCGA-A2-A0CM-01 | IFN-gamma Dominant (Immune C2) |
| TCGA-FD-A3NA-01 | IFN-gamma Dominant (Immune C2) |
| TCGA-85-A4QQ-01 | Wound Healing (Immune C1) |
| TCGA-E2-A15I-01 | Inflammatory (Immune C3) |
| TCGA-A8-A06X-01 | IFN-gamma Dominant (Immune C2) |
| TCGA-KF-A41W-01 | Wound Healing (Immune C1) |
| TCGA-FG-7638-01 | Immunologically Quiet (Immune C5) |
| TCGA-IB-AAUN-01 | Wound Healing (Immune C1) |
| TCGA-UD-AAC4-01 | Wound Healing (Immune C1) |
| TCGA-KB-A93J-01 | IFN-gamma Dominant (Immune C2) |
| TCGA-49-AAR4-01 | IFN-gamma Dominant (Immune C2) |
| TCGA-29-1763-01 | IFN-gamma Dominant (Immune C2) |
| TCGA-22-4601-01 | Wound Healing (Immune C1) |
| TCGA-DU-7013-01 | Lymphocyte Depleted (Immune C4) |
| TCGA-ET-A40Q-01 | Inflammatory (Immune C3) |
| TCGA-EB-A3Y6-01 | Wound Healing (Immune C1) |
| TCGA-63-A5MB-01 | Wound Healing (Immune C1) |
| TCGA-VN-A88Q-01 | Inflammatory (Immune C3) |
| TCGA-OR-A5JP-01 | Lymphocyte Depleted (Immune C4) |
| TCGA-QF-A5YT-01 | Wound Healing (Immune C1) |
| TCGA-BP-5001-01 | Inflammatory (Immune C3) |
| TCGA-A6-5662-01 | Wound Healing (Immune C1) |
| TCGA-G9-6348-01 | Wound Healing (Immune C1) |
| TCGA-32-1970-01 | Lymphocyte Depleted (Immune C4) |
| TCGA-61-2111-01 | IFN-gamma Dominant (Immune C2) |
| TCGA-BP-4968-01 | Inflammatory (Immune C3) |
| TCGA-DK-AA76-01 | Lymphocyte Depleted (Immune C4) |
| TCGA-DJ-A3V4-01 | Inflammatory (Immune C3) |
| TCGA-L5-A4OO-01 | IFN-gamma Dominant (Immune C2) |
| TCGA-2A-A8VL-01 | Inflammatory (Immune C3) |
| TCGA-V1-A9ZK-01 | Inflammatory (Immune C3) |
| TCGA-43-5670-01 | IFN-gamma Dominant (Immune C2) |
| TCGA-B0-4838-01 | Inflammatory (Immune C3) |
| TCGA-62-A46Y-01 | IFN-gamma Dominant (Immune C2) |
| TCGA-AA-3489-01 | Wound Healing (Immune C1) |
| TCGA-MU-A5YI-01 | IFN-gamma Dominant (Immune C2) |
| TCGA-BQ-5882-01 | Inflammatory (Immune C3) |
| TCGA-66-2758-01 | IFN-gamma Dominant (Immune C2) |
| TCGA-DK-A1AA-01 | Wound Healing (Immune C1) |
| TCGA-DJ-A4UQ-01 | Inflammatory (Immune C3) |
| TCGA-CH-5765-01 | Inflammatory (Immune C3) |
| TCGA-A2-A0CZ-01 | Inflammatory (Immune C3) |
| TCGA-B3-3926-01 | Inflammatory (Immune C3) |
| TCGA-AF-2687-01 | Wound Healing (Immune C1) |
| TCGA-E1-A7Z3-01 | Immunologically Quiet (Immune C5) |
| TCGA-VS-A8QA-01 | IFN-gamma Dominant (Immune C2) |
| TCGA-DD-AAD3-01 | Inflammatory (Immune C3) |
| TCGA-AN-A0XS-01 | Inflammatory (Immune C3) |
| TCGA-G9-6377-01 | Wound Healing (Immune C1) |
| TCGA-DD-A39V-01 | IFN-gamma Dominant (Immune C2) |
| TCGA-AG-A016-01 | Wound Healing (Immune C1) |
| TCGA-EJ-5511-01 | Inflammatory (Immune C3) |
| TCGA-A2-A04Q-01 | IFN-gamma Dominant (Immune C2) |
| TCGA-WA-A7GZ-01 | IFN-gamma Dominant (Immune C2) |
| TCGA-HT-7476-01 | Lymphocyte Depleted (Immune C4) |
| TCGA-AZ-6599-01 | Lymphocyte Depleted (Immune C4) |
| TCGA-BR-8381-01 | IFN-gamma Dominant (Immune C2) |
| TCGA-K1-A3PN-01 | Wound Healing (Immune C1) |
| TCGA-ED-A97K-01 | Inflammatory (Immune C3) |
| TCGA-2G-AAGV-01 | Wound Healing (Immune C1) |
| TCGA-CH-5788-01 | Lymphocyte Depleted (Immune C4) |
| TCGA-HZ-A9TJ-01 | Wound Healing (Immune C1) |
| TCGA-TQ-A7RG-01 | Immunologically Quiet (Immune C5) |
| TCGA-D1-A15X-01 | IFN-gamma Dominant (Immune C2) |
| TCGA-94-7557-01 | Wound Healing (Immune C1) |
| TCGA-CN-5374-01 | IFN-gamma Dominant (Immune C2) |
| TCGA-G4-6299-01 | IFN-gamma Dominant (Immune C2) |
| TCGA-98-7454-01 | IFN-gamma Dominant (Immune C2) |
| TCGA-DM-A1D8-01 | Wound Healing (Immune C1) |
| TCGA-E2-A109-01 | IFN-gamma Dominant (Immune C2) |
| TCGA-G9-6336-01 | Inflammatory (Immune C3) |
| TCGA-V1-A8MG-01 | Inflammatory (Immune C3) |
| TCGA-AC-A5XS-01 | IFN-gamma Dominant (Immune C2) |
| TCGA-FD-A6TF-01 | Wound Healing (Immune C1) |
| TCGA-AX-A064-01 | Wound Healing (Immune C1) |
| TCGA-FG-8181-01 | Immunologically Quiet (Immune C5) |
| TCGA-13-1410-01 | IFN-gamma Dominant (Immune C2) |
| TCGA-B6-A1KF-01 | Wound Healing (Immune C1) |
| TCGA-BT-A20W-01 | Wound Healing (Immune C1) |
| TCGA-BH-A1FL-01 | Inflammatory (Immune C3) |
| TCGA-IQ-A6SG-01 | Wound Healing (Immune C1) |
| TCGA-B2-A4SR-01 | Inflammatory (Immune C3) |
| TCGA-E9-A1NI-01 | IFN-gamma Dominant (Immune C2) |
| TCGA-33-A5GW-01 | Wound Healing (Immune C1) |
| TCGA-A5-A0R8-01 | IFN-gamma Dominant (Immune C2) |
| TCGA-EM-A1YA-01 | Inflammatory (Immune C3) |
| TCGA-DU-A7T6-01 | Lymphocyte Depleted (Immune C4) |
| TCGA-YU-A90S-01 | IFN-gamma Dominant (Immune C2) |
| TCGA-BC-A69H-01 | Lymphocyte Depleted (Immune C4) |
| TCGA-24-1553-01 | IFN-gamma Dominant (Immune C2) |
| TCGA-DB-A4XC-01 | Immunologically Quiet (Immune C5) |
| TCGA-XE-AAOD-01 | IFN-gamma Dominant (Immune C2) |
| TCGA-A8-A07R-01 | IFN-gamma Dominant (Immune C2) |
| TCGA-62-A46R-01 | IFN-gamma Dominant (Immune C2) |
| TCGA-CJ-4903-01 | Inflammatory (Immune C3) |
| TCGA-EP-A2KA-01 | IFN-gamma Dominant (Immune C2) |
| TCGA-AN-A03Y-01 | Wound Healing (Immune C1) |
| TCGA-A2-A0T7-01 | Wound Healing (Immune C1) |
| TCGA-B5-A3FA-01 | IFN-gamma Dominant (Immune C2) |
| TCGA-EL-A3CL-01 | Inflammatory (Immune C3) |
| TCGA-A2-A0CV-01 | Inflammatory (Immune C3) |
| TCGA-63-A5MV-01 | Wound Healing (Immune C1) |
| TCGA-DX-AB2E-01 | Wound Healing (Immune C1) |
| TCGA-GU-A767-01 | IFN-gamma Dominant (Immune C2) |
| TCGA-DD-AAE2-01 | Inflammatory (Immune C3) |
| TCGA-FG-A4MW-01 | Lymphocyte Depleted (Immune C4) |
| TCGA-BR-6456-01 | IFN-gamma Dominant (Immune C2) |
| TCGA-CC-A5UC-01 | Wound Healing (Immune C1) |
| TCGA-B1-A657-01 | Inflammatory (Immune C3) |
| TCGA-BH-A1FH-01 | Wound Healing (Immune C1) |
| TCGA-F9-A8NY-01 | Inflammatory (Immune C3) |
| TCGA-29-1710-01 | IFN-gamma Dominant (Immune C2) |
| TCGA-W2-A7HD-01 | Lymphocyte Depleted (Immune C4) |
| TCGA-55-6712-01 | Inflammatory (Immune C3) |
| TCGA-2G-AAH2-01 | IFN-gamma Dominant (Immune C2) |
| TCGA-BP-5189-01 | Inflammatory (Immune C3) |
| TCGA-HT-7467-01 | Immunologically Quiet (Immune C5) |
| TCGA-CN-4731-01 | IFN-gamma Dominant (Immune C2) |
| TCGA-D7-A6EV-01 | Wound Healing (Immune C1) |
| TCGA-HC-7818-01 | Inflammatory (Immune C3) |
| TCGA-L5-A8NT-01 | Wound Healing (Immune C1) |
| TCGA-C5-A2M2-01 | Lymphocyte Depleted (Immune C4) |
| TCGA-CJ-4881-01 | Inflammatory (Immune C3) |
| TCGA-3X-AAVA-01 | Inflammatory (Immune C3) |
| TCGA-QR-A70C-01 | Inflammatory (Immune C3) |
| TCGA-IG-A3YA-01 | Wound Healing (Immune C1) |
| TCGA-HZ-A49H-01 | Inflammatory (Immune C3) |
| TCGA-KR-A7K8-01 | Inflammatory (Immune C3) |
| TCGA-EJ-5497-01 | Inflammatory (Immune C3) |
| TCGA-AN-A0XN-01 | TGF-beta Dominant (Immune C6) |
| TCGA-A1-A0SK-01 | Wound Healing (Immune C1) |
| TCGA-BP-4807-01 | Inflammatory (Immune C3) |
| TCGA-AO-A125-01 | Inflammatory (Immune C3) |
| TCGA-XF-A9T5-01 | IFN-gamma Dominant (Immune C2) |
| TCGA-B8-A54G-01 | Inflammatory (Immune C3) |
| TCGA-06-0219-01 | Lymphocyte Depleted (Immune C4) |
| TCGA-PE-A5DC-01 | IFN-gamma Dominant (Immune C2) |
| TCGA-AD-5900-01 | IFN-gamma Dominant (Immune C2) |
| TCGA-EO-A1Y5-01 | IFN-gamma Dominant (Immune C2) |
| TCGA-DH-5142-01 | Immunologically Quiet (Immune C5) |
| TCGA-05-4244-01 | Lymphocyte Depleted (Immune C4) |
| TCGA-AX-A060-01 | Wound Healing (Immune C1) |
| TCGA-XF-A8HE-01 | IFN-gamma Dominant (Immune C2) |
| TCGA-BH-A0AU-01 | IFN-gamma Dominant (Immune C2) |
| TCGA-AK-3450-01 | Inflammatory (Immune C3) |
| TCGA-14-0787-01 | Lymphocyte Depleted (Immune C4) |
| TCGA-E3-A3E0-01 | Inflammatory (Immune C3) |
| TCGA-AF-3400-01 | Lymphocyte Depleted (Immune C4) |
| TCGA-BQ-5890-01 | Lymphocyte Depleted (Immune C4) |
| TCGA-S9-A6U2-01 | Immunologically Quiet (Immune C5) |
| TCGA-L5-A8NW-01 | IFN-gamma Dominant (Immune C2) |
| TCGA-IA-A83T-01 | Inflammatory (Immune C3) |
| TCGA-DX-A1KU-01 | TGF-beta Dominant (Immune C6) |
| TCGA-GC-A3RB-01 | IFN-gamma Dominant (Immune C2) |
| TCGA-S6-A8JW-01 | IFN-gamma Dominant (Immune C2) |
| TCGA-CC-A7IE-01 | IFN-gamma Dominant (Immune C2) |
| TCGA-SU-A7E7-01 | Inflammatory (Immune C3) |
| TCGA-12-0618-01 | Lymphocyte Depleted (Immune C4) |
| TCGA-BJ-A28X-01 | Inflammatory (Immune C3) |
| TCGA-L9-A8F4-01 | IFN-gamma Dominant (Immune C2) |
| TCGA-G7-6795-01 | Inflammatory (Immune C3) |
| TCGA-H5-A2HR-01 | IFN-gamma Dominant (Immune C2) |
| TCGA-B0-4811-01 | Inflammatory (Immune C3) |
| TCGA-27-2524-01 | Lymphocyte Depleted (Immune C4) |
| TCGA-GM-A2DB-01 | IFN-gamma Dominant (Immune C2) |
| TCGA-EC-A1NJ-01 | IFN-gamma Dominant (Immune C2) |
| TCGA-VN-A88N-01 | Inflammatory (Immune C3) |
| TCGA-RD-A8N0-01 | IFN-gamma Dominant (Immune C2) |
| TCGA-TQ-A7RH-01 | Immunologically Quiet (Immune C5) |
| TCGA-L5-A4OI-01 | Lymphocyte Depleted (Immune C4) |
| TCGA-B8-5550-01 | Inflammatory (Immune C3) |
| TCGA-N8-A4PL-01 | Wound Healing (Immune C1) |
| TCGA-4R-AA8I-01 | Lymphocyte Depleted (Immune C4) |
| TCGA-VF-A8A8-01 | Wound Healing (Immune C1) |
| TCGA-E9-A244-01 | Wound Healing (Immune C1) |
| TCGA-CG-4442-01 | Wound Healing (Immune C1) |
| TCGA-S7-A7X0-01 | Inflammatory (Immune C3) |
| TCGA-DQ-7593-01 | IFN-gamma Dominant (Immune C2) |
| TCGA-A8-A0AB-01 | Wound Healing (Immune C1) |
| TCGA-55-7726-01 | Wound Healing (Immune C1) |
| TCGA-2E-A9G8-01 | IFN-gamma Dominant (Immune C2) |
| TCGA-A2-A3XV-01 | Wound Healing (Immune C1) |
| TCGA-KV-A74V-01 | Inflammatory (Immune C3) |
| TCGA-A6-5666-01 | Wound Healing (Immune C1) |
| TCGA-08-0386-01 | Lymphocyte Depleted (Immune C4) |
| TCGA-BP-5194-01 | Inflammatory (Immune C3) |
| TCGA-CN-4735-01 | IFN-gamma Dominant (Immune C2) |
| TCGA-91-6849-01 | Inflammatory (Immune C3) |
| TCGA-G9-6356-01 | Inflammatory (Immune C3) |
| TCGA-49-4512-01 | TGF-beta Dominant (Immune C6) |
| TCGA-OR-A5JO-01 | Inflammatory (Immune C3) |
| TCGA-FU-A5XV-01 | IFN-gamma Dominant (Immune C2) |
| TCGA-QR-A70U-01 | Lymphocyte Depleted (Immune C4) |
| TCGA-DB-A75M-01 | Immunologically Quiet (Immune C5) |
| TCGA-DM-A1D7-01 | Wound Healing (Immune C1) |
| TCGA-AN-A04A-01 | Wound Healing (Immune C1) |
| TCGA-HT-8012-01 | Immunologically Quiet (Immune C5) |
| TCGA-AA-A022-01 | IFN-gamma Dominant (Immune C2) |
| TCGA-EX-A8YF-01 | IFN-gamma Dominant (Immune C2) |
| TCGA-EM-A22P-01 | Inflammatory (Immune C3) |
| TCGA-TS-A7OU-01 | Lymphocyte Depleted (Immune C4) |
| TCGA-E2-A14O-01 | Wound Healing (Immune C1) |
| TCGA-A7-A0D9-01 | Wound Healing (Immune C1) |
| TCGA-S9-A7QW-01 | Immunologically Quiet (Immune C5) |
| TCGA-BF-AAOX-01 | Wound Healing (Immune C1) |
| TCGA-P5-A733-01 | Immunologically Quiet (Immune C5) |
| TCGA-66-2770-01 | Wound Healing (Immune C1) |
| TCGA-ED-A7PX-01 | Wound Healing (Immune C1) |
| TCGA-2J-AABK-01 | IFN-gamma Dominant (Immune C2) |
| TCGA-EM-A2CK-01 | Inflammatory (Immune C3) |
| TCGA-2Z-A9JO-01 | Lymphocyte Depleted (Immune C4) |
| TCGA-24-1567-01 | Wound Healing (Immune C1) |
| TCGA-B8-4154-01 | Inflammatory (Immune C3) |
| TCGA-AO-A0JJ-01 | TGF-beta Dominant (Immune C6) |
| TCGA-AA-A02Y-01 | Wound Healing (Immune C1) |
| TCGA-CJ-4890-01 | Inflammatory (Immune C3) |
| TCGA-DX-A240-01 | Wound Healing (Immune C1) |
| TCGA-MX-A666-01 | Wound Healing (Immune C1) |
| TCGA-3A-A9J0-01 | Wound Healing (Immune C1) |
| TCGA-34-5241-01 | IFN-gamma Dominant (Immune C2) |
| TCGA-E1-A7Z4-01 | Immunologically Quiet (Immune C5) |
| TCGA-DU-A5TR-01 | Lymphocyte Depleted (Immune C4) |
| TCGA-75-6203-01 | Inflammatory (Immune C3) |
| TCGA-77-8133-01 | IFN-gamma Dominant (Immune C2) |
| TCGA-28-5204-01 | Lymphocyte Depleted (Immune C4) |
| TCGA-EL-A3H1-01 | Inflammatory (Immune C3) |
| TCGA-36-1577-01 | Wound Healing (Immune C1) |
| TCGA-AG-3887-01 | Wound Healing (Immune C1) |
| TCGA-ZH-A8Y4-01 | Lymphocyte Depleted (Immune C4) |
| TCGA-43-6647-01 | Wound Healing (Immune C1) |
| TCGA-CD-5800-01 | IFN-gamma Dominant (Immune C2) |
| TCGA-SW-A7EA-01 | IFN-gamma Dominant (Immune C2) |
| TCGA-GL-A9DC-01 | Inflammatory (Immune C3) |
| TCGA-ZJ-A8QR-01 | IFN-gamma Dominant (Immune C2) |
| TCGA-V7-A7HQ-01 | Wound Healing (Immune C1) |
| TCGA-AN-A046-01 | IFN-gamma Dominant (Immune C2) |
| TCGA-09-0367-01 | Lymphocyte Depleted (Immune C4) |
| TCGA-FG-8185-01 | Immunologically Quiet (Immune C5) |
| TCGA-BK-A4ZD-01 | Lymphocyte Depleted (Immune C4) |
| TCGA-N5-A4R8-01 | Wound Healing (Immune C1) |
| TCGA-VR-A8ER-01 | Wound Healing (Immune C1) |
| TCGA-FX-A8OO-01 | Wound Healing (Immune C1) |
| TCGA-KS-A41F-01 | Inflammatory (Immune C3) |
| TCGA-XF-AAME-01 | Wound Healing (Immune C1) |
| TCGA-KO-8411-01 | Immunologically Quiet (Immune C5) |
| TCGA-OR-A5JX-01 | Inflammatory (Immune C3) |
| TCGA-73-A9RS-01 | Wound Healing (Immune C1) |
| TCGA-CJ-4893-01 | Inflammatory (Immune C3) |
| TCGA-A3-3329-01 | Inflammatory (Immune C3) |
| TCGA-HW-8320-01 | Immunologically Quiet (Immune C5) |
| TCGA-HZ-A77P-01 | Inflammatory (Immune C3) |
| TCGA-MP-A4TD-01 | Inflammatory (Immune C3) |
| TCGA-EW-A3U0-01 | IFN-gamma Dominant (Immune C2) |
| TCGA-A6-2683-01 | Lymphocyte Depleted (Immune C4) |
| TCGA-EL-A3H5-01 | Inflammatory (Immune C3) |
| TCGA-IA-A40Y-01 | Lymphocyte Depleted (Immune C4) |
| TCGA-FD-A6TE-01 | Wound Healing (Immune C1) |
| TCGA-2A-A8VV-01 | Wound Healing (Immune C1) |
| TCGA-R6-A8W5-01 | Wound Healing (Immune C1) |
| TCGA-UZ-A9PP-01 | Inflammatory (Immune C3) |
| TCGA-L7-A56G-01 | Wound Healing (Immune C1) |
| TCGA-Y8-A898-01 | Lymphocyte Depleted (Immune C4) |
| TCGA-DF-A2KY-01 | IFN-gamma Dominant (Immune C2) |
| TCGA-RW-A67W-01 | Lymphocyte Depleted (Immune C4) |
| TCGA-NJ-A4YG-01 | Inflammatory (Immune C3) |
| TCGA-AO-A0JF-01 | Inflammatory (Immune C3) |
| TCGA-A2-A0T0-01 | Wound Healing (Immune C1) |
| TCGA-BH-A0EA-01 | Inflammatory (Immune C3) |
| TCGA-BT-A20O-01 | IFN-gamma Dominant (Immune C2) |
| TCGA-CS-6667-01 | Immunologically Quiet (Immune C5) |
| TCGA-75-5147-01 | IFN-gamma Dominant (Immune C2) |
| TCGA-KV-A6GE-01 | Inflammatory (Immune C3) |
| TCGA-E2-A572-01 | Lymphocyte Depleted (Immune C4) |
| TCGA-DU-5849-01 | Immunologically Quiet (Immune C5) |
| TCGA-56-7731-01 | Wound Healing (Immune C1) |
| TCGA-61-1740-01 | IFN-gamma Dominant (Immune C2) |
| TCGA-63-A5MN-01 | TGF-beta Dominant (Immune C6) |
| TCGA-DB-5279-01 | Immunologically Quiet (Immune C5) |
| TCGA-D8-A147-01 | IFN-gamma Dominant (Immune C2) |
| TCGA-44-7671-01 | Inflammatory (Immune C3) |
| TCGA-F6-A8O3-01 | Immunologically Quiet (Immune C5) |
| TCGA-LL-A5YN-01 | IFN-gamma Dominant (Immune C2) |
| TCGA-MI-A75C-01 | Lymphocyte Depleted (Immune C4) |
| TCGA-AK-3458-01 | IFN-gamma Dominant (Immune C2) |
| TCGA-NA-A4QV-01 | Wound Healing (Immune C1) |
| TCGA-V5-A7RB-01 | IFN-gamma Dominant (Immune C2) |
| TCGA-DQ-5630-01 | IFN-gamma Dominant (Immune C2) |
| TCGA-06-0190-01 | Lymphocyte Depleted (Immune C4) |
| TCGA-E2-A1LE-01 | Wound Healing (Immune C1) |
| TCGA-38-4631-01 | IFN-gamma Dominant (Immune C2) |
| TCGA-A3-3358-01 | Inflammatory (Immune C3) |
| TCGA-CM-6676-01 | Wound Healing (Immune C1) |
| TCGA-L5-A8NS-01 | Wound Healing (Immune C1) |
| TCGA-CJ-4882-01 | Inflammatory (Immune C3) |
| TCGA-IR-A3LB-01 | IFN-gamma Dominant (Immune C2) |
| TCGA-F1-6177-01 | IFN-gamma Dominant (Immune C2) |
| TCGA-EY-A4KR-01 | IFN-gamma Dominant (Immune C2) |
| TCGA-AG-3878-01 | Inflammatory (Immune C3) |
| TCGA-IB-AAUQ-01 | IFN-gamma Dominant (Immune C2) |
| TCGA-HC-A9TH-01 | IFN-gamma Dominant (Immune C2) |
| TCGA-C8-A26Y-01 | Wound Healing (Immune C1) |
| TCGA-MZ-A7D7-01 | IFN-gamma Dominant (Immune C2) |
| TCGA-D7-8570-01 | IFN-gamma Dominant (Immune C2) |
| TCGA-CV-A6JT-01 | IFN-gamma Dominant (Immune C2) |
| TCGA-B0-4833-01 | Inflammatory (Immune C3) |
| TCGA-NC-A5HF-01 | Wound Healing (Immune C1) |
| TCGA-G4-6626-01 | Wound Healing (Immune C1) |
| TCGA-SR-A6MS-01 | Lymphocyte Depleted (Immune C4) |
| TCGA-A8-A08H-01 | TGF-beta Dominant (Immune C6) |
| TCGA-AN-A0FJ-01 | Wound Healing (Immune C1) |
| TCGA-CG-5723-01 | IFN-gamma Dominant (Immune C2) |
| TCGA-NJ-A55O-01 | Inflammatory (Immune C3) |
| TCGA-EL-A3ZS-01 | Inflammatory (Immune C3) |
| TCGA-DB-A64V-01 | Immunologically Quiet (Immune C5) |
| TCGA-DD-A3A1-01 | Lymphocyte Depleted (Immune C4) |
| TCGA-BH-A1EV-01 | Wound Healing (Immune C1) |
| TCGA-IM-A3EB-01 | IFN-gamma Dominant (Immune C2) |
| TCGA-D7-6519-01 | IFN-gamma Dominant (Immune C2) |
| TCGA-DD-A73E-01 | Inflammatory (Immune C3) |
| TCGA-EK-A2PI-01 | Wound Healing (Immune C1) |
| TCGA-VQ-A8PE-01 | Wound Healing (Immune C1) |
| TCGA-BR-A452-01 | Wound Healing (Immune C1) |
| TCGA-RM-A68T-01 | Inflammatory (Immune C3) |
| TCGA-75-6211-01 | Wound Healing (Immune C1) |
| TCGA-CH-5772-01 | Inflammatory (Immune C3) |
| TCGA-DU-5854-01 | Lymphocyte Depleted (Immune C4) |
| TCGA-UF-A7JV-01 | IFN-gamma Dominant (Immune C2) |
| TCGA-F7-A50I-01 | IFN-gamma Dominant (Immune C2) |
| TCGA-CV-7253-01 | Wound Healing (Immune C1) |
| TCGA-3B-A9I1-01 | Lymphocyte Depleted (Immune C4) |
| TCGA-BF-A5EO-01 | Wound Healing (Immune C1) |
| TCGA-DJ-A3V8-01 | Inflammatory (Immune C3) |
| TCGA-AA-3530-01 | Wound Healing (Immune C1) |
| TCGA-JL-A3YW-01 | Wound Healing (Immune C1) |
| TCGA-L5-A8NH-01 | IFN-gamma Dominant (Immune C2) |
| TCGA-BP-4973-01 | Inflammatory (Immune C3) |
| TCGA-DI-A2QY-01 | Wound Healing (Immune C1) |
| TCGA-A3-3326-01 | Inflammatory (Immune C3) |
| TCGA-HM-A6W2-01 | Wound Healing (Immune C1) |
| TCGA-EK-A2IR-01 | Wound Healing (Immune C1) |
| TCGA-AK-3445-01 | Inflammatory (Immune C3) |
| TCGA-A5-A0VO-01 | Wound Healing (Immune C1) |
| TCGA-UB-A7MB-01 | Lymphocyte Depleted (Immune C4) |
| TCGA-AG-4005-01 | Wound Healing (Immune C1) |
| TCGA-CV-7432-01 | IFN-gamma Dominant (Immune C2) |
| TCGA-D6-6515-01 | IFN-gamma Dominant (Immune C2) |
| TCGA-CJ-4636-01 | Inflammatory (Immune C3) |
| TCGA-IR-A3LF-01 | IFN-gamma Dominant (Immune C2) |
| TCGA-PA-A5YG-01 | Inflammatory (Immune C3) |
| TCGA-90-6837-01 | Wound Healing (Immune C1) |
| TCGA-DK-A3IM-01 | Wound Healing (Immune C1) |
| TCGA-Y8-A897-01 | Inflammatory (Immune C3) |
| TCGA-BR-8382-01 | IFN-gamma Dominant (Immune C2) |
| TCGA-V5-AASW-01 | IFN-gamma Dominant (Immune C2) |
| TCGA-EY-A1GI-01 | Wound Healing (Immune C1) |
| TCGA-AA-A00F-01 | Wound Healing (Immune C1) |
| TCGA-ZS-A9CD-01 | Inflammatory (Immune C3) |
| TCGA-DH-A7UR-01 | Lymphocyte Depleted (Immune C4) |
| TCGA-S7-A7WP-01 | Inflammatory (Immune C3) |
| TCGA-WB-A81W-01 | Lymphocyte Depleted (Immune C4) |
| TCGA-FY-A3NP-01 | Inflammatory (Immune C3) |
| TCGA-S9-A6WL-01 | Lymphocyte Depleted (Immune C4) |
| TCGA-CV-7236-01 | IFN-gamma Dominant (Immune C2) |
| TCGA-CW-5583-01 | Inflammatory (Immune C3) |
| TCGA-XF-AAMJ-01 | IFN-gamma Dominant (Immune C2) |
| TCGA-SX-A7SR-01 | Inflammatory (Immune C3) |
| TCGA-EJ-7331-01 | Inflammatory (Immune C3) |
| TCGA-E2-A15D-01 | Inflammatory (Immune C3) |
| TCGA-DU-A5TU-01 | Immunologically Quiet (Immune C5) |
| TCGA-BP-5185-01 | Inflammatory (Immune C3) |
| TCGA-AO-A12D-01 | IFN-gamma Dominant (Immune C2) |
| TCGA-AO-A1KR-01 | IFN-gamma Dominant (Immune C2) |
| TCGA-IG-A3QL-01 | IFN-gamma Dominant (Immune C2) |
| TCGA-CF-A1HR-01 | Wound Healing (Immune C1) |
| TCGA-B9-4114-01 | Inflammatory (Immune C3) |
| TCGA-EM-A4G1-01 | Inflammatory (Immune C3) |
| TCGA-CJ-4897-01 | Inflammatory (Immune C3) |
| TCGA-V4-A9EV-01 | Inflammatory (Immune C3) |
| TCGA-QR-A702-01 | Lymphocyte Depleted (Immune C4) |
| TCGA-HM-A4S6-01 | IFN-gamma Dominant (Immune C2) |
| TCGA-55-1594-01 | IFN-gamma Dominant (Immune C2) |
| TCGA-AX-A06J-01 | Inflammatory (Immune C3) |
| TCGA-SP-A6QJ-01 | Lymphocyte Depleted (Immune C4) |
| TCGA-CF-A9FL-01 | Wound Healing (Immune C1) |
| TCGA-ET-A39N-01 | Inflammatory (Immune C3) |
| TCGA-85-8071-01 | Wound Healing (Immune C1) |
| TCGA-NH-A8F7-01 | Lymphocyte Depleted (Immune C4) |
| TCGA-26-5135-01 | Lymphocyte Depleted (Immune C4) |
| TCGA-S9-A6WE-01 | Immunologically Quiet (Immune C5) |
| TCGA-BH-A18H-01 | IFN-gamma Dominant (Immune C2) |
| TCGA-BH-A0HF-01 | Wound Healing (Immune C1) |
| TCGA-DX-A23R-01 | IFN-gamma Dominant (Immune C2) |
| TCGA-BP-5198-01 | TGF-beta Dominant (Immune C6) |
| TCGA-C8-A12M-01 | Inflammatory (Immune C3) |
| TCGA-FP-8209-01 | Inflammatory (Immune C3) |
| TCGA-BP-4962-01 | Inflammatory (Immune C3) |
| TCGA-V1-A8ML-01 | Inflammatory (Immune C3) |
| TCGA-VS-A8QF-01 | IFN-gamma Dominant (Immune C2) |
| TCGA-21-1076-01 | IFN-gamma Dominant (Immune C2) |
| TCGA-EJ-A46I-01 | Inflammatory (Immune C3) |
| TCGA-4P-AA8J-01 | IFN-gamma Dominant (Immune C2) |
| TCGA-DJ-A3V2-01 | Inflammatory (Immune C3) |
| TCGA-EF-5830-01 | Wound Healing (Immune C1) |
| TCGA-BA-4076-01 | Wound Healing (Immune C1) |
| TCGA-4K-AA1H-01 | IFN-gamma Dominant (Immune C2) |
| TCGA-C8-A26V-01 | IFN-gamma Dominant (Immune C2) |
| TCGA-29-1785-01 | IFN-gamma Dominant (Immune C2) |
| TCGA-E1-5318-01 | Immunologically Quiet (Immune C5) |
| TCGA-56-1622-01 | IFN-gamma Dominant (Immune C2) |
| TCGA-CR-5247-01 | IFN-gamma Dominant (Immune C2) |
| TCGA-H4-A2HO-01 | Inflammatory (Immune C3) |
| TCGA-DE-A69J-01 | Inflammatory (Immune C3) |
| TCGA-CZ-5464-01 | Inflammatory (Immune C3) |
| TCGA-CH-5745-01 | Inflammatory (Immune C3) |
| TCGA-IG-A3YB-01 | Inflammatory (Immune C3) |
| TCGA-DE-A4MD-01 | Inflammatory (Immune C3) |
| TCGA-W2-A7HC-01 | Lymphocyte Depleted (Immune C4) |
| TCGA-IN-A6RS-01 | Wound Healing (Immune C1) |
| TCGA-EL-A3GX-01 | Lymphocyte Depleted (Immune C4) |
| TCGA-XF-A9SP-01 | IFN-gamma Dominant (Immune C2) |
| TCGA-QH-A6XA-01 | Immunologically Quiet (Immune C5) |
| TCGA-WB-A81S-01 | Inflammatory (Immune C3) |
| TCGA-LK-A4O6-01 | Inflammatory (Immune C3) |
| TCGA-24-1604-01 | IFN-gamma Dominant (Immune C2) |
| TCGA-FY-A76V-01 | Inflammatory (Immune C3) |
| TCGA-DS-A1OC-01 | IFN-gamma Dominant (Immune C2) |
| TCGA-RY-A83X-01 | Immunologically Quiet (Immune C5) |
| TCGA-IQ-A61H-01 | Wound Healing (Immune C1) |
| TCGA-CE-A484-01 | Inflammatory (Immune C3) |
| TCGA-E2-A15S-01 | Wound Healing (Immune C1) |
| TCGA-VQ-A94O-01 | Wound Healing (Immune C1) |
| TCGA-E9-A1RI-01 | Wound Healing (Immune C1) |
| TCGA-EA-A5FO-01 | Wound Healing (Immune C1) |
| TCGA-GM-A2DI-01 | Inflammatory (Immune C3) |
| TCGA-E2-A15L-01 | Wound Healing (Immune C1) |
| TCGA-DU-6393-01 | Immunologically Quiet (Immune C5) |
| TCGA-61-2094-01 | IFN-gamma Dominant (Immune C2) |
| TCGA-B9-A5W8-01 | Inflammatory (Immune C3) |
| TCGA-BA-A6DJ-01 | IFN-gamma Dominant (Immune C2) |
| TCGA-CJ-4886-01 | Inflammatory (Immune C3) |
| TCGA-A8-A09T-01 | Lymphocyte Depleted (Immune C4) |
| TCGA-A3-3346-01 | Lymphocyte Depleted (Immune C4) |
| TCGA-B6-A0RV-01 | IFN-gamma Dominant (Immune C2) |
| TCGA-41-3915-01 | Lymphocyte Depleted (Immune C4) |
| TCGA-QR-A706-01 | Inflammatory (Immune C3) |
| TCGA-D8-A27T-01 | IFN-gamma Dominant (Immune C2) |
| TCGA-34-5232-01 | IFN-gamma Dominant (Immune C2) |
| TCGA-KS-A41I-01 | Inflammatory (Immune C3) |
| TCGA-KK-A7B4-01 | Wound Healing (Immune C1) |
| TCGA-50-5941-01 | IFN-gamma Dominant (Immune C2) |
| TCGA-C8-A278-01 | IFN-gamma Dominant (Immune C2) |
| TCGA-C5-A7UE-01 | IFN-gamma Dominant (Immune C2) |
| TCGA-EM-A2CQ-01 | Inflammatory (Immune C3) |
| TCGA-BC-A217-01 | IFN-gamma Dominant (Immune C2) |
| TCGA-CH-5746-01 | Inflammatory (Immune C3) |
| TCGA-AA-3989-01 | Wound Healing (Immune C1) |
| TCGA-A8-A09C-01 | Wound Healing (Immune C1) |
| TCGA-BH-A18L-01 | Lymphocyte Depleted (Immune C4) |
| TCGA-60-2723-01 | Wound Healing (Immune C1) |
| TCGA-QT-A5XN-01 | Inflammatory (Immune C3) |
| TCGA-44-2662-01 | Lymphocyte Depleted (Immune C4) |
| TCGA-XJ-A9DI-01 | Inflammatory (Immune C3) |
| TCGA-DQ-7590-01 | IFN-gamma Dominant (Immune C2) |
| TCGA-A5-A0GB-01 | Wound Healing (Immune C1) |
| TCGA-21-1072-01 | Wound Healing (Immune C1) |
| TCGA-E9-A22A-01 | IFN-gamma Dominant (Immune C2) |
| TCGA-HC-7750-01 | Inflammatory (Immune C3) |
| TCGA-D1-A17C-01 | Inflammatory (Immune C3) |
| TCGA-DD-A3A9-01 | Inflammatory (Immune C3) |
| TCGA-AC-A3YJ-01 | Inflammatory (Immune C3) |
| TCGA-EM-A3AK-01 | Inflammatory (Immune C3) |
| TCGA-CM-4743-01 | IFN-gamma Dominant (Immune C2) |
| TCGA-IW-A3M4-01 | Inflammatory (Immune C3) |
| TCGA-10-0928-01 | Lymphocyte Depleted (Immune C4) |
| TCGA-Z5-AAPL-01 | IFN-gamma Dominant (Immune C2) |
| TCGA-DS-A5RQ-01 | IFN-gamma Dominant (Immune C2) |
| TCGA-CN-4736-01 | IFN-gamma Dominant (Immune C2) |
| TCGA-CZ-5460-01 | Inflammatory (Immune C3) |
| TCGA-B0-4816-01 | Inflammatory (Immune C3) |
| TCGA-BI-A20A-01 | IFN-gamma Dominant (Immune C2) |
| TCGA-VP-A87K-01 | Inflammatory (Immune C3) |
| TCGA-D6-6517-01 | Wound Healing (Immune C1) |
| TCGA-OR-A5L9-01 | Inflammatory (Immune C3) |
| TCGA-NC-A5HN-01 | IFN-gamma Dominant (Immune C2) |
| TCGA-VQ-AA6B-01 | Wound Healing (Immune C1) |
| TCGA-CN-A63W-01 | IFN-gamma Dominant (Immune C2) |
| TCGA-D8-A1XM-01 | IFN-gamma Dominant (Immune C2) |
| TCGA-DD-A119-01 | Lymphocyte Depleted (Immune C4) |
| TCGA-C5-A7UI-01 | IFN-gamma Dominant (Immune C2) |
| TCGA-60-2720-01 | Wound Healing (Immune C1) |
| TCGA-BH-A1EU-01 | Inflammatory (Immune C3) |
| TCGA-GM-A2DD-01 | IFN-gamma Dominant (Immune C2) |
| TCGA-OL-A5S0-01 | IFN-gamma Dominant (Immune C2) |
| TCGA-E2-A15G-01 | Inflammatory (Immune C3) |
| TCGA-AP-A1DK-01 | Wound Healing (Immune C1) |
| TCGA-VQ-A8PB-01 | Wound Healing (Immune C1) |
| TCGA-SX-A71V-01 | Lymphocyte Depleted (Immune C4) |
| TCGA-DU-6397-01 | Immunologically Quiet (Immune C5) |
| TCGA-LP-A4AW-01 | Wound Healing (Immune C1) |
| TCGA-DJ-A3US-01 | Inflammatory (Immune C3) |
| TCGA-DX-A8BX-01 | Lymphocyte Depleted (Immune C4) |
| TCGA-DU-5853-01 | Immunologically Quiet (Immune C5) |
| TCGA-DJ-A1QH-01 | Inflammatory (Immune C3) |
| TCGA-BS-A0UT-01 | Wound Healing (Immune C1) |
| TCGA-13-1405-01 | Wound Healing (Immune C1) |
| TCGA-EL-A3TA-01 | Inflammatory (Immune C3) |
| TCGA-LA-A7SW-01 | Wound Healing (Immune C1) |
| TCGA-BP-4970-01 | Inflammatory (Immune C3) |
| TCGA-DD-AACI-01 | Inflammatory (Immune C3) |
| TCGA-97-A4M2-01 | Inflammatory (Immune C3) |
| TCGA-CR-7398-01 | IFN-gamma Dominant (Immune C2) |
| TCGA-CH-5771-01 | Inflammatory (Immune C3) |
| TCGA-A2-A0YG-01 | Wound Healing (Immune C1) |
| TCGA-FI-A2D5-01 | Wound Healing (Immune C1) |
| TCGA-BB-7870-01 | Wound Healing (Immune C1) |
| TCGA-A8-A09G-01 | IFN-gamma Dominant (Immune C2) |
| TCGA-G4-6315-01 | Wound Healing (Immune C1) |
| TCGA-HC-7749-01 | Inflammatory (Immune C3) |
| TCGA-CV-7435-01 | IFN-gamma Dominant (Immune C2) |
| TCGA-W5-AA2R-01 | Inflammatory (Immune C3) |
| TCGA-NK-A7XE-01 | Wound Healing (Immune C1) |
| TCGA-V1-A9OX-01 | Inflammatory (Immune C3) |
| TCGA-FJ-A3Z7-01 | Wound Healing (Immune C1) |
| TCGA-AA-3538-01 | Wound Healing (Immune C1) |
| TCGA-EW-A6SA-01 | Wound Healing (Immune C1) |
| TCGA-AX-A1CN-01 | Wound Healing (Immune C1) |
| TCGA-AC-A8OQ-01 | Wound Healing (Immune C1) |
| TCGA-A5-A0GM-01 | Wound Healing (Immune C1) |
| TCGA-HD-8634-01 | IFN-gamma Dominant (Immune C2) |
| TCGA-IN-A6RN-01 | Wound Healing (Immune C1) |
| TCGA-TM-A84J-01 | Lymphocyte Depleted (Immune C4) |
| TCGA-4A-A93Y-01 | Inflammatory (Immune C3) |
| TCGA-AF-6136-01 | Wound Healing (Immune C1) |
| TCGA-B0-4703-01 | TGF-beta Dominant (Immune C6) |
| TCGA-UB-A7ME-01 | Inflammatory (Immune C3) |
| TCGA-CM-4747-01 | Wound Healing (Immune C1) |
| TCGA-AX-A05U-01 | Wound Healing (Immune C1) |
| TCGA-CZ-4856-01 | Inflammatory (Immune C3) |
| TCGA-A1-A0SO-01 | Wound Healing (Immune C1) |
| TCGA-S7-A7WW-01 | Inflammatory (Immune C3) |
| TCGA-R6-A8WG-01 | IFN-gamma Dominant (Immune C2) |
| TCGA-BR-4191-01 | IFN-gamma Dominant (Immune C2) |
| TCGA-XN-A8T3-01 | Wound Healing (Immune C1) |
| TCGA-CN-4728-01 | Wound Healing (Immune C1) |
| TCGA-EA-A3HU-01 | IFN-gamma Dominant (Immune C2) |
| TCGA-FG-5962-01 | Immunologically Quiet (Immune C5) |
| TCGA-VD-AA8P-01 | Inflammatory (Immune C3) |
| TCGA-DY-A1DD-01 | Wound Healing (Immune C1) |
| TCGA-E1-5319-01 | Immunologically Quiet (Immune C5) |
| TCGA-BP-4165-01 | Inflammatory (Immune C3) |
| TCGA-77-A5G1-01 | Wound Healing (Immune C1) |
| TCGA-OR-A5LL-01 | Immunologically Quiet (Immune C5) |
| TCGA-CJ-4876-01 | Inflammatory (Immune C3) |
| TCGA-X6-A7WB-01 | Wound Healing (Immune C1) |
| TCGA-E2-A10B-01 | IFN-gamma Dominant (Immune C2) |
| TCGA-97-A4M1-01 | Inflammatory (Immune C3) |
| TCGA-D8-A1XA-01 | IFN-gamma Dominant (Immune C2) |
| TCGA-AO-A1KQ-01 | IFN-gamma Dominant (Immune C2) |
| TCGA-A8-A0A6-01 | Inflammatory (Immune C3) |
| TCGA-56-A4ZK-01 | Wound Healing (Immune C1) |
| TCGA-L5-A43J-01 | Wound Healing (Immune C1) |
| TCGA-78-7149-01 | Inflammatory (Immune C3) |
| TCGA-AQ-A04J-01 | Wound Healing (Immune C1) |
| TCGA-QT-A5XM-01 | Lymphocyte Depleted (Immune C4) |
| TCGA-37-3789-01 | IFN-gamma Dominant (Immune C2) |
| TCGA-27-1831-01 | Lymphocyte Depleted (Immune C4) |
| TCGA-BH-A0W4-01 | Wound Healing (Immune C1) |
| TCGA-WZ-A7V4-01 | IFN-gamma Dominant (Immune C2) |
| TCGA-EJ-7314-01 | Inflammatory (Immune C3) |
| TCGA-V9-A7HT-01 | Lymphocyte Depleted (Immune C4) |
| TCGA-SP-A6QH-01 | Inflammatory (Immune C3) |
| TCGA-FX-A3RE-01 | Lymphocyte Depleted (Immune C4) |
| TCGA-AA-3519-01 | Wound Healing (Immune C1) |
| TCGA-F6-A8O4-01 | Immunologically Quiet (Immune C5) |
| TCGA-AP-A5FX-01 | IFN-gamma Dominant (Immune C2) |
| TCGA-EK-A2RM-01 | IFN-gamma Dominant (Immune C2) |
| TCGA-28-1753-01 | Lymphocyte Depleted (Immune C4) |
| TCGA-FI-A2D0-01 | IFN-gamma Dominant (Immune C2) |
| TCGA-37-A5EM-01 | IFN-gamma Dominant (Immune C2) |
| TCGA-DK-AA6L-01 | Wound Healing (Immune C1) |
| TCGA-RD-A8N4-01 | IFN-gamma Dominant (Immune C2) |
| TCGA-DD-A4NH-01 | Inflammatory (Immune C3) |
| TCGA-BP-4965-01 | Inflammatory (Immune C3) |
| TCGA-EL-A3H2-01 | Inflammatory (Immune C3) |
| TCGA-HR-A5NC-01 | Lymphocyte Depleted (Immune C4) |
| TCGA-CV-7102-01 | Wound Healing (Immune C1) |
| TCGA-D8-A1X5-01 | Inflammatory (Immune C3) |
| TCGA-DG-A2KK-01 | Wound Healing (Immune C1) |
| TCGA-YU-A912-01 | IFN-gamma Dominant (Immune C2) |
| TCGA-66-2792-01 | Wound Healing (Immune C1) |
| TCGA-12-5295-01 | Lymphocyte Depleted (Immune C4) |
| TCGA-AA-A00K-01 | Wound Healing (Immune C1) |
| TCGA-24-2024-01 | Wound Healing (Immune C1) |
| TCGA-EM-A2P3-01 | Inflammatory (Immune C3) |
| TCGA-GM-A3XG-01 | Wound Healing (Immune C1) |
| TCGA-AR-A2LO-01 | Inflammatory (Immune C3) |
| TCGA-AX-A1CJ-01 | IFN-gamma Dominant (Immune C2) |
| TCGA-HC-7231-01 | Inflammatory (Immune C3) |
| TCGA-SR-A6MP-01 | Inflammatory (Immune C3) |
| TCGA-EL-A3D4-01 | Inflammatory (Immune C3) |
| TCGA-BT-A3PJ-01 | IFN-gamma Dominant (Immune C2) |
| TCGA-B0-4707-01 | Inflammatory (Immune C3) |
| TCGA-VP-AA1N-01 | Inflammatory (Immune C3) |
| TCGA-EY-A1GQ-01 | Wound Healing (Immune C1) |
| TCGA-85-7699-01 | Wound Healing (Immune C1) |
| TCGA-A2-A4RW-01 | Inflammatory (Immune C3) |
| TCGA-RT-A6YC-01 | Inflammatory (Immune C3) |
| TCGA-AZ-6606-01 | IFN-gamma Dominant (Immune C2) |
| TCGA-D8-A1XV-01 | Lymphocyte Depleted (Immune C4) |
| TCGA-D8-A13Y-01 | Lymphocyte Depleted (Immune C4) |
| TCGA-E8-A419-01 | Inflammatory (Immune C3) |
| TCGA-CJ-6031-01 | Inflammatory (Immune C3) |
| TCGA-B1-7332-01 | Inflammatory (Immune C3) |
| TCGA-AR-A0TU-01 | IFN-gamma Dominant (Immune C2) |
| TCGA-EL-A3GS-01 | Lymphocyte Depleted (Immune C4) |
| TCGA-HC-7075-01 | Lymphocyte Depleted (Immune C4) |
| TCGA-AA-3831-01 | Wound Healing (Immune C1) |
| TCGA-UT-A88C-01 | IFN-gamma Dominant (Immune C2) |
| TCGA-QR-A70P-01 | Inflammatory (Immune C3) |
| TCGA-E2-A15P-01 | IFN-gamma Dominant (Immune C2) |
| TCGA-UU-A93S-01 | IFN-gamma Dominant (Immune C2) |
| TCGA-MH-A55Z-01 | Inflammatory (Immune C3) |
| TCGA-EP-A26S-01 | Lymphocyte Depleted (Immune C4) |
| TCGA-MP-A4TC-01 | Wound Healing (Immune C1) |
| TCGA-BH-A0B8-01 | Wound Healing (Immune C1) |
| TCGA-BP-4994-01 | Inflammatory (Immune C3) |
| TCGA-C5-A1MI-01 | IFN-gamma Dominant (Immune C2) |
| TCGA-HT-8107-01 | Immunologically Quiet (Immune C5) |
| TCGA-E9-A1RD-01 | Lymphocyte Depleted (Immune C4) |
| TCGA-BQ-7046-01 | Inflammatory (Immune C3) |
| TCGA-LN-A49U-01 | Wound Healing (Immune C1) |
| TCGA-K4-A5RH-01 | IFN-gamma Dominant (Immune C2) |
| TCGA-P5-A5ET-01 | Immunologically Quiet (Immune C5) |
| TCGA-XV-A9W5-01 | Wound Healing (Immune C1) |
| TCGA-CJ-4640-01 | Inflammatory (Immune C3) |
| TCGA-HT-7880-01 | Immunologically Quiet (Immune C5) |
| TCGA-V1-A8WW-01 | IFN-gamma Dominant (Immune C2) |
| TCGA-AX-A1CE-01 | Wound Healing (Immune C1) |
| TCGA-W5-AA2U-01 | Inflammatory (Immune C3) |
| TCGA-EY-A1GE-01 | Inflammatory (Immune C3) |
| TCGA-NC-A5HM-01 | IFN-gamma Dominant (Immune C2) |
| TCGA-A2-A0ET-01 | Lymphocyte Depleted (Immune C4) |
| TCGA-WE-A8K4-01 | IFN-gamma Dominant (Immune C2) |
| TCGA-21-5783-01 | Wound Healing (Immune C1) |
| TCGA-DJ-A3VD-01 | Inflammatory (Immune C3) |
| TCGA-EM-A4FK-01 | Inflammatory (Immune C3) |
| TCGA-AP-A0L9-01 | IFN-gamma Dominant (Immune C2) |
| TCGA-60-2724-01 | IFN-gamma Dominant (Immune C2) |
| TCGA-AG-A01W-01 | IFN-gamma Dominant (Immune C2) |
| TCGA-DJ-A3V3-01 | Inflammatory (Immune C3) |
| TCGA-HW-7487-01 | Immunologically Quiet (Immune C5) |
| TCGA-2Y-A9H4-01 | Lymphocyte Depleted (Immune C4) |
| TCGA-BT-A0YX-01 | Wound Healing (Immune C1) |
| TCGA-34-8456-01 | Wound Healing (Immune C1) |
| TCGA-XF-A9SK-01 | IFN-gamma Dominant (Immune C2) |
| TCGA-QR-A70G-01 | Lymphocyte Depleted (Immune C4) |
| TCGA-E2-A15R-01 | Inflammatory (Immune C3) |
| TCGA-A3-3325-01 | Inflammatory (Immune C3) |
| TCGA-P3-A6SW-01 | IFN-gamma Dominant (Immune C2) |
| TCGA-DM-A285-01 | Wound Healing (Immune C1) |
| TCGA-85-7696-01 | Wound Healing (Immune C1) |
| TCGA-2H-A9GI-01 | IFN-gamma Dominant (Immune C2) |
| TCGA-UY-A9PH-01 | IFN-gamma Dominant (Immune C2) |
| TCGA-AJ-A8CW-01 | Wound Healing (Immune C1) |
| TCGA-D6-A6EP-01 | Wound Healing (Immune C1) |
| TCGA-BP-4974-01 | Inflammatory (Immune C3) |
| TCGA-CN-6994-01 | Wound Healing (Immune C1) |
| TCGA-34-5928-01 | Wound Healing (Immune C1) |
| TCGA-A2-A3KD-01 | Inflammatory (Immune C3) |
| TCGA-UT-A88G-01 | TGF-beta Dominant (Immune C6) |
| TCGA-2G-AAGG-01 | IFN-gamma Dominant (Immune C2) |
| TCGA-KK-A8IC-01 | Inflammatory (Immune C3) |
| TCGA-A6-6142-01 | Wound Healing (Immune C1) |
| TCGA-Y6-A9XI-01 | Inflammatory (Immune C3) |
| TCGA-J8-A3NZ-01 | Inflammatory (Immune C3) |
| TCGA-VQ-AA6J-01 | IFN-gamma Dominant (Immune C2) |
| TCGA-Z6-A8JD-01 | IFN-gamma Dominant (Immune C2) |
| TCGA-CM-6171-01 | Wound Healing (Immune C1) |
| TCGA-A5-A0G1-01 | IFN-gamma Dominant (Immune C2) |
| TCGA-KL-8335-01 | Inflammatory (Immune C3) |
| TCGA-ZF-A9RC-01 | Wound Healing (Immune C1) |
| TCGA-P5-A72W-01 | Immunologically Quiet (Immune C5) |
| TCGA-AP-A1DH-01 | Wound Healing (Immune C1) |
| TCGA-AJ-A3OJ-01 | Wound Healing (Immune C1) |
| TCGA-HT-7884-01 | Immunologically Quiet (Immune C5) |
| TCGA-KO-8415-01 | Inflammatory (Immune C3) |
| TCGA-RW-A680-01 | Lymphocyte Depleted (Immune C4) |
| TCGA-86-8054-01 | Wound Healing (Immune C1) |
| TCGA-W5-AA2Q-01 | Lymphocyte Depleted (Immune C4) |
| TCGA-68-8250-01 | Wound Healing (Immune C1) |
| TCGA-A3-3308-01 | Inflammatory (Immune C3) |
| TCGA-LN-A8I1-01 | IFN-gamma Dominant (Immune C2) |
| TCGA-BS-A0WQ-01 | Wound Healing (Immune C1) |
| TCGA-QR-A6H2-01 | Lymphocyte Depleted (Immune C4) |
| TCGA-A2-A0CO-01 | IFN-gamma Dominant (Immune C2) |
| TCGA-66-2780-01 | IFN-gamma Dominant (Immune C2) |
| TCGA-NH-A50V-01 | Wound Healing (Immune C1) |
| TCGA-BP-4349-01 | Inflammatory (Immune C3) |
| TCGA-CG-4438-01 | IFN-gamma Dominant (Immune C2) |
| TCGA-DD-AAVR-01 | Inflammatory (Immune C3) |
| TCGA-AN-A0FZ-01 | Wound Healing (Immune C1) |
| TCGA-97-A4M5-01 | Inflammatory (Immune C3) |
| TCGA-99-8028-01 | Inflammatory (Immune C3) |
| TCGA-A2-A0YD-01 | Wound Healing (Immune C1) |
| TCGA-DE-A0Y2-01 | Inflammatory (Immune C3) |
| TCGA-BA-6869-01 | Wound Healing (Immune C1) |
| TCGA-BR-4294-01 | Inflammatory (Immune C3) |
| TCGA-CQ-5323-01 | IFN-gamma Dominant (Immune C2) |
| TCGA-EM-A4FO-01 | Inflammatory (Immune C3) |
| TCGA-FI-A2F8-01 | IFN-gamma Dominant (Immune C2) |
| TCGA-BR-6458-01 | IFN-gamma Dominant (Immune C2) |
| TCGA-EJ-A7NK-01 | Inflammatory (Immune C3) |
| TCGA-EM-A3OB-01 | Inflammatory (Immune C3) |
| TCGA-EW-A1J2-01 | Inflammatory (Immune C3) |
| TCGA-C8-A12N-01 | IFN-gamma Dominant (Immune C2) |
| TCGA-Y8-A8S0-01 | Inflammatory (Immune C3) |
| TCGA-B0-4827-01 | Inflammatory (Immune C3) |
| TCGA-P3-A6T8-01 | IFN-gamma Dominant (Immune C2) |
| TCGA-FD-A62P-01 | Wound Healing (Immune C1) |
| TCGA-AF-A56L-01 | Wound Healing (Immune C1) |
| TCGA-09-1674-01 | Wound Healing (Immune C1) |
| TCGA-B0-5703-01 | Inflammatory (Immune C3) |
| TCGA-X6-A7WA-01 | Inflammatory (Immune C3) |
| TCGA-A2-A25F-01 | IFN-gamma Dominant (Immune C2) |
| TCGA-18-3406-01 | IFN-gamma Dominant (Immune C2) |
| TCGA-LN-A4A5-01 | IFN-gamma Dominant (Immune C2) |
| TCGA-EK-A2RA-01 | IFN-gamma Dominant (Immune C2) |
| TCGA-10-0936-01 | IFN-gamma Dominant (Immune C2) |
| TCGA-A8-A0A1-01 | Inflammatory (Immune C3) |
| TCGA-DU-A6S8-01 | Immunologically Quiet (Immune C5) |
| TCGA-S7-A7WO-01 | Inflammatory (Immune C3) |
| TCGA-YY-A8LH-01 | IFN-gamma Dominant (Immune C2) |
| TCGA-BP-4969-01 | Inflammatory (Immune C3) |
| TCGA-CV-5435-01 | Wound Healing (Immune C1) |
| TCGA-CQ-5332-01 | Wound Healing (Immune C1) |
| TCGA-HU-A4H0-01 | IFN-gamma Dominant (Immune C2) |
| TCGA-44-A47B-01 | Inflammatory (Immune C3) |
| TCGA-CH-5767-01 | Inflammatory (Immune C3) |
| TCGA-CG-4469-01 | Lymphocyte Depleted (Immune C4) |
| TCGA-EL-A3N3-01 | Inflammatory (Immune C3) |
| TCGA-EW-A1IZ-01 | IFN-gamma Dominant (Immune C2) |
| TCGA-AP-A051-01 | Wound Healing (Immune C1) |
| TCGA-A6-2675-01 | IFN-gamma Dominant (Immune C2) |
| TCGA-WB-A81P-01 | Inflammatory (Immune C3) |
| TCGA-D1-A0ZP-01 | IFN-gamma Dominant (Immune C2) |
| TCGA-2Y-A9GX-01 | Inflammatory (Immune C3) |
| TCGA-KL-8331-01 | Inflammatory (Immune C3) |
| TCGA-CN-4726-01 | Wound Healing (Immune C1) |
| TCGA-AA-3862-01 | IFN-gamma Dominant (Immune C2) |
| TCGA-B5-A11Z-01 | Wound Healing (Immune C1) |
| TCGA-A8-A08Z-01 | TGF-beta Dominant (Immune C6) |
| TCGA-VR-AA7D-01 | IFN-gamma Dominant (Immune C2) |
| TCGA-V4-A9EK-01 | Inflammatory (Immune C3) |
| TCGA-VQ-A91D-01 | IFN-gamma Dominant (Immune C2) |
| TCGA-BH-A0DT-01 | Inflammatory (Immune C3) |
| TCGA-66-2759-01 | Wound Healing (Immune C1) |
| TCGA-DD-AADQ-01 | Lymphocyte Depleted (Immune C4) |
| TCGA-BG-A18C-01 | IFN-gamma Dominant (Immune C2) |
| TCGA-EK-A2RB-01 | IFN-gamma Dominant (Immune C2) |
| TCGA-AC-A3QQ-01 | Wound Healing (Immune C1) |
| TCGA-LL-A7SZ-01 | IFN-gamma Dominant (Immune C2) |
| TCGA-XF-A9SX-01 | IFN-gamma Dominant (Immune C2) |
| TCGA-FG-A70Y-01 | Immunologically Quiet (Immune C5) |
| TCGA-AA-A00W-01 | Wound Healing (Immune C1) |
| TCGA-F7-A622-01 | IFN-gamma Dominant (Immune C2) |
| TCGA-3C-AALI-01 | Wound Healing (Immune C1) |
| TCGA-EV-5901-01 | Inflammatory (Immune C3) |
| TCGA-IN-A7NR-01 | IFN-gamma Dominant (Immune C2) |
| TCGA-TM-A84R-01 | Immunologically Quiet (Immune C5) |
| TCGA-HU-A4HD-01 | Wound Healing (Immune C1) |
| TCGA-HT-A74L-01 | Immunologically Quiet (Immune C5) |
| TCGA-HW-A5KM-01 | Lymphocyte Depleted (Immune C4) |
| TCGA-EB-A82B-01 | IFN-gamma Dominant (Immune C2) |
| TCGA-HT-7477-01 | Lymphocyte Depleted (Immune C4) |
| TCGA-38-4628-01 | Lymphocyte Depleted (Immune C4) |
| TCGA-G4-6298-01 | Wound Healing (Immune C1) |
| TCGA-AR-A0TV-01 | IFN-gamma Dominant (Immune C2) |
| TCGA-22-1016-01 | IFN-gamma Dominant (Immune C2) |
| TCGA-IB-A5SS-01 | Wound Healing (Immune C1) |
| TCGA-B0-5707-01 | Inflammatory (Immune C3) |
| TCGA-CQ-A4CI-01 | Wound Healing (Immune C1) |
| TCGA-EJ-5495-01 | Inflammatory (Immune C3) |
| TCGA-OR-A5KW-01 | Lymphocyte Depleted (Immune C4) |
| TCGA-BR-8077-01 | IFN-gamma Dominant (Immune C2) |
| TCGA-CM-6163-01 | Wound Healing (Immune C1) |
| TCGA-W5-AA38-01 | Lymphocyte Depleted (Immune C4) |
| TCGA-FG-A87Q-01 | Lymphocyte Depleted (Immune C4) |
| TCGA-E2-A154-01 | Wound Healing (Immune C1) |
| TCGA-BP-4993-01 | Inflammatory (Immune C3) |
| TCGA-85-A4CN-01 | Wound Healing (Immune C1) |
| TCGA-56-A5DS-01 | Wound Healing (Immune C1) |
| TCGA-BQ-5884-01 | Inflammatory (Immune C3) |
| TCGA-FG-A70Z-01 | Lymphocyte Depleted (Immune C4) |
| TCGA-BC-A69I-01 | Inflammatory (Immune C3) |
| TCGA-TM-A84G-01 | Immunologically Quiet (Immune C5) |
| TCGA-B0-5694-01 | Inflammatory (Immune C3) |
| TCGA-C5-A1MJ-01 | Wound Healing (Immune C1) |
| TCGA-DV-5575-01 | Inflammatory (Immune C3) |
| TCGA-CG-4474-01 | IFN-gamma Dominant (Immune C2) |
| TCGA-FG-A4MU-01 | Lymphocyte Depleted (Immune C4) |
| TCGA-K1-A6RV-01 | Lymphocyte Depleted (Immune C4) |
| TCGA-EJ-7784-01 | Inflammatory (Immune C3) |
| TCGA-HC-8256-01 | Lymphocyte Depleted (Immune C4) |
| TCGA-MY-A5BE-01 | IFN-gamma Dominant (Immune C2) |
| TCGA-CZ-5452-01 | Inflammatory (Immune C3) |
| TCGA-67-3774-01 | Inflammatory (Immune C3) |
| TCGA-80-5608-01 | Wound Healing (Immune C1) |
| TCGA-CN-6021-01 | Wound Healing (Immune C1) |
| TCGA-DB-A4XB-01 | Immunologically Quiet (Immune C5) |
| TCGA-B5-A11U-01 | Wound Healing (Immune C1) |
| TCGA-FD-A43X-01 | Lymphocyte Depleted (Immune C4) |
| TCGA-AA-3970-01 | IFN-gamma Dominant (Immune C2) |
| TCGA-DU-6394-01 | Lymphocyte Depleted (Immune C4) |
| TCGA-C5-A1BE-01 | IFN-gamma Dominant (Immune C2) |
| TCGA-ZJ-AAXT-01 | IFN-gamma Dominant (Immune C2) |
| TCGA-FP-7998-01 | IFN-gamma Dominant (Immune C2) |
| TCGA-K4-A3WS-01 | Wound Healing (Immune C1) |
| TCGA-WY-A85D-01 | Immunologically Quiet (Immune C5) |
| TCGA-IG-A4QS-01 | IFN-gamma Dominant (Immune C2) |
| TCGA-25-1329-01 | IFN-gamma Dominant (Immune C2) |
| TCGA-FG-7641-01 | Immunologically Quiet (Immune C5) |
| TCGA-39-5036-01 | Wound Healing (Immune C1) |
| TCGA-ED-A7PZ-01 | Lymphocyte Depleted (Immune C4) |
| TCGA-GM-A4E0-01 | Inflammatory (Immune C3) |
| TCGA-EL-A3CZ-01 | Inflammatory (Immune C3) |
| TCGA-AO-A124-01 | IFN-gamma Dominant (Immune C2) |
| TCGA-S3-A6ZF-01 | IFN-gamma Dominant (Immune C2) |
| TCGA-G2-AA3D-01 | IFN-gamma Dominant (Immune C2) |
| TCGA-CH-5790-01 | Inflammatory (Immune C3) |
| TCGA-OR-A5J9-01 | Inflammatory (Immune C3) |
| TCGA-AM-5820-01 | Lymphocyte Depleted (Immune C4) |
| TCGA-A8-A09D-01 | TGF-beta Dominant (Immune C6) |
| TCGA-60-2707-01 | Wound Healing (Immune C1) |
| TCGA-GU-AATQ-01 | IFN-gamma Dominant (Immune C2) |
| TCGA-X8-AAAR-01 | IFN-gamma Dominant (Immune C2) |
| TCGA-05-4389-01 | IFN-gamma Dominant (Immune C2) |
| TCGA-22-1012-01 | Wound Healing (Immune C1) |
| TCGA-N5-A4RU-01 | Wound Healing (Immune C1) |
| TCGA-AN-A0XO-01 | Wound Healing (Immune C1) |
| TCGA-2Y-A9GW-01 | Inflammatory (Immune C3) |
| TCGA-18-3417-01 | Wound Healing (Immune C1) |
| TCGA-ZG-A8QX-01 | Inflammatory (Immune C3) |
| TCGA-DD-A4NQ-01 | Lymphocyte Depleted (Immune C4) |
| TCGA-GD-A3OQ-01 | IFN-gamma Dominant (Immune C2) |
| TCGA-KK-A8IB-01 | Wound Healing (Immune C1) |
| TCGA-SI-AA8C-01 | Wound Healing (Immune C1) |
| TCGA-IA-A83V-01 | Inflammatory (Immune C3) |
| TCGA-IB-7886-01 | Wound Healing (Immune C1) |
| TCGA-24-1555-01 | Lymphocyte Depleted (Immune C4) |
| TCGA-QR-A7IP-01 | Inflammatory (Immune C3) |
| TCGA-A5-A0G2-01 | Wound Healing (Immune C1) |
| TCGA-MH-A562-01 | Inflammatory (Immune C3) |
| TCGA-BR-8679-01 | IFN-gamma Dominant (Immune C2) |
| TCGA-A2-A4S3-01 | IFN-gamma Dominant (Immune C2) |
| TCGA-13-0916-01 | IFN-gamma Dominant (Immune C2) |
| TCGA-AX-A2HH-01 | Wound Healing (Immune C1) |
| TCGA-VW-A8FI-01 | Lymphocyte Depleted (Immune C4) |
| TCGA-EA-A3QD-01 | IFN-gamma Dominant (Immune C2) |
| TCGA-A2-A0D1-01 | Wound Healing (Immune C1) |
| TCGA-AA-A02O-01 | Wound Healing (Immune C1) |
| TCGA-94-7943-01 | Wound Healing (Immune C1) |
| TCGA-OR-A5L8-01 | Inflammatory (Immune C3) |
| TCGA-AJ-A3IA-01 | IFN-gamma Dominant (Immune C2) |
| TCGA-N8-A4PM-01 | Wound Healing (Immune C1) |
| TCGA-J4-A83L-01 | Inflammatory (Immune C3) |
| TCGA-OR-A5J6-01 | Lymphocyte Depleted (Immune C4) |
| TCGA-AO-A0J3-01 | Lymphocyte Depleted (Immune C4) |
| TCGA-CV-7183-01 | IFN-gamma Dominant (Immune C2) |
| TCGA-KB-A6F7-01 | IFN-gamma Dominant (Immune C2) |
| TCGA-29-1781-01 | IFN-gamma Dominant (Immune C2) |
| TCGA-CG-4304-01 | TGF-beta Dominant (Immune C6) |
| TCGA-KK-A8I6-01 | Inflammatory (Immune C3) |
| TCGA-A2-A0ST-01 | IFN-gamma Dominant (Immune C2) |
| TCGA-BF-A9VF-01 | Wound Healing (Immune C1) |
| TCGA-DX-A8BL-01 | IFN-gamma Dominant (Immune C2) |
| TCGA-E9-A1RE-01 | Wound Healing (Immune C1) |
| TCGA-DD-AAW3-01 | Lymphocyte Depleted (Immune C4) |
| TCGA-B0-4693-01 | Inflammatory (Immune C3) |
| TCGA-IZ-A6M8-01 | Lymphocyte Depleted (Immune C4) |
| TCGA-25-1312-01 | IFN-gamma Dominant (Immune C2) |
| TCGA-A6-6654-01 | Wound Healing (Immune C1) |
| TCGA-C9-A480-01 | IFN-gamma Dominant (Immune C2) |
| TCGA-DD-AACN-01 | Inflammatory (Immune C3) |
| TCGA-VQ-A925-01 | Wound Healing (Immune C1) |
| TCGA-FG-6689-01 | Immunologically Quiet (Immune C5) |
| TCGA-A2-A25A-01 | Wound Healing (Immune C1) |
| TCGA-CH-5794-01 | Inflammatory (Immune C3) |
| TCGA-IB-A6UF-01 | Wound Healing (Immune C1) |
| TCGA-A5-A1OK-01 | Inflammatory (Immune C3) |
| TCGA-CK-5912-01 | Wound Healing (Immune C1) |
| TCGA-D1-A17S-01 | Inflammatory (Immune C3) |
| TCGA-DB-5277-01 | Lymphocyte Depleted (Immune C4) |
| TCGA-2H-A9GQ-01 | Wound Healing (Immune C1) |
| TCGA-C8-A1HK-01 | IFN-gamma Dominant (Immune C2) |
| TCGA-GL-A59R-01 | Lymphocyte Depleted (Immune C4) |
| TCGA-EL-A3MX-01 | Inflammatory (Immune C3) |
| TCGA-63-A5MG-01 | Wound Healing (Immune C1) |
| TCGA-AG-3580-01 | Wound Healing (Immune C1) |
| TCGA-2G-AAGK-01 | IFN-gamma Dominant (Immune C2) |
| TCGA-SC-A6LM-01 | Lymphocyte Depleted (Immune C4) |
| TCGA-29-1696-01 | IFN-gamma Dominant (Immune C2) |
| TCGA-43-7657-01 | Wound Healing (Immune C1) |
| TCGA-IC-A6RE-01 | IFN-gamma Dominant (Immune C2) |
| TCGA-EB-A44P-01 | Wound Healing (Immune C1) |
| TCGA-AG-4008-01 | Wound Healing (Immune C1) |
| TCGA-6D-AA2E-01 | Inflammatory (Immune C3) |
| TCGA-29-1770-01 | Lymphocyte Depleted (Immune C4) |
| TCGA-CR-7402-01 | Wound Healing (Immune C1) |
| TCGA-A6-6653-01 | Wound Healing (Immune C1) |
| TCGA-OR-A5K3-01 | Lymphocyte Depleted (Immune C4) |
| TCGA-EM-A3O3-01 | Inflammatory (Immune C3) |
| TCGA-HT-A615-01 | Lymphocyte Depleted (Immune C4) |
| TCGA-CZ-5455-01 | Inflammatory (Immune C3) |
| TCGA-EL-A3GV-01 | Inflammatory (Immune C3) |
| TCGA-94-A4VJ-01 | Wound Healing (Immune C1) |
| TCGA-RC-A6M3-01 | Wound Healing (Immune C1) |
| TCGA-HU-A4H8-01 | IFN-gamma Dominant (Immune C2) |
| TCGA-AX-A2HC-01 | Wound Healing (Immune C1) |
| TCGA-BP-4326-01 | Inflammatory (Immune C3) |
| TCGA-BP-4327-01 | Inflammatory (Immune C3) |
| TCGA-E3-A3E5-01 | Inflammatory (Immune C3) |
| TCGA-CM-5349-01 | Wound Healing (Immune C1) |
| TCGA-2J-AAB6-01 | IFN-gamma Dominant (Immune C2) |
| TCGA-DU-7301-01 | Immunologically Quiet (Immune C5) |
| TCGA-AR-A0TR-01 | Inflammatory (Immune C3) |
| TCGA-DJ-A1QL-01 | Inflammatory (Immune C3) |
| TCGA-D1-A15V-01 | Wound Healing (Immune C1) |
| TCGA-DU-6410-01 | Lymphocyte Depleted (Immune C4) |
| TCGA-2W-A8YY-01 | Wound Healing (Immune C1) |
| TCGA-B0-5109-01 | Wound Healing (Immune C1) |
| TCGA-06-2570-01 | Lymphocyte Depleted (Immune C4) |
| TCGA-P5-A5EW-01 | Immunologically Quiet (Immune C5) |
| TCGA-AG-A00C-01 | Wound Healing (Immune C1) |
| TCGA-AG-3574-01 | Wound Healing (Immune C1) |
| TCGA-85-6175-01 | Wound Healing (Immune C1) |
| TCGA-13-1511-01 | IFN-gamma Dominant (Immune C2) |
| TCGA-39-5024-01 | IFN-gamma Dominant (Immune C2) |
| TCGA-20-1687-01 | Lymphocyte Depleted (Immune C4) |
| TCGA-PQ-A6FI-01 | IFN-gamma Dominant (Immune C2) |
| TCGA-EA-A411-01 | Wound Healing (Immune C1) |
| TCGA-AC-A2B8-01 | TGF-beta Dominant (Immune C6) |
| TCGA-B0-5080-01 | Inflammatory (Immune C3) |
| TCGA-06-2569-01 | Wound Healing (Immune C1) |
| TCGA-KM-8439-01 | Lymphocyte Depleted (Immune C4) |
| TCGA-25-1316-01 | Wound Healing (Immune C1) |
| TCGA-C5-A1MN-01 | IFN-gamma Dominant (Immune C2) |
| TCGA-KC-A4BR-01 | Inflammatory (Immune C3) |
| TCGA-H6-8124-01 | Wound Healing (Immune C1) |
| TCGA-5P-A9K3-01 | Lymphocyte Depleted (Immune C4) |
| TCGA-CV-5979-01 | IFN-gamma Dominant (Immune C2) |
| TCGA-DX-A1KZ-01 | Lymphocyte Depleted (Immune C4) |
| TCGA-YL-A9WL-01 | Wound Healing (Immune C1) |
| TCGA-AG-3584-01 | Wound Healing (Immune C1) |
| TCGA-55-6981-01 | Wound Healing (Immune C1) |
| TCGA-D1-A1NX-01 | IFN-gamma Dominant (Immune C2) |
| TCGA-55-7903-01 | IFN-gamma Dominant (Immune C2) |
| TCGA-HT-A5RB-01 | Immunologically Quiet (Immune C5) |
| TCGA-HZ-A8P0-01 | Inflammatory (Immune C3) |
| TCGA-44-2655-01 | IFN-gamma Dominant (Immune C2) |
| TCGA-BA-A4IH-01 | IFN-gamma Dominant (Immune C2) |
| TCGA-BJ-A0Z5-01 | Inflammatory (Immune C3) |
| TCGA-DM-A28A-01 | IFN-gamma Dominant (Immune C2) |
| TCGA-13-2060-01 | IFN-gamma Dominant (Immune C2) |
| TCGA-AA-3527-01 | Wound Healing (Immune C1) |
| TCGA-49-AAR9-01 | Wound Healing (Immune C1) |
| TCGA-B6-A0RL-01 | Lymphocyte Depleted (Immune C4) |
| TCGA-EL-A3CV-01 | Inflammatory (Immune C3) |
| TCGA-DV-5576-01 | Inflammatory (Immune C3) |
| TCGA-09-1668-01 | IFN-gamma Dominant (Immune C2) |
| TCGA-49-4488-01 | IFN-gamma Dominant (Immune C2) |
| TCGA-63-A5ML-01 | IFN-gamma Dominant (Immune C2) |
| TCGA-TS-A7P8-01 | IFN-gamma Dominant (Immune C2) |
| TCGA-22-5477-01 | Wound Healing (Immune C1) |
| TCGA-NF-A5CP-01 | IFN-gamma Dominant (Immune C2) |
| TCGA-55-7283-01 | Inflammatory (Immune C3) |
| TCGA-78-7537-01 | Inflammatory (Immune C3) |
| TCGA-BL-A5ZZ-01 | Wound Healing (Immune C1) |
| TCGA-EL-A3T2-01 | Inflammatory (Immune C3) |
| TCGA-98-A53H-01 | Inflammatory (Immune C3) |
| TCGA-A7-A26G-01 | Wound Healing (Immune C1) |
| TCGA-E2-A1LG-01 | IFN-gamma Dominant (Immune C2) |
| TCGA-N6-A4VD-01 | Wound Healing (Immune C1) |
| TCGA-AA-3819-01 | Wound Healing (Immune C1) |
| TCGA-E7-A5KE-01 | Wound Healing (Immune C1) |
| TCGA-B8-4143-01 | IFN-gamma Dominant (Immune C2) |
| TCGA-24-2271-01 | IFN-gamma Dominant (Immune C2) |
| TCGA-CV-7243-01 | IFN-gamma Dominant (Immune C2) |
| TCGA-S6-A8JY-01 | IFN-gamma Dominant (Immune C2) |
| TCGA-DX-AB2V-01 | Inflammatory (Immune C3) |
| TCGA-FY-A3BL-01 | Inflammatory (Immune C3) |
| TCGA-XK-AAIW-01 | Wound Healing (Immune C1) |
| TCGA-FD-A3SQ-01 | IFN-gamma Dominant (Immune C2) |
| TCGA-CG-4465-01 | IFN-gamma Dominant (Immune C2) |
| TCGA-63-A5MH-01 | Wound Healing (Immune C1) |
| TCGA-DW-7837-01 | Inflammatory (Immune C3) |
| TCGA-E1-A7YN-01 | Lymphocyte Depleted (Immune C4) |
| TCGA-A7-A6VW-01 | IFN-gamma Dominant (Immune C2) |
| TCGA-ZF-A9RD-01 | IFN-gamma Dominant (Immune C2) |
| TCGA-CQ-A4CE-01 | IFN-gamma Dominant (Immune C2) |
| TCGA-29-1699-01 | IFN-gamma Dominant (Immune C2) |
| TCGA-EL-A3CU-01 | Inflammatory (Immune C3) |
| TCGA-61-2109-01 | IFN-gamma Dominant (Immune C2) |
| TCGA-86-A4D0-01 | IFN-gamma Dominant (Immune C2) |
| TCGA-CJ-5684-01 | Inflammatory (Immune C3) |
| TCGA-KH-A6WC-01 | Lymphocyte Depleted (Immune C4) |
| TCGA-60-2704-01 | TGF-beta Dominant (Immune C6) |
| TCGA-MQ-A4KX-01 | IFN-gamma Dominant (Immune C2) |
| TCGA-IB-AAUS-01 | Wound Healing (Immune C1) |
| TCGA-OR-A5JJ-01 | Lymphocyte Depleted (Immune C4) |
| TCGA-KK-A8I9-01 | Wound Healing (Immune C1) |
| TCGA-AH-6549-01 | Wound Healing (Immune C1) |
| TCGA-YL-A9WH-01 | Wound Healing (Immune C1) |
| TCGA-97-7941-01 | Inflammatory (Immune C3) |
| TCGA-LN-A5U7-01 | IFN-gamma Dominant (Immune C2) |
| TCGA-EM-A22O-01 | Inflammatory (Immune C3) |
| TCGA-CJ-5671-01 | Inflammatory (Immune C3) |
| TCGA-AC-A3W7-01 | Inflammatory (Immune C3) |
| TCGA-3U-A98J-01 | Inflammatory (Immune C3) |
| TCGA-WB-A81A-01 | Immunologically Quiet (Immune C5) |
| TCGA-E2-A1LK-01 | IFN-gamma Dominant (Immune C2) |
| TCGA-EK-A2PK-01 | IFN-gamma Dominant (Immune C2) |
| TCGA-91-6840-01 | IFN-gamma Dominant (Immune C2) |
| TCGA-AA-3869-01 | IFN-gamma Dominant (Immune C2) |
| TCGA-DD-AAVY-01 | Lymphocyte Depleted (Immune C4) |
| TCGA-09-2051-01 | IFN-gamma Dominant (Immune C2) |
| TCGA-NF-A4X2-01 | Wound Healing (Immune C1) |
| TCGA-V1-A9ZI-01 | Inflammatory (Immune C3) |
| TCGA-EI-6507-01 | Wound Healing (Immune C1) |
| TCGA-ET-A39S-01 | Inflammatory (Immune C3) |
| TCGA-EJ-7330-01 | Inflammatory (Immune C3) |
| TCGA-CE-A482-01 | Inflammatory (Immune C3) |
| TCGA-VS-A952-01 | Wound Healing (Immune C1) |
| TCGA-EK-A2IP-01 | IFN-gamma Dominant (Immune C2) |
| TCGA-VN-A88R-01 | Lymphocyte Depleted (Immune C4) |
| TCGA-XV-A9VZ-01 | Wound Healing (Immune C1) |
| TCGA-A3-3370-01 | Inflammatory (Immune C3) |
| TCGA-A6-6649-01 | Wound Healing (Immune C1) |
| TCGA-KM-8441-01 | Inflammatory (Immune C3) |
| TCGA-DK-A1AG-01 | Wound Healing (Immune C1) |
| TCGA-AG-3602-01 | Wound Healing (Immune C1) |
| TCGA-D7-6528-01 | IFN-gamma Dominant (Immune C2) |
| TCGA-SC-AA5Z-01 | TGF-beta Dominant (Immune C6) |
| TCGA-C8-A3M7-01 | Inflammatory (Immune C3) |
| TCGA-KO-8409-01 | Lymphocyte Depleted (Immune C4) |
| TCGA-CJ-4634-01 | Inflammatory (Immune C3) |
| TCGA-27-1830-01 | Lymphocyte Depleted (Immune C4) |
| TCGA-R6-A6Y2-01 | Wound Healing (Immune C1) |
| TCGA-QH-A6XC-01 | Lymphocyte Depleted (Immune C4) |
| TCGA-52-7811-01 | Wound Healing (Immune C1) |
| TCGA-50-5930-01 | Inflammatory (Immune C3) |
| TCGA-CV-7252-01 | IFN-gamma Dominant (Immune C2) |
| TCGA-WB-A815-01 | Inflammatory (Immune C3) |
| TCGA-D8-A1Y3-01 | IFN-gamma Dominant (Immune C2) |
| TCGA-CQ-7063-01 | IFN-gamma Dominant (Immune C2) |
| TCGA-EK-A2PG-01 | Wound Healing (Immune C1) |
| TCGA-QQ-A5V9-01 | TGF-beta Dominant (Immune C6) |
| TCGA-06-2559-01 | Lymphocyte Depleted (Immune C4) |
| TCGA-A8-A07C-01 | IFN-gamma Dominant (Immune C2) |
| TCGA-W4-A7U3-01 | Wound Healing (Immune C1) |
| TCGA-BJ-A0YZ-01 | Inflammatory (Immune C3) |
| TCGA-S9-A6WN-01 | Lymphocyte Depleted (Immune C4) |
| TCGA-93-8067-01 | IFN-gamma Dominant (Immune C2) |
| TCGA-E3-A3E1-01 | Inflammatory (Immune C3) |
| TCGA-A8-A06U-01 | IFN-gamma Dominant (Immune C2) |
| TCGA-S9-A7R2-01 | Lymphocyte Depleted (Immune C4) |
| TCGA-AG-3592-01 | Wound Healing (Immune C1) |
| TCGA-D1-A15Z-01 | Wound Healing (Immune C1) |
| TCGA-FU-A3YQ-01 | IFN-gamma Dominant (Immune C2) |
| TCGA-AX-A3G7-01 | IFN-gamma Dominant (Immune C2) |
| TCGA-BA-A4IG-01 | IFN-gamma Dominant (Immune C2) |
| TCGA-D8-A73W-01 | IFN-gamma Dominant (Immune C2) |
| TCGA-DB-A64L-01 | Lymphocyte Depleted (Immune C4) |
| TCGA-B0-4848-01 | Inflammatory (Immune C3) |
| TCGA-G5-6641-01 | Wound Healing (Immune C1) |
| TCGA-E9-A245-01 | Wound Healing (Immune C1) |
| TCGA-P5-A72X-01 | Immunologically Quiet (Immune C5) |
| TCGA-CR-7377-01 | IFN-gamma Dominant (Immune C2) |
| TCGA-34-2596-01 | Wound Healing (Immune C1) |
| TCGA-53-7626-01 | Inflammatory (Immune C3) |
| TCGA-DK-A3IT-01 | Wound Healing (Immune C1) |
| TCGA-3P-A9WA-01 | Lymphocyte Depleted (Immune C4) |
| TCGA-A4-8515-01 | Lymphocyte Depleted (Immune C4) |
| TCGA-BQ-5887-01 | Inflammatory (Immune C3) |
| TCGA-EM-A2OV-01 | Lymphocyte Depleted (Immune C4) |
| TCGA-M9-A5M8-01 | IFN-gamma Dominant (Immune C2) |
| TCGA-OL-A5D6-01 | Wound Healing (Immune C1) |
| TCGA-D1-A1O8-01 | IFN-gamma Dominant (Immune C2) |
| TCGA-24-1842-01 | IFN-gamma Dominant (Immune C2) |
| TCGA-EI-6508-01 | Wound Healing (Immune C1) |
| TCGA-KC-A7FA-01 | Wound Healing (Immune C1) |
| TCGA-FP-A8CX-01 | IFN-gamma Dominant (Immune C2) |
| TCGA-EM-A1YC-01 | Lymphocyte Depleted (Immune C4) |
| TCGA-04-1364-01 | Wound Healing (Immune C1) |
| TCGA-2J-AAB1-01 | TGF-beta Dominant (Immune C6) |
| TCGA-ND-A4WC-01 | IFN-gamma Dominant (Immune C2) |
| TCGA-FY-A2QD-01 | Inflammatory (Immune C3) |
| TCGA-EJ-A6RA-01 | Inflammatory (Immune C3) |
| TCGA-EJ-5530-01 | Inflammatory (Immune C3) |
| TCGA-BG-A187-01 | Inflammatory (Immune C3) |
| TCGA-XF-AAMR-01 | Wound Healing (Immune C1) |
| TCGA-PG-A914-01 | IFN-gamma Dominant (Immune C2) |
| TCGA-BH-A42V-01 | Inflammatory (Immune C3) |
| TCGA-WS-AB45-01 | TGF-beta Dominant (Immune C6) |
| TCGA-EA-A43B-01 | IFN-gamma Dominant (Immune C2) |
| TCGA-AJ-A23M-01 | Wound Healing (Immune C1) |
| TCGA-BA-5556-01 | IFN-gamma Dominant (Immune C2) |
| TCGA-P4-A5E6-01 | Inflammatory (Immune C3) |
| TCGA-AG-3599-01 | Wound Healing (Immune C1) |
| TCGA-WY-A858-01 | Immunologically Quiet (Immune C5) |
| TCGA-56-8082-01 | Wound Healing (Immune C1) |
| TCGA-EB-A3XD-01 | Inflammatory (Immune C3) |
| TCGA-2Z-A9JS-01 | Inflammatory (Immune C3) |
| TCGA-85-A5B5-01 | Wound Healing (Immune C1) |
| TCGA-CV-A6JE-01 | IFN-gamma Dominant (Immune C2) |
| TCGA-SX-A71W-01 | Inflammatory (Immune C3) |
| TCGA-78-7152-01 | Inflammatory (Immune C3) |
| TCGA-AR-A1AX-01 | IFN-gamma Dominant (Immune C2) |
| TCGA-SI-A71P-01 | Lymphocyte Depleted (Immune C4) |
| TCGA-QR-A6ZZ-01 | Inflammatory (Immune C3) |
| TCGA-IG-A3YC-01 | IFN-gamma Dominant (Immune C2) |
| TCGA-A8-A06Q-01 | Lymphocyte Depleted (Immune C4) |
| TCGA-IE-A4EK-01 | Inflammatory (Immune C3) |
| TCGA-CZ-4863-01 | Inflammatory (Immune C3) |
| TCGA-2G-AAFH-01 | IFN-gamma Dominant (Immune C2) |
| TCGA-E9-A1R0-01 | Inflammatory (Immune C3) |
| TCGA-FK-A3SH-01 | Inflammatory (Immune C3) |
| TCGA-TS-A8AV-01 | IFN-gamma Dominant (Immune C2) |
| TCGA-DS-A1OB-01 | IFN-gamma Dominant (Immune C2) |
| TCGA-AG-A026-01 | Wound Healing (Immune C1) |
| TCGA-55-8619-01 | Inflammatory (Immune C3) |
| TCGA-DW-5560-01 | Inflammatory (Immune C3) |
| TCGA-VS-A959-01 | IFN-gamma Dominant (Immune C2) |
| TCGA-B5-A0K1-01 | Wound Healing (Immune C1) |
| TCGA-22-1011-01 | Wound Healing (Immune C1) |
| TCGA-EK-A2R7-01 | Wound Healing (Immune C1) |
| TCGA-B6-A0RO-01 | IFN-gamma Dominant (Immune C2) |
| TCGA-W2-A7H5-01 | Lymphocyte Depleted (Immune C4) |
| TCGA-CQ-6220-01 | IFN-gamma Dominant (Immune C2) |
| TCGA-CN-4729-01 | Wound Healing (Immune C1) |
| TCGA-BH-A0DZ-01 | Wound Healing (Immune C1) |
| TCGA-E9-A1N5-01 | Wound Healing (Immune C1) |
| TCGA-AD-6548-01 | Wound Healing (Immune C1) |
| TCGA-23-2084-01 | IFN-gamma Dominant (Immune C2) |
| TCGA-CN-5355-01 | Wound Healing (Immune C1) |
| TCGA-24-1556-01 | IFN-gamma Dominant (Immune C2) |
| TCGA-D1-A101-01 | IFN-gamma Dominant (Immune C2) |
| TCGA-OR-A5LJ-01 | Inflammatory (Immune C3) |
| TCGA-MH-A857-01 | Lymphocyte Depleted (Immune C4) |
| TCGA-ED-A5KG-01 | IFN-gamma Dominant (Immune C2) |
| TCGA-AN-A0XP-01 | IFN-gamma Dominant (Immune C2) |
| TCGA-FI-A2D4-01 | Wound Healing (Immune C1) |
| TCGA-ZH-A8Y2-01 | Inflammatory (Immune C3) |
| TCGA-61-1724-01 | IFN-gamma Dominant (Immune C2) |
| TCGA-EW-A6S9-01 | IFN-gamma Dominant (Immune C2) |
| TCGA-ZF-AA52-01 | Wound Healing (Immune C1) |
| TCGA-E7-A541-01 | IFN-gamma Dominant (Immune C2) |
| TCGA-ED-A66X-01 | Inflammatory (Immune C3) |
| TCGA-QR-A6GZ-01 | Inflammatory (Immune C3) |
| TCGA-27-2526-01 | Lymphocyte Depleted (Immune C4) |
| TCGA-H7-A6C4-01 | IFN-gamma Dominant (Immune C2) |
| TCGA-DC-6682-01 | Wound Healing (Immune C1) |
| TCGA-CQ-6218-01 | IFN-gamma Dominant (Immune C2) |
| TCGA-FK-A3SD-01 | Inflammatory (Immune C3) |
| TCGA-EB-A42Y-01 | IFN-gamma Dominant (Immune C2) |
| TCGA-E8-A436-01 | Inflammatory (Immune C3) |
| TCGA-B5-A0K2-01 | IFN-gamma Dominant (Immune C2) |
| TCGA-EJ-8470-01 | Inflammatory (Immune C3) |
| TCGA-FU-A2QG-01 | IFN-gamma Dominant (Immune C2) |
| TCGA-E8-A2JQ-01 | Inflammatory (Immune C3) |
| TCGA-A8-A095-01 | Wound Healing (Immune C1) |
| TCGA-UY-A8OB-01 | IFN-gamma Dominant (Immune C2) |
| TCGA-D8-A143-01 | Wound Healing (Immune C1) |
| TCGA-BF-A5EQ-01 | Wound Healing (Immune C1) |
| TCGA-HU-A4H4-01 | IFN-gamma Dominant (Immune C2) |
| TCGA-CN-4737-01 | Wound Healing (Immune C1) |
| TCGA-OR-A5JM-01 | Lymphocyte Depleted (Immune C4) |
| TCGA-BR-8058-01 | IFN-gamma Dominant (Immune C2) |
| TCGA-06-5856-01 | Lymphocyte Depleted (Immune C4) |
| TCGA-EM-A3O6-01 | Inflammatory (Immune C3) |
| TCGA-EM-A3AJ-01 | Inflammatory (Immune C3) |
| TCGA-AA-3509-01 | Wound Healing (Immune C1) |
| TCGA-JW-A5VJ-01 | Wound Healing (Immune C1) |
| TCGA-XF-AAN5-01 | IFN-gamma Dominant (Immune C2) |
| TCGA-A5-A1OH-01 | Wound Healing (Immune C1) |
| TCGA-HT-7608-01 | Immunologically Quiet (Immune C5) |
| TCGA-E7-A97P-01 | IFN-gamma Dominant (Immune C2) |
| TCGA-PE-A5DE-01 | IFN-gamma Dominant (Immune C2) |
| TCGA-S9-A7IQ-01 | Immunologically Quiet (Immune C5) |
| TCGA-HT-7480-01 | Immunologically Quiet (Immune C5) |
| TCGA-BH-A0WA-01 | IFN-gamma Dominant (Immune C2) |
| TCGA-EL-A3ZO-01 | Inflammatory (Immune C3) |
| TCGA-EI-6883-01 | Wound Healing (Immune C1) |
| TCGA-DD-AACH-01 | Lymphocyte Depleted (Immune C4) |
| TCGA-C5-A7UH-01 | Wound Healing (Immune C1) |
| TCGA-2V-A95S-01 | IFN-gamma Dominant (Immune C2) |
| TCGA-WB-A81I-01 | Inflammatory (Immune C3) |
| TCGA-44-A479-01 | IFN-gamma Dominant (Immune C2) |
| TCGA-IW-A3M6-01 | TGF-beta Dominant (Immune C6) |
| TCGA-55-8087-01 | Inflammatory (Immune C3) |
| TCGA-CE-A481-01 | Inflammatory (Immune C3) |
| TCGA-BH-AB28-01 | Inflammatory (Immune C3) |
| TCGA-A8-A09N-01 | Wound Healing (Immune C1) |
| TCGA-NA-A4QW-01 | Wound Healing (Immune C1) |
| TCGA-86-6562-01 | TGF-beta Dominant (Immune C6) |
| TCGA-05-4425-01 | TGF-beta Dominant (Immune C6) |
| TCGA-E2-A15A-01 | IFN-gamma Dominant (Immune C2) |
| TCGA-RW-A67Y-01 | Lymphocyte Depleted (Immune C4) |
| TCGA-GC-A3RD-01 | Wound Healing (Immune C1) |
| TCGA-EM-A3O8-01 | Inflammatory (Immune C3) |
| TCGA-EJ-5526-01 | Inflammatory (Immune C3) |
| TCGA-A5-A0GW-01 | Wound Healing (Immune C1) |
| TCGA-CS-4938-01 | Immunologically Quiet (Immune C5) |
| TCGA-PK-A5HB-01 | Lymphocyte Depleted (Immune C4) |
| TCGA-DJ-A3UR-01 | Inflammatory (Immune C3) |
| TCGA-AG-3999-01 | Wound Healing (Immune C1) |
| TCGA-22-4607-01 | IFN-gamma Dominant (Immune C2) |
| TCGA-BG-A0MK-01 | Inflammatory (Immune C3) |
| TCGA-3B-A9HP-01 | Wound Healing (Immune C1) |
| TCGA-MP-A4SW-01 | IFN-gamma Dominant (Immune C2) |
| TCGA-L5-A4OG-01 | Wound Healing (Immune C1) |
| TCGA-BH-A0HQ-01 | Wound Healing (Immune C1) |
| TCGA-E2-A3DX-01 | Inflammatory (Immune C3) |
| TCGA-HZ-8317-01 | Wound Healing (Immune C1) |
| TCGA-BJ-A4O8-01 | Inflammatory (Immune C3) |
| TCGA-DX-A3LT-01 | TGF-beta Dominant (Immune C6) |
| TCGA-A4-7915-01 | Wound Healing (Immune C1) |
| TCGA-AX-A05W-01 | IFN-gamma Dominant (Immune C2) |
| TCGA-90-A59Q-01 | IFN-gamma Dominant (Immune C2) |
| TCGA-24-1423-01 | IFN-gamma Dominant (Immune C2) |
| TCGA-E7-A7XN-01 | IFN-gamma Dominant (Immune C2) |
| TCGA-CR-6491-01 | IFN-gamma Dominant (Immune C2) |
| TCGA-DX-A6B9-01 | Inflammatory (Immune C3) |
| TCGA-IF-A4AJ-01 | IFN-gamma Dominant (Immune C2) |
| TCGA-AG-3732-01 | Inflammatory (Immune C3) |
| TCGA-HC-7748-01 | Inflammatory (Immune C3) |
| TCGA-E2-A1L6-01 | Wound Healing (Immune C1) |
| TCGA-BP-4766-01 | Inflammatory (Immune C3) |
| TCGA-EY-A1GL-01 | Wound Healing (Immune C1) |
| TCGA-B6-A0I2-01 | IFN-gamma Dominant (Immune C2) |
| TCGA-CV-5443-01 | IFN-gamma Dominant (Immune C2) |
| TCGA-OR-A5JF-01 | Lymphocyte Depleted (Immune C4) |
| TCGA-86-8668-01 | Inflammatory (Immune C3) |
| TCGA-KC-A7FE-01 | Inflammatory (Immune C3) |
| TCGA-55-8512-01 | Inflammatory (Immune C3) |
| TCGA-YU-A94I-01 | Wound Healing (Immune C1) |
| TCGA-B0-5712-01 | Inflammatory (Immune C3) |
| TCGA-13-0920-01 | IFN-gamma Dominant (Immune C2) |
| TCGA-B0-4845-01 | Inflammatory (Immune C3) |
| TCGA-67-3770-01 | IFN-gamma Dominant (Immune C2) |
| TCGA-HE-A5NH-01 | Inflammatory (Immune C3) |
| TCGA-BS-A0TG-01 | IFN-gamma Dominant (Immune C2) |
| TCGA-CR-6484-01 | IFN-gamma Dominant (Immune C2) |
| TCGA-DU-7292-01 | Inflammatory (Immune C3) |
| TCGA-SX-A7SN-01 | Lymphocyte Depleted (Immune C4) |
| TCGA-XE-AAOF-01 | IFN-gamma Dominant (Immune C2) |
| TCGA-CN-6018-01 | IFN-gamma Dominant (Immune C2) |
| TCGA-D1-A175-01 | Wound Healing (Immune C1) |
| TCGA-AZ-4616-01 | IFN-gamma Dominant (Immune C2) |
| TCGA-D8-A73X-01 | Inflammatory (Immune C3) |
| TCGA-GV-A3JX-01 | IFN-gamma Dominant (Immune C2) |
| TCGA-BJ-A28V-01 | Inflammatory (Immune C3) |
| TCGA-MX-A663-01 | Wound Healing (Immune C1) |
| TCGA-S9-A6TU-01 | Immunologically Quiet (Immune C5) |
| TCGA-29-1702-01 | Lymphocyte Depleted (Immune C4) |
| TCGA-B0-4818-01 | Inflammatory (Immune C3) |
| TCGA-DB-A75P-01 | Inflammatory (Immune C3) |
| TCGA-BP-4774-01 | Inflammatory (Immune C3) |
| TCGA-NC-A5HP-01 | Wound Healing (Immune C1) |
| TCGA-33-4566-01 | IFN-gamma Dominant (Immune C2) |
| TCGA-S2-AA1A-01 | Inflammatory (Immune C3) |
| TCGA-RW-A68B-01 | Inflammatory (Immune C3) |
| TCGA-D1-A1O0-01 | IFN-gamma Dominant (Immune C2) |
| TCGA-KO-8405-01 | Inflammatory (Immune C3) |
| TCGA-55-8511-01 | Inflammatory (Immune C3) |
| TCGA-06-0178-01 | Lymphocyte Depleted (Immune C4) |
| TCGA-FY-A3TY-01 | Inflammatory (Immune C3) |
| TCGA-FG-6691-01 | Immunologically Quiet (Immune C5) |
| TCGA-D1-A167-01 | Wound Healing (Immune C1) |
| TCGA-BA-A6DI-01 | IFN-gamma Dominant (Immune C2) |
| TCGA-DD-A1EE-01 | Lymphocyte Depleted (Immune C4) |
| TCGA-77-A5GH-01 | IFN-gamma Dominant (Immune C2) |
| TCGA-EB-A3XC-01 | Wound Healing (Immune C1) |
| TCGA-AX-A05S-01 | IFN-gamma Dominant (Immune C2) |
| TCGA-KK-A8IF-01 | Lymphocyte Depleted (Immune C4) |
| TCGA-4Z-AA83-01 | Wound Healing (Immune C1) |
| TCGA-18-5592-01 | Wound Healing (Immune C1) |
| TCGA-34-8454-01 | Wound Healing (Immune C1) |
| TCGA-3E-AAAZ-01 | Inflammatory (Immune C3) |
| TCGA-BP-4163-01 | Inflammatory (Immune C3) |
| TCGA-CV-6951-01 | IFN-gamma Dominant (Immune C2) |
| TCGA-AA-3952-01 | Wound Healing (Immune C1) |
| TCGA-AR-A252-01 | IFN-gamma Dominant (Immune C2) |
| TCGA-HM-A3JK-01 | Wound Healing (Immune C1) |
| TCGA-EL-A3H8-01 | Inflammatory (Immune C3) |
| TCGA-AR-A0U2-01 | Inflammatory (Immune C3) |
| TCGA-ZF-AA5N-01 | IFN-gamma Dominant (Immune C2) |
| TCGA-AA-A02K-01 | IFN-gamma Dominant (Immune C2) |
| TCGA-63-7021-01 | IFN-gamma Dominant (Immune C2) |
| TCGA-23-1114-01 | Wound Healing (Immune C1) |
| TCGA-85-7698-01 | IFN-gamma Dominant (Immune C2) |
| TCGA-DU-6406-01 | Lymphocyte Depleted (Immune C4) |
| TCGA-SO-A8JP-01 | Wound Healing (Immune C1) |
| TCGA-IB-AAUT-01 | Inflammatory (Immune C3) |
| TCGA-B0-4821-01 | Inflammatory (Immune C3) |
| TCGA-AR-A24L-01 | IFN-gamma Dominant (Immune C2) |
| TCGA-HC-7081-01 | Inflammatory (Immune C3) |
| TCGA-D7-8576-01 | IFN-gamma Dominant (Immune C2) |
| TCGA-B5-A0K6-01 | Inflammatory (Immune C3) |
| TCGA-D1-A168-01 | Lymphocyte Depleted (Immune C4) |
| TCGA-24-1436-01 | IFN-gamma Dominant (Immune C2) |
| TCGA-BR-8368-01 | Wound Healing (Immune C1) |
| TCGA-MH-A560-01 | Lymphocyte Depleted (Immune C4) |
| TCGA-G2-A2EF-01 | IFN-gamma Dominant (Immune C2) |
| TCGA-HC-7736-01 | Inflammatory (Immune C3) |
| TCGA-BP-4989-01 | Inflammatory (Immune C3) |
| TCGA-55-8092-01 | IFN-gamma Dominant (Immune C2) |
| TCGA-FI-A2D2-01 | Lymphocyte Depleted (Immune C4) |
| TCGA-CR-7383-01 | IFN-gamma Dominant (Immune C2) |
| TCGA-BH-A0E2-01 | Wound Healing (Immune C1) |
| TCGA-CQ-6228-01 | IFN-gamma Dominant (Immune C2) |
| TCGA-DJ-A3VF-01 | Inflammatory (Immune C3) |
| TCGA-AP-A0LP-01 | IFN-gamma Dominant (Immune C2) |
| TCGA-36-1581-01 | IFN-gamma Dominant (Immune C2) |
| TCGA-29-1776-01 | IFN-gamma Dominant (Immune C2) |
| TCGA-EL-A4KI-01 | Inflammatory (Immune C3) |
| TCGA-RW-A68F-01 | Lymphocyte Depleted (Immune C4) |
| TCGA-BH-A18V-01 | IFN-gamma Dominant (Immune C2) |
| TCGA-D5-6898-01 | Wound Healing (Immune C1) |
| TCGA-EB-A44N-01 | Wound Healing (Immune C1) |
| TCGA-DJ-A3VM-01 | Inflammatory (Immune C3) |
| TCGA-J9-A8CK-01 | Wound Healing (Immune C1) |
| TCGA-56-7222-01 | IFN-gamma Dominant (Immune C2) |
| TCGA-B6-A0WY-01 | IFN-gamma Dominant (Immune C2) |
| TCGA-L9-A50W-01 | Inflammatory (Immune C3) |
| TCGA-EW-A423-01 | Inflammatory (Immune C3) |
| TCGA-39-5028-01 | IFN-gamma Dominant (Immune C2) |
| TCGA-A6-2685-01 | Wound Healing (Immune C1) |
| TCGA-FE-A23A-01 | Inflammatory (Immune C3) |
| TCGA-BP-4756-01 | Inflammatory (Immune C3) |
| TCGA-22-5482-01 | Wound Healing (Immune C1) |
| TCGA-55-7281-01 | TGF-beta Dominant (Immune C6) |
| TCGA-A3-3324-01 | Inflammatory (Immune C3) |
| TCGA-66-2800-01 | Wound Healing (Immune C1) |
| TCGA-DK-AA77-01 | IFN-gamma Dominant (Immune C2) |
| TCGA-MH-A55W-01 | Inflammatory (Immune C3) |
| TCGA-DX-A7EM-01 | Wound Healing (Immune C1) |
| TCGA-CN-6013-01 | IFN-gamma Dominant (Immune C2) |
| TCGA-A8-A096-01 | TGF-beta Dominant (Immune C6) |
| TCGA-CE-A485-01 | Inflammatory (Immune C3) |
| TCGA-VQ-A8P3-01 | Wound Healing (Immune C1) |
| TCGA-VR-A8EW-01 | Wound Healing (Immune C1) |
| TCGA-LN-A9FO-01 | IFN-gamma Dominant (Immune C2) |
| TCGA-DU-6399-01 | Immunologically Quiet (Immune C5) |
| TCGA-EB-A24D-01 | Wound Healing (Immune C1) |
| TCGA-C8-A12X-01 | IFN-gamma Dominant (Immune C2) |
| TCGA-AA-3517-01 | Wound Healing (Immune C1) |
| TCGA-KN-8432-01 | Lymphocyte Depleted (Immune C4) |
| TCGA-TQ-A7RP-01 | Immunologically Quiet (Immune C5) |
| TCGA-V1-A8WN-01 | Inflammatory (Immune C3) |
| TCGA-SG-A6Z7-01 | Wound Healing (Immune C1) |
| TCGA-E9-A5UO-01 | Lymphocyte Depleted (Immune C4) |
| TCGA-DJ-A3VJ-01 | Wound Healing (Immune C1) |
| TCGA-37-3792-01 | IFN-gamma Dominant (Immune C2) |
| TCGA-F2-A8YN-01 | Wound Healing (Immune C1) |
| TCGA-BH-A18U-01 | IFN-gamma Dominant (Immune C2) |
| TCGA-57-1583-01 | IFN-gamma Dominant (Immune C2) |
| TCGA-BH-A0C0-01 | IFN-gamma Dominant (Immune C2) |
| TCGA-DC-6681-01 | Wound Healing (Immune C1) |
| TCGA-A8-A09I-01 | IFN-gamma Dominant (Immune C2) |
| TCGA-KO-8417-01 | Immunologically Quiet (Immune C5) |
| TCGA-E9-A22D-01 | IFN-gamma Dominant (Immune C2) |
| TCGA-AA-A00E-01 | Wound Healing (Immune C1) |
| TCGA-A4-8630-01 | Inflammatory (Immune C3) |
| TCGA-77-A5GB-01 | Wound Healing (Immune C1) |
| TCGA-SQ-A6I6-01 | Lymphocyte Depleted (Immune C4) |
| TCGA-B0-5110-01 | Inflammatory (Immune C3) |
| TCGA-CV-7414-01 | IFN-gamma Dominant (Immune C2) |
| TCGA-FE-A235-01 | Inflammatory (Immune C3) |
| TCGA-DM-A1HA-01 | Wound Healing (Immune C1) |
| TCGA-UY-A9PF-01 | Wound Healing (Immune C1) |
| TCGA-A5-A2K4-01 | IFN-gamma Dominant (Immune C2) |
| TCGA-AJ-A2QM-01 | IFN-gamma Dominant (Immune C2) |
| TCGA-AJ-A3BD-01 | IFN-gamma Dominant (Immune C2) |
| TCGA-HD-A633-01 | Wound Healing (Immune C1) |
| TCGA-ET-A25G-01 | Inflammatory (Immune C3) |
| TCGA-77-6842-01 | IFN-gamma Dominant (Immune C2) |
| TCGA-CV-7423-01 | IFN-gamma Dominant (Immune C2) |
| TCGA-D8-A27L-01 | Inflammatory (Immune C3) |
| TCGA-05-4426-01 | TGF-beta Dominant (Immune C6) |
| TCGA-BR-7957-01 | Wound Healing (Immune C1) |
| TCGA-05-4390-01 | Wound Healing (Immune C1) |
| TCGA-S9-A7R3-01 | Immunologically Quiet (Immune C5) |
| TCGA-AX-A06H-01 | Wound Healing (Immune C1) |
| TCGA-KD-A5QU-01 | Wound Healing (Immune C1) |
| TCGA-KO-8413-01 | Inflammatory (Immune C3) |
| TCGA-E6-A2P9-01 | Wound Healing (Immune C1) |
| TCGA-3B-A9HX-01 | Wound Healing (Immune C1) |
| TCGA-AA-3688-01 | Wound Healing (Immune C1) |
| TCGA-69-7761-01 | TGF-beta Dominant (Immune C6) |
| TCGA-VQ-A94R-01 | Wound Healing (Immune C1) |
| TCGA-55-8299-01 | TGF-beta Dominant (Immune C6) |
| TCGA-HD-7831-01 | Wound Healing (Immune C1) |
| TCGA-C8-A12O-01 | Wound Healing (Immune C1) |
| TCGA-SC-A6LN-01 | Wound Healing (Immune C1) |
| TCGA-AA-3841-01 | Wound Healing (Immune C1) |
| TCGA-33-4532-01 | Wound Healing (Immune C1) |
| TCGA-13-1505-01 | Wound Healing (Immune C1) |
| TCGA-XE-AAO4-01 | IFN-gamma Dominant (Immune C2) |
| TCGA-95-8494-01 | Lymphocyte Depleted (Immune C4) |
| TCGA-S7-A7WM-01 | Inflammatory (Immune C3) |
| TCGA-DX-A7EI-01 | IFN-gamma Dominant (Immune C2) |
| TCGA-85-A511-01 | IFN-gamma Dominant (Immune C2) |
| TCGA-BA-4074-01 | IFN-gamma Dominant (Immune C2) |
| TCGA-EJ-A7NJ-01 | Lymphocyte Depleted (Immune C4) |
| TCGA-60-2710-01 | Wound Healing (Immune C1) |
| TCGA-QQ-A8VB-01 | Inflammatory (Immune C3) |
| TCGA-BP-4347-01 | Inflammatory (Immune C3) |
| TCGA-F7-8489-01 | IFN-gamma Dominant (Immune C2) |
| TCGA-VQ-AA64-01 | IFN-gamma Dominant (Immune C2) |
| TCGA-DM-A280-01 | Wound Healing (Immune C1) |
| TCGA-D8-A1XG-01 | Lymphocyte Depleted (Immune C4) |
| TCGA-AA-3713-01 | Wound Healing (Immune C1) |
| TCGA-55-8301-01 | IFN-gamma Dominant (Immune C2) |
| TCGA-06-0174-01 | Lymphocyte Depleted (Immune C4) |
| TCGA-S9-A6WI-01 | Immunologically Quiet (Immune C5) |
| TCGA-DD-AACC-01 | IFN-gamma Dominant (Immune C2) |
| TCGA-UZ-A9Q1-01 | Inflammatory (Immune C3) |
| TCGA-C8-A8HQ-01 | Wound Healing (Immune C1) |
| TCGA-DB-A64S-01 | Immunologically Quiet (Immune C5) |
| TCGA-WB-A81Q-01 | Inflammatory (Immune C3) |
| TCGA-AG-A01Y-01 | Wound Healing (Immune C1) |
| TCGA-CV-5434-01 | IFN-gamma Dominant (Immune C2) |
| TCGA-44-A4SU-01 | Inflammatory (Immune C3) |
| TCGA-VS-A9UR-01 | IFN-gamma Dominant (Immune C2) |
| TCGA-E2-A15M-01 | IFN-gamma Dominant (Immune C2) |
| TCGA-AA-3842-01 | Wound Healing (Immune C1) |
| TCGA-A8-A083-01 | Lymphocyte Depleted (Immune C4) |
| TCGA-CQ-6223-01 | IFN-gamma Dominant (Immune C2) |
| TCGA-CW-6090-01 | Inflammatory (Immune C3) |
| TCGA-FG-6692-01 | Lymphocyte Depleted (Immune C4) |
| TCGA-39-5019-01 | Wound Healing (Immune C1) |
| TCGA-EV-5903-01 | Inflammatory (Immune C3) |
| TCGA-DZ-6131-01 | Lymphocyte Depleted (Immune C4) |
| TCGA-D7-6818-01 | Wound Healing (Immune C1) |
| TCGA-OR-A5K9-01 | Lymphocyte Depleted (Immune C4) |
| TCGA-AX-A2H2-01 | IFN-gamma Dominant (Immune C2) |
| TCGA-D7-A4YY-01 | IFN-gamma Dominant (Immune C2) |
| TCGA-HW-7489-01 | Immunologically Quiet (Immune C5) |
| TCGA-R8-A6YH-01 | Immunologically Quiet (Immune C5) |
| TCGA-FY-A3R6-01 | Inflammatory (Immune C3) |
| TCGA-EJ-7785-01 | Inflammatory (Immune C3) |
| TCGA-G9-A9S4-01 | Lymphocyte Depleted (Immune C4) |
| TCGA-HI-7170-01 | Inflammatory (Immune C3) |
| TCGA-06-5417-01 | Lymphocyte Depleted (Immune C4) |
| TCGA-25-1625-01 | Lymphocyte Depleted (Immune C4) |
| TCGA-ZM-AA0F-01 | IFN-gamma Dominant (Immune C2) |
| TCGA-24-1847-01 | IFN-gamma Dominant (Immune C2) |
| TCGA-69-7765-01 | Inflammatory (Immune C3) |
| TCGA-DD-A1EF-01 | Inflammatory (Immune C3) |
| TCGA-OR-A5LM-01 | Lymphocyte Depleted (Immune C4) |
| TCGA-VD-A8KD-01 | Inflammatory (Immune C3) |
| TCGA-CV-6938-01 | IFN-gamma Dominant (Immune C2) |
| TCGA-A6-3807-01 | Wound Healing (Immune C1) |
| TCGA-WB-A81E-01 | Inflammatory (Immune C3) |
| TCGA-26-5134-01 | Lymphocyte Depleted (Immune C4) |
| TCGA-AX-A0IS-01 | Wound Healing (Immune C1) |
| TCGA-OR-A5KZ-01 | Wound Healing (Immune C1) |
| TCGA-AC-A3TN-01 | Inflammatory (Immune C3) |
| TCGA-DD-A73A-01 | Inflammatory (Immune C3) |
| TCGA-56-8629-01 | IFN-gamma Dominant (Immune C2) |
| TCGA-AA-3850-01 | Wound Healing (Immune C1) |
| TCGA-A3-A8OW-01 | Inflammatory (Immune C3) |
| TCGA-SN-A84W-01 | Wound Healing (Immune C1) |
| TCGA-V4-A9F5-01 | Lymphocyte Depleted (Immune C4) |
| TCGA-B0-4822-01 | Inflammatory (Immune C3) |
| TCGA-QR-A70V-01 | Immunologically Quiet (Immune C5) |
| TCGA-BP-4334-01 | Immunologically Quiet (Immune C5) |
| TCGA-B0-5696-01 | Inflammatory (Immune C3) |
| TCGA-05-4433-01 | Inflammatory (Immune C3) |
| TCGA-SA-A6C2-01 | Inflammatory (Immune C3) |
| TCGA-EL-A3GU-01 | Inflammatory (Immune C3) |
| TCGA-DS-A3LQ-01 | IFN-gamma Dominant (Immune C2) |
| TCGA-CV-7099-01 | IFN-gamma Dominant (Immune C2) |
| TCGA-XF-A9SU-01 | Wound Healing (Immune C1) |
| TCGA-55-8508-01 | Inflammatory (Immune C3) |
| TCGA-BC-A10S-01 | Inflammatory (Immune C3) |
| TCGA-DK-A3WY-01 | IFN-gamma Dominant (Immune C2) |
| TCGA-B5-A11O-01 | Wound Healing (Immune C1) |
| TCGA-B6-A0IC-01 | Inflammatory (Immune C3) |
| TCGA-IQ-A61E-01 | IFN-gamma Dominant (Immune C2) |
| TCGA-H6-A45N-01 | Inflammatory (Immune C3) |
| TCGA-F2-A44G-01 | Wound Healing (Immune C1) |
| TCGA-AP-A05P-01 | Wound Healing (Immune C1) |
| TCGA-56-A5DR-01 | Wound Healing (Immune C1) |
| TCGA-QH-A65S-01 | Immunologically Quiet (Immune C5) |
| TCGA-V4-A9EM-01 | Lymphocyte Depleted (Immune C4) |
| TCGA-5N-A9KM-01 | IFN-gamma Dominant (Immune C2) |
| TCGA-ER-A196-01 | Wound Healing (Immune C1) |
| TCGA-D5-6920-01 | Wound Healing (Immune C1) |
| TCGA-QK-A6VB-01 | IFN-gamma Dominant (Immune C2) |
| TCGA-TK-A8OK-01 | Inflammatory (Immune C3) |
| TCGA-2Y-A9H7-01 | Inflammatory (Immune C3) |
| TCGA-A8-A092-01 | IFN-gamma Dominant (Immune C2) |
| TCGA-CQ-7067-01 | IFN-gamma Dominant (Immune C2) |
| TCGA-D8-A1XS-01 | Wound Healing (Immune C1) |
| TCGA-DU-6395-01 | Immunologically Quiet (Immune C5) |
| TCGA-HC-8257-01 | Inflammatory (Immune C3) |
| TCGA-O9-A75Z-01 | Inflammatory (Immune C3) |
| TCGA-5P-A9KE-01 | Inflammatory (Immune C3) |
| TCGA-DK-AA6T-01 | IFN-gamma Dominant (Immune C2) |
| TCGA-BG-A0M7-01 | Wound Healing (Immune C1) |
| TCGA-FC-A4JI-01 | Wound Healing (Immune C1) |
| TCGA-WK-A8XO-01 | Wound Healing (Immune C1) |
| TCGA-50-5044-01 | IFN-gamma Dominant (Immune C2) |
| TCGA-BH-A18Q-01 | IFN-gamma Dominant (Immune C2) |
| TCGA-RW-A685-01 | Inflammatory (Immune C3) |
| TCGA-AO-A12B-01 | Lymphocyte Depleted (Immune C4) |
| TCGA-BR-A4CS-01 | IFN-gamma Dominant (Immune C2) |
| TCGA-BS-A0UJ-01 | Inflammatory (Immune C3) |
| TCGA-76-4925-01 | Lymphocyte Depleted (Immune C4) |
| TCGA-ZJ-AAXU-01 | IFN-gamma Dominant (Immune C2) |
| TCGA-05-4384-01 | Inflammatory (Immune C3) |
| TCGA-EW-A1P3-01 | Wound Healing (Immune C1) |
| TCGA-E5-A4TZ-01 | IFN-gamma Dominant (Immune C2) |
| TCGA-OR-A5K1-01 | Inflammatory (Immune C3) |
| TCGA-2H-A9GL-01 | Wound Healing (Immune C1) |
| TCGA-25-1328-01 | Inflammatory (Immune C3) |
| TCGA-DJ-A3UM-01 | Inflammatory (Immune C3) |
| TCGA-FD-A43N-01 | IFN-gamma Dominant (Immune C2) |
| TCGA-O1-A52J-01 | Inflammatory (Immune C3) |
| TCGA-TM-A84H-01 | Immunologically Quiet (Immune C5) |
| TCGA-DI-A1BU-01 | Wound Healing (Immune C1) |
| TCGA-CR-6472-01 | Wound Healing (Immune C1) |
| TCGA-VS-A9UY-01 | IFN-gamma Dominant (Immune C2) |
| TCGA-B0-4718-01 | Inflammatory (Immune C3) |
| TCGA-N8-A4PP-01 | IFN-gamma Dominant (Immune C2) |
| TCGA-BP-4330-01 | Inflammatory (Immune C3) |
| TCGA-05-4422-01 | IFN-gamma Dominant (Immune C2) |
| TCGA-B0-5692-01 | Inflammatory (Immune C3) |
| TCGA-A7-A26F-01 | IFN-gamma Dominant (Immune C2) |
| TCGA-CK-4952-01 | Wound Healing (Immune C1) |
| TCGA-EM-A3FN-01 | Inflammatory (Immune C3) |
| TCGA-BH-A0B2-01 | IFN-gamma Dominant (Immune C2) |
| TCGA-AO-A12F-01 | Wound Healing (Immune C1) |
| TCGA-61-2092-01 | Lymphocyte Depleted (Immune C4) |
| TCGA-DD-A114-01 | IFN-gamma Dominant (Immune C2) |
| TCGA-CK-5913-01 | Wound Healing (Immune C1) |
| TCGA-28-2510-01 | Lymphocyte Depleted (Immune C4) |
| TCGA-EW-A1P6-01 | Wound Healing (Immune C1) |
| TCGA-S9-A7R8-01 | Immunologically Quiet (Immune C5) |
| TCGA-GM-A2DF-01 | IFN-gamma Dominant (Immune C2) |
| TCGA-E2-A14X-01 | Wound Healing (Immune C1) |
| TCGA-KP-A3W4-01 | IFN-gamma Dominant (Immune C2) |
| TCGA-BJ-A45E-01 | Inflammatory (Immune C3) |
| TCGA-CH-5753-01 | Lymphocyte Depleted (Immune C4) |
| TCGA-BR-8080-01 | Wound Healing (Immune C1) |
| TCGA-F7-A620-01 | IFN-gamma Dominant (Immune C2) |
| TCGA-AJ-A3BG-01 | Lymphocyte Depleted (Immune C4) |
| TCGA-5N-A9KI-01 | IFN-gamma Dominant (Immune C2) |
| TCGA-29-1783-01 | IFN-gamma Dominant (Immune C2) |
| TCGA-24-1424-01 | IFN-gamma Dominant (Immune C2) |
| TCGA-A8-A09Q-01 | IFN-gamma Dominant (Immune C2) |
| TCGA-AC-A2FF-01 | Inflammatory (Immune C3) |
| TCGA-ZJ-AAXA-01 | Wound Healing (Immune C1) |
| TCGA-67-6215-01 | Lymphocyte Depleted (Immune C4) |
| TCGA-4N-A93T-01 | Lymphocyte Depleted (Immune C4) |
| TCGA-W5-AA30-01 | Inflammatory (Immune C3) |
| TCGA-FY-A3R9-01 | Inflammatory (Immune C3) |
| TCGA-BH-A18J-01 | Lymphocyte Depleted (Immune C4) |
| TCGA-2K-A9WE-01 | Inflammatory (Immune C3) |
| TCGA-61-1917-01 | IFN-gamma Dominant (Immune C2) |
| TCGA-H2-A421-01 | Inflammatory (Immune C3) |
| TCGA-YZ-A980-01 | Inflammatory (Immune C3) |
| TCGA-AK-3460-01 | Inflammatory (Immune C3) |
| TCGA-KN-8423-01 | Inflammatory (Immune C3) |
| TCGA-DJ-A3UZ-01 | Inflammatory (Immune C3) |
| TCGA-L5-A4OF-01 | IFN-gamma Dominant (Immune C2) |
| TCGA-EK-A2RO-01 | IFN-gamma Dominant (Immune C2) |
| TCGA-S7-A7WQ-01 | Inflammatory (Immune C3) |
| TCGA-VQ-A91V-01 | IFN-gamma Dominant (Immune C2) |
| TCGA-77-6845-01 | Wound Healing (Immune C1) |
| TCGA-C8-A12W-01 | Wound Healing (Immune C1) |
| TCGA-A2-A0YJ-01 | Wound Healing (Immune C1) |
| TCGA-CN-4723-01 | IFN-gamma Dominant (Immune C2) |
| TCGA-F4-6855-01 | Wound Healing (Immune C1) |
| TCGA-BP-5169-01 | IFN-gamma Dominant (Immune C2) |
| TCGA-WD-A7RX-01 | Inflammatory (Immune C3) |
| TCGA-DD-AACO-01 | Lymphocyte Depleted (Immune C4) |
| TCGA-CV-A6K2-01 | IFN-gamma Dominant (Immune C2) |
| TCGA-G3-A3CI-01 | Inflammatory (Immune C3) |
| TCGA-DU-7019-01 | Immunologically Quiet (Immune C5) |
| TCGA-IB-7893-01 | Wound Healing (Immune C1) |
| TCGA-S9-A7R4-01 | Immunologically Quiet (Immune C5) |
| TCGA-S9-A7J3-01 | Immunologically Quiet (Immune C5) |
| TCGA-MY-A913-01 | IFN-gamma Dominant (Immune C2) |
| TCGA-A3-A8OX-01 | Inflammatory (Immune C3) |
| TCGA-24-1549-01 | IFN-gamma Dominant (Immune C2) |
| TCGA-HC-7740-01 | Inflammatory (Immune C3) |
| TCGA-44-6779-01 | TGF-beta Dominant (Immune C6) |
| TCGA-AA-3846-01 | Wound Healing (Immune C1) |
| TCGA-J7-8537-01 | IFN-gamma Dominant (Immune C2) |
| TCGA-VQ-AA6K-01 | IFN-gamma Dominant (Immune C2) |
| TCGA-30-1718-01 | IFN-gamma Dominant (Immune C2) |
| TCGA-A5-A0GG-01 | IFN-gamma Dominant (Immune C2) |
| TCGA-D8-A1XD-01 | Wound Healing (Immune C1) |
| TCGA-TM-A84L-01 | Immunologically Quiet (Immune C5) |
| TCGA-DJ-A4UW-01 | Inflammatory (Immune C3) |
| TCGA-EX-A1H5-01 | IFN-gamma Dominant (Immune C2) |
| TCGA-66-2734-01 | Lymphocyte Depleted (Immune C4) |
| TCGA-LN-A9FR-01 | Wound Healing (Immune C1) |
| TCGA-C8-A1HJ-01 | IFN-gamma Dominant (Immune C2) |
| TCGA-IQ-A61J-01 | IFN-gamma Dominant (Immune C2) |
| TCGA-BP-4160-01 | Inflammatory (Immune C3) |
| TCGA-EL-A4KH-01 | Inflammatory (Immune C3) |
| TCGA-E1-A7YH-01 | Immunologically Quiet (Immune C5) |
| TCGA-FU-A3EO-01 | IFN-gamma Dominant (Immune C2) |
| TCGA-QG-A5YV-01 | Wound Healing (Immune C1) |
| TCGA-DQ-5625-01 | IFN-gamma Dominant (Immune C2) |
| TCGA-06-5413-01 | Lymphocyte Depleted (Immune C4) |
| TCGA-CV-A45X-01 | IFN-gamma Dominant (Immune C2) |
| TCGA-BH-A0BV-01 | IFN-gamma Dominant (Immune C2) |
| TCGA-ET-A2MZ-01 | Inflammatory (Immune C3) |
| TCGA-CZ-5458-01 | Inflammatory (Immune C3) |
| TCGA-CM-6166-01 | Wound Healing (Immune C1) |
| TCGA-2H-A9GG-01 | Inflammatory (Immune C3) |
| TCGA-77-A5FZ-01 | Inflammatory (Immune C3) |
| TCGA-DD-A1EB-01 | Lymphocyte Depleted (Immune C4) |
| TCGA-AQ-A1H2-01 | Lymphocyte Depleted (Immune C4) |
| TCGA-L5-A4OQ-01 | IFN-gamma Dominant (Immune C2) |
| TCGA-AR-A24O-01 | IFN-gamma Dominant (Immune C2) |
| TCGA-L5-A43C-01 | Wound Healing (Immune C1) |
| TCGA-G4-6321-01 | Lymphocyte Depleted (Immune C4) |
| TCGA-UF-A71D-01 | IFN-gamma Dominant (Immune C2) |
| TCGA-06-0646-01 | Lymphocyte Depleted (Immune C4) |
| TCGA-24-1418-01 | IFN-gamma Dominant (Immune C2) |
| TCGA-ET-A40T-01 | Inflammatory (Immune C3) |
| TCGA-EI-6513-01 | Wound Healing (Immune C1) |
| TCGA-P5-A5EV-01 | Immunologically Quiet (Immune C5) |
| TCGA-BH-A1FE-01 | IFN-gamma Dominant (Immune C2) |
| TCGA-SH-A7BC-01 | TGF-beta Dominant (Immune C6) |
| TCGA-DJ-A4V0-01 | Inflammatory (Immune C3) |
| TCGA-BF-A3DJ-01 | Lymphocyte Depleted (Immune C4) |
| TCGA-D5-6539-01 | Wound Healing (Immune C1) |
| TCGA-J8-A3O0-01 | Inflammatory (Immune C3) |
| TCGA-DD-AADB-01 | IFN-gamma Dominant (Immune C2) |
| TCGA-WT-AB41-01 | IFN-gamma Dominant (Immune C2) |
| TCGA-ZM-AA0E-01 | IFN-gamma Dominant (Immune C2) |
| TCGA-QM-A5NM-01 | Wound Healing (Immune C1) |
| TCGA-EK-A2RK-01 | IFN-gamma Dominant (Immune C2) |
| TCGA-86-8672-01 | IFN-gamma Dominant (Immune C2) |
| TCGA-VQ-A923-01 | IFN-gamma Dominant (Immune C2) |
| TCGA-CR-7388-01 | Wound Healing (Immune C1) |
| TCGA-ET-A3DS-01 | Lymphocyte Depleted (Immune C4) |
| TCGA-A8-A0AD-01 | Lymphocyte Depleted (Immune C4) |
| TCGA-EB-A299-01 | Wound Healing (Immune C1) |
| TCGA-A7-A3J1-01 | IFN-gamma Dominant (Immune C2) |
| TCGA-AP-A0LS-01 | Wound Healing (Immune C1) |
| TCGA-DM-A1D6-01 | Wound Healing (Immune C1) |
| TCGA-A2-A0EQ-01 | IFN-gamma Dominant (Immune C2) |
| TCGA-BR-8373-01 | Inflammatory (Immune C3) |
| TCGA-BP-5004-01 | Inflammatory (Immune C3) |
| TCGA-DX-AB2L-01 | TGF-beta Dominant (Immune C6) |
| TCGA-BR-7901-01 | Wound Healing (Immune C1) |
| TCGA-55-6983-01 | Inflammatory (Immune C3) |
| TCGA-DU-A7TD-01 | Lymphocyte Depleted (Immune C4) |
| TCGA-91-8499-01 | IFN-gamma Dominant (Immune C2) |
| TCGA-VD-A8KG-01 | Inflammatory (Immune C3) |
| TCGA-FX-A48G-01 | Wound Healing (Immune C1) |
| TCGA-UF-A7JD-01 | Wound Healing (Immune C1) |
| TCGA-EJ-A65F-01 | Inflammatory (Immune C3) |
| TCGA-CM-5344-01 | Wound Healing (Immune C1) |
| TCGA-38-7271-01 | TGF-beta Dominant (Immune C6) |
| TCGA-N5-A4RF-01 | IFN-gamma Dominant (Immune C2) |
| TCGA-A6-2674-01 | Wound Healing (Immune C1) |
| TCGA-AJ-A3BH-01 | Wound Healing (Immune C1) |
| TCGA-O2-A52Q-01 | IFN-gamma Dominant (Immune C2) |
| TCGA-LD-A7W6-01 | IFN-gamma Dominant (Immune C2) |
| TCGA-S3-AA12-01 | Inflammatory (Immune C3) |
| TCGA-2Y-A9H1-01 | Lymphocyte Depleted (Immune C4) |
| TCGA-C5-A1BL-01 | IFN-gamma Dominant (Immune C2) |
| TCGA-DX-AB36-01 | Wound Healing (Immune C1) |
| TCGA-ZR-A9CJ-01 | Wound Healing (Immune C1) |
| TCGA-HZ-A8P1-01 | Inflammatory (Immune C3) |
| TCGA-BR-8483-01 | IFN-gamma Dominant (Immune C2) |
| TCGA-L5-A43H-01 | Wound Healing (Immune C1) |
| TCGA-CD-8529-01 | IFN-gamma Dominant (Immune C2) |
| TCGA-Q1-A73S-01 | Wound Healing (Immune C1) |
| TCGA-D5-6536-01 | Lymphocyte Depleted (Immune C4) |
| TCGA-DX-A6YV-01 | IFN-gamma Dominant (Immune C2) |
| TCGA-ET-A3BV-01 | Wound Healing (Immune C1) |
| TCGA-MM-A563-01 | Inflammatory (Immune C3) |
| TCGA-VD-A8K8-01 | Inflammatory (Immune C3) |
| TCGA-AA-3488-01 | Wound Healing (Immune C1) |
| TCGA-EJ-A7NG-01 | Inflammatory (Immune C3) |
| TCGA-CE-A27D-01 | Inflammatory (Immune C3) |
| TCGA-A6-6140-01 | Wound Healing (Immune C1) |
| TCGA-BH-A1FR-01 | Inflammatory (Immune C3) |
| TCGA-BG-A0W1-01 | Lymphocyte Depleted (Immune C4) |
| TCGA-B6-A0IK-01 | Wound Healing (Immune C1) |
| TCGA-BP-5177-01 | Inflammatory (Immune C3) |
| TCGA-EL-A4JW-01 | IFN-gamma Dominant (Immune C2) |
| TCGA-B8-4621-01 | Inflammatory (Immune C3) |
| TCGA-73-4658-01 | TGF-beta Dominant (Immune C6) |
| TCGA-A8-A08B-01 | IFN-gamma Dominant (Immune C2) |
| TCGA-HW-A5KL-01 | Immunologically Quiet (Immune C5) |
| TCGA-A6-A566-01 | Wound Healing (Immune C1) |
| TCGA-F5-6864-01 | Wound Healing (Immune C1) |
| TCGA-XE-AAO3-01 | IFN-gamma Dominant (Immune C2) |
| TCGA-77-A5G7-01 | Wound Healing (Immune C1) |
| TCGA-BF-A3DN-01 | Wound Healing (Immune C1) |
| TCGA-N9-A4Q7-01 | IFN-gamma Dominant (Immune C2) |
| TCGA-ZP-A9CY-01 | Lymphocyte Depleted (Immune C4) |
| TCGA-D8-A1XK-01 | IFN-gamma Dominant (Immune C2) |
| TCGA-A7-A4SA-01 | IFN-gamma Dominant (Immune C2) |
| TCGA-3C-AAAU-01 | Wound Healing (Immune C1) |
| TCGA-86-8076-01 | Inflammatory (Immune C3) |
| TCGA-BR-4280-01 | IFN-gamma Dominant (Immune C2) |
| TCGA-EY-A210-01 | IFN-gamma Dominant (Immune C2) |
| TCGA-24-1844-01 | Lymphocyte Depleted (Immune C4) |
| TCGA-66-2786-01 | IFN-gamma Dominant (Immune C2) |
| TCGA-BH-A0BT-01 | IFN-gamma Dominant (Immune C2) |
| TCGA-VR-AA7I-01 | IFN-gamma Dominant (Immune C2) |
| TCGA-DD-AAVP-01 | Inflammatory (Immune C3) |
| TCGA-BR-8362-01 | Wound Healing (Immune C1) |
| TCGA-HT-7687-01 | Immunologically Quiet (Immune C5) |
| TCGA-77-8144-01 | Wound Healing (Immune C1) |
| TCGA-HU-8249-01 | Wound Healing (Immune C1) |
| TCGA-BR-A4J8-01 | Wound Healing (Immune C1) |
| TCGA-BA-6872-01 | IFN-gamma Dominant (Immune C2) |
| TCGA-G7-7501-01 | Lymphocyte Depleted (Immune C4) |
| TCGA-WN-AB4C-01 | Lymphocyte Depleted (Immune C4) |
| TCGA-CQ-5325-01 | IFN-gamma Dominant (Immune C2) |
| TCGA-DJ-A13S-01 | Inflammatory (Immune C3) |
| TCGA-FD-A3SP-01 | Wound Healing (Immune C1) |
| TCGA-NJ-A4YP-01 | Wound Healing (Immune C1) |
| TCGA-61-1910-01 | Lymphocyte Depleted (Immune C4) |
| TCGA-77-A5G3-01 | Wound Healing (Immune C1) |
| TCGA-B0-5077-01 | Inflammatory (Immune C3) |
| TCGA-AR-A1AJ-01 | IFN-gamma Dominant (Immune C2) |
| TCGA-R6-A6XQ-01 | Wound Healing (Immune C1) |
| TCGA-MT-A67A-01 | IFN-gamma Dominant (Immune C2) |
| TCGA-3U-A98G-01 | TGF-beta Dominant (Immune C6) |
| TCGA-CH-5754-01 | IFN-gamma Dominant (Immune C2) |
| TCGA-BJ-A2NA-01 | Inflammatory (Immune C3) |
| TCGA-PK-A5H8-01 | Lymphocyte Depleted (Immune C4) |
| TCGA-BJ-A0ZG-01 | Inflammatory (Immune C3) |
| TCGA-WY-A85A-01 | Immunologically Quiet (Immune C5) |
| TCGA-CD-A4MG-01 | Wound Healing (Immune C1) |
| TCGA-AA-3971-01 | Wound Healing (Immune C1) |
| TCGA-DH-A66G-01 | Immunologically Quiet (Immune C5) |
| TCGA-AA-3555-01 | Lymphocyte Depleted (Immune C4) |
| TCGA-52-7810-01 | Wound Healing (Immune C1) |
| TCGA-CZ-5453-01 | Inflammatory (Immune C3) |
| TCGA-YZ-A983-01 | Lymphocyte Depleted (Immune C4) |
| TCGA-55-8096-01 | TGF-beta Dominant (Immune C6) |
| TCGA-22-4599-01 | IFN-gamma Dominant (Immune C2) |
| TCGA-D5-5541-01 | Wound Healing (Immune C1) |
| TCGA-AA-3666-01 | Wound Healing (Immune C1) |
| TCGA-BQ-5878-01 | Inflammatory (Immune C3) |
| TCGA-EM-A1CU-01 | Inflammatory (Immune C3) |
| TCGA-EI-6506-01 | Wound Healing (Immune C1) |
| TCGA-AM-5821-01 | IFN-gamma Dominant (Immune C2) |
| TCGA-BH-A0BC-01 | IFN-gamma Dominant (Immune C2) |
| TCGA-G6-A5PC-01 | Inflammatory (Immune C3) |
| TCGA-12-3650-01 | Lymphocyte Depleted (Immune C4) |
| TCGA-AA-3867-01 | Wound Healing (Immune C1) |
| TCGA-AN-A0G0-01 | Wound Healing (Immune C1) |
| TCGA-RW-A689-01 | Lymphocyte Depleted (Immune C4) |
| TCGA-BG-A18A-01 | Inflammatory (Immune C3) |
| TCGA-XF-AAMH-01 | IFN-gamma Dominant (Immune C2) |
| TCGA-ND-A4WA-01 | Wound Healing (Immune C1) |
| TCGA-PN-A8MA-01 | Wound Healing (Immune C1) |
| TCGA-75-5122-01 | IFN-gamma Dominant (Immune C2) |
| TCGA-CC-5262-01 | IFN-gamma Dominant (Immune C2) |
| TCGA-85-8584-01 | IFN-gamma Dominant (Immune C2) |
| TCGA-WB-A818-01 | Lymphocyte Depleted (Immune C4) |
| TCGA-LN-A5U6-01 | Wound Healing (Immune C1) |
| TCGA-LP-A7HU-01 | IFN-gamma Dominant (Immune C2) |
| TCGA-50-5931-01 | Wound Healing (Immune C1) |
| TCGA-DS-A7WF-01 | IFN-gamma Dominant (Immune C2) |
| TCGA-DB-5281-01 | Immunologically Quiet (Immune C5) |
| TCGA-E1-A7YK-01 | Immunologically Quiet (Immune C5) |
| TCGA-2J-AAB8-01 | IFN-gamma Dominant (Immune C2) |
| TCGA-2G-AAGX-01 | Wound Healing (Immune C1) |
| TCGA-CV-6945-01 | IFN-gamma Dominant (Immune C2) |
| TCGA-DK-A2I2-01 | Lymphocyte Depleted (Immune C4) |
| TCGA-KQ-A41O-01 | Wound Healing (Immune C1) |
| TCGA-AD-6901-01 | Wound Healing (Immune C1) |
| TCGA-B6-A401-01 | Wound Healing (Immune C1) |
| TCGA-CM-6161-01 | Wound Healing (Immune C1) |
| TCGA-OR-A5J7-01 | Lymphocyte Depleted (Immune C4) |
| TCGA-T3-A92N-01 | IFN-gamma Dominant (Immune C2) |
| TCGA-E2-A14Q-01 | Wound Healing (Immune C1) |
| TCGA-ES-A2HS-01 | Inflammatory (Immune C3) |
| TCGA-OR-A5LD-01 | Lymphocyte Depleted (Immune C4) |
| TCGA-A2-A1FZ-01 | Inflammatory (Immune C3) |
| TCGA-BF-AAP6-01 | Wound Healing (Immune C1) |
| TCGA-FB-A5VM-01 | IFN-gamma Dominant (Immune C2) |
| TCGA-BQ-5886-01 | Lymphocyte Depleted (Immune C4) |
| TCGA-57-1993-01 | Lymphocyte Depleted (Immune C4) |
| TCGA-DO-A1K0-01 | Inflammatory (Immune C3) |
| TCGA-G9-6384-01 | Inflammatory (Immune C3) |
| TCGA-BK-A0C9-01 | Wound Healing (Immune C1) |
| TCGA-IB-7888-01 | Inflammatory (Immune C3) |
| TCGA-22-1002-01 | Wound Healing (Immune C1) |
| TCGA-AH-6547-01 | Inflammatory (Immune C3) |
| TCGA-25-2400-01 | Lymphocyte Depleted (Immune C4) |
| TCGA-39-5011-01 | IFN-gamma Dominant (Immune C2) |
| TCGA-2G-AAG8-01 | IFN-gamma Dominant (Immune C2) |
| TCGA-21-5786-01 | Wound Healing (Immune C1) |
| TCGA-4Z-AA7Q-01 | IFN-gamma Dominant (Immune C2) |
| TCGA-EL-A3GZ-01 | Inflammatory (Immune C3) |
| TCGA-73-4668-01 | Wound Healing (Immune C1) |
| TCGA-CR-5248-01 | IFN-gamma Dominant (Immune C2) |
| TCGA-B5-A11S-01 | Wound Healing (Immune C1) |
| TCGA-2Z-A9JJ-01 | Inflammatory (Immune C3) |
| TCGA-AA-3814-01 | Wound Healing (Immune C1) |
| TCGA-AR-A1AQ-01 | IFN-gamma Dominant (Immune C2) |
| TCGA-3H-AB3T-01 | IFN-gamma Dominant (Immune C2) |
| TCGA-ZG-A9L0-01 | IFN-gamma Dominant (Immune C2) |
| TCGA-EW-A1P8-01 | IFN-gamma Dominant (Immune C2) |
| TCGA-85-6798-01 | Wound Healing (Immune C1) |
| TCGA-E8-A438-01 | Inflammatory (Immune C3) |
| TCGA-IN-A6RI-01 | IFN-gamma Dominant (Immune C2) |
| TCGA-Y8-A896-01 | Inflammatory (Immune C3) |
| TCGA-L9-A444-01 | Inflammatory (Immune C3) |
| TCGA-G3-A7M9-01 | Wound Healing (Immune C1) |
| TCGA-IH-A3EA-01 | Wound Healing (Immune C1) |
| TCGA-5M-AAT5-01 | Wound Healing (Immune C1) |
| TCGA-EL-A3CX-01 | Lymphocyte Depleted (Immune C4) |
| TCGA-CQ-6229-01 | IFN-gamma Dominant (Immune C2) |
| TCGA-CS-5395-01 | Immunologically Quiet (Immune C5) |
| TCGA-AO-A03U-01 | Inflammatory (Immune C3) |
| TCGA-EJ-A7NN-01 | IFN-gamma Dominant (Immune C2) |
| TCGA-IB-7652-01 | Inflammatory (Immune C3) |
| TCGA-E9-A226-01 | IFN-gamma Dominant (Immune C2) |
| TCGA-CJ-4920-01 | Inflammatory (Immune C3) |
| TCGA-DK-A2I6-01 | Wound Healing (Immune C1) |
| TCGA-CK-6748-01 | Wound Healing (Immune C1) |
| TCGA-05-5425-01 | IFN-gamma Dominant (Immune C2) |
| TCGA-TQ-A7RR-01 | Immunologically Quiet (Immune C5) |
| TCGA-AN-A0XR-01 | IFN-gamma Dominant (Immune C2) |
| TCGA-ZJ-AAXJ-01 | IFN-gamma Dominant (Immune C2) |
| TCGA-24-1557-01 | Lymphocyte Depleted (Immune C4) |
| TCGA-AA-3521-01 | Wound Healing (Immune C1) |
| TCGA-HU-8608-01 | IFN-gamma Dominant (Immune C2) |
| TCGA-B0-5691-01 | Inflammatory (Immune C3) |
| TCGA-F2-A7TX-01 | IFN-gamma Dominant (Immune C2) |
| TCGA-CV-5430-01 | IFN-gamma Dominant (Immune C2) |
| TCGA-BC-A8YO-01 | Lymphocyte Depleted (Immune C4) |
| TCGA-77-8140-01 | Wound Healing (Immune C1) |
| TCGA-HE-A5NF-01 | Lymphocyte Depleted (Immune C4) |
| TCGA-A2-A0YC-01 | IFN-gamma Dominant (Immune C2) |
| TCGA-2G-AAF1-01 | IFN-gamma Dominant (Immune C2) |
| TCGA-A2-A4S1-01 | TGF-beta Dominant (Immune C6) |
| TCGA-44-6775-01 | TGF-beta Dominant (Immune C6) |
| TCGA-QR-A6GW-01 | Inflammatory (Immune C3) |
| TCGA-ZG-A9ND-01 | Inflammatory (Immune C3) |
| TCGA-Q1-A73R-01 | IFN-gamma Dominant (Immune C2) |
| TCGA-P3-A6T0-01 | Wound Healing (Immune C1) |
| TCGA-AX-A3G9-01 | Wound Healing (Immune C1) |
| TCGA-HT-A5R5-01 | Lymphocyte Depleted (Immune C4) |
| TCGA-HZ-8637-01 | IFN-gamma Dominant (Immune C2) |
| TCGA-CV-7263-01 | Wound Healing (Immune C1) |
| TCGA-DK-A6B6-01 | Lymphocyte Depleted (Immune C4) |
| TCGA-75-7025-01 | Inflammatory (Immune C3) |
| TCGA-BR-4370-01 | Wound Healing (Immune C1) |
| TCGA-2Y-A9H3-01 | Inflammatory (Immune C3) |
| TCGA-12-0616-01 | Lymphocyte Depleted (Immune C4) |
| TCGA-BR-8365-01 | Inflammatory (Immune C3) |
| TCGA-T2-A6X0-01 | IFN-gamma Dominant (Immune C2) |
| TCGA-HU-A4H6-01 | IFN-gamma Dominant (Immune C2) |
| TCGA-LN-A8HZ-01 | IFN-gamma Dominant (Immune C2) |
| TCGA-RD-A7C1-01 | IFN-gamma Dominant (Immune C2) |
| TCGA-ZG-A9L4-01 | Wound Healing (Immune C1) |
| TCGA-DQ-5629-01 | IFN-gamma Dominant (Immune C2) |
| TCGA-CU-A72E-01 | Wound Healing (Immune C1) |
| TCGA-C8-A26W-01 | IFN-gamma Dominant (Immune C2) |
| TCGA-AA-3979-01 | IFN-gamma Dominant (Immune C2) |
| TCGA-EJ-A8FS-01 | Lymphocyte Depleted (Immune C4) |
| TCGA-FK-A3SE-01 | Inflammatory (Immune C3) |
| TCGA-QH-A65V-01 | Immunologically Quiet (Immune C5) |
| TCGA-RD-A7BW-01 | TGF-beta Dominant (Immune C6) |
| TCGA-56-8309-01 | TGF-beta Dominant (Immune C6) |
| TCGA-E9-A1RB-01 | Wound Healing (Immune C1) |
| TCGA-FR-A728-01 | IFN-gamma Dominant (Immune C2) |
| TCGA-SB-A76C-01 | IFN-gamma Dominant (Immune C2) |
| TCGA-BG-A0YV-01 | IFN-gamma Dominant (Immune C2) |
| TCGA-OR-A5K4-01 | Lymphocyte Depleted (Immune C4) |
| TCGA-85-8352-01 | Wound Healing (Immune C1) |
| TCGA-77-8139-01 | Wound Healing (Immune C1) |
| TCGA-HU-A4H2-01 | Wound Healing (Immune C1) |
| TCGA-CR-7368-01 | IFN-gamma Dominant (Immune C2) |
| TCGA-D8-A1JH-01 | Inflammatory (Immune C3) |
| TCGA-AG-A032-01 | Wound Healing (Immune C1) |
| TCGA-LK-A4NW-01 | IFN-gamma Dominant (Immune C2) |
| TCGA-JV-A5VE-01 | IFN-gamma Dominant (Immune C2) |
| TCGA-05-5429-01 | Wound Healing (Immune C1) |
| TCGA-QS-A744-01 | Wound Healing (Immune C1) |
| TCGA-BJ-A3EZ-01 | Inflammatory (Immune C3) |
| TCGA-IG-A625-01 | Wound Healing (Immune C1) |
| TCGA-FT-A61P-01 | Wound Healing (Immune C1) |
| TCGA-VS-A9V1-01 | Wound Healing (Immune C1) |
| TCGA-EY-A2ON-01 | IFN-gamma Dominant (Immune C2) |
| TCGA-EJ-7781-01 | Inflammatory (Immune C3) |
| TCGA-QR-A70I-01 | Inflammatory (Immune C3) |
| TCGA-28-5215-01 | Lymphocyte Depleted (Immune C4) |
| TCGA-B9-7268-01 | Inflammatory (Immune C3) |
| TCGA-CQ-A4C6-01 | IFN-gamma Dominant (Immune C2) |
| TCGA-BB-4223-01 | IFN-gamma Dominant (Immune C2) |
| TCGA-19-2620-01 | Lymphocyte Depleted (Immune C4) |
| TCGA-BP-4999-01 | Inflammatory (Immune C3) |
| TCGA-EA-A78R-01 | IFN-gamma Dominant (Immune C2) |
| TCGA-EL-A3T8-01 | Inflammatory (Immune C3) |
| TCGA-FI-A2EW-01 | Wound Healing (Immune C1) |
| TCGA-S9-A6U5-01 | Lymphocyte Depleted (Immune C4) |
| TCGA-XY-A8S2-01 | Wound Healing (Immune C1) |
| TCGA-E1-A7YO-01 | Immunologically Quiet (Immune C5) |
| TCGA-F5-6813-01 | Wound Healing (Immune C1) |
| TCGA-DK-A3IQ-01 | Wound Healing (Immune C1) |
| TCGA-JW-A5VG-01 | IFN-gamma Dominant (Immune C2) |
| TCGA-CV-6941-01 | IFN-gamma Dominant (Immune C2) |
| TCGA-E9-A3Q9-01 | Inflammatory (Immune C3) |
| TCGA-BH-A0DL-01 | IFN-gamma Dominant (Immune C2) |
| TCGA-QH-A6X8-01 | Immunologically Quiet (Immune C5) |
| TCGA-DJ-A2Q7-01 | Inflammatory (Immune C3) |
| TCGA-UZ-A9PJ-01 | Inflammatory (Immune C3) |
| TCGA-99-7458-01 | Inflammatory (Immune C3) |
| TCGA-97-7546-01 | Inflammatory (Immune C3) |
| TCGA-M8-A5N4-01 | Wound Healing (Immune C1) |
| TCGA-AN-A0AL-01 | IFN-gamma Dominant (Immune C2) |
| TCGA-UY-A78N-01 | Wound Healing (Immune C1) |
| TCGA-EM-A3ST-01 | Inflammatory (Immune C3) |
| TCGA-DX-A8BK-01 | IFN-gamma Dominant (Immune C2) |
| TCGA-67-3772-01 | IFN-gamma Dominant (Immune C2) |
| TCGA-04-1514-01 | IFN-gamma Dominant (Immune C2) |
| TCGA-3B-A9HI-01 | Wound Healing (Immune C1) |
| TCGA-B6-A0WW-01 | IFN-gamma Dominant (Immune C2) |
| TCGA-HE-7128-01 | Inflammatory (Immune C3) |
| TCGA-CN-A63V-01 | Wound Healing (Immune C1) |
| TCGA-HC-A631-01 | Lymphocyte Depleted (Immune C4) |
| TCGA-OL-A5RZ-01 | Wound Healing (Immune C1) |
| TCGA-HT-7610-01 | Immunologically Quiet (Immune C5) |
| TCGA-CR-7397-01 | Wound Healing (Immune C1) |
| TCGA-85-7710-01 | Wound Healing (Immune C1) |
| TCGA-D8-A1JL-01 | IFN-gamma Dominant (Immune C2) |
| TCGA-FI-A2EX-01 | Wound Healing (Immune C1) |
| TCGA-F9-A97G-01 | Inflammatory (Immune C3) |
| TCGA-05-4395-01 | IFN-gamma Dominant (Immune C2) |
| TCGA-HT-7875-01 | Immunologically Quiet (Immune C5) |
| TCGA-A7-A0DB-01 | TGF-beta Dominant (Immune C6) |
| TCGA-B0-4690-01 | TGF-beta Dominant (Immune C6) |
| TCGA-DX-AB35-01 | Wound Healing (Immune C1) |
| TCGA-CN-6023-01 | IFN-gamma Dominant (Immune C2) |
| TCGA-S8-A6BV-01 | Inflammatory (Immune C3) |
| TCGA-AK-3440-01 | Immunologically Quiet (Immune C5) |
| TCGA-22-4595-01 | Wound Healing (Immune C1) |
| TCGA-AZ-4684-01 | Wound Healing (Immune C1) |
| TCGA-BP-4795-01 | Inflammatory (Immune C3) |
| TCGA-19-2624-01 | Lymphocyte Depleted (Immune C4) |
| TCGA-AX-A3GB-01 | Inflammatory (Immune C3) |
| TCGA-DU-7304-01 | Immunologically Quiet (Immune C5) |
| TCGA-AR-A1AV-01 | Lymphocyte Depleted (Immune C4) |
| TCGA-KK-A8ID-01 | Inflammatory (Immune C3) |
| TCGA-BS-A0U8-01 | Wound Healing (Immune C1) |
| TCGA-KL-8330-01 | Immunologically Quiet (Immune C5) |
| TCGA-A6-6652-01 | Wound Healing (Immune C1) |
| TCGA-C5-A8YT-01 | Wound Healing (Immune C1) |
| TCGA-CV-6953-01 | Wound Healing (Immune C1) |
| TCGA-4Z-AA7Y-01 | Wound Healing (Immune C1) |
| TCGA-FV-A496-01 | Inflammatory (Immune C3) |
| TCGA-2Z-A9JE-01 | Lymphocyte Depleted (Immune C4) |
| TCGA-EY-A1GU-01 | Inflammatory (Immune C3) |
| TCGA-CV-A45R-01 | IFN-gamma Dominant (Immune C2) |
| TCGA-D8-A1JC-01 | IFN-gamma Dominant (Immune C2) |
| TCGA-BF-A3DM-01 | IFN-gamma Dominant (Immune C2) |
| TCGA-ZX-AA5X-01 | IFN-gamma Dominant (Immune C2) |
| TCGA-5M-AAT6-01 | IFN-gamma Dominant (Immune C2) |
| TCGA-B0-4842-01 | Inflammatory (Immune C3) |
| TCGA-83-5908-01 | IFN-gamma Dominant (Immune C2) |
| TCGA-EW-A1OW-01 | IFN-gamma Dominant (Immune C2) |
| TCGA-DX-AB2P-01 | Wound Healing (Immune C1) |
| TCGA-DD-AAEH-01 | Inflammatory (Immune C3) |
| TCGA-02-0047-01 | Lymphocyte Depleted (Immune C4) |
| TCGA-B6-A2IU-01 | Inflammatory (Immune C3) |
| TCGA-2A-A8VT-01 | Lymphocyte Depleted (Immune C4) |
| TCGA-XK-AAIR-01 | Inflammatory (Immune C3) |
| TCGA-91-7771-01 | Inflammatory (Immune C3) |
| TCGA-C8-A137-01 | Wound Healing (Immune C1) |
| TCGA-DJ-A2PR-01 | Inflammatory (Immune C3) |
| TCGA-IG-A4P3-01 | IFN-gamma Dominant (Immune C2) |
| TCGA-B0-5107-01 | IFN-gamma Dominant (Immune C2) |
| TCGA-AG-A020-01 | Wound Healing (Immune C1) |
| TCGA-29-2414-01 | Wound Healing (Immune C1) |
| TCGA-55-6984-01 | Wound Healing (Immune C1) |
| TCGA-24-1843-01 | IFN-gamma Dominant (Immune C2) |
| TCGA-C8-A1HN-01 | Lymphocyte Depleted (Immune C4) |
| TCGA-AA-3522-01 | IFN-gamma Dominant (Immune C2) |
| TCGA-DX-AB3B-01 | Inflammatory (Immune C3) |
| TCGA-ET-A39K-01 | Inflammatory (Immune C3) |
| TCGA-S9-A6TS-01 | Immunologically Quiet (Immune C5) |
| TCGA-S9-A6TW-01 | Inflammatory (Immune C3) |
| TCGA-XC-AA0X-01 | Wound Healing (Immune C1) |
| TCGA-BR-8369-01 | Wound Healing (Immune C1) |
| TCGA-QR-A700-01 | Lymphocyte Depleted (Immune C4) |
| TCGA-G2-A2EK-01 | Inflammatory (Immune C3) |
| TCGA-CD-A489-01 | Wound Healing (Immune C1) |
| TCGA-W5-AA39-01 | Lymphocyte Depleted (Immune C4) |
| TCGA-E2-A1L8-01 | Wound Healing (Immune C1) |
| TCGA-IB-AAUU-01 | Wound Healing (Immune C1) |
| TCGA-E1-5303-01 | Immunologically Quiet (Immune C5) |
| TCGA-FD-A3SO-01 | Wound Healing (Immune C1) |
| TCGA-G4-6302-01 | Wound Healing (Immune C1) |
| TCGA-V1-A9Z9-01 | Inflammatory (Immune C3) |
| TCGA-GC-A3WC-01 | IFN-gamma Dominant (Immune C2) |
| TCGA-XE-A8H4-01 | IFN-gamma Dominant (Immune C2) |
| TCGA-50-8459-01 | Inflammatory (Immune C3) |
| TCGA-CZ-4866-01 | Inflammatory (Immune C3) |
| TCGA-DX-A8BJ-01 | IFN-gamma Dominant (Immune C2) |
| TCGA-2Z-A9JR-01 | Inflammatory (Immune C3) |
| TCGA-ZM-AA06-01 | IFN-gamma Dominant (Immune C2) |
| TCGA-DD-A3A3-01 | Lymphocyte Depleted (Immune C4) |
| TCGA-A7-A26J-01 | IFN-gamma Dominant (Immune C2) |
| TCGA-D1-A16V-01 | IFN-gamma Dominant (Immune C2) |
| TCGA-YL-A8SR-01 | Inflammatory (Immune C3) |
| TCGA-98-8023-01 | Wound Healing (Immune C1) |
| TCGA-BH-A0E7-01 | Inflammatory (Immune C3) |
| TCGA-39-5029-01 | IFN-gamma Dominant (Immune C2) |
| TCGA-VD-A8KN-01 | Inflammatory (Immune C3) |
| TCGA-EY-A547-01 | Wound Healing (Immune C1) |
| TCGA-RB-A7B8-01 | Wound Healing (Immune C1) |
| TCGA-TT-A6YP-01 | Inflammatory (Immune C3) |
| TCGA-CV-A6JO-01 | IFN-gamma Dominant (Immune C2) |
| TCGA-VS-A958-01 | IFN-gamma Dominant (Immune C2) |
| TCGA-BR-A4QI-01 | IFN-gamma Dominant (Immune C2) |
| TCGA-25-1870-01 | Lymphocyte Depleted (Immune C4) |
| TCGA-AG-3582-01 | Wound Healing (Immune C1) |
| TCGA-IM-A420-01 | Inflammatory (Immune C3) |
| TCGA-69-7980-01 | IFN-gamma Dominant (Immune C2) |
| TCGA-77-7465-01 | IFN-gamma Dominant (Immune C2) |
| TCGA-WC-A882-01 | Inflammatory (Immune C3) |
| TCGA-DC-6683-01 | Wound Healing (Immune C1) |
| TCGA-HT-7606-01 | Lymphocyte Depleted (Immune C4) |
| TCGA-DJ-A2PV-01 | Inflammatory (Immune C3) |
| TCGA-4Z-AA7M-01 | Wound Healing (Immune C1) |
| TCGA-BL-A3JM-01 | IFN-gamma Dominant (Immune C2) |
| TCGA-ZF-AA4U-01 | Wound Healing (Immune C1) |
| TCGA-ZF-AA53-01 | IFN-gamma Dominant (Immune C2) |
| TCGA-CF-A9FF-01 | IFN-gamma Dominant (Immune C2) |
| TCGA-77-8131-01 | IFN-gamma Dominant (Immune C2) |
| TCGA-DJ-A3VK-01 | Inflammatory (Immune C3) |
| TCGA-AJ-A2QO-01 | Wound Healing (Immune C1) |
| TCGA-VQ-A8DU-01 | IFN-gamma Dominant (Immune C2) |
| TCGA-R5-A7ZE-01 | Wound Healing (Immune C1) |
| TCGA-BJ-A45I-01 | IFN-gamma Dominant (Immune C2) |
| TCGA-AJ-A3NG-01 | Wound Healing (Immune C1) |
| TCGA-R6-A6L4-01 | Lymphocyte Depleted (Immune C4) |
| TCGA-D8-A73U-01 | Inflammatory (Immune C3) |
| TCGA-EL-A4KD-01 | Inflammatory (Immune C3) |
| TCGA-D8-A27N-01 | IFN-gamma Dominant (Immune C2) |
| TCGA-E2-A14Y-01 | IFN-gamma Dominant (Immune C2) |
| TCGA-24-2262-01 | Wound Healing (Immune C1) |
| TCGA-G7-A8LE-01 | Inflammatory (Immune C3) |
| TCGA-AA-A01G-01 | Wound Healing (Immune C1) |
| TCGA-LL-A8F5-01 | IFN-gamma Dominant (Immune C2) |
| TCGA-DQ-7595-01 | IFN-gamma Dominant (Immune C2) |
| TCGA-22-5472-01 | TGF-beta Dominant (Immune C6) |
| TCGA-E2-A14Z-01 | IFN-gamma Dominant (Immune C2) |
| TCGA-XJ-A9DK-01 | Inflammatory (Immune C3) |
| TCGA-L5-A88Z-01 | Wound Healing (Immune C1) |
| TCGA-DK-A3IV-01 | IFN-gamma Dominant (Immune C2) |
| TCGA-BP-4174-01 | Inflammatory (Immune C3) |
| TCGA-CV-5973-01 | IFN-gamma Dominant (Immune C2) |
| TCGA-KS-A4I7-01 | Inflammatory (Immune C3) |
| TCGA-FJ-A3Z9-01 | Lymphocyte Depleted (Immune C4) |
| TCGA-C8-A8HP-01 | Wound Healing (Immune C1) |
| TCGA-DU-6408-01 | Immunologically Quiet (Immune C5) |
| TCGA-2G-AALR-01 | Wound Healing (Immune C1) |
| TCGA-CN-5358-01 | Wound Healing (Immune C1) |
| TCGA-BH-A6R8-01 | IFN-gamma Dominant (Immune C2) |
| TCGA-AK-3426-01 | Inflammatory (Immune C3) |
| TCGA-FR-A3R1-01 | IFN-gamma Dominant (Immune C2) |
| TCGA-BH-A202-01 | Wound Healing (Immune C1) |
| TCGA-EB-A430-01 | Wound Healing (Immune C1) |
| TCGA-XF-AAMT-01 | IFN-gamma Dominant (Immune C2) |
| TCGA-FG-5964-01 | Immunologically Quiet (Immune C5) |
| TCGA-XF-AAN4-01 | IFN-gamma Dominant (Immune C2) |
| TCGA-77-8150-01 | Wound Healing (Immune C1) |
| TCGA-DQ-7588-01 | Wound Healing (Immune C1) |
| TCGA-13-1498-01 | IFN-gamma Dominant (Immune C2) |
| TCGA-CE-A13K-01 | Inflammatory (Immune C3) |
| TCGA-50-5939-01 | Wound Healing (Immune C1) |
| TCGA-OL-A5RY-01 | Wound Healing (Immune C1) |
| TCGA-SB-A6J6-01 | IFN-gamma Dominant (Immune C2) |
| TCGA-78-7143-01 | IFN-gamma Dominant (Immune C2) |
| TCGA-EM-A1CT-01 | Inflammatory (Immune C3) |
| TCGA-CR-7390-01 | Wound Healing (Immune C1) |
| TCGA-AX-A1C9-01 | IFN-gamma Dominant (Immune C2) |
| TCGA-DB-A64Q-01 | Immunologically Quiet (Immune C5) |
| TCGA-56-5898-01 | Wound Healing (Immune C1) |
| TCGA-BT-A0S7-01 | Wound Healing (Immune C1) |
| TCGA-S9-A7IZ-01 | Immunologically Quiet (Immune C5) |
| TCGA-CN-5364-01 | IFN-gamma Dominant (Immune C2) |
| TCGA-S9-A6WH-01 | Immunologically Quiet (Immune C5) |
| TCGA-75-5126-01 | Lymphocyte Depleted (Immune C4) |
| TCGA-HT-7602-01 | Immunologically Quiet (Immune C5) |
| TCGA-OL-A66I-01 | IFN-gamma Dominant (Immune C2) |
| TCGA-VS-A8EL-01 | IFN-gamma Dominant (Immune C2) |
| TCGA-HT-A74O-01 | Lymphocyte Depleted (Immune C4) |
| TCGA-BP-4346-01 | Inflammatory (Immune C3) |
| TCGA-YL-A8HL-01 | Inflammatory (Immune C3) |
| TCGA-FD-A5C1-01 | IFN-gamma Dominant (Immune C2) |
| TCGA-FE-A233-01 | Inflammatory (Immune C3) |
| TCGA-DX-A1KX-01 | Wound Healing (Immune C1) |
| TCGA-HT-7858-01 | Lymphocyte Depleted (Immune C4) |
| TCGA-CV-7438-01 | IFN-gamma Dominant (Immune C2) |
| TCGA-EY-A214-01 | IFN-gamma Dominant (Immune C2) |
| TCGA-AP-A0LT-01 | Wound Healing (Immune C1) |
| TCGA-2G-AAFV-01 | Wound Healing (Immune C1) |
| TCGA-CQ-6222-01 | IFN-gamma Dominant (Immune C2) |
| TCGA-C8-A12V-01 | IFN-gamma Dominant (Immune C2) |
| TCGA-46-3765-01 | IFN-gamma Dominant (Immune C2) |
| TCGA-32-1982-01 | Lymphocyte Depleted (Immune C4) |
| TCGA-DB-A64U-01 | Immunologically Quiet (Immune C5) |
| TCGA-D1-A1O5-01 | Wound Healing (Immune C1) |
| TCGA-E9-A1NF-01 | Wound Healing (Immune C1) |
| TCGA-S9-A6WG-01 | Lymphocyte Depleted (Immune C4) |
| TCGA-AO-A03N-01 | Wound Healing (Immune C1) |
| TCGA-MP-A5C7-01 | Inflammatory (Immune C3) |
| TCGA-OR-A5LT-01 | Lymphocyte Depleted (Immune C4) |
| TCGA-B0-5094-01 | Inflammatory (Immune C3) |
| TCGA-DX-A48N-01 | Lymphocyte Depleted (Immune C4) |
| TCGA-MH-A855-01 | Inflammatory (Immune C3) |
| TCGA-ZP-A9D4-01 | Lymphocyte Depleted (Immune C4) |
| TCGA-AA-A01I-01 | Wound Healing (Immune C1) |
| TCGA-44-5645-01 | Inflammatory (Immune C3) |
| TCGA-BH-A0HO-01 | Lymphocyte Depleted (Immune C4) |
| TCGA-CV-A45U-01 | IFN-gamma Dominant (Immune C2) |
| TCGA-WB-A81G-01 | Inflammatory (Immune C3) |
| TCGA-24-2267-01 | IFN-gamma Dominant (Immune C2) |
| TCGA-NH-A6GA-01 | Wound Healing (Immune C1) |
| TCGA-DK-A1A5-01 | Wound Healing (Immune C1) |
| TCGA-AR-A1AN-01 | Wound Healing (Immune C1) |
| TCGA-04-1362-01 | Lymphocyte Depleted (Immune C4) |
| TCGA-VF-A8AB-01 | IFN-gamma Dominant (Immune C2) |
| TCGA-HC-8260-01 | Inflammatory (Immune C3) |
| TCGA-MT-A51X-01 | IFN-gamma Dominant (Immune C2) |
| TCGA-A8-A09X-01 | IFN-gamma Dominant (Immune C2) |
| TCGA-HT-7860-01 | Lymphocyte Depleted (Immune C4) |
| TCGA-HC-8266-01 | Inflammatory (Immune C3) |
| TCGA-AA-3852-01 | Wound Healing (Immune C1) |
| TCGA-AR-A254-01 | TGF-beta Dominant (Immune C6) |
| TCGA-EJ-8472-01 | Inflammatory (Immune C3) |
| TCGA-AA-3655-01 | IFN-gamma Dominant (Immune C2) |
| TCGA-DU-7006-01 | Lymphocyte Depleted (Immune C4) |
| TCGA-50-5935-01 | Inflammatory (Immune C3) |
| TCGA-NA-A5I1-01 | Wound Healing (Immune C1) |
| TCGA-55-7914-01 | Inflammatory (Immune C3) |
| TCGA-ZJ-AB0I-01 | IFN-gamma Dominant (Immune C2) |
| TCGA-C5-A2LZ-01 | IFN-gamma Dominant (Immune C2) |
| TCGA-XF-A8HC-01 | Lymphocyte Depleted (Immune C4) |
| TCGA-DX-A7ES-01 | Inflammatory (Immune C3) |
| TCGA-BH-A0HL-01 | Lymphocyte Depleted (Immune C4) |
| TCGA-DX-A6BF-01 | Lymphocyte Depleted (Immune C4) |
| TCGA-HU-A4GF-01 | IFN-gamma Dominant (Immune C2) |
| TCGA-SX-A71S-01 | Inflammatory (Immune C3) |
| TCGA-39-5034-01 | Wound Healing (Immune C1) |
| TCGA-VQ-A91X-01 | Wound Healing (Immune C1) |
| TCGA-ZQ-A9CR-01 | Wound Healing (Immune C1) |
| TCGA-DS-A0VM-01 | IFN-gamma Dominant (Immune C2) |
| TCGA-KN-8437-01 | Immunologically Quiet (Immune C5) |
| TCGA-CQ-7065-01 | IFN-gamma Dominant (Immune C2) |
| TCGA-CM-5863-01 | Wound Healing (Immune C1) |
| TCGA-IB-7645-01 | Inflammatory (Immune C3) |
| TCGA-QK-AA3K-01 | IFN-gamma Dominant (Immune C2) |
| TCGA-VS-A950-01 | Wound Healing (Immune C1) |
| TCGA-AC-A3HN-01 | Inflammatory (Immune C3) |
| TCGA-A3-A6NJ-01 | Inflammatory (Immune C3) |
| TCGA-55-7576-01 | IFN-gamma Dominant (Immune C2) |
| TCGA-IR-A3LK-01 | IFN-gamma Dominant (Immune C2) |
| TCGA-BH-A0H7-01 | IFN-gamma Dominant (Immune C2) |
| TCGA-ED-A7XO-01 | IFN-gamma Dominant (Immune C2) |
| TCGA-DC-6155-01 | Wound Healing (Immune C1) |
| TCGA-DU-7009-01 | Immunologically Quiet (Immune C5) |
| TCGA-GL-A4EM-01 | Inflammatory (Immune C3) |
| TCGA-AC-A6IW-01 | IFN-gamma Dominant (Immune C2) |
| TCGA-EW-A1J5-01 | IFN-gamma Dominant (Immune C2) |
| TCGA-DD-A4NE-01 | Lymphocyte Depleted (Immune C4) |
| TCGA-DX-A48J-01 | Inflammatory (Immune C3) |
| TCGA-EY-A1GT-01 | Wound Healing (Immune C1) |
| TCGA-ZP-A9D0-01 | Inflammatory (Immune C3) |
| TCGA-CN-A498-01 | IFN-gamma Dominant (Immune C2) |
| TCGA-KK-A6E5-01 | Inflammatory (Immune C3) |
| TCGA-JY-A6FD-01 | IFN-gamma Dominant (Immune C2) |
| TCGA-44-2666-01 | IFN-gamma Dominant (Immune C2) |
| TCGA-XE-AANV-01 | IFN-gamma Dominant (Immune C2) |
| TCGA-BF-A1PU-01 | Wound Healing (Immune C1) |
| TCGA-55-7284-01 | Inflammatory (Immune C3) |
| TCGA-A5-A0GR-01 | Inflammatory (Immune C3) |
| TCGA-EO-A3AV-01 | IFN-gamma Dominant (Immune C2) |
| TCGA-DJ-A3UO-01 | Lymphocyte Depleted (Immune C4) |
| TCGA-DD-A73G-01 | Lymphocyte Depleted (Immune C4) |
| TCGA-B6-A1KN-01 | IFN-gamma Dominant (Immune C2) |
| TCGA-DU-7010-01 | Lymphocyte Depleted (Immune C4) |
| TCGA-BG-A0M4-01 | Inflammatory (Immune C3) |
| TCGA-73-4676-01 | Lymphocyte Depleted (Immune C4) |
| TCGA-WC-A880-01 | Lymphocyte Depleted (Immune C4) |
| TCGA-CM-6678-01 | Wound Healing (Immune C1) |
| TCGA-VD-AA8N-01 | Inflammatory (Immune C3) |
| TCGA-C5-A3HE-01 | IFN-gamma Dominant (Immune C2) |
| TCGA-QH-A6X3-01 | Immunologically Quiet (Immune C5) |
| TCGA-CR-7386-01 | IFN-gamma Dominant (Immune C2) |
| TCGA-SC-A6LQ-01 | Wound Healing (Immune C1) |
| TCGA-D1-A169-01 | Inflammatory (Immune C3) |
| TCGA-BG-A0M9-01 | Wound Healing (Immune C1) |
| TCGA-V4-A9E7-01 | Lymphocyte Depleted (Immune C4) |
| TCGA-DU-8164-01 | Immunologically Quiet (Immune C5) |
| TCGA-OL-A6VO-01 | IFN-gamma Dominant (Immune C2) |
| TCGA-AC-A23E-01 | Lymphocyte Depleted (Immune C4) |
| TCGA-RD-A8MW-01 | Wound Healing (Immune C1) |
| TCGA-DX-A2J4-01 | Inflammatory (Immune C3) |
| TCGA-AG-A01J-01 | Wound Healing (Immune C1) |
| TCGA-CV-6936-01 | IFN-gamma Dominant (Immune C2) |
| TCGA-D5-6529-01 | Wound Healing (Immune C1) |
| TCGA-78-7166-01 | Wound Healing (Immune C1) |
| TCGA-LK-A4O5-01 | TGF-beta Dominant (Immune C6) |
| TCGA-FD-A3N5-01 | IFN-gamma Dominant (Immune C2) |
| TCGA-D5-6922-01 | Wound Healing (Immune C1) |
| TCGA-P5-A5F0-01 | Immunologically Quiet (Immune C5) |
| TCGA-AJ-A3NH-01 | IFN-gamma Dominant (Immune C2) |
| TCGA-MA-AA3W-01 | IFN-gamma Dominant (Immune C2) |
| TCGA-50-5049-01 | IFN-gamma Dominant (Immune C2) |
| TCGA-G9-6370-01 | Inflammatory (Immune C3) |
| TCGA-B8-5164-01 | Inflammatory (Immune C3) |
| TCGA-GV-A40G-01 | Wound Healing (Immune C1) |
| TCGA-CC-A9FV-01 | Inflammatory (Immune C3) |
| TCGA-55-8506-01 | TGF-beta Dominant (Immune C6) |
| TCGA-CN-6012-01 | IFN-gamma Dominant (Immune C2) |
| TCGA-58-A46K-01 | IFN-gamma Dominant (Immune C2) |
| TCGA-A8-A09W-01 | IFN-gamma Dominant (Immune C2) |
| TCGA-AX-A2IN-01 | Wound Healing (Immune C1) |
| TCGA-B6-A0IM-01 | Wound Healing (Immune C1) |
| TCGA-44-3396-01 | IFN-gamma Dominant (Immune C2) |
| TCGA-HU-A4GU-01 | IFN-gamma Dominant (Immune C2) |
| TCGA-BP-5010-01 | Inflammatory (Immune C3) |
| TCGA-61-1919-01 | IFN-gamma Dominant (Immune C2) |
| TCGA-97-8172-01 | Inflammatory (Immune C3) |
| TCGA-64-5774-01 | Wound Healing (Immune C1) |
| TCGA-CN-A642-01 | IFN-gamma Dominant (Immune C2) |
| TCGA-N5-A59E-01 | Wound Healing (Immune C1) |
| TCGA-EM-A22I-01 | Inflammatory (Immune C3) |
| TCGA-DU-7014-01 | Immunologically Quiet (Immune C5) |
| TCGA-AP-A0LE-01 | Wound Healing (Immune C1) |
| TCGA-BR-4366-01 | Lymphocyte Depleted (Immune C4) |
| TCGA-AA-A01D-01 | Wound Healing (Immune C1) |
| TCGA-CN-6996-01 | IFN-gamma Dominant (Immune C2) |
| TCGA-BH-A1F0-01 | IFN-gamma Dominant (Immune C2) |
| TCGA-WK-A8XY-01 | Lymphocyte Depleted (Immune C4) |
| TCGA-HT-7693-01 | Immunologically Quiet (Immune C5) |
| TCGA-AC-A5EH-01 | Wound Healing (Immune C1) |
| TCGA-F4-6459-01 | Wound Healing (Immune C1) |
| TCGA-4Z-AA86-01 | IFN-gamma Dominant (Immune C2) |
| TCGA-UF-A7JK-01 | Wound Healing (Immune C1) |
| TCGA-AF-2692-01 | Wound Healing (Immune C1) |
| TCGA-BP-4161-01 | Inflammatory (Immune C3) |
| TCGA-EM-A22J-01 | Inflammatory (Immune C3) |
| TCGA-NG-A4VU-01 | Lymphocyte Depleted (Immune C4) |
| TCGA-85-8481-01 | IFN-gamma Dominant (Immune C2) |
| TCGA-B0-5098-01 | Wound Healing (Immune C1) |
| TCGA-MK-A84Z-01 | Inflammatory (Immune C3) |
| TCGA-D1-A0ZZ-01 | Wound Healing (Immune C1) |
| TCGA-T9-A92H-01 | Wound Healing (Immune C1) |
| TCGA-SH-A9CT-01 | Wound Healing (Immune C1) |
| TCGA-50-6597-01 | IFN-gamma Dominant (Immune C2) |
| TCGA-69-8255-01 | IFN-gamma Dominant (Immune C2) |
| TCGA-DD-AACY-01 | Lymphocyte Depleted (Immune C4) |
| TCGA-DD-AADW-01 | Wound Healing (Immune C1) |
| TCGA-EW-A1PD-01 | Wound Healing (Immune C1) |
| TCGA-A5-A3LO-01 | IFN-gamma Dominant (Immune C2) |
| TCGA-CV-A45Y-01 | IFN-gamma Dominant (Immune C2) |
| TCGA-D8-A1J8-01 | IFN-gamma Dominant (Immune C2) |
| TCGA-KM-8443-01 | Immunologically Quiet (Immune C5) |
| TCGA-23-1122-01 | IFN-gamma Dominant (Immune C2) |
| TCGA-3A-A9IZ-01 | Wound Healing (Immune C1) |
| TCGA-37-4141-01 | IFN-gamma Dominant (Immune C2) |
| TCGA-62-A46U-01 | Inflammatory (Immune C3) |
| TCGA-AG-3896-01 | Wound Healing (Immune C1) |
| TCGA-FY-A40M-01 | Inflammatory (Immune C3) |
| TCGA-CN-A641-01 | Wound Healing (Immune C1) |
| TCGA-AR-A24N-01 | IFN-gamma Dominant (Immune C2) |
| TCGA-A2-A0CQ-01 | Lymphocyte Depleted (Immune C4) |
| TCGA-DX-A6Z0-01 | Wound Healing (Immune C1) |
| TCGA-A4-A48D-01 | Inflammatory (Immune C3) |
| TCGA-3B-A9HY-01 | IFN-gamma Dominant (Immune C2) |
| TCGA-FY-A3I5-01 | Inflammatory (Immune C3) |
| TCGA-G9-6366-01 | Inflammatory (Immune C3) |
| TCGA-FB-AAQ2-01 | Wound Healing (Immune C1) |
| TCGA-FV-A3I1-01 | Inflammatory (Immune C3) |
| TCGA-AQ-A1H3-01 | Wound Healing (Immune C1) |
| TCGA-2G-AAFN-01 | IFN-gamma Dominant (Immune C2) |
| TCGA-BP-4771-01 | Inflammatory (Immune C3) |
| TCGA-BQ-7044-01 | Inflammatory (Immune C3) |
| TCGA-J8-A3YG-01 | Inflammatory (Immune C3) |
| TCGA-28-5220-01 | Lymphocyte Depleted (Immune C4) |
| TCGA-57-1584-01 | Lymphocyte Depleted (Immune C4) |
| TCGA-CC-A3M9-01 | IFN-gamma Dominant (Immune C2) |
| TCGA-25-1623-01 | IFN-gamma Dominant (Immune C2) |
| TCGA-NF-A4WU-01 | Wound Healing (Immune C1) |
| TCGA-58-8388-01 | Wound Healing (Immune C1) |
| TCGA-IB-A7LX-01 | Wound Healing (Immune C1) |
| TCGA-DD-A1EH-01 | Inflammatory (Immune C3) |
| TCGA-97-8176-01 | Wound Healing (Immune C1) |
| TCGA-C8-A131-01 | Wound Healing (Immune C1) |
| TCGA-K4-A6FZ-01 | IFN-gamma Dominant (Immune C2) |
| TCGA-ET-A3DR-01 | Inflammatory (Immune C3) |
| TCGA-ET-A25I-01 | Inflammatory (Immune C3) |
| TCGA-BR-6566-01 | IFN-gamma Dominant (Immune C2) |
| TCGA-EL-A3MY-01 | Inflammatory (Immune C3) |
| TCGA-EO-A22R-01 | Wound Healing (Immune C1) |
| TCGA-AA-A01S-01 | Wound Healing (Immune C1) |
| TCGA-B8-A54H-01 | Inflammatory (Immune C3) |
| TCGA-A2-A0YE-01 | IFN-gamma Dominant (Immune C2) |
| TCGA-A6-5657-01 | IFN-gamma Dominant (Immune C2) |
| TCGA-BT-A2LB-01 | IFN-gamma Dominant (Immune C2) |
| TCGA-GV-A3QF-01 | Wound Healing (Immune C1) |
| TCGA-AX-A1C5-01 | Wound Healing (Immune C1) |
| TCGA-DX-A8BU-01 | Wound Healing (Immune C1) |
| TCGA-G3-A25Z-01 | Lymphocyte Depleted (Immune C4) |
| TCGA-86-8358-01 | Wound Healing (Immune C1) |
| TCGA-V4-A9F3-01 | Inflammatory (Immune C3) |
| TCGA-29-1691-01 | IFN-gamma Dominant (Immune C2) |
| TCGA-DX-A6YT-01 | Wound Healing (Immune C1) |
| TCGA-D5-6929-01 | Wound Healing (Immune C1) |
| TCGA-D1-A16Q-01 | Wound Healing (Immune C1) |
| TCGA-3K-AAZ8-01 | Lymphocyte Depleted (Immune C4) |
| TCGA-E7-A7DV-01 | Lymphocyte Depleted (Immune C4) |
| TCGA-GC-A3I6-01 | IFN-gamma Dominant (Immune C2) |
| TCGA-LN-A9FQ-01 | Wound Healing (Immune C1) |
| TCGA-5P-A9K9-01 | Lymphocyte Depleted (Immune C4) |
| TCGA-V4-A9EL-01 | Lymphocyte Depleted (Immune C4) |
| TCGA-CC-A8HU-01 | Lymphocyte Depleted (Immune C4) |
| TCGA-05-4415-01 | IFN-gamma Dominant (Immune C2) |
| TCGA-B6-A40C-01 | Wound Healing (Immune C1) |
| TCGA-2Y-A9H0-01 | IFN-gamma Dominant (Immune C2) |
| TCGA-HT-7472-01 | Immunologically Quiet (Immune C5) |
| TCGA-D5-6930-01 | Wound Healing (Immune C1) |
| TCGA-BJ-A2N7-01 | Inflammatory (Immune C3) |
| TCGA-BS-A0V7-01 | TGF-beta Dominant (Immune C6) |
| TCGA-CJ-4908-01 | Inflammatory (Immune C3) |
| TCGA-VS-A9UL-01 | Wound Healing (Immune C1) |
| TCGA-C4-A0F7-01 | Wound Healing (Immune C1) |
| TCGA-BP-4158-01 | Inflammatory (Immune C3) |
| TCGA-HT-7854-01 | Immunologically Quiet (Immune C5) |
| TCGA-DD-AADD-01 | Lymphocyte Depleted (Immune C4) |
| TCGA-SR-A6MZ-01 | Lymphocyte Depleted (Immune C4) |
| TCGA-C5-A905-01 | Wound Healing (Immune C1) |
| TCGA-S4-A8RP-01 | Inflammatory (Immune C3) |
| TCGA-EW-A6SC-01 | Wound Healing (Immune C1) |
| TCGA-BQ-7055-01 | Inflammatory (Immune C3) |
| TCGA-BR-6452-01 | Wound Healing (Immune C1) |
| TCGA-R5-A7ZF-01 | IFN-gamma Dominant (Immune C2) |
| TCGA-CV-A464-01 | IFN-gamma Dominant (Immune C2) |
| TCGA-D1-A161-01 | Wound Healing (Immune C1) |
| TCGA-IA-A40U-01 | Inflammatory (Immune C3) |
| TCGA-BP-5176-01 | Inflammatory (Immune C3) |
| TCGA-YL-A9WJ-01 | Inflammatory (Immune C3) |
| TCGA-EO-A3AU-01 | IFN-gamma Dominant (Immune C2) |
| TCGA-DD-A4NF-01 | Lymphocyte Depleted (Immune C4) |
| TCGA-06-5408-01 | Lymphocyte Depleted (Immune C4) |
| TCGA-25-1632-01 | Lymphocyte Depleted (Immune C4) |
| TCGA-CD-8524-01 | IFN-gamma Dominant (Immune C2) |
| TCGA-EJ-5519-01 | Inflammatory (Immune C3) |
| TCGA-DD-A1EL-01 | Lymphocyte Depleted (Immune C4) |
| TCGA-DK-A6AW-01 | Lymphocyte Depleted (Immune C4) |
| TCGA-EI-6885-01 | Wound Healing (Immune C1) |
| TCGA-BR-A4J5-01 | Wound Healing (Immune C1) |
| TCGA-18-3407-01 | IFN-gamma Dominant (Immune C2) |
| TCGA-FD-A62O-01 | Wound Healing (Immune C1) |
| TCGA-VS-A9UO-01 | Lymphocyte Depleted (Immune C4) |
| TCGA-BR-6802-01 | IFN-gamma Dominant (Immune C2) |
| TCGA-DB-A4XG-01 | Immunologically Quiet (Immune C5) |
| TCGA-DT-5265-01 | IFN-gamma Dominant (Immune C2) |
| TCGA-24-1544-01 | IFN-gamma Dominant (Immune C2) |
| TCGA-CJ-4907-01 | Inflammatory (Immune C3) |
| TCGA-A4-7584-01 | Lymphocyte Depleted (Immune C4) |
| TCGA-WB-A80O-01 | Inflammatory (Immune C3) |
| TCGA-G7-A8LB-01 | Inflammatory (Immune C3) |
| TCGA-C5-A8XJ-01 | IFN-gamma Dominant (Immune C2) |
| TCGA-QL-A97D-01 | Wound Healing (Immune C1) |
| TCGA-E2-A1IF-01 | Wound Healing (Immune C1) |
| TCGA-HT-A5RC-01 | Lymphocyte Depleted (Immune C4) |
| TCGA-C5-A7CL-01 | IFN-gamma Dominant (Immune C2) |
| TCGA-AO-A0JE-01 | IFN-gamma Dominant (Immune C2) |
| TCGA-S3-AA11-01 | Lymphocyte Depleted (Immune C4) |
| TCGA-JY-A93D-01 | TGF-beta Dominant (Immune C6) |
| TCGA-CJ-4912-01 | Inflammatory (Immune C3) |
| TCGA-ER-A19T-01 | Wound Healing (Immune C1) |
| TCGA-FU-A3NI-01 | IFN-gamma Dominant (Immune C2) |
| TCGA-EA-A439-01 | IFN-gamma Dominant (Immune C2) |
| TCGA-D5-7000-01 | Wound Healing (Immune C1) |
| TCGA-23-1111-01 | IFN-gamma Dominant (Immune C2) |
| TCGA-EO-A22Y-01 | Wound Healing (Immune C1) |
| TCGA-49-4507-01 | IFN-gamma Dominant (Immune C2) |
| TCGA-HF-7133-01 | IFN-gamma Dominant (Immune C2) |
| TCGA-3X-AAVE-01 | IFN-gamma Dominant (Immune C2) |
| TCGA-AP-A1DQ-01 | IFN-gamma Dominant (Immune C2) |
| TCGA-AA-3877-01 | IFN-gamma Dominant (Immune C2) |
| TCGA-KR-A7K0-01 | Lymphocyte Depleted (Immune C4) |
| TCGA-FE-A230-01 | Inflammatory (Immune C3) |
| TCGA-VM-A8C8-01 | Immunologically Quiet (Immune C5) |
| TCGA-56-8504-01 | IFN-gamma Dominant (Immune C2) |
| TCGA-KN-8426-01 | Lymphocyte Depleted (Immune C4) |
| TCGA-55-8621-01 | Inflammatory (Immune C3) |
| TCGA-95-7948-01 | Wound Healing (Immune C1) |
| TCGA-B8-5163-01 | IFN-gamma Dominant (Immune C2) |
| TCGA-D8-A27I-01 | Inflammatory (Immune C3) |
| TCGA-CD-8536-01 | Wound Healing (Immune C1) |
| TCGA-BP-4760-01 | Inflammatory (Immune C3) |
| TCGA-2G-AALP-01 | Wound Healing (Immune C1) |
| TCGA-V4-A9EX-01 | Lymphocyte Depleted (Immune C4) |
| TCGA-HT-7681-01 | Lymphocyte Depleted (Immune C4) |
| TCGA-2G-AAGM-01 | Wound Healing (Immune C1) |
| TCGA-52-7622-01 | IFN-gamma Dominant (Immune C2) |
| TCGA-2G-AALN-01 | Wound Healing (Immune C1) |
| TCGA-LN-A7HX-01 | Wound Healing (Immune C1) |
| TCGA-A3-3317-01 | Inflammatory (Immune C3) |
| TCGA-BR-4201-01 | IFN-gamma Dominant (Immune C2) |
| TCGA-G3-A5SM-01 | Inflammatory (Immune C3) |
| TCGA-63-A5MI-01 | Wound Healing (Immune C1) |
| TCGA-KM-8476-01 | Inflammatory (Immune C3) |
| TCGA-AA-3866-01 | IFN-gamma Dominant (Immune C2) |
| TCGA-B5-A11F-01 | Wound Healing (Immune C1) |
| TCGA-BJ-A3PT-01 | Inflammatory (Immune C3) |
| TCGA-HC-A4ZV-01 | Inflammatory (Immune C3) |
| TCGA-UD-AABZ-01 | Wound Healing (Immune C1) |
| TCGA-20-1685-01 | Lymphocyte Depleted (Immune C4) |
| TCGA-BQ-7062-01 | Inflammatory (Immune C3) |
| TCGA-CV-7248-01 | Wound Healing (Immune C1) |
| TCGA-D6-6823-01 | IFN-gamma Dominant (Immune C2) |
| TCGA-13-0913-01 | IFN-gamma Dominant (Immune C2) |
| TCGA-FE-A3PA-01 | Inflammatory (Immune C3) |
| TCGA-BR-7723-01 | Wound Healing (Immune C1) |
| TCGA-DX-A3UB-01 | Inflammatory (Immune C3) |
| TCGA-AC-A3W6-01 | Wound Healing (Immune C1) |
| TCGA-H7-7774-01 | IFN-gamma Dominant (Immune C2) |
| TCGA-BH-A0AZ-01 | Wound Healing (Immune C1) |
| TCGA-CG-5720-01 | Wound Healing (Immune C1) |
| TCGA-BF-AAP4-01 | IFN-gamma Dominant (Immune C2) |
| TCGA-A2-A0D3-01 | Inflammatory (Immune C3) |
| TCGA-55-6970-01 | Inflammatory (Immune C3) |
| TCGA-AR-A0TX-01 | Inflammatory (Immune C3) |
| TCGA-B0-5119-01 | Inflammatory (Immune C3) |
| TCGA-CJ-4916-01 | Inflammatory (Immune C3) |
| TCGA-G9-6385-01 | Inflammatory (Immune C3) |
| TCGA-DD-A11C-01 | Inflammatory (Immune C3) |
| TCGA-75-6207-01 | IFN-gamma Dominant (Immune C2) |
| TCGA-LT-A5Z6-01 | Wound Healing (Immune C1) |
| TCGA-CN-6997-01 | Wound Healing (Immune C1) |
| TCGA-2L-AAQJ-01 | Inflammatory (Immune C3) |
| TCGA-B5-A11J-01 | IFN-gamma Dominant (Immune C2) |
| TCGA-A8-A07L-01 | Wound Healing (Immune C1) |
| TCGA-B0-4696-01 | Lymphocyte Depleted (Immune C4) |
| TCGA-AP-A05N-01 | Wound Healing (Immune C1) |
| TCGA-MF-A522-01 | Wound Healing (Immune C1) |
| TCGA-V4-A9EO-01 | Lymphocyte Depleted (Immune C4) |
| TCGA-A2-A0SV-01 | IFN-gamma Dominant (Immune C2) |
| TCGA-BH-A1FC-01 | IFN-gamma Dominant (Immune C2) |
| TCGA-CM-5860-01 | Wound Healing (Immune C1) |
| TCGA-E2-A1B5-01 | IFN-gamma Dominant (Immune C2) |
| TCGA-CZ-5989-01 | Lymphocyte Depleted (Immune C4) |
| TCGA-2G-AAM3-01 | Wound Healing (Immune C1) |
| TCGA-C5-A7CG-01 | IFN-gamma Dominant (Immune C2) |
| TCGA-VS-A94X-01 | IFN-gamma Dominant (Immune C2) |
| TCGA-DU-A6S2-01 | Immunologically Quiet (Immune C5) |
| TCGA-FD-A3B8-01 | IFN-gamma Dominant (Immune C2) |
| TCGA-CD-8532-01 | IFN-gamma Dominant (Immune C2) |
| TCGA-59-A5PD-01 | Wound Healing (Immune C1) |
| TCGA-EJ-7788-01 | Inflammatory (Immune C3) |
| TCGA-BF-AAP8-01 | Inflammatory (Immune C3) |
| TCGA-BB-4225-01 | IFN-gamma Dominant (Immune C2) |
| TCGA-A7-A5ZW-01 | Wound Healing (Immune C1) |
| TCGA-DK-AA6R-01 | IFN-gamma Dominant (Immune C2) |
| TCGA-AA-A01P-01 | IFN-gamma Dominant (Immune C2) |
| TCGA-MX-A5UJ-01 | IFN-gamma Dominant (Immune C2) |
| TCGA-S9-A6WD-01 | Immunologically Quiet (Immune C5) |
| TCGA-AN-A0FT-01 | Wound Healing (Immune C1) |
| TCGA-VD-A8KI-01 | Lymphocyte Depleted (Immune C4) |
| TCGA-A2-A1G6-01 | Inflammatory (Immune C3) |
| TCGA-DU-7309-01 | Immunologically Quiet (Immune C5) |
| TCGA-BR-8289-01 | Wound Healing (Immune C1) |
| TCGA-L5-A4OT-01 | Wound Healing (Immune C1) |
| TCGA-EU-5906-01 | Inflammatory (Immune C3) |
| TCGA-D6-6827-01 | Wound Healing (Immune C1) |
| TCGA-AA-3542-01 | Wound Healing (Immune C1) |
| TCGA-DD-A73D-01 | Lymphocyte Depleted (Immune C4) |
| TCGA-BA-A4IF-01 | IFN-gamma Dominant (Immune C2) |
| TCGA-IM-A4EB-01 | Inflammatory (Immune C3) |
| TCGA-C5-A1BI-01 | IFN-gamma Dominant (Immune C2) |
| TCGA-UZ-A9PL-01 | Inflammatory (Immune C3) |
| TCGA-CW-5580-01 | Inflammatory (Immune C3) |
| TCGA-CN-5370-01 | IFN-gamma Dominant (Immune C2) |
| TCGA-66-2753-01 | Wound Healing (Immune C1) |
| TCGA-BR-8485-01 | Wound Healing (Immune C1) |
| TCGA-CG-5724-01 | IFN-gamma Dominant (Immune C2) |
| TCGA-VD-AA8M-01 | Inflammatory (Immune C3) |
| TCGA-DJ-A13V-01 | Inflammatory (Immune C3) |
| TCGA-DD-A4NB-01 | Inflammatory (Immune C3) |
| TCGA-A2-A0YF-01 | Lymphocyte Depleted (Immune C4) |
| TCGA-HU-A4GY-01 | IFN-gamma Dominant (Immune C2) |
| TCGA-B0-5690-01 | Inflammatory (Immune C3) |
| TCGA-CK-4950-01 | Wound Healing (Immune C1) |
| TCGA-HM-A3JJ-01 | IFN-gamma Dominant (Immune C2) |
| TCGA-J8-A3O1-01 | Inflammatory (Immune C3) |
| TCGA-KN-8429-01 | Inflammatory (Immune C3) |
| TCGA-98-A53B-01 | Wound Healing (Immune C1) |
| TCGA-86-8056-01 | Inflammatory (Immune C3) |
| TCGA-55-8205-01 | IFN-gamma Dominant (Immune C2) |
| TCGA-BC-A10Z-01 | Lymphocyte Depleted (Immune C4) |
| TCGA-EM-A1YE-01 | Inflammatory (Immune C3) |
| TCGA-EM-A1CV-01 | Inflammatory (Immune C3) |
| TCGA-AL-3473-01 | IFN-gamma Dominant (Immune C2) |
| TCGA-AX-A3FV-01 | Wound Healing (Immune C1) |
| TCGA-BJ-A0ZB-01 | Inflammatory (Immune C3) |
| TCGA-DZ-6134-01 | Inflammatory (Immune C3) |
| TCGA-FD-A6TA-01 | Wound Healing (Immune C1) |
| TCGA-HD-8314-01 | IFN-gamma Dominant (Immune C2) |
| TCGA-25-1635-01 | IFN-gamma Dominant (Immune C2) |
| TCGA-G3-A25V-01 | Inflammatory (Immune C3) |
| TCGA-63-A5MR-01 | IFN-gamma Dominant (Immune C2) |
| TCGA-69-7763-01 | IFN-gamma Dominant (Immune C2) |
| TCGA-ZJ-AAXD-01 | Wound Healing (Immune C1) |
| TCGA-SS-A7HO-01 | Wound Healing (Immune C1) |
| TCGA-66-2763-01 | IFN-gamma Dominant (Immune C2) |
| TCGA-BH-A0BR-01 | Wound Healing (Immune C1) |
| TCGA-Z7-A8R6-01 | Wound Healing (Immune C1) |
| TCGA-EK-A2R8-01 | IFN-gamma Dominant (Immune C2) |
| TCGA-AX-A2IO-01 | Wound Healing (Immune C1) |
| TCGA-2L-AAQA-01 | Wound Healing (Immune C1) |
| TCGA-WC-A87T-01 | Lymphocyte Depleted (Immune C4) |
| TCGA-ND-A4W6-01 | Wound Healing (Immune C1) |
| TCGA-94-A5I6-01 | Wound Healing (Immune C1) |
| TCGA-TS-A7P0-01 | Wound Healing (Immune C1) |
| TCGA-VD-A8KM-01 | Inflammatory (Immune C3) |
| TCGA-D5-6537-01 | IFN-gamma Dominant (Immune C2) |
| TCGA-62-8397-01 | Inflammatory (Immune C3) |
| TCGA-WQ-A9G7-01 | Wound Healing (Immune C1) |
| TCGA-OL-A5D8-01 | IFN-gamma Dominant (Immune C2) |
| TCGA-AA-3549-01 | Wound Healing (Immune C1) |
| TCGA-DB-A4XH-01 | Immunologically Quiet (Immune C5) |
| TCGA-HT-7485-01 | Immunologically Quiet (Immune C5) |
| TCGA-23-1023-01 | IFN-gamma Dominant (Immune C2) |
| TCGA-VW-A7QS-01 | Lymphocyte Depleted (Immune C4) |
| TCGA-30-1891-01 | Wound Healing (Immune C1) |
| TCGA-CW-5584-01 | Inflammatory (Immune C3) |
| TCGA-66-2742-01 | Wound Healing (Immune C1) |
| TCGA-B6-A0IJ-01 | IFN-gamma Dominant (Immune C2) |
| TCGA-FJ-A3ZE-01 | Lymphocyte Depleted (Immune C4) |
| TCGA-EJ-7783-01 | Inflammatory (Immune C3) |
| TCGA-04-1348-01 | IFN-gamma Dominant (Immune C2) |
| TCGA-06-0211-01 | Lymphocyte Depleted (Immune C4) |
| TCGA-A3-3376-01 | Inflammatory (Immune C3) |
| TCGA-AA-3664-01 | IFN-gamma Dominant (Immune C2) |
| TCGA-VV-A86M-01 | Immunologically Quiet (Immune C5) |
| TCGA-D7-6522-01 | Inflammatory (Immune C3) |
| TCGA-73-4659-01 | Lymphocyte Depleted (Immune C4) |
| TCGA-CZ-5986-01 | Inflammatory (Immune C3) |
| TCGA-2L-AAQE-01 | IFN-gamma Dominant (Immune C2) |
| TCGA-CG-4449-01 | Wound Healing (Immune C1) |
| TCGA-VS-A9UP-01 | Wound Healing (Immune C1) |
| TCGA-BH-A0AY-01 | Wound Healing (Immune C1) |
| TCGA-A6-5665-01 | Wound Healing (Immune C1) |
| TCGA-BS-A0TJ-01 | Wound Healing (Immune C1) |
| TCGA-ET-A3BN-01 | Inflammatory (Immune C3) |
| TCGA-E1-A7YV-01 | Lymphocyte Depleted (Immune C4) |
| TCGA-AK-3454-01 | Inflammatory (Immune C3) |
| TCGA-PE-A5DD-01 | Wound Healing (Immune C1) |
| TCGA-CA-5254-01 | Wound Healing (Immune C1) |
| TCGA-TQ-A7RW-01 | Immunologically Quiet (Immune C5) |
| TCGA-A2-A1FW-01 | Lymphocyte Depleted (Immune C4) |
| TCGA-E2-A576-01 | Wound Healing (Immune C1) |
| TCGA-HT-7468-01 | Immunologically Quiet (Immune C5) |
| TCGA-AP-A1E0-01 | Wound Healing (Immune C1) |
| TCGA-ZF-A9RM-01 | Lymphocyte Depleted (Immune C4) |
| TCGA-56-8304-01 | Wound Healing (Immune C1) |
| TCGA-G7-A8LD-01 | Inflammatory (Immune C3) |
| TCGA-AD-A5EK-01 | Wound Healing (Immune C1) |
| TCGA-37-4130-01 | IFN-gamma Dominant (Immune C2) |
| TCGA-91-A4BC-01 | IFN-gamma Dominant (Immune C2) |
| TCGA-O8-A75V-01 | Inflammatory (Immune C3) |
| TCGA-CK-6746-01 | IFN-gamma Dominant (Immune C2) |
| TCGA-CJ-5681-01 | Lymphocyte Depleted (Immune C4) |
| TCGA-AK-3456-01 | Inflammatory (Immune C3) |
| TCGA-FP-7829-01 | Wound Healing (Immune C1) |
| TCGA-CA-6718-01 | IFN-gamma Dominant (Immune C2) |
| TCGA-HT-7677-01 | Immunologically Quiet (Immune C5) |
| TCGA-AJ-A3TW-01 | IFN-gamma Dominant (Immune C2) |
| TCGA-YS-AA4M-01 | IFN-gamma Dominant (Immune C2) |
| TCGA-UY-A78L-01 | Wound Healing (Immune C1) |
| TCGA-VQ-A8E2-01 | Wound Healing (Immune C1) |
| TCGA-W5-AA2G-01 | Inflammatory (Immune C3) |
| TCGA-DE-A3KN-01 | Inflammatory (Immune C3) |
| TCGA-A7-A13H-01 | Inflammatory (Immune C3) |
| TCGA-50-8457-01 | Inflammatory (Immune C3) |
| TCGA-A4-A7UZ-01 | Inflammatory (Immune C3) |
| TCGA-B0-5121-01 | Inflammatory (Immune C3) |
| TCGA-04-1519-01 | Lymphocyte Depleted (Immune C4) |
| TCGA-C5-A1BF-01 | Wound Healing (Immune C1) |
| TCGA-E2-A1IO-01 | Inflammatory (Immune C3) |
| TCGA-CR-6474-01 | Wound Healing (Immune C1) |
| TCGA-13-0801-01 | IFN-gamma Dominant (Immune C2) |
| TCGA-AY-5543-01 | IFN-gamma Dominant (Immune C2) |
| TCGA-EJ-5504-01 | Inflammatory (Immune C3) |
| TCGA-97-8547-01 | TGF-beta Dominant (Immune C6) |
| TCGA-K4-A4AB-01 | Wound Healing (Immune C1) |
| TCGA-AN-A049-01 | IFN-gamma Dominant (Immune C2) |
| TCGA-WT-AB44-01 | Wound Healing (Immune C1) |
| TCGA-97-8175-01 | IFN-gamma Dominant (Immune C2) |
| TCGA-BR-8297-01 | IFN-gamma Dominant (Immune C2) |
| TCGA-CG-5732-01 | IFN-gamma Dominant (Immune C2) |
| TCGA-76-4931-01 | Lymphocyte Depleted (Immune C4) |
| TCGA-BH-A0BF-01 | IFN-gamma Dominant (Immune C2) |
| TCGA-AR-A5QN-01 | IFN-gamma Dominant (Immune C2) |
| TCGA-DK-A2I1-01 | Wound Healing (Immune C1) |
| TCGA-DU-A5TY-01 | Lymphocyte Depleted (Immune C4) |
| TCGA-HT-8109-01 | Immunologically Quiet (Immune C5) |
| TCGA-QT-A7U0-01 | Inflammatory (Immune C3) |
| TCGA-FS-A1ZN-01 | Wound Healing (Immune C1) |
| TCGA-27-2519-01 | Lymphocyte Depleted (Immune C4) |
| TCGA-2G-AAEX-01 | IFN-gamma Dominant (Immune C2) |
| TCGA-BJ-A45H-01 | Inflammatory (Immune C3) |
| TCGA-F4-6807-01 | Wound Healing (Immune C1) |
| TCGA-06-0156-01 | Lymphocyte Depleted (Immune C4) |
| TCGA-23-1809-01 | IFN-gamma Dominant (Immune C2) |
| TCGA-06-0686-01 | Lymphocyte Depleted (Immune C4) |
| TCGA-EM-A3FK-01 | Inflammatory (Immune C3) |
| TCGA-CV-6433-01 | IFN-gamma Dominant (Immune C2) |
| TCGA-NI-A8LF-01 | Inflammatory (Immune C3) |
| TCGA-AG-4001-01 | Wound Healing (Immune C1) |
| TCGA-AQ-A54N-01 | Lymphocyte Depleted (Immune C4) |
| TCGA-34-5239-01 | Wound Healing (Immune C1) |
| TCGA-AX-A0J0-01 | Wound Healing (Immune C1) |
| TCGA-S8-A6BW-01 | IFN-gamma Dominant (Immune C2) |
| TCGA-EJ-5512-01 | Inflammatory (Immune C3) |
| TCGA-EA-A5ZD-01 | IFN-gamma Dominant (Immune C2) |
| TCGA-CV-7410-01 | IFN-gamma Dominant (Immune C2) |
| TCGA-A2-A04V-01 | Wound Healing (Immune C1) |
| TCGA-EM-A1YB-01 | Inflammatory (Immune C3) |
| TCGA-66-2757-01 | IFN-gamma Dominant (Immune C2) |
| TCGA-DD-AADG-01 | Lymphocyte Depleted (Immune C4) |
| TCGA-CK-5915-01 | Wound Healing (Immune C1) |
| TCGA-85-A4QR-01 | Wound Healing (Immune C1) |
| TCGA-ET-A40R-01 | Inflammatory (Immune C3) |
| TCGA-E2-A14T-01 | Inflammatory (Immune C3) |
| TCGA-BF-A3DL-01 | Wound Healing (Immune C1) |
| TCGA-90-7767-01 | IFN-gamma Dominant (Immune C2) |
| TCGA-64-1676-01 | IFN-gamma Dominant (Immune C2) |
| TCGA-A6-3810-01 | Wound Healing (Immune C1) |
| TCGA-MP-A4TJ-01 | Inflammatory (Immune C3) |
| TCGA-VQ-A91A-01 | Wound Healing (Immune C1) |
| TCGA-24-1103-01 | IFN-gamma Dominant (Immune C2) |
| TCGA-E9-A295-01 | Wound Healing (Immune C1) |
| TCGA-2H-A9GN-01 | Wound Healing (Immune C1) |
| TCGA-B9-A69E-01 | Inflammatory (Immune C3) |
| TCGA-A3-3352-01 | Inflammatory (Immune C3) |
| TCGA-BB-A6UO-01 | IFN-gamma Dominant (Immune C2) |
| TCGA-86-8075-01 | Wound Healing (Immune C1) |
| TCGA-VT-A80G-01 | Wound Healing (Immune C1) |
| TCGA-HT-7479-01 | Immunologically Quiet (Immune C5) |
| TCGA-PR-A5PF-01 | Lymphocyte Depleted (Immune C4) |
| TCGA-FB-A4P6-01 | Inflammatory (Immune C3) |
| TCGA-BR-8486-01 | Wound Healing (Immune C1) |
| TCGA-S6-A8JX-01 | Wound Healing (Immune C1) |
| TCGA-B8-5159-01 | Inflammatory (Immune C3) |
| TCGA-DJ-A4UR-01 | Inflammatory (Immune C3) |
| TCGA-XF-AAMZ-01 | Wound Healing (Immune C1) |
| TCGA-55-6985-01 | TGF-beta Dominant (Immune C6) |
| TCGA-UF-A7JF-01 | Wound Healing (Immune C1) |
| TCGA-SP-A6QD-01 | Inflammatory (Immune C3) |
| TCGA-CC-A9FS-01 | Inflammatory (Immune C3) |
| TCGA-EW-A1P4-01 | Wound Healing (Immune C1) |
| TCGA-DJ-A2Q9-01 | Inflammatory (Immune C3) |
| TCGA-FD-A3SM-01 | Wound Healing (Immune C1) |
| TCGA-CS-6186-01 | Lymphocyte Depleted (Immune C4) |
| TCGA-EP-A2KB-01 | Inflammatory (Immune C3) |
| TCGA-NK-A5CX-01 | IFN-gamma Dominant (Immune C2) |
| TCGA-B4-5843-01 | Inflammatory (Immune C3) |
| TCGA-DX-A6YS-01 | Inflammatory (Immune C3) |
| TCGA-44-7659-01 | Inflammatory (Immune C3) |
| TCGA-JX-A3Q0-01 | IFN-gamma Dominant (Immune C2) |
| TCGA-43-6771-01 | Wound Healing (Immune C1) |
| TCGA-D1-A2G5-01 | Wound Healing (Immune C1) |
| TCGA-YL-A8SH-01 | Inflammatory (Immune C3) |
| TCGA-BJ-A0ZE-01 | Inflammatory (Immune C3) |
| TCGA-AG-A015-01 | Wound Healing (Immune C1) |
| TCGA-A2-A0T4-01 | IFN-gamma Dominant (Immune C2) |
| TCGA-BI-A0VS-01 | IFN-gamma Dominant (Immune C2) |
| TCGA-BH-A0W3-01 | Lymphocyte Depleted (Immune C4) |
| TCGA-CG-4306-01 | IFN-gamma Dominant (Immune C2) |
| TCGA-VS-A8EJ-01 | IFN-gamma Dominant (Immune C2) |
| TCGA-BH-A42U-01 | Inflammatory (Immune C3) |
| TCGA-PJ-A5Z9-01 | Inflammatory (Immune C3) |
| TCGA-DD-AAE1-01 | Lymphocyte Depleted (Immune C4) |
| TCGA-B8-A7U6-01 | Inflammatory (Immune C3) |
| TCGA-A8-A08G-01 | IFN-gamma Dominant (Immune C2) |
| TCGA-DF-A2KR-01 | IFN-gamma Dominant (Immune C2) |
| TCGA-DJ-A2PO-01 | Inflammatory (Immune C3) |
| TCGA-BH-A0BQ-01 | IFN-gamma Dominant (Immune C2) |
| TCGA-IG-A97I-01 | IFN-gamma Dominant (Immune C2) |
| TCGA-HZ-A49G-01 | Inflammatory (Immune C3) |
| TCGA-EW-A1IX-01 | Inflammatory (Immune C3) |
| TCGA-DB-5275-01 | Immunologically Quiet (Immune C5) |
| TCGA-AG-A008-01 | Wound Healing (Immune C1) |
| TCGA-MI-A75G-01 | Lymphocyte Depleted (Immune C4) |
| TCGA-DM-A28K-01 | Wound Healing (Immune C1) |
| TCGA-DX-A23Z-01 | Inflammatory (Immune C3) |
| TCGA-DR-A0ZL-01 | Wound Healing (Immune C1) |
| TCGA-JW-A69B-01 | IFN-gamma Dominant (Immune C2) |
| TCGA-A1-A0SH-01 | TGF-beta Dominant (Immune C6) |
| TCGA-YU-A90P-01 | IFN-gamma Dominant (Immune C2) |
| TCGA-TQ-A7RS-01 | Immunologically Quiet (Immune C5) |
| TCGA-AU-3779-01 | IFN-gamma Dominant (Immune C2) |
| TCGA-ET-A3DV-01 | Lymphocyte Depleted (Immune C4) |
| TCGA-5P-A9K0-01 | Inflammatory (Immune C3) |
| TCGA-QR-A6GR-01 | Inflammatory (Immune C3) |
| TCGA-YL-A9WX-01 | Inflammatory (Immune C3) |
| TCGA-E2-A10E-01 | IFN-gamma Dominant (Immune C2) |
| TCGA-SX-A71R-01 | Inflammatory (Immune C3) |
| TCGA-56-A49D-01 | IFN-gamma Dominant (Immune C2) |
| TCGA-LN-A8I0-01 | IFN-gamma Dominant (Immune C2) |
| TCGA-CC-A7IF-01 | Lymphocyte Depleted (Immune C4) |
| TCGA-TM-A7C4-01 | Immunologically Quiet (Immune C5) |
| TCGA-EM-A2CM-01 | Inflammatory (Immune C3) |
| TCGA-MT-A67D-01 | IFN-gamma Dominant (Immune C2) |
| TCGA-Z6-AAPN-01 | IFN-gamma Dominant (Immune C2) |
| TCGA-HZ-8003-01 | Inflammatory (Immune C3) |
| TCGA-3B-A9HL-01 | TGF-beta Dominant (Immune C6) |
| TCGA-B5-A3FB-01 | Wound Healing (Immune C1) |
| TCGA-E7-A85H-01 | Wound Healing (Immune C1) |
| TCGA-G4-6304-01 | Lymphocyte Depleted (Immune C4) |
| TCGA-CV-7424-01 | Wound Healing (Immune C1) |
| TCGA-A7-A0CG-01 | Inflammatory (Immune C3) |
| TCGA-HD-7229-01 | IFN-gamma Dominant (Immune C2) |
| TCGA-44-2657-01 | IFN-gamma Dominant (Immune C2) |
| TCGA-DX-AB2F-01 | Lymphocyte Depleted (Immune C4) |
| TCGA-95-7562-01 | Wound Healing (Immune C1) |
| TCGA-EL-A3D1-01 | Inflammatory (Immune C3) |
| TCGA-DD-A3A5-01 | Lymphocyte Depleted (Immune C4) |
| TCGA-55-7725-01 | Inflammatory (Immune C3) |
| TCGA-EM-A3AO-01 | Inflammatory (Immune C3) |
| TCGA-A2-A0CS-01 | Wound Healing (Immune C1) |
| TCGA-OR-A5L6-01 | Lymphocyte Depleted (Immune C4) |
| TCGA-A1-A0SG-01 | Inflammatory (Immune C3) |
| TCGA-GJ-A3OU-01 | Inflammatory (Immune C3) |
| TCGA-CQ-6219-01 | IFN-gamma Dominant (Immune C2) |
| TCGA-XF-A9T2-01 | Wound Healing (Immune C1) |
| TCGA-MA-AA41-01 | IFN-gamma Dominant (Immune C2) |
| TCGA-KS-A4IB-01 | Inflammatory (Immune C3) |
| TCGA-LN-A5U5-01 | IFN-gamma Dominant (Immune C2) |
| TCGA-HC-7820-01 | Lymphocyte Depleted (Immune C4) |
| TCGA-BC-A5W4-01 | Lymphocyte Depleted (Immune C4) |
| TCGA-22-5474-01 | IFN-gamma Dominant (Immune C2) |
| TCGA-D6-A4ZB-01 | IFN-gamma Dominant (Immune C2) |
| TCGA-A4-8517-01 | Inflammatory (Immune C3) |
| TCGA-LN-A49R-01 | IFN-gamma Dominant (Immune C2) |
| TCGA-CA-6717-01 | Wound Healing (Immune C1) |
| TCGA-DX-A1L1-01 | IFN-gamma Dominant (Immune C2) |
| TCGA-WX-AA47-01 | Lymphocyte Depleted (Immune C4) |
| TCGA-AJ-A5DV-01 | IFN-gamma Dominant (Immune C2) |
| TCGA-ZG-A9NI-01 | Inflammatory (Immune C3) |
| TCGA-DX-A3LU-01 | IFN-gamma Dominant (Immune C2) |
| TCGA-DU-A76K-01 | Lymphocyte Depleted (Immune C4) |
| TCGA-E7-A678-01 | Lymphocyte Depleted (Immune C4) |
| TCGA-69-A59K-01 | Inflammatory (Immune C3) |
| TCGA-EM-A3SZ-01 | Inflammatory (Immune C3) |
| TCGA-D1-A16D-01 | Wound Healing (Immune C1) |
| TCGA-E2-A1BD-01 | Wound Healing (Immune C1) |
| TCGA-CR-7371-01 | Wound Healing (Immune C1) |
| TCGA-2G-AAKO-01 | IFN-gamma Dominant (Immune C2) |
| TCGA-XE-AAOL-01 | IFN-gamma Dominant (Immune C2) |
| TCGA-UF-A718-01 | IFN-gamma Dominant (Immune C2) |
| TCGA-70-6723-01 | IFN-gamma Dominant (Immune C2) |
| TCGA-CV-7568-01 | IFN-gamma Dominant (Immune C2) |
| TCGA-56-5897-01 | Wound Healing (Immune C1) |
| TCGA-DV-A4VZ-01 | Inflammatory (Immune C3) |
| TCGA-V4-A9F2-01 | Inflammatory (Immune C3) |
| TCGA-20-1682-01 | Wound Healing (Immune C1) |
| TCGA-B8-5553-01 | Inflammatory (Immune C3) |
| TCGA-A2-A04R-01 | Wound Healing (Immune C1) |
| TCGA-CC-A7IJ-01 | IFN-gamma Dominant (Immune C2) |
| TCGA-EL-A3CY-01 | Inflammatory (Immune C3) |
| TCGA-55-A492-01 | Inflammatory (Immune C3) |
| TCGA-KO-8403-01 | Inflammatory (Immune C3) |
| TCGA-96-8170-01 | Wound Healing (Immune C1) |
| TCGA-A4-7286-01 | Inflammatory (Immune C3) |
| TCGA-18-3412-01 | Wound Healing (Immune C1) |
| TCGA-D1-A3DH-01 | IFN-gamma Dominant (Immune C2) |
| TCGA-DM-A1D0-01 | Wound Healing (Immune C1) |
| TCGA-MB-A5YA-01 | Lymphocyte Depleted (Immune C4) |
| TCGA-A7-A425-01 | Inflammatory (Immune C3) |
| TCGA-E9-A1RF-01 | IFN-gamma Dominant (Immune C2) |
| TCGA-B6-A408-01 | IFN-gamma Dominant (Immune C2) |
| TCGA-D7-6820-01 | Wound Healing (Immune C1) |
| TCGA-WB-A80M-01 | Lymphocyte Depleted (Immune C4) |
| TCGA-LP-A5U2-01 | IFN-gamma Dominant (Immune C2) |
| TCGA-DI-A1C3-01 | Inflammatory (Immune C3) |
| TCGA-UZ-A9PK-01 | Lymphocyte Depleted (Immune C4) |
| TCGA-AR-A1AL-01 | Inflammatory (Immune C3) |
| TCGA-3Z-A93Z-01 | Inflammatory (Immune C3) |
| TCGA-DH-A7UU-01 | Immunologically Quiet (Immune C5) |
| TCGA-AN-A0AM-01 | IFN-gamma Dominant (Immune C2) |
| TCGA-EY-A212-01 | IFN-gamma Dominant (Immune C2) |
| TCGA-CF-A9FH-01 | IFN-gamma Dominant (Immune C2) |
| TCGA-D8-A1JU-01 | Inflammatory (Immune C3) |
| TCGA-FG-8186-01 | Lymphocyte Depleted (Immune C4) |
| TCGA-CJ-4872-01 | Inflammatory (Immune C3) |
| TCGA-EQ-8122-01 | IFN-gamma Dominant (Immune C2) |
| TCGA-LN-A49V-01 | IFN-gamma Dominant (Immune C2) |
| TCGA-FG-A4MT-01 | Immunologically Quiet (Immune C5) |
| TCGA-G3-AAV2-01 | Lymphocyte Depleted (Immune C4) |
| TCGA-2Z-A9JL-01 | Inflammatory (Immune C3) |
| TCGA-DU-A76O-01 | Immunologically Quiet (Immune C5) |
| TCGA-63-A5MY-01 | Wound Healing (Immune C1) |
| TCGA-B5-A0JZ-01 | IFN-gamma Dominant (Immune C2) |
| TCGA-CV-7091-01 | IFN-gamma Dominant (Immune C2) |
| TCGA-BA-A6DE-01 | IFN-gamma Dominant (Immune C2) |
| TCGA-VF-A8AC-01 | IFN-gamma Dominant (Immune C2) |
| TCGA-FI-A2EY-01 | Wound Healing (Immune C1) |
| TCGA-IN-AB1V-01 | Inflammatory (Immune C3) |
| TCGA-B5-A5OE-01 | Wound Healing (Immune C1) |
| TCGA-AR-A1AH-01 | Wound Healing (Immune C1) |
| TCGA-EJ-7125-01 | Inflammatory (Immune C3) |
| TCGA-86-8278-01 | TGF-beta Dominant (Immune C6) |
| TCGA-YU-AA61-01 | IFN-gamma Dominant (Immune C2) |
| TCGA-BC-A3KF-01 | Inflammatory (Immune C3) |
| TCGA-EL-A3T3-01 | Inflammatory (Immune C3) |
| TCGA-69-8453-01 | Inflammatory (Immune C3) |
| TCGA-19-1787-01 | Lymphocyte Depleted (Immune C4) |
| TCGA-A4-A6HP-01 | Inflammatory (Immune C3) |
| TCGA-BR-4368-01 | IFN-gamma Dominant (Immune C2) |
| TCGA-T2-A6WX-01 | IFN-gamma Dominant (Immune C2) |
| TCGA-E1-5305-01 | Immunologically Quiet (Immune C5) |
| TCGA-34-7107-01 | Wound Healing (Immune C1) |
| TCGA-BJ-A290-01 | Inflammatory (Immune C3) |
| TCGA-C5-A3HD-01 | IFN-gamma Dominant (Immune C2) |
| TCGA-AA-3939-01 | Wound Healing (Immune C1) |
| TCGA-RW-A7D0-01 | Inflammatory (Immune C3) |
| TCGA-IR-A3LI-01 | Lymphocyte Depleted (Immune C4) |
| TCGA-BH-A0EB-01 | TGF-beta Dominant (Immune C6) |
| TCGA-63-A5M9-01 | Wound Healing (Immune C1) |
| TCGA-92-8063-01 | Wound Healing (Immune C1) |
| TCGA-NC-A5HR-01 | Wound Healing (Immune C1) |
| TCGA-VS-A8EI-01 | IFN-gamma Dominant (Immune C2) |
| TCGA-BH-A1EY-01 | Inflammatory (Immune C3) |
| TCGA-B4-5844-01 | Inflammatory (Immune C3) |
| TCGA-LN-A49Y-01 | Wound Healing (Immune C1) |
| TCGA-DX-A23V-01 | TGF-beta Dominant (Immune C6) |
| TCGA-25-2409-01 | IFN-gamma Dominant (Immune C2) |
| TCGA-OR-A5K5-01 | Lymphocyte Depleted (Immune C4) |
| TCGA-DD-AAC8-01 | Lymphocyte Depleted (Immune C4) |
| TCGA-EY-A3L3-01 | Wound Healing (Immune C1) |
| TCGA-AC-A6IV-01 | TGF-beta Dominant (Immune C6) |
| TCGA-D8-A1JB-01 | IFN-gamma Dominant (Immune C2) |
| TCGA-EB-A550-01 | IFN-gamma Dominant (Immune C2) |
| TCGA-FX-A3NK-01 | Wound Healing (Immune C1) |
| TCGA-FP-A9TM-01 | IFN-gamma Dominant (Immune C2) |
| TCGA-CG-4437-01 | IFN-gamma Dominant (Immune C2) |
| TCGA-D8-A27P-01 | Inflammatory (Immune C3) |
| TCGA-2G-AAF8-01 | IFN-gamma Dominant (Immune C2) |
| TCGA-VQ-A8PQ-01 | Inflammatory (Immune C3) |
| TCGA-E9-A5FL-01 | IFN-gamma Dominant (Immune C2) |
| TCGA-LL-A5YM-01 | Wound Healing (Immune C1) |
| TCGA-MN-A4N5-01 | IFN-gamma Dominant (Immune C2) |
| TCGA-BP-4763-01 | IFN-gamma Dominant (Immune C2) |
| TCGA-GL-6846-01 | Inflammatory (Immune C3) |
| TCGA-B8-5549-01 | Inflammatory (Immune C3) |
| TCGA-DI-A0WH-01 | Wound Healing (Immune C1) |
| TCGA-CM-6675-01 | IFN-gamma Dominant (Immune C2) |
| TCGA-BJ-A192-01 | Lymphocyte Depleted (Immune C4) |
| TCGA-DC-4745-01 | Wound Healing (Immune C1) |
| TCGA-CS-5393-01 | Immunologically Quiet (Immune C5) |
| TCGA-AF-A56K-01 | Wound Healing (Immune C1) |
| TCGA-CV-7440-01 | IFN-gamma Dominant (Immune C2) |
| TCGA-ET-A39T-01 | Inflammatory (Immune C3) |
| TCGA-KL-8324-01 | Lymphocyte Depleted (Immune C4) |
| TCGA-WC-A884-01 | Inflammatory (Immune C3) |
| TCGA-V5-AASX-01 | IFN-gamma Dominant (Immune C2) |
| TCGA-CJ-4644-01 | Inflammatory (Immune C3) |
| TCGA-XX-A89A-01 | Inflammatory (Immune C3) |
| TCGA-Z4-AAPG-01 | Lymphocyte Depleted (Immune C4) |
| TCGA-EM-A2CJ-01 | Inflammatory (Immune C3) |
| TCGA-CF-A47T-01 | Wound Healing (Immune C1) |
| TCGA-4Z-AA7W-01 | IFN-gamma Dominant (Immune C2) |
| TCGA-GM-A2DL-01 | Wound Healing (Immune C1) |
| TCGA-64-5778-01 | IFN-gamma Dominant (Immune C2) |
| TCGA-DE-A0XZ-01 | Inflammatory (Immune C3) |
| TCGA-AK-3436-01 | Inflammatory (Immune C3) |
| TCGA-AC-A23H-01 | Wound Healing (Immune C1) |
| TCGA-A7-A26H-01 | Wound Healing (Immune C1) |
| TCGA-EL-A3ZK-01 | Inflammatory (Immune C3) |
| TCGA-C4-A0F6-01 | Wound Healing (Immune C1) |
| TCGA-68-A59J-01 | IFN-gamma Dominant (Immune C2) |
| TCGA-86-8074-01 | IFN-gamma Dominant (Immune C2) |
| TCGA-F7-A50J-01 | IFN-gamma Dominant (Immune C2) |
| TCGA-55-A4DF-01 | IFN-gamma Dominant (Immune C2) |
| TCGA-VQ-A8PF-01 | IFN-gamma Dominant (Immune C2) |
| TCGA-4Z-AA7S-01 | Wound Healing (Immune C1) |
| TCGA-L5-A8NG-01 | IFN-gamma Dominant (Immune C2) |
| TCGA-DD-AADV-01 | Lymphocyte Depleted (Immune C4) |
| TCGA-66-2777-01 | Wound Healing (Immune C1) |
| TCGA-BS-A0TC-01 | Inflammatory (Immune C3) |
| TCGA-EA-A4BA-01 | Wound Healing (Immune C1) |
| TCGA-S9-A7IS-01 | Lymphocyte Depleted (Immune C4) |
| TCGA-J2-8194-01 | Inflammatory (Immune C3) |
| TCGA-2G-AALT-01 | IFN-gamma Dominant (Immune C2) |
| TCGA-2G-AAF4-01 | IFN-gamma Dominant (Immune C2) |
| TCGA-CV-6960-01 | Wound Healing (Immune C1) |
| TCGA-55-A57B-01 | TGF-beta Dominant (Immune C6) |
| TCGA-D1-A0ZO-01 | IFN-gamma Dominant (Immune C2) |
| TCGA-A6-4105-01 | Wound Healing (Immune C1) |
| TCGA-G9-6367-01 | Inflammatory (Immune C3) |
| TCGA-38-4627-01 | Wound Healing (Immune C1) |
| TCGA-BJ-A2P4-01 | Inflammatory (Immune C3) |
| TCGA-CC-5261-01 | Inflammatory (Immune C3) |
| TCGA-ZS-A9CG-01 | Inflammatory (Immune C3) |
| TCGA-VT-AB3D-01 | IFN-gamma Dominant (Immune C2) |
| TCGA-AA-A004-01 | Lymphocyte Depleted (Immune C4) |
| TCGA-DC-4749-01 | Wound Healing (Immune C1) |
| TCGA-IB-A5ST-01 | Inflammatory (Immune C3) |
| TCGA-UY-A78K-01 | IFN-gamma Dominant (Immune C2) |
| TCGA-77-8130-01 | IFN-gamma Dominant (Immune C2) |
| TCGA-AR-A24S-01 | IFN-gamma Dominant (Immune C2) |
| TCGA-KL-8339-01 | Lymphocyte Depleted (Immune C4) |
| TCGA-HT-7856-01 | Immunologically Quiet (Immune C5) |
| TCGA-E2-A15K-01 | Wound Healing (Immune C1) |
| TCGA-AJ-A3EL-01 | IFN-gamma Dominant (Immune C2) |
| TCGA-AG-4007-01 | Wound Healing (Immune C1) |
| TCGA-AO-A12G-01 | Wound Healing (Immune C1) |
| TCGA-2G-AAKG-01 | IFN-gamma Dominant (Immune C2) |
| TCGA-24-1603-01 | IFN-gamma Dominant (Immune C2) |
| TCGA-4Z-AA87-01 | Wound Healing (Immune C1) |
| TCGA-VD-AA8T-01 | Lymphocyte Depleted (Immune C4) |
| TCGA-HS-A5N8-01 | Lymphocyte Depleted (Immune C4) |
| TCGA-YL-A8SC-01 | Lymphocyte Depleted (Immune C4) |
| TCGA-23-1123-01 | IFN-gamma Dominant (Immune C2) |
| TCGA-AX-A3FZ-01 | Wound Healing (Immune C1) |
| TCGA-BH-A0HA-01 | IFN-gamma Dominant (Immune C2) |
| TCGA-S9-A7R7-01 | Lymphocyte Depleted (Immune C4) |
| TCGA-AP-A0LO-01 | Wound Healing (Immune C1) |
| TCGA-BH-A0DQ-01 | IFN-gamma Dominant (Immune C2) |
| TCGA-5P-A9JW-01 | Lymphocyte Depleted (Immune C4) |
| TCGA-ET-A39M-01 | Inflammatory (Immune C3) |
| TCGA-06-0750-01 | Lymphocyte Depleted (Immune C4) |
| TCGA-BG-A0LW-01 | Inflammatory (Immune C3) |
| TCGA-AX-A05Z-01 | IFN-gamma Dominant (Immune C2) |
| TCGA-61-2009-01 | Wound Healing (Immune C1) |
| TCGA-IN-AB1X-01 | IFN-gamma Dominant (Immune C2) |
| TCGA-BP-4770-01 | Wound Healing (Immune C1) |
| TCGA-85-8072-01 | Wound Healing (Immune C1) |
| TCGA-09-1670-01 | Lymphocyte Depleted (Immune C4) |
| TCGA-D1-A2G6-01 | Wound Healing (Immune C1) |
| TCGA-DK-A3IN-01 | Wound Healing (Immune C1) |
| TCGA-DD-AAD6-01 | Lymphocyte Depleted (Immune C4) |
| TCGA-B9-A5W9-01 | Lymphocyte Depleted (Immune C4) |
| TCGA-N7-A4Y5-01 | Wound Healing (Immune C1) |
| TCGA-CV-A45O-01 | Lymphocyte Depleted (Immune C4) |
| TCGA-F5-6571-01 | Inflammatory (Immune C3) |
| TCGA-77-7337-01 | Wound Healing (Immune C1) |
| TCGA-TS-A7PB-01 | IFN-gamma Dominant (Immune C2) |
| TCGA-G4-6293-01 | Wound Healing (Immune C1) |
| TCGA-05-5423-01 | IFN-gamma Dominant (Immune C2) |
| TCGA-BP-4961-01 | Inflammatory (Immune C3) |
| TCGA-56-8623-01 | Inflammatory (Immune C3) |
| TCGA-G3-A3CH-01 | Inflammatory (Immune C3) |
| TCGA-21-1071-01 | Wound Healing (Immune C1) |
| TCGA-J8-A3YF-01 | Inflammatory (Immune C3) |
| TCGA-LG-A6GG-01 | Inflammatory (Immune C3) |
| TCGA-VQ-A91Y-01 | IFN-gamma Dominant (Immune C2) |
| TCGA-IB-8126-01 | Inflammatory (Immune C3) |
| TCGA-CN-6989-01 | Wound Healing (Immune C1) |
| TCGA-ZF-A9R5-01 | Lymphocyte Depleted (Immune C4) |
| TCGA-B0-5102-01 | Inflammatory (Immune C3) |
| TCGA-E2-A153-01 | TGF-beta Dominant (Immune C6) |
| TCGA-WB-A81T-01 | Lymphocyte Depleted (Immune C4) |
| TCGA-32-5222-01 | Lymphocyte Depleted (Immune C4) |
| TCGA-E8-A3X7-01 | Inflammatory (Immune C3) |
| TCGA-AR-A24W-01 | Inflammatory (Immune C3) |
| TCGA-QT-A5XJ-01 | Inflammatory (Immune C3) |
| TCGA-D8-A1JJ-01 | Wound Healing (Immune C1) |
| TCGA-4G-AAZO-01 | Lymphocyte Depleted (Immune C4) |
| TCGA-QN-A5NN-01 | IFN-gamma Dominant (Immune C2) |
| TCGA-AZ-4313-01 | Wound Healing (Immune C1) |
| TCGA-DD-AADM-01 | Lymphocyte Depleted (Immune C4) |
| TCGA-2Z-A9J2-01 | Inflammatory (Immune C3) |
| TCGA-CC-5258-01 | Lymphocyte Depleted (Immune C4) |
| TCGA-DX-A48P-01 | Inflammatory (Immune C3) |
| TCGA-AR-A2LH-01 | IFN-gamma Dominant (Immune C2) |
| TCGA-ZA-A8F6-01 | Inflammatory (Immune C3) |
| TCGA-BK-A6W3-01 | IFN-gamma Dominant (Immune C2) |
| TCGA-EJ-5524-01 | Inflammatory (Immune C3) |
| TCGA-HC-7210-01 | Inflammatory (Immune C3) |
| TCGA-DJ-A1QO-01 | Inflammatory (Immune C3) |
| TCGA-28-5209-01 | Lymphocyte Depleted (Immune C4) |
| TCGA-C5-A3HL-01 | Wound Healing (Immune C1) |
| TCGA-CJ-4885-01 | Inflammatory (Immune C3) |
| TCGA-S9-A7R1-01 | Immunologically Quiet (Immune C5) |
| TCGA-AJ-A3NE-01 | IFN-gamma Dominant (Immune C2) |
| TCGA-91-8496-01 | Lymphocyte Depleted (Immune C4) |
| TCGA-BR-7851-01 | Wound Healing (Immune C1) |
| TCGA-24-2020-01 | Wound Healing (Immune C1) |
| TCGA-G4-6307-01 | Wound Healing (Immune C1) |
| TCGA-24-1413-01 | Lymphocyte Depleted (Immune C4) |
| TCGA-24-2254-01 | Lymphocyte Depleted (Immune C4) |
| TCGA-D6-A74Q-01 | IFN-gamma Dominant (Immune C2) |
| TCGA-ET-A3DP-01 | Inflammatory (Immune C3) |
| TCGA-E2-A158-01 | Wound Healing (Immune C1) |
| TCGA-AP-A0LV-01 | Wound Healing (Immune C1) |
| TCGA-B5-A0JN-01 | IFN-gamma Dominant (Immune C2) |
| TCGA-BR-4187-01 | IFN-gamma Dominant (Immune C2) |
| TCGA-B0-4712-01 | IFN-gamma Dominant (Immune C2) |
| TCGA-CJ-4868-01 | TGF-beta Dominant (Immune C6) |
| TCGA-A8-A079-01 | Wound Healing (Immune C1) |
| TCGA-EL-A4K2-01 | Inflammatory (Immune C3) |
| TCGA-A3-3307-01 | Inflammatory (Immune C3) |
| TCGA-86-8280-01 | Inflammatory (Immune C3) |
| TCGA-3M-AB47-01 | Wound Healing (Immune C1) |
| TCGA-EA-A3Y4-01 | IFN-gamma Dominant (Immune C2) |
| TCGA-BH-A204-01 | Lymphocyte Depleted (Immune C4) |
| TCGA-BH-A0B4-01 | Wound Healing (Immune C1) |
| TCGA-ZF-A9R1-01 | Wound Healing (Immune C1) |
| TCGA-CF-A47S-01 | Lymphocyte Depleted (Immune C4) |
| TCGA-61-2098-01 | Wound Healing (Immune C1) |
| TCGA-R5-A7O7-01 | Wound Healing (Immune C1) |
| TCGA-BA-5151-01 | IFN-gamma Dominant (Immune C2) |
| TCGA-FD-A62N-01 | IFN-gamma Dominant (Immune C2) |
| TCGA-CC-A8HS-01 | Inflammatory (Immune C3) |
| TCGA-24-2023-01 | IFN-gamma Dominant (Immune C2) |
| TCGA-B0-4815-01 | Inflammatory (Immune C3) |
| TCGA-AO-A03T-01 | IFN-gamma Dominant (Immune C2) |
| TCGA-IQ-A61G-01 | IFN-gamma Dominant (Immune C2) |
| TCGA-QR-A6H5-01 | Inflammatory (Immune C3) |
| TCGA-V1-A9O7-01 | Inflammatory (Immune C3) |
| TCGA-DI-A1NO-01 | Wound Healing (Immune C1) |
| TCGA-96-A4JL-01 | IFN-gamma Dominant (Immune C2) |
| TCGA-GM-A2DM-01 | Inflammatory (Immune C3) |
| TCGA-2Z-A9J6-01 | Inflammatory (Immune C3) |
| TCGA-FD-A3SJ-01 | Wound Healing (Immune C1) |
| TCGA-EM-A2CR-01 | Inflammatory (Immune C3) |
| TCGA-V4-A9EI-01 | Lymphocyte Depleted (Immune C4) |
| TCGA-DU-7008-01 | Immunologically Quiet (Immune C5) |
| TCGA-AX-A2HD-01 | Wound Healing (Immune C1) |
| TCGA-A2-A4RX-01 | Inflammatory (Immune C3) |
| TCGA-60-2698-01 | IFN-gamma Dominant (Immune C2) |
| TCGA-FE-A231-01 | Inflammatory (Immune C3) |
| TCGA-HT-7688-01 | Immunologically Quiet (Immune C5) |
| TCGA-G3-A25Y-01 | IFN-gamma Dominant (Immune C2) |
| TCGA-V3-A9ZX-01 | Inflammatory (Immune C3) |
| TCGA-EO-A3KX-01 | Wound Healing (Immune C1) |
| TCGA-2G-AALW-01 | Wound Healing (Immune C1) |
| TCGA-DU-5872-01 | Lymphocyte Depleted (Immune C4) |
| TCGA-J9-A8CL-01 | Inflammatory (Immune C3) |
| TCGA-24-0975-01 | Wound Healing (Immune C1) |
| TCGA-S3-A6ZH-01 | Wound Healing (Immune C1) |
| TCGA-D8-A27H-01 | Wound Healing (Immune C1) |
| TCGA-A6-6781-01 | Wound Healing (Immune C1) |
| TCGA-AP-A1DP-01 | Wound Healing (Immune C1) |
| TCGA-BR-8591-01 | IFN-gamma Dominant (Immune C2) |
| TCGA-HU-A4GP-01 | Wound Healing (Immune C1) |
| TCGA-E9-A1NC-01 | IFN-gamma Dominant (Immune C2) |
| TCGA-YC-A89H-01 | Wound Healing (Immune C1) |
| TCGA-L5-A88S-01 | Wound Healing (Immune C1) |
| TCGA-UC-A7PF-01 | IFN-gamma Dominant (Immune C2) |
| TCGA-62-8394-01 | IFN-gamma Dominant (Immune C2) |
| TCGA-B0-5096-01 | Inflammatory (Immune C3) |
| TCGA-VQ-AA6A-01 | Wound Healing (Immune C1) |
| TCGA-A2-A3XS-01 | IFN-gamma Dominant (Immune C2) |
| TCGA-44-6146-01 | Inflammatory (Immune C3) |
| TCGA-EL-A4K6-01 | Lymphocyte Depleted (Immune C4) |
| TCGA-A3-3316-01 | Inflammatory (Immune C3) |
| TCGA-DU-A6S3-01 | Immunologically Quiet (Immune C5) |
| TCGA-63-7023-01 | IFN-gamma Dominant (Immune C2) |
| TCGA-5C-AAPD-01 | IFN-gamma Dominant (Immune C2) |
| TCGA-A7-A26E-01 | Inflammatory (Immune C3) |
| TCGA-CF-A47X-01 | Lymphocyte Depleted (Immune C4) |
| TCGA-AK-3465-01 | Inflammatory (Immune C3) |
| TCGA-WB-A81N-01 | Lymphocyte Depleted (Immune C4) |
| TCGA-DD-A116-01 | Lymphocyte Depleted (Immune C4) |
| TCGA-BF-A1PX-01 | IFN-gamma Dominant (Immune C2) |
| TCGA-EM-A22L-01 | Inflammatory (Immune C3) |
| TCGA-AR-A0TZ-01 | Wound Healing (Immune C1) |
| TCGA-DJ-A1QD-01 | Inflammatory (Immune C3) |
| TCGA-DU-A76L-01 | Lymphocyte Depleted (Immune C4) |
| TCGA-L6-A4EQ-01 | Inflammatory (Immune C3) |
| TCGA-UZ-A9PX-01 | Inflammatory (Immune C3) |
| TCGA-AA-3994-01 | Wound Healing (Immune C1) |
| TCGA-AO-A0J7-01 | Wound Healing (Immune C1) |
| TCGA-SX-A71U-01 | Inflammatory (Immune C3) |
| TCGA-D8-A1XY-01 | IFN-gamma Dominant (Immune C2) |
| TCGA-L5-A43I-01 | Wound Healing (Immune C1) |
| TCGA-EY-A3QX-01 | IFN-gamma Dominant (Immune C2) |
| TCGA-ER-A42H-01 | Lymphocyte Depleted (Immune C4) |
| TCGA-HT-A61C-01 | Lymphocyte Depleted (Immune C4) |
| TCGA-58-8386-01 | Lymphocyte Depleted (Immune C4) |
| TCGA-A2-A0CK-01 | Wound Healing (Immune C1) |
| TCGA-AZ-5403-01 | Wound Healing (Immune C1) |
| TCGA-CN-A6V1-01 | IFN-gamma Dominant (Immune C2) |
| TCGA-V4-A9E9-01 | Lymphocyte Depleted (Immune C4) |
| TCGA-DD-AACJ-01 | Lymphocyte Depleted (Immune C4) |
| TCGA-AC-A8OR-01 | Lymphocyte Depleted (Immune C4) |
| TCGA-G3-AAV5-01 | Lymphocyte Depleted (Immune C4) |
| TCGA-CM-5861-01 | Wound Healing (Immune C1) |
| TCGA-A2-A0YH-01 | IFN-gamma Dominant (Immune C2) |
| TCGA-78-8648-01 | Inflammatory (Immune C3) |
| TCGA-DD-A11B-01 | Inflammatory (Immune C3) |
| TCGA-WC-A888-01 | Inflammatory (Immune C3) |
| TCGA-EJ-7797-01 | Inflammatory (Immune C3) |
| TCGA-UF-A71E-01 | IFN-gamma Dominant (Immune C2) |
| TCGA-G3-A5SK-01 | Lymphocyte Depleted (Immune C4) |
| TCGA-DE-A4MC-01 | Inflammatory (Immune C3) |
| TCGA-A3-A6NL-01 | Inflammatory (Immune C3) |
| TCGA-DW-7834-01 | Inflammatory (Immune C3) |
| TCGA-2G-AAGW-01 | Wound Healing (Immune C1) |
| TCGA-2F-A9KQ-01 | Wound Healing (Immune C1) |
| TCGA-DK-A1A7-01 | Wound Healing (Immune C1) |
| TCGA-D8-A1X6-01 | Lymphocyte Depleted (Immune C4) |
| TCGA-FX-A3TO-01 | Wound Healing (Immune C1) |
| TCGA-DD-AAD5-01 | Lymphocyte Depleted (Immune C4) |
| TCGA-AQ-A7U7-01 | IFN-gamma Dominant (Immune C2) |
| TCGA-TQ-A7RF-01 | Immunologically Quiet (Immune C5) |
| TCGA-B0-4700-01 | TGF-beta Dominant (Immune C6) |
| TCGA-B0-5092-01 | Inflammatory (Immune C3) |
| TCGA-ED-A8O6-01 | Inflammatory (Immune C3) |
| TCGA-E2-A10A-01 | Lymphocyte Depleted (Immune C4) |
| TCGA-EY-A1GR-01 | IFN-gamma Dominant (Immune C2) |
| TCGA-B5-A1MZ-01 | Wound Healing (Immune C1) |
| TCGA-CZ-4859-01 | Inflammatory (Immune C3) |
| TCGA-19-2619-01 | Lymphocyte Depleted (Immune C4) |
| TCGA-31-1956-01 | IFN-gamma Dominant (Immune C2) |
| TCGA-E9-A54Y-01 | Wound Healing (Immune C1) |
| TCGA-CN-A49B-01 | IFN-gamma Dominant (Immune C2) |
| TCGA-CQ-A4CB-01 | IFN-gamma Dominant (Immune C2) |
| TCGA-S7-A7WR-01 | Lymphocyte Depleted (Immune C4) |
| TCGA-B7-A5TN-01 | Wound Healing (Immune C1) |
| TCGA-06-0745-01 | Lymphocyte Depleted (Immune C4) |
| TCGA-94-8491-01 | Wound Healing (Immune C1) |
| TCGA-VS-A9UB-01 | IFN-gamma Dominant (Immune C2) |
| TCGA-LN-A4A3-01 | IFN-gamma Dominant (Immune C2) |
| TCGA-CH-5763-01 | Inflammatory (Immune C3) |
| TCGA-CA-5796-01 | Inflammatory (Immune C3) |
| TCGA-DD-AADJ-01 | Inflammatory (Immune C3) |
| TCGA-A1-A0SF-01 | Wound Healing (Immune C1) |
| TCGA-A7-A13F-01 | Lymphocyte Depleted (Immune C4) |
| TCGA-P3-A6T4-01 | IFN-gamma Dominant (Immune C2) |
| TCGA-ET-A2MX-01 | Inflammatory (Immune C3) |
| TCGA-AZ-4323-01 | Wound Healing (Immune C1) |
| TCGA-EU-5907-01 | Lymphocyte Depleted (Immune C4) |
| TCGA-2G-AALX-01 | Wound Healing (Immune C1) |
| TCGA-QR-A70H-01 | Inflammatory (Immune C3) |
| TCGA-LT-A8JT-01 | Inflammatory (Immune C3) |
| TCGA-DX-A3M1-01 | IFN-gamma Dominant (Immune C2) |
| TCGA-5S-A9Q8-01 | Inflammatory (Immune C3) |
| TCGA-HD-A634-01 | Wound Healing (Immune C1) |
| TCGA-AJ-A3EJ-01 | IFN-gamma Dominant (Immune C2) |
| TCGA-60-2697-01 | Inflammatory (Immune C3) |
| TCGA-G4-6323-01 | Wound Healing (Immune C1) |
| TCGA-HC-7213-01 | Inflammatory (Immune C3) |
| TCGA-UZ-A9PV-01 | Inflammatory (Immune C3) |
| TCGA-D8-A1X9-01 | IFN-gamma Dominant (Immune C2) |
| TCGA-L5-A891-01 | IFN-gamma Dominant (Immune C2) |
| TCGA-E9-A1RC-01 | Wound Healing (Immune C1) |
| TCGA-UF-A719-01 | IFN-gamma Dominant (Immune C2) |
| TCGA-AC-A23C-01 | IFN-gamma Dominant (Immune C2) |
| TCGA-27-1835-01 | Lymphocyte Depleted (Immune C4) |
| TCGA-C8-A8HR-01 | IFN-gamma Dominant (Immune C2) |
| TCGA-AX-A1CF-01 | IFN-gamma Dominant (Immune C2) |
| TCGA-FI-A2CY-01 | IFN-gamma Dominant (Immune C2) |
| TCGA-DX-A2J1-01 | TGF-beta Dominant (Immune C6) |
| TCGA-EM-A3FR-01 | Inflammatory (Immune C3) |
| TCGA-21-5784-01 | Wound Healing (Immune C1) |
| TCGA-D8-A1Y0-01 | IFN-gamma Dominant (Immune C2) |
| TCGA-F7-A61W-01 | IFN-gamma Dominant (Immune C2) |
| TCGA-24-2027-01 | IFN-gamma Dominant (Immune C2) |
| TCGA-V5-A7RE-01 | IFN-gamma Dominant (Immune C2) |
| TCGA-B0-5084-01 | Lymphocyte Depleted (Immune C4) |
| TCGA-QH-A870-01 | Immunologically Quiet (Immune C5) |
| TCGA-EW-A1PG-01 | Wound Healing (Immune C1) |
| TCGA-QS-A5YQ-01 | IFN-gamma Dominant (Immune C2) |
| TCGA-F9-A7VF-01 | Inflammatory (Immune C3) |
| TCGA-BK-A0CB-01 | Wound Healing (Immune C1) |
| TCGA-HC-7209-01 | Inflammatory (Immune C3) |
| TCGA-UT-A88D-01 | Inflammatory (Immune C3) |
| TCGA-D5-6928-01 | IFN-gamma Dominant (Immune C2) |
| TCGA-DZ-6132-01 | Inflammatory (Immune C3) |
| TCGA-P3-A6SX-01 | IFN-gamma Dominant (Immune C2) |
| TCGA-EM-A3OA-01 | Inflammatory (Immune C3) |
| TCGA-E6-A1M0-01 | Wound Healing (Immune C1) |
| TCGA-FB-A78T-01 | Inflammatory (Immune C3) |
| TCGA-AN-A0FN-01 | TGF-beta Dominant (Immune C6) |
| TCGA-GV-A6ZA-01 | IFN-gamma Dominant (Immune C2) |
| TCGA-E7-A4IJ-01 | IFN-gamma Dominant (Immune C2) |
| TCGA-61-2102-01 | Lymphocyte Depleted (Immune C4) |
| TCGA-25-1321-01 | Lymphocyte Depleted (Immune C4) |
| TCGA-AA-3685-01 | IFN-gamma Dominant (Immune C2) |
| TCGA-N1-A6IA-01 | Wound Healing (Immune C1) |
| TCGA-L5-A8NV-01 | IFN-gamma Dominant (Immune C2) |
| TCGA-CI-6623-01 | Wound Healing (Immune C1) |
| TCGA-A8-A08J-01 | IFN-gamma Dominant (Immune C2) |
| TCGA-OR-A5LE-01 | Lymphocyte Depleted (Immune C4) |
| TCGA-C5-A1MF-01 | IFN-gamma Dominant (Immune C2) |
| TCGA-M7-A71Y-01 | Inflammatory (Immune C3) |
| TCGA-AA-A01F-01 | Wound Healing (Immune C1) |
| TCGA-4C-A93U-01 | Inflammatory (Immune C3) |
| TCGA-CN-A6UY-01 | IFN-gamma Dominant (Immune C2) |
| TCGA-98-A53D-01 | TGF-beta Dominant (Immune C6) |
| TCGA-HT-8111-01 | Immunologically Quiet (Immune C5) |
| TCGA-FD-A5BT-01 | IFN-gamma Dominant (Immune C2) |
| TCGA-MQ-A4LM-01 | IFN-gamma Dominant (Immune C2) |
| TCGA-S9-A7QZ-01 | Immunologically Quiet (Immune C5) |
| TCGA-AF-2690-01 | IFN-gamma Dominant (Immune C2) |
| TCGA-12-0619-01 | Lymphocyte Depleted (Immune C4) |
| TCGA-F7-A61V-01 | Wound Healing (Immune C1) |
| TCGA-UB-AA0V-01 | Inflammatory (Immune C3) |
| TCGA-GV-A3QH-01 | IFN-gamma Dominant (Immune C2) |
| TCGA-S7-A7WX-01 | Lymphocyte Depleted (Immune C4) |
| TCGA-EW-A1PF-01 | Wound Healing (Immune C1) |
| TCGA-AO-A03L-01 | IFN-gamma Dominant (Immune C2) |
| TCGA-XY-A9T9-01 | IFN-gamma Dominant (Immune C2) |
| TCGA-BW-A5NP-01 | Inflammatory (Immune C3) |
| TCGA-25-1322-01 | Lymphocyte Depleted (Immune C4) |
| TCGA-AN-A0FY-01 | Wound Healing (Immune C1) |
| TCGA-QK-A8Z7-01 | Wound Healing (Immune C1) |
| TCGA-J4-A6M7-01 | Inflammatory (Immune C3) |
| TCGA-XE-A9SE-01 | IFN-gamma Dominant (Immune C2) |
| TCGA-77-7138-01 | IFN-gamma Dominant (Immune C2) |
| TCGA-L6-A4EU-01 | Inflammatory (Immune C3) |
| TCGA-AC-A7VC-01 | Lymphocyte Depleted (Immune C4) |
| TCGA-KK-A6E0-01 | Inflammatory (Immune C3) |
| TCGA-DD-A4NL-01 | Inflammatory (Immune C3) |
| TCGA-EP-A3JL-01 | IFN-gamma Dominant (Immune C2) |
| TCGA-DA-A960-01 | Lymphocyte Depleted (Immune C4) |
| TCGA-73-7498-01 | Inflammatory (Immune C3) |
| TCGA-SY-A9G0-01 | Inflammatory (Immune C3) |
| TCGA-UC-A7PI-01 | IFN-gamma Dominant (Immune C2) |
| TCGA-E9-A1R3-01 | TGF-beta Dominant (Immune C6) |
| TCGA-E2-A1IN-01 | Wound Healing (Immune C1) |
| TCGA-75-6214-01 | Wound Healing (Immune C1) |
| TCGA-93-7348-01 | Inflammatory (Immune C3) |
| TCGA-XE-AANJ-01 | IFN-gamma Dominant (Immune C2) |
| TCGA-A4-7734-01 | Inflammatory (Immune C3) |
| TCGA-A6-5664-01 | Wound Healing (Immune C1) |
| TCGA-AZ-5407-01 | Wound Healing (Immune C1) |
| TCGA-CD-A4MJ-01 | IFN-gamma Dominant (Immune C2) |
| TCGA-97-8174-01 | Inflammatory (Immune C3) |
| TCGA-EW-A1OV-01 | IFN-gamma Dominant (Immune C2) |
| TCGA-WB-A80N-01 | Inflammatory (Immune C3) |
| TCGA-DX-AB37-01 | IFN-gamma Dominant (Immune C2) |
| TCGA-A2-A25E-01 | Wound Healing (Immune C1) |
| TCGA-DG-A2KL-01 | Wound Healing (Immune C1) |
| TCGA-HU-A4H3-01 | IFN-gamma Dominant (Immune C2) |
| TCGA-DB-5273-01 | Lymphocyte Depleted (Immune C4) |
| TCGA-ET-A3DO-01 | Inflammatory (Immune C3) |
| TCGA-BT-A2LD-01 | IFN-gamma Dominant (Immune C2) |
| TCGA-B0-4839-01 | Inflammatory (Immune C3) |
| TCGA-DK-A1AC-01 | Wound Healing (Immune C1) |
| TCGA-55-8620-01 | IFN-gamma Dominant (Immune C2) |
| TCGA-CZ-5468-01 | TGF-beta Dominant (Immune C6) |
| TCGA-EJ-5499-01 | Wound Healing (Immune C1) |
| TCGA-DD-AAD1-01 | Inflammatory (Immune C3) |
| TCGA-2J-AABU-01 | Wound Healing (Immune C1) |
| TCGA-YS-A95B-01 | Wound Healing (Immune C1) |
| TCGA-58-8390-01 | Wound Healing (Immune C1) |
| TCGA-CN-A6V6-01 | IFN-gamma Dominant (Immune C2) |
| TCGA-EW-A1IY-01 | IFN-gamma Dominant (Immune C2) |
| TCGA-B6-A0X4-01 | Lymphocyte Depleted (Immune C4) |
| TCGA-A6-2676-01 | IFN-gamma Dominant (Immune C2) |
| TCGA-D8-A1XQ-01 | IFN-gamma Dominant (Immune C2) |
| TCGA-KK-A6DY-01 | Inflammatory (Immune C3) |
| TCGA-62-8399-01 | Inflammatory (Immune C3) |
| TCGA-JW-AAVH-01 | Wound Healing (Immune C1) |
| TCGA-EJ-5510-01 | Inflammatory (Immune C3) |
| TCGA-EB-A41B-01 | Lymphocyte Depleted (Immune C4) |
| TCGA-W5-AA36-01 | Lymphocyte Depleted (Immune C4) |
| TCGA-HT-7470-01 | Immunologically Quiet (Immune C5) |
| TCGA-80-5607-01 | IFN-gamma Dominant (Immune C2) |
| TCGA-A2-A0CL-01 | IFN-gamma Dominant (Immune C2) |
| TCGA-KM-8477-01 | Inflammatory (Immune C3) |
| TCGA-CJ-4892-01 | Inflammatory (Immune C3) |
| TCGA-FG-A4MY-01 | Immunologically Quiet (Immune C5) |
| TCGA-2G-AAGP-01 | IFN-gamma Dominant (Immune C2) |
| TCGA-C8-A27A-01 | IFN-gamma Dominant (Immune C2) |
| TCGA-B0-5088-01 | Inflammatory (Immune C3) |
| TCGA-DD-AADF-01 | Lymphocyte Depleted (Immune C4) |
| TCGA-BG-A0VX-01 | Wound Healing (Immune C1) |
| TCGA-29-1762-01 | Lymphocyte Depleted (Immune C4) |
| TCGA-KU-A6H8-01 | Wound Healing (Immune C1) |
| TCGA-CQ-5331-01 | IFN-gamma Dominant (Immune C2) |
| TCGA-61-2110-01 | Lymphocyte Depleted (Immune C4) |
| TCGA-FD-A6TD-01 | IFN-gamma Dominant (Immune C2) |
| TCGA-DJ-A4UP-01 | Inflammatory (Immune C3) |
| TCGA-AA-3679-01 | Wound Healing (Immune C1) |
| TCGA-AY-6196-01 | Inflammatory (Immune C3) |
| TCGA-E9-A249-01 | IFN-gamma Dominant (Immune C2) |
| TCGA-77-7141-01 | IFN-gamma Dominant (Immune C2) |
| TCGA-90-7766-01 | IFN-gamma Dominant (Immune C2) |
| TCGA-HT-8108-01 | Lymphocyte Depleted (Immune C4) |
| TCGA-59-2363-01 | IFN-gamma Dominant (Immune C2) |
| TCGA-3H-AB3K-01 | TGF-beta Dominant (Immune C6) |
| TCGA-N5-A4RV-01 | Wound Healing (Immune C1) |
| TCGA-B0-5711-01 | Inflammatory (Immune C3) |
| TCGA-LN-A49L-01 | Wound Healing (Immune C1) |
| TCGA-BQ-5883-01 | Inflammatory (Immune C3) |
| TCGA-2J-AABE-01 | TGF-beta Dominant (Immune C6) |
| TCGA-DB-A4XE-01 | Immunologically Quiet (Immune C5) |
| TCGA-XR-A8TC-01 | Lymphocyte Depleted (Immune C4) |
| TCGA-CH-5764-01 | Inflammatory (Immune C3) |
| TCGA-BA-A6D8-01 | Wound Healing (Immune C1) |
| TCGA-A8-A07P-01 | Wound Healing (Immune C1) |
| TCGA-43-2581-01 | TGF-beta Dominant (Immune C6) |
| TCGA-DD-AADR-01 | Lymphocyte Depleted (Immune C4) |
| TCGA-PQ-A6FN-01 | Wound Healing (Immune C1) |
| TCGA-DX-A6YR-01 | Wound Healing (Immune C1) |
| TCGA-B3-A6W5-01 | Lymphocyte Depleted (Immune C4) |
| TCGA-DJ-A4UT-01 | Inflammatory (Immune C3) |
| TCGA-XF-A9SL-01 | Inflammatory (Immune C3) |
| TCGA-A2-A0CW-01 | IFN-gamma Dominant (Immune C2) |
| TCGA-DD-AAE3-01 | Lymphocyte Depleted (Immune C4) |
| TCGA-CG-4477-01 | IFN-gamma Dominant (Immune C2) |
| TCGA-B8-4151-01 | Lymphocyte Depleted (Immune C4) |
| TCGA-55-6543-01 | Inflammatory (Immune C3) |
| TCGA-JY-A93F-01 | Wound Healing (Immune C1) |
| TCGA-QT-A5XP-01 | Lymphocyte Depleted (Immune C4) |
| TCGA-W2-A7HF-01 | Inflammatory (Immune C3) |
| TCGA-2Z-A9J5-01 | Inflammatory (Immune C3) |
| TCGA-GN-A4U5-01 | IFN-gamma Dominant (Immune C2) |
| TCGA-DU-8158-01 | Lymphocyte Depleted (Immune C4) |
| TCGA-HT-7474-01 | Immunologically Quiet (Immune C5) |
| TCGA-2J-AABF-01 | IFN-gamma Dominant (Immune C2) |
| TCGA-G9-6363-01 | Wound Healing (Immune C1) |
| TCGA-49-4501-01 | IFN-gamma Dominant (Immune C2) |
| TCGA-78-7156-01 | Inflammatory (Immune C3) |
| TCGA-ZG-A9L2-01 | Inflammatory (Immune C3) |
| TCGA-CV-A461-01 | IFN-gamma Dominant (Immune C2) |
| TCGA-AA-3973-01 | Inflammatory (Immune C3) |
| TCGA-EO-A1Y7-01 | IFN-gamma Dominant (Immune C2) |
| TCGA-2X-A9D6-01 | IFN-gamma Dominant (Immune C2) |
| TCGA-IA-A40X-01 | Inflammatory (Immune C3) |
| TCGA-VN-A88L-01 | Inflammatory (Immune C3) |
| TCGA-TQ-A7RJ-01 | Immunologically Quiet (Immune C5) |
| TCGA-RC-A6M4-01 | Lymphocyte Depleted (Immune C4) |
| TCGA-E2-A108-01 | Inflammatory (Immune C3) |
| TCGA-G7-6789-01 | Inflammatory (Immune C3) |
| TCGA-5C-A9VG-01 | Lymphocyte Depleted (Immune C4) |
| TCGA-GC-A6I1-01 | IFN-gamma Dominant (Immune C2) |
| TCGA-FD-A5BX-01 | Lymphocyte Depleted (Immune C4) |
| TCGA-24-1417-01 | IFN-gamma Dominant (Immune C2) |
| TCGA-BB-8596-01 | Wound Healing (Immune C1) |
| TCGA-G4-6297-01 | Wound Healing (Immune C1) |
| TCGA-S9-A6U0-01 | Lymphocyte Depleted (Immune C4) |
| TCGA-62-A46S-01 | Inflammatory (Immune C3) |
| TCGA-TM-A84I-01 | Immunologically Quiet (Immune C5) |
| TCGA-85-8354-01 | Wound Healing (Immune C1) |
| TCGA-LN-A7HZ-01 | IFN-gamma Dominant (Immune C2) |
| TCGA-AC-A5XU-01 | Wound Healing (Immune C1) |
| TCGA-EJ-5496-01 | Inflammatory (Immune C3) |
| TCGA-E8-A44K-01 | Inflammatory (Immune C3) |
| TCGA-KK-A8IA-01 | Wound Healing (Immune C1) |
| TCGA-OR-A5JA-01 | Lymphocyte Depleted (Immune C4) |
| TCGA-CC-A3MB-01 | Lymphocyte Depleted (Immune C4) |
| TCGA-24-1552-01 | Lymphocyte Depleted (Immune C4) |
| TCGA-49-AARQ-01 | IFN-gamma Dominant (Immune C2) |
| TCGA-AP-A1DO-01 | Wound Healing (Immune C1) |
| TCGA-BP-5190-01 | Inflammatory (Immune C3) |
| TCGA-Q2-A5QZ-01 | Lymphocyte Depleted (Immune C4) |
| TCGA-EJ-5509-01 | Inflammatory (Immune C3) |
| TCGA-25-1326-01 | Wound Healing (Immune C1) |
| TCGA-AN-A0XL-01 | Wound Healing (Immune C1) |
| TCGA-BQ-5892-01 | Inflammatory (Immune C3) |
| TCGA-MY-A5BF-01 | IFN-gamma Dominant (Immune C2) |
| TCGA-L5-A8NU-01 | Inflammatory (Immune C3) |
| TCGA-E9-A1ND-01 | IFN-gamma Dominant (Immune C2) |
| TCGA-AN-A03X-01 | Wound Healing (Immune C1) |
| TCGA-V1-A9OF-01 | Inflammatory (Immune C3) |
| TCGA-S9-A7QY-01 | Immunologically Quiet (Immune C5) |
| TCGA-EL-A3CM-01 | Inflammatory (Immune C3) |
| TCGA-DB-A4XA-01 | Immunologically Quiet (Immune C5) |
| TCGA-XR-A8TG-01 | Inflammatory (Immune C3) |
| TCGA-A8-A08R-01 | IFN-gamma Dominant (Immune C2) |
| TCGA-G7-6797-01 | Inflammatory (Immune C3) |
| TCGA-63-A5MW-01 | Wound Healing (Immune C1) |
| TCGA-XA-A8JR-01 | Lymphocyte Depleted (Immune C4) |
| TCGA-28-5213-01 | Lymphocyte Depleted (Immune C4) |
| TCGA-VQ-A92D-01 | IFN-gamma Dominant (Immune C2) |
| TCGA-EB-A5VU-01 | Wound Healing (Immune C1) |
| TCGA-AG-3894-01 | Wound Healing (Immune C1) |
| TCGA-B1-A656-01 | Inflammatory (Immune C3) |
| TCGA-GV-A3JW-01 | Wound Healing (Immune C1) |
| TCGA-P5-A731-01 | Lymphocyte Depleted (Immune C4) |
| TCGA-J4-A6G1-01 | Inflammatory (Immune C3) |
| TCGA-D5-6540-01 | Wound Healing (Immune C1) |
| TCGA-N8-A4PN-01 | Wound Healing (Immune C1) |
| TCGA-GM-A2DC-01 | Inflammatory (Immune C3) |
| TCGA-93-A4JN-01 | IFN-gamma Dominant (Immune C2) |
| TCGA-E1-A7YE-01 | Lymphocyte Depleted (Immune C4) |
| TCGA-Q1-A5R1-01 | Wound Healing (Immune C1) |
| TCGA-FU-A57G-01 | Wound Healing (Immune C1) |
| TCGA-N5-A4RN-01 | Wound Healing (Immune C1) |
| TCGA-A2-A3XT-01 | IFN-gamma Dominant (Immune C2) |
| TCGA-FG-A710-01 | Lymphocyte Depleted (Immune C4) |
| TCGA-EK-A3GJ-01 | IFN-gamma Dominant (Immune C2) |
| TCGA-H7-A76A-01 | IFN-gamma Dominant (Immune C2) |
| TCGA-OR-A5J5-01 | Lymphocyte Depleted (Immune C4) |
| TCGA-AN-A04C-01 | Wound Healing (Immune C1) |
| TCGA-HT-8010-01 | Immunologically Quiet (Immune C5) |
| TCGA-E8-A44M-01 | Inflammatory (Immune C3) |
| TCGA-BQ-7050-01 | Inflammatory (Immune C3) |
| TCGA-C5-A1BQ-01 | IFN-gamma Dominant (Immune C2) |
| TCGA-2H-A9GR-01 | IFN-gamma Dominant (Immune C2) |
| TCGA-DH-5143-01 | Immunologically Quiet (Immune C5) |
| TCGA-60-2708-01 | Wound Healing (Immune C1) |
| TCGA-DU-7299-01 | Immunologically Quiet (Immune C5) |
| TCGA-HC-7821-01 | Inflammatory (Immune C3) |
| TCGA-KS-A4ID-01 | Inflammatory (Immune C3) |
| TCGA-29-1688-01 | IFN-gamma Dominant (Immune C2) |
| TCGA-B8-A54J-01 | Inflammatory (Immune C3) |
| TCGA-VR-A8ET-01 | IFN-gamma Dominant (Immune C2) |
| TCGA-A2-A0YT-01 | IFN-gamma Dominant (Immune C2) |
| TCGA-91-6847-01 | IFN-gamma Dominant (Immune C2) |
| TCGA-DB-5270-01 | Immunologically Quiet (Immune C5) |
| TCGA-B8-5551-01 | Inflammatory (Immune C3) |
| TCGA-FE-A3PC-01 | Inflammatory (Immune C3) |
| TCGA-CF-A1HS-01 | IFN-gamma Dominant (Immune C2) |
| TCGA-14-0789-01 | Lymphocyte Depleted (Immune C4) |
| TCGA-25-1319-01 | IFN-gamma Dominant (Immune C2) |
| TCGA-22-4593-01 | Wound Healing (Immune C1) |
| TCGA-K1-A42W-01 | Lymphocyte Depleted (Immune C4) |
| TCGA-85-7843-01 | Wound Healing (Immune C1) |
| TCGA-20-1683-01 | IFN-gamma Dominant (Immune C2) |
| TCGA-HE-A5NL-01 | Inflammatory (Immune C3) |
| TCGA-55-6642-01 | Inflammatory (Immune C3) |
| TCGA-34-5240-01 | IFN-gamma Dominant (Immune C2) |
| TCGA-M7-A721-01 | Inflammatory (Immune C3) |
| TCGA-D7-8579-01 | Inflammatory (Immune C3) |
| TCGA-UZ-A9PR-01 | Lymphocyte Depleted (Immune C4) |
| TCGA-B0-4836-01 | Inflammatory (Immune C3) |
| TCGA-36-1575-01 | Lymphocyte Depleted (Immune C4) |
| TCGA-CU-A5W6-01 | IFN-gamma Dominant (Immune C2) |
| TCGA-CG-4443-01 | Lymphocyte Depleted (Immune C4) |
| TCGA-85-A50M-01 | Wound Healing (Immune C1) |
| TCGA-S3-AA10-01 | IFN-gamma Dominant (Immune C2) |
| TCGA-LK-A4NZ-01 | IFN-gamma Dominant (Immune C2) |
| TCGA-CV-7180-01 | IFN-gamma Dominant (Immune C2) |
| TCGA-KT-A74X-01 | Immunologically Quiet (Immune C5) |
| TCGA-BH-A1EO-01 | TGF-beta Dominant (Immune C6) |
| TCGA-DB-A75L-01 | Immunologically Quiet (Immune C5) |
| TCGA-EY-A1H0-01 | Wound Healing (Immune C1) |
| TCGA-AA-3947-01 | Lymphocyte Depleted (Immune C4) |
| TCGA-E2-A107-01 | Wound Healing (Immune C1) |
| TCGA-EK-A2PL-01 | IFN-gamma Dominant (Immune C2) |
| TCGA-55-8203-01 | Inflammatory (Immune C3) |
| TCGA-A6-2686-01 | IFN-gamma Dominant (Immune C2) |
| TCGA-KB-A93H-01 | IFN-gamma Dominant (Immune C2) |
| TCGA-CV-7416-01 | IFN-gamma Dominant (Immune C2) |
| TCGA-PR-A5PH-01 | Inflammatory (Immune C3) |
| TCGA-OR-A5JZ-01 | Inflammatory (Immune C3) |
| TCGA-CJ-4891-01 | TGF-beta Dominant (Immune C6) |
| TCGA-BP-5202-01 | Inflammatory (Immune C3) |
| TCGA-3H-AB3M-01 | Lymphocyte Depleted (Immune C4) |
| TCGA-FD-A6TC-01 | Wound Healing (Immune C1) |
| TCGA-AA-A024-01 | Inflammatory (Immune C3) |
| TCGA-UF-A71A-01 | Wound Healing (Immune C1) |
| TCGA-66-2771-01 | IFN-gamma Dominant (Immune C2) |
| TCGA-D9-A3Z4-01 | IFN-gamma Dominant (Immune C2) |
| TCGA-2J-AAB9-01 | Inflammatory (Immune C3) |
| TCGA-XE-AAOJ-01 | Wound Healing (Immune C1) |
| TCGA-DM-A28F-01 | Wound Healing (Immune C1) |
| TCGA-VR-A8EP-01 | IFN-gamma Dominant (Immune C2) |
| TCGA-CC-A123-01 | Lymphocyte Depleted (Immune C4) |
| TCGA-HT-A616-01 | Immunologically Quiet (Immune C5) |
| TCGA-V1-A8MJ-01 | IFN-gamma Dominant (Immune C2) |
| TCGA-BF-AAOU-01 | Wound Healing (Immune C1) |
| TCGA-RD-A8MV-01 | IFN-gamma Dominant (Immune C2) |
| TCGA-HT-A74K-01 | Lymphocyte Depleted (Immune C4) |
| TCGA-P7-A5NY-01 | Lymphocyte Depleted (Immune C4) |
| TCGA-94-A5I4-01 | IFN-gamma Dominant (Immune C2) |
| TCGA-CV-7090-01 | IFN-gamma Dominant (Immune C2) |
| TCGA-AA-A02W-01 | Wound Healing (Immune C1) |
| TCGA-85-A53L-01 | Wound Healing (Immune C1) |
| TCGA-AO-A0JG-01 | Inflammatory (Immune C3) |
| TCGA-LD-A74U-01 | TGF-beta Dominant (Immune C6) |
| TCGA-WY-A85C-01 | Immunologically Quiet (Immune C5) |
| TCGA-FG-8188-01 | Immunologically Quiet (Immune C5) |
| TCGA-DX-A7EO-01 | Wound Healing (Immune C1) |
| TCGA-CD-5801-01 | IFN-gamma Dominant (Immune C2) |
| TCGA-V1-A8X3-01 | Inflammatory (Immune C3) |
| TCGA-N6-A4V9-01 | IFN-gamma Dominant (Immune C2) |
| TCGA-MK-A4N7-01 | Inflammatory (Immune C3) |
| TCGA-W2-A7HA-01 | Inflammatory (Immune C3) |
| TCGA-LN-A49O-01 | IFN-gamma Dominant (Immune C2) |
| TCGA-BH-A0RX-01 | IFN-gamma Dominant (Immune C2) |
| TCGA-HZ-A77Q-01 | Wound Healing (Immune C1) |
| TCGA-Q1-A6DW-01 | IFN-gamma Dominant (Immune C2) |
| TCGA-C5-A2M1-01 | IFN-gamma Dominant (Immune C2) |
| TCGA-18-3410-01 | IFN-gamma Dominant (Immune C2) |
| TCGA-R6-A8W8-01 | Wound Healing (Immune C1) |
| TCGA-CW-6093-01 | Inflammatory (Immune C3) |
| TCGA-EJ-A46G-01 | Inflammatory (Immune C3) |
| TCGA-R2-A69V-01 | IFN-gamma Dominant (Immune C2) |
| TCGA-J8-A4HW-01 | Inflammatory (Immune C3) |
| TCGA-44-7670-01 | Wound Healing (Immune C1) |
| TCGA-AC-A2QJ-01 | Wound Healing (Immune C1) |
| TCGA-CA-5256-01 | Wound Healing (Immune C1) |
| TCGA-AA-3956-01 | Wound Healing (Immune C1) |
| TCGA-NC-A5HT-01 | IFN-gamma Dominant (Immune C2) |
| TCGA-A6-2682-01 | Wound Healing (Immune C1) |
| TCGA-CV-7421-01 | IFN-gamma Dominant (Immune C2) |
| TCGA-L5-A8NQ-01 | IFN-gamma Dominant (Immune C2) |
| TCGA-DU-A6S7-01 | Immunologically Quiet (Immune C5) |
| TCGA-92-7341-01 | Wound Healing (Immune C1) |
| TCGA-DW-7838-01 | Inflammatory (Immune C3) |
| TCGA-KN-8419-01 | Lymphocyte Depleted (Immune C4) |
| TCGA-CV-7255-01 | IFN-gamma Dominant (Immune C2) |
| TCGA-3B-A9I3-01 | Wound Healing (Immune C1) |
| TCGA-FD-A6TH-01 | IFN-gamma Dominant (Immune C2) |
| TCGA-09-2044-01 | IFN-gamma Dominant (Immune C2) |
| TCGA-WK-A8Y0-01 | Lymphocyte Depleted (Immune C4) |
| TCGA-IB-AAUP-01 | Inflammatory (Immune C3) |
| TCGA-RW-A67V-01 | Lymphocyte Depleted (Immune C4) |
| TCGA-HT-7855-01 | Immunologically Quiet (Immune C5) |
| TCGA-D7-8572-01 | Wound Healing (Immune C1) |
| TCGA-GM-A2DO-01 | IFN-gamma Dominant (Immune C2) |
| TCGA-60-2721-01 | IFN-gamma Dominant (Immune C2) |
| TCGA-TP-A8TT-01 | Inflammatory (Immune C3) |
| TCGA-CJ-4638-01 | Inflammatory (Immune C3) |
| TCGA-06-0747-01 | Lymphocyte Depleted (Immune C4) |
| TCGA-MB-A5Y9-01 | Inflammatory (Immune C3) |
| TCGA-K4-A83P-01 | IFN-gamma Dominant (Immune C2) |
| TCGA-B8-4148-01 | Inflammatory (Immune C3) |
| TCGA-A2-A0T1-01 | Wound Healing (Immune C1) |
| TCGA-BT-A20N-01 | Wound Healing (Immune C1) |
| TCGA-FB-AAPS-01 | TGF-beta Dominant (Immune C6) |
| TCGA-CG-5722-01 | IFN-gamma Dominant (Immune C2) |
| TCGA-D1-A0ZN-01 | Inflammatory (Immune C3) |
| TCGA-38-6178-01 | IFN-gamma Dominant (Immune C2) |
| TCGA-OR-A5JI-01 | Inflammatory (Immune C3) |
| TCGA-85-8052-01 | Wound Healing (Immune C1) |
| TCGA-KV-A6GD-01 | Inflammatory (Immune C3) |
| TCGA-43-3920-01 | Wound Healing (Immune C1) |
| TCGA-B1-A655-01 | Lymphocyte Depleted (Immune C4) |
| TCGA-BH-A1ET-01 | Inflammatory (Immune C3) |
| TCGA-BH-A0EI-01 | Wound Healing (Immune C1) |
| TCGA-C8-A1HO-01 | IFN-gamma Dominant (Immune C2) |
| TCGA-09-1667-01 | IFN-gamma Dominant (Immune C2) |
| TCGA-QT-A5XL-01 | Inflammatory (Immune C3) |
| TCGA-IB-7651-01 | IFN-gamma Dominant (Immune C2) |
| TCGA-E2-A15J-01 | Lymphocyte Depleted (Immune C4) |
| TCGA-22-1000-01 | Wound Healing (Immune C1) |
| TCGA-BP-5187-01 | Inflammatory (Immune C3) |
| TCGA-A1-A0SQ-01 | Inflammatory (Immune C3) |
| TCGA-L5-A8NF-01 | Wound Healing (Immune C1) |
| TCGA-DB-A64X-01 | Immunologically Quiet (Immune C5) |
| TCGA-BP-4971-01 | Inflammatory (Immune C3) |
| TCGA-61-1736-01 | Lymphocyte Depleted (Immune C4) |
| TCGA-CW-6097-01 | Inflammatory (Immune C3) |
| TCGA-B3-8121-01 | Inflammatory (Immune C3) |
| TCGA-BH-A42T-01 | Wound Healing (Immune C1) |
| TCGA-DB-5274-01 | Lymphocyte Depleted (Immune C4) |
| TCGA-52-7812-01 | IFN-gamma Dominant (Immune C2) |
| TCGA-BP-4351-01 | Inflammatory (Immune C3) |
| TCGA-AG-3591-01 | Wound Healing (Immune C1) |
| TCGA-44-6777-01 | Inflammatory (Immune C3) |
| TCGA-FP-A4BE-01 | IFN-gamma Dominant (Immune C2) |
| TCGA-DD-AAEB-01 | Inflammatory (Immune C3) |
| TCGA-BP-5192-01 | Inflammatory (Immune C3) |
| TCGA-QC-A6FX-01 | Lymphocyte Depleted (Immune C4) |
| TCGA-EJ-5522-01 | Inflammatory (Immune C3) |
| TCGA-E2-A1IL-01 | IFN-gamma Dominant (Immune C2) |
| TCGA-CC-5263-01 | Lymphocyte Depleted (Immune C4) |
| TCGA-BR-8380-01 | Wound Healing (Immune C1) |
| TCGA-AN-A04D-01 | Wound Healing (Immune C1) |
| TCGA-55-8616-01 | Inflammatory (Immune C3) |
| TCGA-EA-A5ZF-01 | Wound Healing (Immune C1) |
| TCGA-77-8008-01 | Wound Healing (Immune C1) |
| TCGA-C8-A27B-01 | IFN-gamma Dominant (Immune C2) |
| TCGA-BR-A453-01 | Wound Healing (Immune C1) |
| TCGA-97-A4M0-01 | Inflammatory (Immune C3) |
| TCGA-CV-6959-01 | IFN-gamma Dominant (Immune C2) |
| TCGA-AN-A0AS-01 | TGF-beta Dominant (Immune C6) |
| TCGA-HT-7604-01 | Immunologically Quiet (Immune C5) |
| TCGA-EL-A3ZR-01 | Inflammatory (Immune C3) |
| TCGA-D8-A4Z1-01 | Inflammatory (Immune C3) |
| TCGA-49-6767-01 | Wound Healing (Immune C1) |
| TCGA-BA-4078-01 | Wound Healing (Immune C1) |
| TCGA-A8-A06R-01 | IFN-gamma Dominant (Immune C2) |
| TCGA-FU-A23L-01 | IFN-gamma Dominant (Immune C2) |
| TCGA-TM-A84F-01 | Lymphocyte Depleted (Immune C4) |
| TCGA-YL-A8SA-01 | Lymphocyte Depleted (Immune C4) |
| TCGA-UY-A9PB-01 | IFN-gamma Dominant (Immune C2) |
| TCGA-K7-A6G5-01 | Lymphocyte Depleted (Immune C4) |
| TCGA-D7-6518-01 | Wound Healing (Immune C1) |
| TCGA-AO-A1KP-01 | Wound Healing (Immune C1) |
| TCGA-AA-3531-01 | Wound Healing (Immune C1) |
| TCGA-FE-A3PD-01 | Inflammatory (Immune C3) |
| TCGA-UB-A7MC-01 | Lymphocyte Depleted (Immune C4) |
| TCGA-CG-4440-01 | IFN-gamma Dominant (Immune C2) |
| TCGA-AG-4022-01 | Wound Healing (Immune C1) |
| TCGA-CJ-4895-01 | Inflammatory (Immune C3) |
| TCGA-AA-A01V-01 | Wound Healing (Immune C1) |
| TCGA-A3-3373-01 | Inflammatory (Immune C3) |
| TCGA-BJ-A45G-01 | Inflammatory (Immune C3) |
| TCGA-C8-A12P-01 | IFN-gamma Dominant (Immune C2) |
| TCGA-AG-3605-01 | Wound Healing (Immune C1) |
| TCGA-59-2352-01 | IFN-gamma Dominant (Immune C2) |
| TCGA-DB-A64O-01 | Immunologically Quiet (Immune C5) |
| TCGA-BG-A0YU-01 | Wound Healing (Immune C1) |
| TCGA-LK-A4O4-01 | Inflammatory (Immune C3) |
| TCGA-DU-A5TT-01 | Lymphocyte Depleted (Immune C4) |
| TCGA-DJ-A3V9-01 | Inflammatory (Immune C3) |
| TCGA-55-1595-01 | Wound Healing (Immune C1) |
| TCGA-BH-A8FY-01 | Lymphocyte Depleted (Immune C4) |
| TCGA-CI-6621-01 | Wound Healing (Immune C1) |
| TCGA-5P-A9K2-01 | Inflammatory (Immune C3) |
| TCGA-DB-A75O-01 | Immunologically Quiet (Immune C5) |
| TCGA-24-2288-01 | IFN-gamma Dominant (Immune C2) |
| TCGA-DK-A3IL-01 | Wound Healing (Immune C1) |
| TCGA-HT-A74J-01 | Immunologically Quiet (Immune C5) |
| TCGA-D7-6527-01 | Wound Healing (Immune C1) |
| TCGA-CV-7415-01 | IFN-gamma Dominant (Immune C2) |
| TCGA-D1-A2G7-01 | IFN-gamma Dominant (Immune C2) |
| TCGA-22-5491-01 | IFN-gamma Dominant (Immune C2) |
| TCGA-L7-A6VZ-01 | IFN-gamma Dominant (Immune C2) |
| TCGA-CJ-4637-01 | Inflammatory (Immune C3) |
| TCGA-A8-A09V-01 | Inflammatory (Immune C3) |
| TCGA-GM-A3NY-01 | Wound Healing (Immune C1) |
| TCGA-BH-A5J0-01 | IFN-gamma Dominant (Immune C2) |
| TCGA-E9-A1NG-01 | TGF-beta Dominant (Immune C6) |
| TCGA-61-2008-01 | Wound Healing (Immune C1) |
| TCGA-QQ-A8VF-01 | IFN-gamma Dominant (Immune C2) |
| TCGA-Y6-A8TL-01 | Inflammatory (Immune C3) |
| TCGA-BP-4343-01 | Inflammatory (Immune C3) |
| TCGA-EY-A1GH-01 | IFN-gamma Dominant (Immune C2) |
| TCGA-D1-A0ZU-01 | Wound Healing (Immune C1) |
| TCGA-ZN-A9VO-01 | IFN-gamma Dominant (Immune C2) |
| TCGA-QH-A6CY-01 | Immunologically Quiet (Immune C5) |
| TCGA-B6-A0I6-01 | Wound Healing (Immune C1) |
| TCGA-49-AAR2-01 | IFN-gamma Dominant (Immune C2) |
| TCGA-AX-A3G4-01 | Wound Healing (Immune C1) |
| TCGA-70-6722-01 | IFN-gamma Dominant (Immune C2) |
| TCGA-VQ-A91U-01 | IFN-gamma Dominant (Immune C2) |
| TCGA-S9-A6WM-01 | Lymphocyte Depleted (Immune C4) |
| TCGA-B9-4115-01 | Lymphocyte Depleted (Immune C4) |
| TCGA-FY-A3RA-01 | Inflammatory (Immune C3) |
| TCGA-SX-A7SS-01 | Inflammatory (Immune C3) |
| TCGA-2Z-A9JP-01 | Lymphocyte Depleted (Immune C4) |
| TCGA-4K-AA1I-01 | IFN-gamma Dominant (Immune C2) |
| TCGA-A5-A0GD-01 | IFN-gamma Dominant (Immune C2) |
| TCGA-55-6978-01 | IFN-gamma Dominant (Immune C2) |
| TCGA-28-5218-01 | Wound Healing (Immune C1) |
| TCGA-DS-A1OA-01 | IFN-gamma Dominant (Immune C2) |
| TCGA-MI-A75E-01 | Inflammatory (Immune C3) |
| TCGA-W6-AA0S-01 | Wound Healing (Immune C1) |
| TCGA-AA-A00R-01 | IFN-gamma Dominant (Immune C2) |
| TCGA-VQ-AA69-01 | Lymphocyte Depleted (Immune C4) |
| TCGA-HC-7233-01 | Inflammatory (Immune C3) |
| TCGA-P4-AAVK-01 | Lymphocyte Depleted (Immune C4) |
| TCGA-CQ-6227-01 | IFN-gamma Dominant (Immune C2) |
| TCGA-AC-A6NO-01 | Wound Healing (Immune C1) |
| TCGA-A4-A772-01 | Inflammatory (Immune C3) |
| TCGA-DX-A3UF-01 | Wound Healing (Immune C1) |
| TCGA-A3-3367-01 | Inflammatory (Immune C3) |
| TCGA-BA-A6DL-01 | IFN-gamma Dominant (Immune C2) |
| TCGA-BR-6706-01 | IFN-gamma Dominant (Immune C2) |
| TCGA-49-AARR-01 | Inflammatory (Immune C3) |
| TCGA-85-8276-01 | Wound Healing (Immune C1) |
| TCGA-CJ-4887-01 | Inflammatory (Immune C3) |
| TCGA-B6-A0I5-01 | Wound Healing (Immune C1) |
| TCGA-BH-A18I-01 | Wound Healing (Immune C1) |
| TCGA-D1-A1O7-01 | IFN-gamma Dominant (Immune C2) |
| TCGA-BA-A8YP-01 | Wound Healing (Immune C1) |
| TCGA-55-6972-01 | Lymphocyte Depleted (Immune C4) |
| TCGA-QH-A6CV-01 | Lymphocyte Depleted (Immune C4) |
| TCGA-XR-A8TD-01 | IFN-gamma Dominant (Immune C2) |
| TCGA-CR-7365-01 | IFN-gamma Dominant (Immune C2) |
| TCGA-CH-5744-01 | Inflammatory (Immune C3) |
| TCGA-OR-A5LA-01 | Inflammatory (Immune C3) |
| TCGA-B5-A3FD-01 | Wound Healing (Immune C1) |
| TCGA-DE-A69K-01 | Inflammatory (Immune C3) |
| TCGA-G4-6306-01 | Wound Healing (Immune C1) |
| TCGA-D8-A140-01 | IFN-gamma Dominant (Immune C2) |
| TCGA-CG-5719-01 | Wound Healing (Immune C1) |
| TCGA-A2-A04T-01 | IFN-gamma Dominant (Immune C2) |
| TCGA-ZF-AA51-01 | Wound Healing (Immune C1) |
| TCGA-TS-A8AY-01 | Wound Healing (Immune C1) |
| TCGA-CJ-5676-01 | Inflammatory (Immune C3) |
| TCGA-PK-A5HA-01 | Lymphocyte Depleted (Immune C4) |
| TCGA-DJ-A3UU-01 | Inflammatory (Immune C3) |
| TCGA-DX-A8BZ-01 | Lymphocyte Depleted (Immune C4) |
| TCGA-56-8625-01 | IFN-gamma Dominant (Immune C2) |
| TCGA-60-2703-01 | Wound Healing (Immune C1) |
| TCGA-S7-A7X1-01 | Inflammatory (Immune C3) |
| TCGA-DD-A3A7-01 | Inflammatory (Immune C3) |
| TCGA-A8-A09Z-01 | Inflammatory (Immune C3) |
| TCGA-NC-A5HH-01 | Wound Healing (Immune C1) |
| TCGA-B0-4710-01 | Inflammatory (Immune C3) |
| TCGA-BA-5555-01 | IFN-gamma Dominant (Immune C2) |
| TCGA-CZ-5465-01 | Inflammatory (Immune C3) |
| TCGA-BP-4338-01 | Inflammatory (Immune C3) |
| TCGA-E9-A1RH-01 | Wound Healing (Immune C1) |
| TCGA-CR-6488-01 | Wound Healing (Immune C1) |
| TCGA-V1-A9OA-01 | Inflammatory (Immune C3) |
| TCGA-CN-4734-01 | IFN-gamma Dominant (Immune C2) |
| TCGA-BP-4176-01 | Inflammatory (Immune C3) |
| TCGA-AG-3731-01 | Wound Healing (Immune C1) |
| TCGA-B0-4817-01 | Inflammatory (Immune C3) |
| TCGA-VP-A87J-01 | Inflammatory (Immune C3) |
| TCGA-W5-AA2W-01 | Lymphocyte Depleted (Immune C4) |
| TCGA-GM-A2DH-01 | IFN-gamma Dominant (Immune C2) |
| TCGA-CN-5356-01 | Wound Healing (Immune C1) |
| TCGA-60-2722-01 | Wound Healing (Immune C1) |
| TCGA-Y8-A8RY-01 | Inflammatory (Immune C3) |
| TCGA-BH-A1ES-01 | Wound Healing (Immune C1) |
| TCGA-EM-A2CP-01 | Inflammatory (Immune C3) |
| TCGA-EM-A3FO-01 | Inflammatory (Immune C3) |
| TCGA-EJ-7321-01 | Inflammatory (Immune C3) |
| TCGA-PL-A8LY-01 | Wound Healing (Immune C1) |
| TCGA-LN-A49X-01 | IFN-gamma Dominant (Immune C2) |
| TCGA-A3-3347-01 | Inflammatory (Immune C3) |
| TCGA-BA-A6DG-01 | Wound Healing (Immune C1) |
| TCGA-EY-A54A-01 | Wound Healing (Immune C1) |
| TCGA-13-1403-01 | IFN-gamma Dominant (Immune C2) |
| TCGA-AG-A02N-01 | Wound Healing (Immune C1) |
| TCGA-DJ-A3VB-01 | IFN-gamma Dominant (Immune C2) |
| TCGA-NA-A4QX-01 | Wound Healing (Immune C1) |
| TCGA-OL-A5RW-01 | IFN-gamma Dominant (Immune C2) |
| TCGA-36-1578-01 | IFN-gamma Dominant (Immune C2) |
| TCGA-FX-A3NJ-01 | Inflammatory (Immune C3) |
| TCGA-BH-A18M-01 | IFN-gamma Dominant (Immune C2) |
| TCGA-86-8073-01 | Inflammatory (Immune C3) |
| TCGA-E2-A14R-01 | IFN-gamma Dominant (Immune C2) |
| TCGA-EB-A42Z-01 | Wound Healing (Immune C1) |
| TCGA-EK-A2H1-01 | IFN-gamma Dominant (Immune C2) |
| TCGA-LP-A4AU-01 | IFN-gamma Dominant (Immune C2) |
| TCGA-MN-A4N4-01 | Wound Healing (Immune C1) |
| TCGA-A8-A09M-01 | IFN-gamma Dominant (Immune C2) |
| TCGA-EM-A2CT-01 | Inflammatory (Immune C3) |
| TCGA-VS-A9U7-01 | IFN-gamma Dominant (Immune C2) |
| TCGA-DU-5852-01 | Lymphocyte Depleted (Immune C4) |
| TCGA-A2-A0CU-01 | Wound Healing (Immune C1) |
| TCGA-AA-A00A-01 | Wound Healing (Immune C1) |
| TCGA-DD-AACP-01 | Wound Healing (Immune C1) |
| TCGA-EJ-5521-01 | Inflammatory (Immune C3) |
| TCGA-K7-AAU7-01 | Wound Healing (Immune C1) |
| TCGA-DJ-A3UQ-01 | Inflammatory (Immune C3) |
| TCGA-EO-A3L0-01 | IFN-gamma Dominant (Immune C2) |
| TCGA-P5-A72U-01 | Inflammatory (Immune C3) |
| TCGA-EM-A3AI-01 | Inflammatory (Immune C3) |
| TCGA-B0-4714-01 | Inflammatory (Immune C3) |
| TCGA-IG-A6QS-01 | IFN-gamma Dominant (Immune C2) |
| TCGA-BB-A5HU-01 | Wound Healing (Immune C1) |
| TCGA-CZ-5461-01 | Inflammatory (Immune C3) |
| TCGA-33-4582-01 | IFN-gamma Dominant (Immune C2) |
| TCGA-A1-A0SN-01 | Wound Healing (Immune C1) |
| TCGA-CV-7434-01 | IFN-gamma Dominant (Immune C2) |
| TCGA-E7-A7PW-01 | Wound Healing (Immune C1) |
| TCGA-DX-A3U5-01 | Lymphocyte Depleted (Immune C4) |
| TCGA-DJ-A3VA-01 | Inflammatory (Immune C3) |
| TCGA-28-2514-01 | Lymphocyte Depleted (Immune C4) |
| TCGA-EB-A51B-01 | IFN-gamma Dominant (Immune C2) |
| TCGA-06-5859-01 | Lymphocyte Depleted (Immune C4) |
| TCGA-CJ-4870-01 | Inflammatory (Immune C3) |
| TCGA-KK-A7AU-01 | Lymphocyte Depleted (Immune C4) |
| TCGA-97-A4M3-01 | Inflammatory (Immune C3) |
| TCGA-DB-A75K-01 | Lymphocyte Depleted (Immune C4) |
| TCGA-5P-A9JY-01 | Inflammatory (Immune C3) |
| TCGA-86-7714-01 | Inflammatory (Immune C3) |
| TCGA-G3-A25T-01 | Wound Healing (Immune C1) |
| TCGA-BT-A20J-01 | IFN-gamma Dominant (Immune C2) |
| TCGA-49-4487-01 | IFN-gamma Dominant (Immune C2) |
| TCGA-IG-A50L-01 | Wound Healing (Immune C1) |
| TCGA-IQ-7632-01 | IFN-gamma Dominant (Immune C2) |
| TCGA-VQ-A8PC-01 | IFN-gamma Dominant (Immune C2) |
| TCGA-78-7147-01 | IFN-gamma Dominant (Immune C2) |
| TCGA-B5-A0JX-01 | IFN-gamma Dominant (Immune C2) |
| TCGA-EL-A3D6-01 | Inflammatory (Immune C3) |
| TCGA-78-7542-01 | IFN-gamma Dominant (Immune C2) |
| TCGA-VD-AA8Q-01 | Inflammatory (Immune C3) |
| TCGA-57-1994-01 | IFN-gamma Dominant (Immune C2) |
| TCGA-BH-A18F-01 | Wound Healing (Immune C1) |
| TCGA-85-8666-01 | IFN-gamma Dominant (Immune C2) |
| TCGA-95-A4VK-01 | Inflammatory (Immune C3) |
| TCGA-3B-A9HS-01 | Inflammatory (Immune C3) |
| TCGA-35-3615-01 | IFN-gamma Dominant (Immune C2) |
| TCGA-EK-A2RC-01 | IFN-gamma Dominant (Immune C2) |
| TCGA-2Y-A9GY-01 | IFN-gamma Dominant (Immune C2) |
| TCGA-HD-8635-01 | IFN-gamma Dominant (Immune C2) |
| TCGA-CR-7399-01 | IFN-gamma Dominant (Immune C2) |
| TCGA-DM-A288-01 | Lymphocyte Depleted (Immune C4) |
| TCGA-85-A4PA-01 | IFN-gamma Dominant (Immune C2) |
| TCGA-D1-A17N-01 | Inflammatory (Immune C3) |
| TCGA-BB-7871-01 | IFN-gamma Dominant (Immune C2) |
| TCGA-HP-A5MZ-01 | Inflammatory (Immune C3) |
| TCGA-DD-AACG-01 | Lymphocyte Depleted (Immune C4) |
| TCGA-24-1469-01 | IFN-gamma Dominant (Immune C2) |
| TCGA-CZ-4864-01 | Inflammatory (Immune C3) |
| TCGA-DE-A4MA-01 | Inflammatory (Immune C3) |
| TCGA-CV-7103-01 | Wound Healing (Immune C1) |
| TCGA-CN-4741-01 | IFN-gamma Dominant (Immune C2) |
| TCGA-VQ-A8PK-01 | IFN-gamma Dominant (Immune C2) |
| TCGA-HC-7744-01 | Inflammatory (Immune C3) |
| TCGA-XV-AAZY-01 | IFN-gamma Dominant (Immune C2) |
| TCGA-EY-A1GK-01 | Wound Healing (Immune C1) |
| TCGA-CM-4746-01 | Wound Healing (Immune C1) |
| TCGA-D1-A0ZR-01 | Inflammatory (Immune C3) |
| TCGA-AR-A2LM-01 | Inflammatory (Immune C3) |
| TCGA-E2-A10C-01 | IFN-gamma Dominant (Immune C2) |
| TCGA-AX-A05T-01 | Wound Healing (Immune C1) |
| TCGA-VQ-AA6G-01 | IFN-gamma Dominant (Immune C2) |
| TCGA-WK-A8XT-01 | Inflammatory (Immune C3) |
| TCGA-OR-A5JE-01 | Lymphocyte Depleted (Immune C4) |
| TCGA-DK-AA74-01 | IFN-gamma Dominant (Immune C2) |
| TCGA-S7-A7WV-01 | TGF-beta Dominant (Immune C6) |
| TCGA-E8-A417-01 | Inflammatory (Immune C3) |
| TCGA-49-6742-01 | IFN-gamma Dominant (Immune C2) |
| TCGA-66-2789-01 | Wound Healing (Immune C1) |
| TCGA-GD-A2C5-01 | IFN-gamma Dominant (Immune C2) |
| TCGA-BP-4164-01 | Inflammatory (Immune C3) |
| TCGA-A5-A2K3-01 | IFN-gamma Dominant (Immune C2) |
| TCGA-MP-A4T6-01 | Inflammatory (Immune C3) |
| TCGA-AR-A0TW-01 | IFN-gamma Dominant (Immune C2) |
| TCGA-B6-A0I1-01 | IFN-gamma Dominant (Immune C2) |
| TCGA-EW-A1J1-01 | IFN-gamma Dominant (Immune C2) |
| TCGA-BH-A0H9-01 | Wound Healing (Immune C1) |
| TCGA-DU-5847-01 | Lymphocyte Depleted (Immune C4) |
| TCGA-28-2509-01 | Lymphocyte Depleted (Immune C4) |
| TCGA-QH-A65Z-01 | Immunologically Quiet (Immune C5) |
| TCGA-AA-A00U-01 | Wound Healing (Immune C1) |
| TCGA-CJ-4874-01 | Inflammatory (Immune C3) |
| TCGA-CH-5740-01 | Inflammatory (Immune C3) |
| TCGA-MP-A4TA-01 | IFN-gamma Dominant (Immune C2) |
| TCGA-13-0799-01 | IFN-gamma Dominant (Immune C2) |
| TCGA-EJ-5503-01 | Inflammatory (Immune C3) |
| TCGA-P4-AAVL-01 | TGF-beta Dominant (Immune C6) |
| TCGA-BH-A0W5-01 | Inflammatory (Immune C3) |
| TCGA-YR-A95A-01 | Inflammatory (Immune C3) |
| TCGA-AA-3518-01 | Wound Healing (Immune C1) |
| TCGA-WW-A8ZI-01 | Inflammatory (Immune C3) |
| TCGA-AR-A24K-01 | Wound Healing (Immune C1) |
| TCGA-B5-A0JS-01 | Inflammatory (Immune C3) |
| TCGA-AX-A0IW-01 | IFN-gamma Dominant (Immune C2) |
| TCGA-CV-A6JU-01 | IFN-gamma Dominant (Immune C2) |
| TCGA-14-1825-01 | Lymphocyte Depleted (Immune C4) |
| TCGA-KL-8326-01 | Inflammatory (Immune C3) |
| TCGA-MB-A5Y8-01 | Lymphocyte Depleted (Immune C4) |
| TCGA-C5-A2LS-01 | Wound Healing (Immune C1) |
| TCGA-DY-A1DE-01 | IFN-gamma Dominant (Immune C2) |
| TCGA-BA-A6DA-01 | IFN-gamma Dominant (Immune C2) |
| TCGA-KO-8406-01 | Immunologically Quiet (Immune C5) |
| TCGA-BP-4964-01 | Inflammatory (Immune C3) |
| TCGA-A3-3335-01 | Inflammatory (Immune C3) |
| TCGA-S9-A7IY-01 | Immunologically Quiet (Immune C5) |
| TCGA-BH-A0HY-01 | Wound Healing (Immune C1) |
| TCGA-VQ-A8P8-01 | IFN-gamma Dominant (Immune C2) |
| TCGA-66-2793-01 | Wound Healing (Immune C1) |
| TCGA-68-7757-01 | Wound Healing (Immune C1) |
| TCGA-GU-AATO-01 | Wound Healing (Immune C1) |
| TCGA-38-A44F-01 | Inflammatory (Immune C3) |
| TCGA-T3-A92M-01 | IFN-gamma Dominant (Immune C2) |
| TCGA-EM-A2P2-01 | Lymphocyte Depleted (Immune C4) |
| TCGA-55-8091-01 | Inflammatory (Immune C3) |
| TCGA-P5-A781-01 | Immunologically Quiet (Immune C5) |
| TCGA-CV-7178-01 | IFN-gamma Dominant (Immune C2) |
| TCGA-AX-A1CK-01 | Wound Healing (Immune C1) |
| TCGA-92-8065-01 | IFN-gamma Dominant (Immune C2) |
| TCGA-60-2726-01 | Wound Healing (Immune C1) |
| TCGA-A4-8310-01 | Lymphocyte Depleted (Immune C4) |
| TCGA-3E-AAAY-01 | IFN-gamma Dominant (Immune C2) |
| TCGA-QR-A70Q-01 | Inflammatory (Immune C3) |
| TCGA-DD-A1EJ-01 | Lymphocyte Depleted (Immune C4) |
| TCGA-BT-A3PK-01 | IFN-gamma Dominant (Immune C2) |
| TCGA-FY-A3I4-01 | Inflammatory (Immune C3) |
| TCGA-CH-5739-01 | Inflammatory (Immune C3) |
| TCGA-LN-A49P-01 | IFN-gamma Dominant (Immune C2) |
| TCGA-B0-4852-01 | Inflammatory (Immune C3) |
| TCGA-D8-A1XW-01 | Inflammatory (Immune C3) |
| TCGA-DX-A2J0-01 | Inflammatory (Immune C3) |
| TCGA-AN-A0FL-01 | IFN-gamma Dominant (Immune C2) |
| TCGA-E8-A418-01 | Lymphocyte Depleted (Immune C4) |
| TCGA-F5-6465-01 | Wound Healing (Immune C1) |
| TCGA-91-6828-01 | Inflammatory (Immune C3) |
| TCGA-2Y-A9H5-01 | Inflammatory (Immune C3) |
| TCGA-PL-A8LZ-01 | IFN-gamma Dominant (Immune C2) |
| TCGA-J9-A52B-01 | Inflammatory (Immune C3) |
| TCGA-DX-A6B7-01 | Inflammatory (Immune C3) |
| TCGA-K4-A54R-01 | IFN-gamma Dominant (Immune C2) |
| TCGA-DU-6405-01 | Lymphocyte Depleted (Immune C4) |
| TCGA-MQ-A4LP-01 | Lymphocyte Depleted (Immune C4) |
| TCGA-DK-A1A6-01 | IFN-gamma Dominant (Immune C2) |
| TCGA-2Z-A9J8-01 | Lymphocyte Depleted (Immune C4) |
| TCGA-K4-A5RI-01 | Wound Healing (Immune C1) |
| TCGA-AC-A2BK-01 | Wound Healing (Immune C1) |
| TCGA-MK-A4N9-01 | Inflammatory (Immune C3) |
| TCGA-UT-A88E-01 | IFN-gamma Dominant (Immune C2) |
| TCGA-AJ-A6NU-01 | IFN-gamma Dominant (Immune C2) |
| TCGA-39-5021-01 | Wound Healing (Immune C1) |
| TCGA-CN-6010-01 | Wound Healing (Immune C1) |
| TCGA-34-5929-01 | IFN-gamma Dominant (Immune C2) |
| TCGA-AC-A7VB-01 | IFN-gamma Dominant (Immune C2) |
| TCGA-N9-A4Q8-01 | Wound Healing (Immune C1) |
| TCGA-EJ-A7NM-01 | Inflammatory (Immune C3) |
| TCGA-VP-A87E-01 | Inflammatory (Immune C3) |
| TCGA-BP-5173-01 | Inflammatory (Immune C3) |
| TCGA-58-A46L-01 | Wound Healing (Immune C1) |
| TCGA-52-7809-01 | Wound Healing (Immune C1) |
| TCGA-TT-A6YJ-01 | Lymphocyte Depleted (Immune C4) |
| TCGA-EJ-5525-01 | Wound Healing (Immune C1) |
| TCGA-CV-A6JY-01 | Wound Healing (Immune C1) |
| TCGA-A5-A0G3-01 | IFN-gamma Dominant (Immune C2) |
| TCGA-5C-A9VH-01 | Lymphocyte Depleted (Immune C4) |
| TCGA-WP-A9GB-01 | Lymphocyte Depleted (Immune C4) |
| TCGA-WB-A821-01 | Inflammatory (Immune C3) |
| TCGA-AJ-A3I9-01 | Wound Healing (Immune C1) |
| TCGA-EJ-A65J-01 | Lymphocyte Depleted (Immune C4) |
| TCGA-E2-A1IG-01 | Wound Healing (Immune C1) |
| TCGA-DM-A1HB-01 | Wound Healing (Immune C1) |
| TCGA-BR-A4PF-01 | IFN-gamma Dominant (Immune C2) |
| TCGA-2Y-A9HB-01 | Lymphocyte Depleted (Immune C4) |
| TCGA-CN-6992-01 | Wound Healing (Immune C1) |
| TCGA-B4-5832-01 | Lymphocyte Depleted (Immune C4) |
| TCGA-DJ-A3VE-01 | Inflammatory (Immune C3) |
| TCGA-66-2782-01 | IFN-gamma Dominant (Immune C2) |
| TCGA-DJ-A1QQ-01 | Inflammatory (Immune C3) |
| TCGA-E7-A519-01 | Lymphocyte Depleted (Immune C4) |
| TCGA-X6-A7W8-01 | Lymphocyte Depleted (Immune C4) |
| TCGA-AO-A0J2-01 | IFN-gamma Dominant (Immune C2) |
| TCGA-EJ-7123-01 | Inflammatory (Immune C3) |
| TCGA-97-A4M7-01 | Inflammatory (Immune C3) |
| TCGA-6A-AB49-01 | Wound Healing (Immune C1) |
| TCGA-BH-A28Q-01 | Inflammatory (Immune C3) |
| TCGA-5P-A9JU-01 | Lymphocyte Depleted (Immune C4) |
| TCGA-2J-AABT-01 | Inflammatory (Immune C3) |
| TCGA-37-3783-01 | IFN-gamma Dominant (Immune C2) |
| TCGA-AC-A2FB-01 | IFN-gamma Dominant (Immune C2) |
| TCGA-C8-A12L-01 | Wound Healing (Immune C1) |
| TCGA-66-2768-01 | IFN-gamma Dominant (Immune C2) |
| TCGA-55-7815-01 | Inflammatory (Immune C3) |
| TCGA-FP-7735-01 | Wound Healing (Immune C1) |
| TCGA-OL-A66N-01 | Inflammatory (Immune C3) |
| TCGA-78-7539-01 | Inflammatory (Immune C3) |
| TCGA-D6-A6EQ-01 | IFN-gamma Dominant (Immune C2) |
| TCGA-J2-8192-01 | Inflammatory (Immune C3) |
| TCGA-HC-A48F-01 | Inflammatory (Immune C3) |
| TCGA-HB-A43Z-01 | Lymphocyte Depleted (Immune C4) |
| TCGA-CM-6170-01 | Wound Healing (Immune C1) |
| TCGA-GF-A2C7-01 | Wound Healing (Immune C1) |
| TCGA-77-7335-01 | IFN-gamma Dominant (Immune C2) |
| TCGA-3C-AALK-01 | Wound Healing (Immune C1) |
| TCGA-KK-A6E6-01 | Inflammatory (Immune C3) |
| TCGA-25-2392-01 | Lymphocyte Depleted (Immune C4) |
| TCGA-2F-A9KO-01 | IFN-gamma Dominant (Immune C2) |
| TCGA-OR-A5K2-01 | Lymphocyte Depleted (Immune C4) |
| TCGA-FV-A495-01 | IFN-gamma Dominant (Immune C2) |
| TCGA-FY-A3ON-01 | Inflammatory (Immune C3) |
| TCGA-AP-A054-01 | Wound Healing (Immune C1) |
| TCGA-AA-3821-01 | Wound Healing (Immune C1) |
| TCGA-43-7656-01 | IFN-gamma Dominant (Immune C2) |
| TCGA-FG-A6J3-01 | Lymphocyte Depleted (Immune C4) |
| TCGA-EQ-A4SO-01 | Wound Healing (Immune C1) |
| TCGA-B0-5113-01 | Inflammatory (Immune C3) |
| TCGA-KL-8334-01 | Inflammatory (Immune C3) |
| TCGA-DV-5566-01 | Inflammatory (Immune C3) |
| TCGA-HW-A5KK-01 | Immunologically Quiet (Immune C5) |
| TCGA-ES-A2HT-01 | Lymphocyte Depleted (Immune C4) |
| TCGA-LD-A66U-01 | Wound Healing (Immune C1) |
| TCGA-AP-A05H-01 | IFN-gamma Dominant (Immune C2) |
| TCGA-AO-A1KO-01 | Inflammatory (Immune C3) |
| TCGA-WB-A81R-01 | Lymphocyte Depleted (Immune C4) |
| TCGA-E1-5311-01 | Immunologically Quiet (Immune C5) |
| TCGA-85-8479-01 | Wound Healing (Immune C1) |
| TCGA-V4-A9EE-01 | Lymphocyte Depleted (Immune C4) |
| TCGA-V3-A9ZY-01 | Lymphocyte Depleted (Immune C4) |
| TCGA-KO-8414-01 | Lymphocyte Depleted (Immune C4) |
| TCGA-OR-A5KU-01 | Lymphocyte Depleted (Immune C4) |
| TCGA-C5-A3HF-01 | IFN-gamma Dominant (Immune C2) |
| TCGA-BF-A1Q0-01 | Wound Healing (Immune C1) |
| TCGA-23-1109-01 | Wound Healing (Immune C1) |
| TCGA-EM-A1CW-01 | Inflammatory (Immune C3) |
| TCGA-KC-A7F6-01 | Inflammatory (Immune C3) |
| TCGA-KN-8424-01 | Inflammatory (Immune C3) |
| TCGA-AS-3777-01 | Lymphocyte Depleted (Immune C4) |
| TCGA-OR-A5K8-01 | Inflammatory (Immune C3) |
| TCGA-A5-A2K7-01 | IFN-gamma Dominant (Immune C2) |
| TCGA-LK-A4NY-01 | Wound Healing (Immune C1) |
| TCGA-XF-A9SZ-01 | Wound Healing (Immune C1) |
| TCGA-B4-5836-01 | Inflammatory (Immune C3) |
| TCGA-85-8580-01 | Wound Healing (Immune C1) |
| TCGA-BR-7958-01 | IFN-gamma Dominant (Immune C2) |
| TCGA-YH-A8SY-01 | IFN-gamma Dominant (Immune C2) |
| TCGA-EM-A4FN-01 | Inflammatory (Immune C3) |
| TCGA-AR-A2LJ-01 | TGF-beta Dominant (Immune C6) |
| TCGA-EJ-7786-01 | Inflammatory (Immune C3) |
| TCGA-L1-A7W4-01 | Wound Healing (Immune C1) |
| TCGA-FG-7634-01 | Lymphocyte Depleted (Immune C4) |
| TCGA-F5-6810-01 | Wound Healing (Immune C1) |
| TCGA-EL-A4JX-01 | Inflammatory (Immune C3) |
| TCGA-BC-A216-01 | Wound Healing (Immune C1) |
| TCGA-XF-AAN1-01 | IFN-gamma Dominant (Immune C2) |
| TCGA-ED-A82E-01 | Inflammatory (Immune C3) |
| TCGA-EB-A4OY-01 | Inflammatory (Immune C3) |
| TCGA-CQ-5333-01 | IFN-gamma Dominant (Immune C2) |
| TCGA-B5-A11X-01 | Wound Healing (Immune C1) |
| TCGA-EJ-A7NF-01 | Inflammatory (Immune C3) |
| TCGA-DK-A3IK-01 | Inflammatory (Immune C3) |
| TCGA-GL-7966-01 | Inflammatory (Immune C3) |
| TCGA-63-7022-01 | Wound Healing (Immune C1) |
| TCGA-2J-AAB4-01 | Wound Healing (Immune C1) |
| TCGA-AR-A0TP-01 | Wound Healing (Immune C1) |
| TCGA-B0-5705-01 | Inflammatory (Immune C3) |
| TCGA-DJ-A1QE-01 | Inflammatory (Immune C3) |
| TCGA-S7-A7WN-01 | Inflammatory (Immune C3) |
| TCGA-18-3408-01 | IFN-gamma Dominant (Immune C2) |
| TCGA-56-8626-01 | Wound Healing (Immune C1) |
| TCGA-SR-A6MV-01 | Inflammatory (Immune C3) |
| TCGA-IN-8462-01 | IFN-gamma Dominant (Immune C2) |
| TCGA-MQ-A6BL-01 | IFN-gamma Dominant (Immune C2) |
| TCGA-DK-A2HX-01 | Wound Healing (Immune C1) |
| TCGA-A8-A07I-01 | IFN-gamma Dominant (Immune C2) |
| TCGA-77-A5G8-01 | Wound Healing (Immune C1) |
| TCGA-CG-5726-01 | Wound Healing (Immune C1) |
| TCGA-OR-A5JC-01 | Lymphocyte Depleted (Immune C4) |
| TCGA-A2-A0EY-01 | Wound Healing (Immune C1) |
| TCGA-VQ-A91Q-01 | IFN-gamma Dominant (Immune C2) |
| TCGA-EL-A3N2-01 | Inflammatory (Immune C3) |
| TCGA-DD-AADP-01 | Inflammatory (Immune C3) |
| TCGA-CZ-5457-01 | Inflammatory (Immune C3) |
| TCGA-AF-6655-01 | IFN-gamma Dominant (Immune C2) |
| TCGA-BP-4782-01 | Inflammatory (Immune C3) |
| TCGA-BH-A0B1-01 | IFN-gamma Dominant (Immune C2) |
| TCGA-YG-AA3N-01 | Inflammatory (Immune C3) |
| TCGA-V4-A9EJ-01 | Lymphocyte Depleted (Immune C4) |
| TCGA-2Y-A9GU-01 | Inflammatory (Immune C3) |
| TCGA-DK-AA6P-01 | Wound Healing (Immune C1) |
| TCGA-XF-A9SM-01 | IFN-gamma Dominant (Immune C2) |
| TCGA-CF-A8HX-01 | Inflammatory (Immune C3) |
| TCGA-C5-A2LT-01 | Wound Healing (Immune C1) |
| TCGA-B5-A11W-01 | Wound Healing (Immune C1) |
| TCGA-CN-A497-01 | Wound Healing (Immune C1) |
| TCGA-HD-7754-01 | Wound Healing (Immune C1) |
| TCGA-EB-A85J-01 | IFN-gamma Dominant (Immune C2) |
| TCGA-AA-3818-01 | Wound Healing (Immune C1) |
| TCGA-EM-A2P1-01 | Inflammatory (Immune C3) |
| TCGA-B1-A47N-01 | Inflammatory (Immune C3) |
| TCGA-G9-6361-01 | Inflammatory (Immune C3) |
| TCGA-D7-A748-01 | TGF-beta Dominant (Immune C6) |
| TCGA-H2-A3RH-01 | Inflammatory (Immune C3) |
| TCGA-HT-A4DV-01 | Immunologically Quiet (Immune C5) |
| TCGA-DU-6396-01 | Lymphocyte Depleted (Immune C4) |
| TCGA-QR-A6H1-01 | Lymphocyte Depleted (Immune C4) |
| TCGA-19-4065-01 | Lymphocyte Depleted (Immune C4) |
| TCGA-DI-A2QT-01 | IFN-gamma Dominant (Immune C2) |
| TCGA-67-6216-01 | Inflammatory (Immune C3) |
| TCGA-32-2638-01 | Lymphocyte Depleted (Immune C4) |
| TCGA-X3-A8G4-01 | IFN-gamma Dominant (Immune C2) |
| TCGA-F5-6861-01 | Wound Healing (Immune C1) |
| TCGA-A8-A091-01 | Inflammatory (Immune C3) |
| TCGA-EB-A5SF-01 | Inflammatory (Immune C3) |
| TCGA-EB-A82C-01 | Wound Healing (Immune C1) |
| TCGA-CV-A460-01 | IFN-gamma Dominant (Immune C2) |
| TCGA-D8-A1XT-01 | IFN-gamma Dominant (Immune C2) |
| TCGA-AO-A0JL-01 | Wound Healing (Immune C1) |
| TCGA-FG-7643-01 | Immunologically Quiet (Immune C5) |
| TCGA-F5-6814-01 | IFN-gamma Dominant (Immune C2) |
| TCGA-E2-A15T-01 | Inflammatory (Immune C3) |
| TCGA-EL-A3CT-01 | Inflammatory (Immune C3) |
| TCGA-HC-7077-01 | Lymphocyte Depleted (Immune C4) |
| TCGA-G2-AA3B-01 | IFN-gamma Dominant (Immune C2) |
| TCGA-CH-5769-01 | Wound Healing (Immune C1) |
| TCGA-BH-A0DG-01 | IFN-gamma Dominant (Immune C2) |
| TCGA-G9-6499-01 | Inflammatory (Immune C3) |
| TCGA-VD-A8KK-01 | Inflammatory (Immune C3) |
| TCGA-P3-A6T6-01 | Wound Healing (Immune C1) |
| TCGA-G9-6365-01 | Wound Healing (Immune C1) |
| TCGA-GU-AATP-01 | IFN-gamma Dominant (Immune C2) |
| TCGA-G9-7510-01 | Inflammatory (Immune C3) |
| TCGA-AP-A1DM-01 | Wound Healing (Immune C1) |
| TCGA-RW-A686-01 | Inflammatory (Immune C3) |
| TCGA-QF-A5YS-01 | IFN-gamma Dominant (Immune C2) |
| TCGA-4H-AAAK-01 | Wound Healing (Immune C1) |
| TCGA-C5-A1BK-01 | IFN-gamma Dominant (Immune C2) |
| TCGA-BS-A0UM-01 | IFN-gamma Dominant (Immune C2) |
| TCGA-D7-A4YT-01 | Wound Healing (Immune C1) |
| TCGA-UC-A7PD-01 | IFN-gamma Dominant (Immune C2) |
| TCGA-MY-A5BD-01 | Wound Healing (Immune C1) |
| TCGA-J4-AAU2-01 | Inflammatory (Immune C3) |
| TCGA-E9-A22G-01 | IFN-gamma Dominant (Immune C2) |
| TCGA-44-A47G-01 | Inflammatory (Immune C3) |
| TCGA-BR-8589-01 | IFN-gamma Dominant (Immune C2) |
| TCGA-A8-A08P-01 | IFN-gamma Dominant (Immune C2) |
| TCGA-B0-5695-01 | Inflammatory (Immune C3) |
| TCGA-D8-A1X8-01 | Inflammatory (Immune C3) |
| TCGA-DX-A3U6-01 | Inflammatory (Immune C3) |
| TCGA-VS-A9UZ-01 | IFN-gamma Dominant (Immune C2) |
| TCGA-KP-A3VZ-01 | IFN-gamma Dominant (Immune C2) |
| TCGA-2G-AAGA-01 | Wound Healing (Immune C1) |
| TCGA-CQ-5329-01 | IFN-gamma Dominant (Immune C2) |
| TCGA-C8-A1HM-01 | IFN-gamma Dominant (Immune C2) |
| TCGA-EL-A3ZP-01 | Inflammatory (Immune C3) |
| TCGA-CN-6020-01 | IFN-gamma Dominant (Immune C2) |
| TCGA-2Y-A9HA-01 | Lymphocyte Depleted (Immune C4) |
| TCGA-DJ-A2QB-01 | Inflammatory (Immune C3) |
| TCGA-FD-A43Y-01 | IFN-gamma Dominant (Immune C2) |
| TCGA-WC-AA9E-01 | Lymphocyte Depleted (Immune C4) |
| TCGA-BH-A0E1-01 | Wound Healing (Immune C1) |
| TCGA-B9-A8YI-01 | Lymphocyte Depleted (Immune C4) |
| TCGA-TM-A84C-01 | Lymphocyte Depleted (Immune C4) |
| TCGA-02-2485-01 | Lymphocyte Depleted (Immune C4) |
| TCGA-S3-A6ZG-01 | Inflammatory (Immune C3) |
| TCGA-EO-A3B0-01 | Wound Healing (Immune C1) |
| TCGA-AO-A0J5-01 | Wound Healing (Immune C1) |
| TCGA-A2-A0EM-01 | Wound Healing (Immune C1) |
| TCGA-CH-5750-01 | Inflammatory (Immune C3) |
| TCGA-A8-A08T-01 | Wound Healing (Immune C1) |
| TCGA-CK-4948-01 | Wound Healing (Immune C1) |
| TCGA-WY-A85E-01 | Immunologically Quiet (Immune C5) |
| TCGA-AA-3975-01 | Wound Healing (Immune C1) |
| TCGA-12-1597-01 | Lymphocyte Depleted (Immune C4) |
| TCGA-24-1427-01 | Wound Healing (Immune C1) |
| TCGA-A6-6651-01 | Wound Healing (Immune C1) |
| TCGA-XJ-A83H-01 | Inflammatory (Immune C3) |
| TCGA-A1-A0SD-01 | IFN-gamma Dominant (Immune C2) |
| TCGA-QR-A70E-01 | Inflammatory (Immune C3) |
| TCGA-ZG-A8QY-01 | Inflammatory (Immune C3) |
| TCGA-VD-A8KL-01 | Lymphocyte Depleted (Immune C4) |
| TCGA-28-1747-01 | Lymphocyte Depleted (Immune C4) |
| TCGA-EA-A5O9-01 | IFN-gamma Dominant (Immune C2) |
| TCGA-28-2499-01 | Lymphocyte Depleted (Immune C4) |
| TCGA-CH-5791-01 | Inflammatory (Immune C3) |
| TCGA-P8-A5KD-01 | Lymphocyte Depleted (Immune C4) |
| TCGA-55-7816-01 | Inflammatory (Immune C3) |
| TCGA-OR-A5J8-01 | TGF-beta Dominant (Immune C6) |
| TCGA-FY-A40K-01 | Inflammatory (Immune C3) |
| TCGA-DD-AACL-01 | IFN-gamma Dominant (Immune C2) |
| TCGA-TS-A8AI-01 | Wound Healing (Immune C1) |
| TCGA-77-8148-01 | Wound Healing (Immune C1) |
| TCGA-AA-3949-01 | IFN-gamma Dominant (Immune C2) |
| TCGA-AO-A12A-01 | Wound Healing (Immune C1) |
| TCGA-D5-6926-01 | Wound Healing (Immune C1) |
| TCGA-SR-A6MY-01 | Lymphocyte Depleted (Immune C4) |
| TCGA-A2-A0YM-01 | IFN-gamma Dominant (Immune C2) |
| TCGA-BH-A0DP-01 | Inflammatory (Immune C3) |
| TCGA-IB-A6UG-01 | Inflammatory (Immune C3) |
| TCGA-DD-A4NP-01 | Lymphocyte Depleted (Immune C4) |
| TCGA-OR-A5JQ-01 | Inflammatory (Immune C3) |
| TCGA-DJ-A2QA-01 | Inflammatory (Immune C3) |
| TCGA-G3-A5SI-01 | Lymphocyte Depleted (Immune C4) |
| TCGA-IB-7887-01 | Wound Healing (Immune C1) |
| TCGA-VQ-AA6D-01 | IFN-gamma Dominant (Immune C2) |
| TCGA-DD-A113-01 | Inflammatory (Immune C3) |
| TCGA-DY-A1DF-01 | Wound Healing (Immune C1) |
| TCGA-B0-4706-01 | IFN-gamma Dominant (Immune C2) |
| TCGA-ZF-A9RE-01 | IFN-gamma Dominant (Immune C2) |
| TCGA-OR-A5L5-01 | Inflammatory (Immune C3) |
| TCGA-D7-A6EZ-01 | IFN-gamma Dominant (Immune C2) |
| TCGA-B5-A1MX-01 | IFN-gamma Dominant (Immune C2) |
| TCGA-DU-7298-01 | Immunologically Quiet (Immune C5) |
| TCGA-E2-A9RU-01 | IFN-gamma Dominant (Immune C2) |
| TCGA-AX-A2H5-01 | Wound Healing (Immune C1) |
| TCGA-YL-A9WY-01 | Wound Healing (Immune C1) |
| TCGA-C8-A130-01 | IFN-gamma Dominant (Immune C2) |
| TCGA-AY-4070-01 | Wound Healing (Immune C1) |
| TCGA-61-2088-01 | Inflammatory (Immune C3) |
| TCGA-TM-A84T-01 | Immunologically Quiet (Immune C5) |
| TCGA-AA-3552-01 | IFN-gamma Dominant (Immune C2) |
| TCGA-78-7633-01 | Inflammatory (Immune C3) |
| TCGA-C8-A1HI-01 | IFN-gamma Dominant (Immune C2) |
| TCGA-W5-AA33-01 | Inflammatory (Immune C3) |
| TCGA-DV-5565-01 | Inflammatory (Immune C3) |
| TCGA-BW-A5NQ-01 | IFN-gamma Dominant (Immune C2) |
| TCGA-J4-A83M-01 | Inflammatory (Immune C3) |
| TCGA-CN-6024-01 | Wound Healing (Immune C1) |
| TCGA-E3-A3E3-01 | Inflammatory (Immune C3) |
| TCGA-LL-A6FR-01 | IFN-gamma Dominant (Immune C2) |
| TCGA-HC-7078-01 | Inflammatory (Immune C3) |
| TCGA-DJ-A1QM-01 | Inflammatory (Immune C3) |
| TCGA-A4-A4ZT-01 | Inflammatory (Immune C3) |
| TCGA-OR-A5J3-01 | Lymphocyte Depleted (Immune C4) |
| TCGA-DD-A1EA-01 | Inflammatory (Immune C3) |
| TCGA-P5-A5EU-01 | Lymphocyte Depleted (Immune C4) |
| TCGA-60-2695-01 | IFN-gamma Dominant (Immune C2) |
| TCGA-FG-7637-01 | Lymphocyte Depleted (Immune C4) |
| TCGA-DD-AAW2-01 | Lymphocyte Depleted (Immune C4) |
| TCGA-A8-A07E-01 | Wound Healing (Immune C1) |
| TCGA-CV-A6K1-01 | IFN-gamma Dominant (Immune C2) |
| TCGA-25-1313-01 | Lymphocyte Depleted (Immune C4) |
| TCGA-E9-A247-01 | IFN-gamma Dominant (Immune C2) |
| TCGA-2G-AAGJ-01 | IFN-gamma Dominant (Immune C2) |
| TCGA-VQ-A924-01 | IFN-gamma Dominant (Immune C2) |
| TCGA-B6-A1KI-01 | Inflammatory (Immune C3) |
| TCGA-F4-6856-01 | Wound Healing (Immune C1) |
| TCGA-RM-A68W-01 | Inflammatory (Immune C3) |
| TCGA-86-A4P8-01 | Inflammatory (Immune C3) |
| TCGA-DF-A2KN-01 | IFN-gamma Dominant (Immune C2) |
| TCGA-78-7535-01 | Lymphocyte Depleted (Immune C4) |
| TCGA-86-8671-01 | Inflammatory (Immune C3) |
| TCGA-4A-A93W-01 | Inflammatory (Immune C3) |
| TCGA-F4-6569-01 | Wound Healing (Immune C1) |
| TCGA-AG-3581-01 | Wound Healing (Immune C1) |
| TCGA-B2-5641-01 | Inflammatory (Immune C3) |
| TCGA-18-3421-01 | IFN-gamma Dominant (Immune C2) |
| TCGA-HT-A614-01 | Immunologically Quiet (Immune C5) |
| TCGA-06-0649-01 | Lymphocyte Depleted (Immune C4) |
| TCGA-C8-A1HF-01 | IFN-gamma Dominant (Immune C2) |
| TCGA-A3-3382-01 | Inflammatory (Immune C3) |
| TCGA-VG-A8LO-01 | IFN-gamma Dominant (Immune C2) |
| TCGA-KN-8428-01 | Wound Healing (Immune C1) |
| TCGA-78-7153-01 | Inflammatory (Immune C3) |
| TCGA-2F-A9KW-01 | Lymphocyte Depleted (Immune C4) |
| TCGA-A2-A3XZ-01 | IFN-gamma Dominant (Immune C2) |
| TCGA-29-1690-01 | Lymphocyte Depleted (Immune C4) |
| TCGA-DK-A1AB-01 | IFN-gamma Dominant (Immune C2) |
| TCGA-KK-A7B1-01 | Wound Healing (Immune C1) |
| TCGA-CD-A487-01 | IFN-gamma Dominant (Immune C2) |
| TCGA-2G-AAGN-01 | IFN-gamma Dominant (Immune C2) |
| TCGA-MP-A4TK-01 | Wound Healing (Immune C1) |
| TCGA-DU-A7TG-01 | Immunologically Quiet (Immune C5) |
| TCGA-D1-A1NY-01 | IFN-gamma Dominant (Immune C2) |
| TCGA-AX-A3FX-01 | Wound Healing (Immune C1) |
| TCGA-C5-A8XH-01 | IFN-gamma Dominant (Immune C2) |
| TCGA-CZ-5454-01 | Lymphocyte Depleted (Immune C4) |
| TCGA-91-6835-01 | Inflammatory (Immune C3) |
| TCGA-B5-A1MS-01 | Wound Healing (Immune C1) |
| TCGA-A8-A07F-01 | Inflammatory (Immune C3) |
| TCGA-EM-A4FR-01 | Inflammatory (Immune C3) |
| TCGA-FG-A60J-01 | Immunologically Quiet (Immune C5) |
| TCGA-WB-A80V-01 | Lymphocyte Depleted (Immune C4) |
| TCGA-2J-AABH-01 | TGF-beta Dominant (Immune C6) |
| TCGA-B6-A0RN-01 | Inflammatory (Immune C3) |
| TCGA-UP-A6WW-01 | Wound Healing (Immune C1) |
| TCGA-DU-7300-01 | Immunologically Quiet (Immune C5) |
| TCGA-D1-A15W-01 | Wound Healing (Immune C1) |
| TCGA-EI-6514-01 | Wound Healing (Immune C1) |
| TCGA-J4-A83I-01 | Inflammatory (Immune C3) |
| TCGA-OY-A56Q-01 | Lymphocyte Depleted (Immune C4) |
| TCGA-GC-A3BM-01 | Wound Healing (Immune C1) |
| TCGA-D5-6535-01 | Wound Healing (Immune C1) |
| TCGA-CG-4472-01 | Inflammatory (Immune C3) |
| TCGA-ET-A3BQ-01 | Inflammatory (Immune C3) |
| TCGA-FG-8189-01 | Immunologically Quiet (Immune C5) |
| TCGA-56-6546-01 | IFN-gamma Dominant (Immune C2) |
| TCGA-FD-A5BV-01 | Wound Healing (Immune C1) |
| TCGA-N6-A4VF-01 | Wound Healing (Immune C1) |
| TCGA-43-A56V-01 | Wound Healing (Immune C1) |
| TCGA-B5-A0K3-01 | IFN-gamma Dominant (Immune C2) |
| TCGA-P5-A5EY-01 | Immunologically Quiet (Immune C5) |
| TCGA-EL-A3MW-01 | Inflammatory (Immune C3) |
| TCGA-CV-5978-01 | IFN-gamma Dominant (Immune C2) |
| TCGA-KM-8438-01 | Inflammatory (Immune C3) |
| TCGA-3H-AB3X-01 | Inflammatory (Immune C3) |
| TCGA-25-1317-01 | Wound Healing (Immune C1) |
| TCGA-39-5022-01 | IFN-gamma Dominant (Immune C2) |
| TCGA-AX-A1CC-01 | IFN-gamma Dominant (Immune C2) |
| TCGA-06-2564-01 | Lymphocyte Depleted (Immune C4) |
| TCGA-XK-AAIV-01 | IFN-gamma Dominant (Immune C2) |
| TCGA-DW-7839-01 | Lymphocyte Depleted (Immune C4) |
| TCGA-22-5479-01 | Wound Healing (Immune C1) |
| TCGA-IQ-A6SH-01 | IFN-gamma Dominant (Immune C2) |
| TCGA-BP-5178-01 | Inflammatory (Immune C3) |
| TCGA-LD-A7W5-01 | IFN-gamma Dominant (Immune C2) |
| TCGA-EL-A3CW-01 | Inflammatory (Immune C3) |
| TCGA-ZP-A9CZ-01 | IFN-gamma Dominant (Immune C2) |
| TCGA-UY-A8OC-01 | Wound Healing (Immune C1) |
| TCGA-55-6980-01 | TGF-beta Dominant (Immune C6) |
| TCGA-FI-A2F9-01 | Wound Healing (Immune C1) |
| TCGA-AA-3526-01 | IFN-gamma Dominant (Immune C2) |
| TCGA-JX-A3Q8-01 | IFN-gamma Dominant (Immune C2) |
| TCGA-56-A4BY-01 | Wound Healing (Immune C1) |
| TCGA-3U-A98D-01 | Wound Healing (Immune C1) |
| TCGA-HW-7486-01 | Immunologically Quiet (Immune C5) |
| TCGA-BK-A56F-01 | Wound Healing (Immune C1) |
| TCGA-BR-8361-01 | IFN-gamma Dominant (Immune C2) |
| TCGA-BH-A0BL-01 | Wound Healing (Immune C1) |
| TCGA-37-4132-01 | IFN-gamma Dominant (Immune C2) |
| TCGA-27-1832-01 | Lymphocyte Depleted (Immune C4) |
| TCGA-DE-A4M8-01 | Inflammatory (Immune C3) |
| TCGA-50-5936-01 | Wound Healing (Immune C1) |
| TCGA-AJ-A3OL-01 | IFN-gamma Dominant (Immune C2) |
| TCGA-BJ-A0Z9-01 | Lymphocyte Depleted (Immune C4) |
| TCGA-G7-7502-01 | Inflammatory (Immune C3) |
| TCGA-BR-8680-01 | Wound Healing (Immune C1) |
| TCGA-FD-A3SS-01 | IFN-gamma Dominant (Immune C2) |
| TCGA-AN-A0XV-01 | Wound Healing (Immune C1) |
| TCGA-AA-3680-01 | Inflammatory (Immune C3) |
| TCGA-RD-A8N5-01 | TGF-beta Dominant (Immune C6) |
| TCGA-AC-A2FE-01 | TGF-beta Dominant (Immune C6) |
| TCGA-DF-A2KZ-01 | IFN-gamma Dominant (Immune C2) |
| TCGA-XV-AAZV-01 | TGF-beta Dominant (Immune C6) |
| TCGA-BJ-A0ZH-01 | Inflammatory (Immune C3) |
| TCGA-14-1829-01 | Lymphocyte Depleted (Immune C4) |
| TCGA-WB-A80L-01 | Inflammatory (Immune C3) |
| TCGA-EL-A3H3-01 | Inflammatory (Immune C3) |
| TCGA-ET-A3BX-01 | Inflammatory (Immune C3) |
| TCGA-B0-5812-01 | Inflammatory (Immune C3) |
| TCGA-VN-A88P-01 | Inflammatory (Immune C3) |
| TCGA-A7-A4SB-01 | Inflammatory (Immune C3) |
| TCGA-CQ-A4CD-01 | IFN-gamma Dominant (Immune C2) |
| TCGA-FD-A5BR-01 | Wound Healing (Immune C1) |
| TCGA-AD-6895-01 | IFN-gamma Dominant (Immune C2) |
| TCGA-EJ-5505-01 | Inflammatory (Immune C3) |
| TCGA-D7-5578-01 | Wound Healing (Immune C1) |
| TCGA-32-2615-01 | Lymphocyte Depleted (Immune C4) |
| TCGA-BC-4072-01 | Inflammatory (Immune C3) |
| TCGA-AR-A2LQ-01 | Inflammatory (Immune C3) |
| TCGA-OR-A5LS-01 | Lymphocyte Depleted (Immune C4) |
| TCGA-AH-6544-01 | Wound Healing (Immune C1) |
| TCGA-EB-A97M-01 | Inflammatory (Immune C3) |
| TCGA-CR-7404-01 | IFN-gamma Dominant (Immune C2) |
| TCGA-F9-A7Q0-01 | Inflammatory (Immune C3) |
| TCGA-AN-A0XU-01 | Wound Healing (Immune C1) |
| TCGA-BG-A18B-01 | Wound Healing (Immune C1) |
| TCGA-A7-A6VV-01 | Wound Healing (Immune C1) |
| TCGA-AO-A03P-01 | IFN-gamma Dominant (Immune C2) |
| TCGA-18-3415-01 | Wound Healing (Immune C1) |
| TCGA-G9-6351-01 | Inflammatory (Immune C3) |
| TCGA-B4-5835-01 | Lymphocyte Depleted (Immune C4) |
| TCGA-06-0158-01 | Lymphocyte Depleted (Immune C4) |
| TCGA-CC-A3MC-01 | Inflammatory (Immune C3) |
| TCGA-X2-A95T-01 | Wound Healing (Immune C1) |
| TCGA-CF-A8HY-01 | Wound Healing (Immune C1) |
| TCGA-QQ-A8VH-01 | Wound Healing (Immune C1) |
| TCGA-BQ-5893-01 | Inflammatory (Immune C3) |
| TCGA-DS-A7WI-01 | IFN-gamma Dominant (Immune C2) |
| TCGA-AC-A3YI-01 | Wound Healing (Immune C1) |
| TCGA-61-2104-01 | IFN-gamma Dominant (Immune C2) |
| TCGA-73-4675-01 | Inflammatory (Immune C3) |
| TCGA-A2-A0EP-01 | Inflammatory (Immune C3) |
| TCGA-B2-4099-01 | Lymphocyte Depleted (Immune C4) |
| TCGA-KK-A8IK-01 | Lymphocyte Depleted (Immune C4) |
| TCGA-50-5932-01 | Inflammatory (Immune C3) |
| TCGA-A2-A04X-01 | IFN-gamma Dominant (Immune C2) |
| TCGA-X6-A8C3-01 | Lymphocyte Depleted (Immune C4) |
| TCGA-BJ-A0Z0-01 | Inflammatory (Immune C3) |
| TCGA-CV-7406-01 | Wound Healing (Immune C1) |
| TCGA-E2-A1LI-01 | IFN-gamma Dominant (Immune C2) |
| TCGA-GD-A76B-01 | IFN-gamma Dominant (Immune C2) |
| TCGA-DJ-A13O-01 | Inflammatory (Immune C3) |
| TCGA-DK-AA71-01 | Wound Healing (Immune C1) |
| TCGA-B6-A0X0-01 | Inflammatory (Immune C3) |
| TCGA-EM-A2OX-01 | Inflammatory (Immune C3) |
| TCGA-93-A4JO-01 | Inflammatory (Immune C3) |
| TCGA-CN-5360-01 | IFN-gamma Dominant (Immune C2) |
| TCGA-OL-A66L-01 | Wound Healing (Immune C1) |
| TCGA-B6-A0WS-01 | IFN-gamma Dominant (Immune C2) |
| TCGA-F7-A623-01 | IFN-gamma Dominant (Immune C2) |
| TCGA-C8-A138-01 | IFN-gamma Dominant (Immune C2) |
| TCGA-IE-A4EI-01 | Lymphocyte Depleted (Immune C4) |
| TCGA-AA-A03F-01 | Inflammatory (Immune C3) |
| TCGA-24-2026-01 | IFN-gamma Dominant (Immune C2) |
| TCGA-VS-A953-01 | IFN-gamma Dominant (Immune C2) |
| TCGA-IM-A41Z-01 | Inflammatory (Immune C3) |
| TCGA-AA-A02F-01 | Wound Healing (Immune C1) |
| TCGA-55-8510-01 | Inflammatory (Immune C3) |
| TCGA-G6-A8L6-01 | Inflammatory (Immune C3) |
| TCGA-DD-AAW1-01 | Lymphocyte Depleted (Immune C4) |
| TCGA-06-2558-01 | Lymphocyte Depleted (Immune C4) |
| TCGA-W4-A7U2-01 | IFN-gamma Dominant (Immune C2) |
| TCGA-A7-A56D-01 | IFN-gamma Dominant (Immune C2) |
| TCGA-B8-4146-01 | Lymphocyte Depleted (Immune C4) |
| TCGA-EJ-A6RC-01 | Inflammatory (Immune C3) |
| TCGA-HT-8011-01 | Lymphocyte Depleted (Immune C4) |
| TCGA-AF-6672-01 | Wound Healing (Immune C1) |
| TCGA-4Z-AA7R-01 | Wound Healing (Immune C1) |
| TCGA-HT-8106-01 | Inflammatory (Immune C3) |
| TCGA-O2-A52V-01 | Wound Healing (Immune C1) |
| TCGA-FK-A4UB-01 | Inflammatory (Immune C3) |
| TCGA-WB-A814-01 | Inflammatory (Immune C3) |
| TCGA-ZF-AA58-01 | IFN-gamma Dominant (Immune C2) |
| TCGA-22-1005-01 | TGF-beta Dominant (Immune C6) |
| TCGA-AX-A3G6-01 | IFN-gamma Dominant (Immune C2) |
| TCGA-A8-A07B-01 | Wound Healing (Immune C1) |
| TCGA-ZD-A8I3-01 | Wound Healing (Immune C1) |
| TCGA-BH-A0H3-01 | Inflammatory (Immune C3) |
| TCGA-A8-A086-01 | Wound Healing (Immune C1) |
| TCGA-BR-4292-01 | IFN-gamma Dominant (Immune C2) |
| TCGA-VD-A8K7-01 | Lymphocyte Depleted (Immune C4) |
| TCGA-49-AAR3-01 | IFN-gamma Dominant (Immune C2) |
| TCGA-EM-A3SX-01 | Inflammatory (Immune C3) |
| TCGA-78-7154-01 | Wound Healing (Immune C1) |
| TCGA-DX-A6BE-01 | Inflammatory (Immune C3) |
| TCGA-24-1564-01 | Lymphocyte Depleted (Immune C4) |
| TCGA-AR-A1AR-01 | IFN-gamma Dominant (Immune C2) |
| TCGA-QR-A6GX-01 | Inflammatory (Immune C3) |
| TCGA-13-0897-01 | IFN-gamma Dominant (Immune C2) |
| TCGA-EA-A44S-01 | IFN-gamma Dominant (Immune C2) |
| TCGA-BH-A1FU-01 | Wound Healing (Immune C1) |
| TCGA-YL-A8SL-01 | Wound Healing (Immune C1) |
| TCGA-W5-AA2I-01 | Inflammatory (Immune C3) |
| TCGA-CQ-5334-01 | IFN-gamma Dominant (Immune C2) |
| TCGA-M7-A720-01 | Inflammatory (Immune C3) |
| TCGA-HC-7080-01 | Inflammatory (Immune C3) |
| TCGA-QK-A8ZA-01 | IFN-gamma Dominant (Immune C2) |
| TCGA-85-8288-01 | Wound Healing (Immune C1) |
| TCGA-B6-A0RI-01 | Wound Healing (Immune C1) |
| TCGA-B0-4846-01 | Inflammatory (Immune C3) |
| TCGA-XK-AAJU-01 | Inflammatory (Immune C3) |
| TCGA-YU-AA4L-01 | Lymphocyte Depleted (Immune C4) |
| TCGA-85-8351-01 | Wound Healing (Immune C1) |
| TCGA-E2-A1B6-01 | IFN-gamma Dominant (Immune C2) |
| TCGA-EI-7002-01 | Wound Healing (Immune C1) |
| TCGA-E9-A1N4-01 | TGF-beta Dominant (Immune C6) |
| TCGA-OR-A5LH-01 | Lymphocyte Depleted (Immune C4) |
| TCGA-CR-7367-01 | Wound Healing (Immune C1) |
| TCGA-K4-A6MB-01 | Wound Healing (Immune C1) |
| TCGA-VR-AA7B-01 | Wound Healing (Immune C1) |
| TCGA-BQ-5889-01 | Inflammatory (Immune C3) |
| TCGA-VS-A957-01 | IFN-gamma Dominant (Immune C2) |
| TCGA-EL-A3CS-01 | Inflammatory (Immune C3) |
| TCGA-EJ-5531-01 | Inflammatory (Immune C3) |
| TCGA-XR-A8TE-01 | Inflammatory (Immune C3) |
| TCGA-PG-A915-01 | IFN-gamma Dominant (Immune C2) |
| TCGA-BR-7716-01 | IFN-gamma Dominant (Immune C2) |
| TCGA-28-5216-01 | Lymphocyte Depleted (Immune C4) |
| TCGA-85-8277-01 | Wound Healing (Immune C1) |
| TCGA-VQ-A8DZ-01 | Wound Healing (Immune C1) |
| TCGA-A7-A6VY-01 | IFN-gamma Dominant (Immune C2) |
| TCGA-77-8143-01 | Wound Healing (Immune C1) |
| TCGA-DK-A6B5-01 | IFN-gamma Dominant (Immune C2) |
| TCGA-T7-A92I-01 | Inflammatory (Immune C3) |
| TCGA-XF-A9SI-01 | IFN-gamma Dominant (Immune C2) |
| TCGA-FK-A3SB-01 | Inflammatory (Immune C3) |
| TCGA-XF-A9SJ-01 | IFN-gamma Dominant (Immune C2) |
| TCGA-WY-A859-01 | Immunologically Quiet (Immune C5) |
| TCGA-QK-A6IF-01 | IFN-gamma Dominant (Immune C2) |
| TCGA-E8-A434-01 | Inflammatory (Immune C3) |
| TCGA-B5-A0K0-01 | Wound Healing (Immune C1) |
| TCGA-J4-8198-01 | Inflammatory (Immune C3) |
| TCGA-DX-A2IZ-01 | Lymphocyte Depleted (Immune C4) |
| TCGA-EB-A44O-01 | Wound Healing (Immune C1) |
| TCGA-B9-A44B-01 | Inflammatory (Immune C3) |
| TCGA-AA-A02R-01 | IFN-gamma Dominant (Immune C2) |
| TCGA-DF-A2KV-01 | IFN-gamma Dominant (Immune C2) |
| TCGA-E7-A8O8-01 | Wound Healing (Immune C1) |
| TCGA-AG-3725-01 | Wound Healing (Immune C1) |
| TCGA-HN-A2OB-01 | Inflammatory (Immune C3) |
| TCGA-D8-A141-01 | TGF-beta Dominant (Immune C6) |
| TCGA-KL-8344-01 | Inflammatory (Immune C3) |
| TCGA-AA-3976-01 | Wound Healing (Immune C1) |
| TCGA-CZ-4862-01 | Inflammatory (Immune C3) |
| TCGA-22-4605-01 | Wound Healing (Immune C1) |
| TCGA-CR-6487-01 | IFN-gamma Dominant (Immune C2) |
| TCGA-4B-A93V-01 | Wound Healing (Immune C1) |
| TCGA-D3-A5GT-01 | Lymphocyte Depleted (Immune C4) |
| TCGA-WY-A85B-01 | Immunologically Quiet (Immune C5) |
| TCGA-BH-A18T-01 | Wound Healing (Immune C1) |
| TCGA-B0-5701-01 | Inflammatory (Immune C3) |
| TCGA-JW-A5VH-01 | Wound Healing (Immune C1) |
| TCGA-CM-6169-01 | IFN-gamma Dominant (Immune C2) |
| TCGA-A5-A1OJ-01 | Inflammatory (Immune C3) |
| TCGA-EY-A72D-01 | Wound Healing (Immune C1) |
| TCGA-RD-A7BT-01 | Wound Healing (Immune C1) |
| TCGA-CR-6493-01 | IFN-gamma Dominant (Immune C2) |
| TCGA-FC-7708-01 | Inflammatory (Immune C3) |
| TCGA-OR-A5JL-01 | Lymphocyte Depleted (Immune C4) |
| TCGA-S9-A6UB-01 | Immunologically Quiet (Immune C5) |
| TCGA-BH-A0BD-01 | IFN-gamma Dominant (Immune C2) |
| TCGA-2G-AAL7-01 | IFN-gamma Dominant (Immune C2) |
| TCGA-CV-6954-01 | IFN-gamma Dominant (Immune C2) |
| TCGA-OL-A5RU-01 | Inflammatory (Immune C3) |
| TCGA-QK-A6II-01 | IFN-gamma Dominant (Immune C2) |
| TCGA-C8-A12T-01 | Wound Healing (Immune C1) |
| TCGA-18-3411-01 | Wound Healing (Immune C1) |
| TCGA-DX-A3U9-01 | Wound Healing (Immune C1) |
| TCGA-24-1558-01 | Lymphocyte Depleted (Immune C4) |
| TCGA-BA-5558-01 | IFN-gamma Dominant (Immune C2) |
| TCGA-IB-7897-01 | Inflammatory (Immune C3) |
| TCGA-YL-A8S8-01 | Lymphocyte Depleted (Immune C4) |
| TCGA-NA-A4QY-01 | Wound Healing (Immune C1) |
| TCGA-29-1711-01 | IFN-gamma Dominant (Immune C2) |
| TCGA-MP-A4SY-01 | Wound Healing (Immune C1) |
| TCGA-AG-3892-01 | IFN-gamma Dominant (Immune C2) |
| TCGA-A6-6650-01 | Wound Healing (Immune C1) |
| TCGA-A7-A0DA-01 | Wound Healing (Immune C1) |
| TCGA-E8-A2EA-01 | Inflammatory (Immune C3) |
| TCGA-75-7030-01 | TGF-beta Dominant (Immune C6) |
| TCGA-A5-A0GV-01 | Wound Healing (Immune C1) |
| TCGA-UT-A97Y-01 | Wound Healing (Immune C1) |
| TCGA-28-5207-01 | Lymphocyte Depleted (Immune C4) |
| TCGA-28-5208-01 | Lymphocyte Depleted (Immune C4) |
| TCGA-HZ-8315-01 | TGF-beta Dominant (Immune C6) |
| TCGA-V1-A9ZG-01 | Wound Healing (Immune C1) |
| TCGA-KN-8418-01 | Inflammatory (Immune C3) |
| TCGA-FY-A4B0-01 | Inflammatory (Immune C3) |
| TCGA-PG-A7D5-01 | IFN-gamma Dominant (Immune C2) |
| TCGA-BF-A5EP-01 | Wound Healing (Immune C1) |
| TCGA-D7-8573-01 | Wound Healing (Immune C1) |
| TCGA-HU-A4H5-01 | Wound Healing (Immune C1) |
| TCGA-S9-A6U6-01 | Immunologically Quiet (Immune C5) |
| TCGA-UY-A78M-01 | Wound Healing (Immune C1) |
| TCGA-E2-A14V-01 | Wound Healing (Immune C1) |
| TCGA-E7-A8O7-01 | Lymphocyte Depleted (Immune C4) |
| TCGA-BP-4170-01 | Inflammatory (Immune C3) |
| TCGA-CV-6942-01 | IFN-gamma Dominant (Immune C2) |
| TCGA-AA-3941-01 | Wound Healing (Immune C1) |
| TCGA-33-4583-01 | IFN-gamma Dominant (Immune C2) |
| TCGA-CV-5441-01 | Wound Healing (Immune C1) |
| TCGA-DU-8168-01 | Immunologically Quiet (Immune C5) |
| TCGA-KK-A8IH-01 | Inflammatory (Immune C3) |
| TCGA-HV-A5A5-01 | TGF-beta Dominant (Immune C6) |
| TCGA-ZG-A9LN-01 | IFN-gamma Dominant (Immune C2) |
| TCGA-BR-4362-01 | IFN-gamma Dominant (Immune C2) |
| TCGA-HC-A632-01 | Inflammatory (Immune C3) |
| TCGA-85-8355-01 | IFN-gamma Dominant (Immune C2) |
| TCGA-MI-A75I-01 | Lymphocyte Depleted (Immune C4) |
| TCGA-06-0130-01 | Lymphocyte Depleted (Immune C4) |
| TCGA-EL-A3ZN-01 | Inflammatory (Immune C3) |
| TCGA-XF-AAN0-01 | Wound Healing (Immune C1) |
| TCGA-DU-7290-01 | Immunologically Quiet (Immune C5) |
| TCGA-EA-A3HR-01 | Wound Healing (Immune C1) |
| TCGA-IE-A3OV-01 | Inflammatory (Immune C3) |
| TCGA-B8-A8YJ-01 | Inflammatory (Immune C3) |
| TCGA-G9-6353-01 | Inflammatory (Immune C3) |
| TCGA-3B-A9HQ-01 | IFN-gamma Dominant (Immune C2) |
| TCGA-D1-A177-01 | Wound Healing (Immune C1) |
| TCGA-AJ-A2QK-01 | IFN-gamma Dominant (Immune C2) |
| TCGA-BH-A0DV-01 | Inflammatory (Immune C3) |
| TCGA-AA-A02E-01 | Wound Healing (Immune C1) |
| TCGA-KK-A59Z-01 | Inflammatory (Immune C3) |
| TCGA-CR-7394-01 | IFN-gamma Dominant (Immune C2) |
| TCGA-MZ-A6I9-01 | Wound Healing (Immune C1) |
| TCGA-BR-7197-01 | Wound Healing (Immune C1) |
| TCGA-B0-4849-01 | Inflammatory (Immune C3) |
| TCGA-MP-A4SV-01 | IFN-gamma Dominant (Immune C2) |
| TCGA-3H-AB3S-01 | Wound Healing (Immune C1) |
| TCGA-29-1703-01 | IFN-gamma Dominant (Immune C2) |
| TCGA-D1-A162-01 | Wound Healing (Immune C1) |
| TCGA-OR-A5JT-01 | Lymphocyte Depleted (Immune C4) |
| TCGA-B6-A0I8-01 | Inflammatory (Immune C3) |
| TCGA-IG-A3I8-01 | Wound Healing (Immune C1) |
| TCGA-E2-A1L7-01 | IFN-gamma Dominant (Immune C2) |
| TCGA-DJ-A2Q4-01 | Inflammatory (Immune C3) |
| TCGA-55-8513-01 | Inflammatory (Immune C3) |
| TCGA-06-5416-01 | Lymphocyte Depleted (Immune C4) |
| TCGA-OR-A5JG-01 | Lymphocyte Depleted (Immune C4) |
| TCGA-D1-A16N-01 | IFN-gamma Dominant (Immune C2) |
| TCGA-P3-A6T7-01 | IFN-gamma Dominant (Immune C2) |
| TCGA-AX-A05Y-01 | Lymphocyte Depleted (Immune C4) |
| TCGA-FB-AAQ6-01 | Wound Healing (Immune C1) |
| TCGA-56-6545-01 | Wound Healing (Immune C1) |
| TCGA-34-2608-01 | Wound Healing (Immune C1) |
| TCGA-WL-A834-01 | Wound Healing (Immune C1) |
| TCGA-DX-A8BM-01 | Lymphocyte Depleted (Immune C4) |
| TCGA-MP-A4T4-01 | IFN-gamma Dominant (Immune C2) |
| TCGA-71-8520-01 | IFN-gamma Dominant (Immune C2) |
| TCGA-4Z-AA82-01 | IFN-gamma Dominant (Immune C2) |
| TCGA-P3-A5QA-01 | IFN-gamma Dominant (Immune C2) |
| TCGA-CM-6680-01 | Wound Healing (Immune C1) |
| TCGA-44-7672-01 | IFN-gamma Dominant (Immune C2) |
| TCGA-D1-A16Y-01 | Lymphocyte Depleted (Immune C4) |
| TCGA-E9-A1N8-01 | Wound Healing (Immune C1) |
| TCGA-H2-A26U-01 | Inflammatory (Immune C3) |
| TCGA-A8-A085-01 | Lymphocyte Depleted (Immune C4) |
| TCGA-50-6593-01 | Wound Healing (Immune C1) |
| TCGA-AR-A24Q-01 | Wound Healing (Immune C1) |
| TCGA-O2-A5IB-01 | IFN-gamma Dominant (Immune C2) |
| TCGA-AR-A0U0-01 | IFN-gamma Dominant (Immune C2) |
| TCGA-67-3771-01 | IFN-gamma Dominant (Immune C2) |
| TCGA-CV-A45Q-01 | IFN-gamma Dominant (Immune C2) |
| TCGA-AA-3663-01 | Wound Healing (Immune C1) |
| TCGA-22-4613-01 | IFN-gamma Dominant (Immune C2) |
| TCGA-AP-A3K1-01 | Wound Healing (Immune C1) |
| TCGA-A4-8311-01 | Inflammatory (Immune C3) |
| TCGA-3L-AA1B-01 | Wound Healing (Immune C1) |
| TCGA-SX-A7SO-01 | Immunologically Quiet (Immune C5) |
| TCGA-30-1862-01 | Wound Healing (Immune C1) |
| TCGA-EC-A24G-01 | Wound Healing (Immune C1) |
| TCGA-CN-6019-01 | TGF-beta Dominant (Immune C6) |
| TCGA-UF-A7JA-01 | Wound Healing (Immune C1) |
| TCGA-99-8033-01 | Wound Healing (Immune C1) |
| TCGA-B0-4823-01 | Inflammatory (Immune C3) |
| TCGA-EB-A5FP-01 | Wound Healing (Immune C1) |
| TCGA-OR-A5LO-01 | Inflammatory (Immune C3) |
| TCGA-BG-A3PP-01 | Wound Healing (Immune C1) |
| TCGA-KO-8404-01 | Lymphocyte Depleted (Immune C4) |
| TCGA-B5-A0K4-01 | Lymphocyte Depleted (Immune C4) |
| TCGA-3B-A9HU-01 | IFN-gamma Dominant (Immune C2) |
| TCGA-22-4596-01 | TGF-beta Dominant (Immune C6) |
| TCGA-F2-A44H-01 | TGF-beta Dominant (Immune C6) |
| TCGA-AP-A0LM-01 | Inflammatory (Immune C3) |
| TCGA-RE-A7BO-01 | IFN-gamma Dominant (Immune C2) |
| TCGA-A7-A0CE-01 | IFN-gamma Dominant (Immune C2) |
| TCGA-MP-A4T8-01 | IFN-gamma Dominant (Immune C2) |
| TCGA-VQ-A91W-01 | IFN-gamma Dominant (Immune C2) |
| TCGA-V1-A8WL-01 | IFN-gamma Dominant (Immune C2) |
| TCGA-BP-4991-01 | Inflammatory (Immune C3) |
| TCGA-RW-A68C-01 | Inflammatory (Immune C3) |
| TCGA-29-1778-01 | IFN-gamma Dominant (Immune C2) |
| TCGA-FP-7916-01 | IFN-gamma Dominant (Immune C2) |
| TCGA-06-0168-01 | Lymphocyte Depleted (Immune C4) |
| TCGA-OR-A5L3-01 | Immunologically Quiet (Immune C5) |
| TCGA-EB-A3XB-01 | Wound Healing (Immune C1) |
| TCGA-B2-4102-01 | Inflammatory (Immune C3) |
| TCGA-KK-A8IG-01 | Inflammatory (Immune C3) |
| TCGA-C5-A901-01 | IFN-gamma Dominant (Immune C2) |
| TCGA-AK-3434-01 | Inflammatory (Immune C3) |
| TCGA-09-1662-01 | Lymphocyte Depleted (Immune C4) |
| TCGA-HN-A2NL-01 | IFN-gamma Dominant (Immune C2) |
| TCGA-BJ-A291-01 | Inflammatory (Immune C3) |
| TCGA-YL-A8HK-01 | Inflammatory (Immune C3) |
| TCGA-A6-6648-01 | Wound Healing (Immune C1) |
| TCGA-E6-A8L9-01 | IFN-gamma Dominant (Immune C2) |
| TCGA-CZ-4861-01 | Inflammatory (Immune C3) |
| TCGA-34-8455-01 | Wound Healing (Immune C1) |
| TCGA-DU-6407-01 | Immunologically Quiet (Immune C5) |
| TCGA-CV-6950-01 | IFN-gamma Dominant (Immune C2) |
| TCGA-22-5480-01 | IFN-gamma Dominant (Immune C2) |
| TCGA-AA-3856-01 | IFN-gamma Dominant (Immune C2) |
| TCGA-YU-A94D-01 | Wound Healing (Immune C1) |
| TCGA-P3-A5QE-01 | IFN-gamma Dominant (Immune C2) |
| TCGA-77-8128-01 | Wound Healing (Immune C1) |
| TCGA-G2-A2ES-01 | IFN-gamma Dominant (Immune C2) |
| TCGA-CR-6481-01 | IFN-gamma Dominant (Immune C2) |
| TCGA-C8-A134-01 | IFN-gamma Dominant (Immune C2) |
| TCGA-DX-A6BA-01 | Lymphocyte Depleted (Immune C4) |
| TCGA-D7-6815-01 | Wound Healing (Immune C1) |
| TCGA-ZF-A9R7-01 | IFN-gamma Dominant (Immune C2) |
| TCGA-DJ-A2PU-01 | Inflammatory (Immune C3) |
| TCGA-AA-A02J-01 | Wound Healing (Immune C1) |
| TCGA-CV-6441-01 | IFN-gamma Dominant (Immune C2) |
| TCGA-C8-A12Y-01 | IFN-gamma Dominant (Immune C2) |
| TCGA-N5-A4RS-01 | Wound Healing (Immune C1) |
| TCGA-DX-A7ET-01 | IFN-gamma Dominant (Immune C2) |
| TCGA-DJ-A3VL-01 | Inflammatory (Immune C3) |
| TCGA-E7-A4XJ-01 | Wound Healing (Immune C1) |
| TCGA-XF-A8HG-01 | Lymphocyte Depleted (Immune C4) |
| TCGA-29-1694-01 | IFN-gamma Dominant (Immune C2) |
| TCGA-02-0055-01 | Lymphocyte Depleted (Immune C4) |
| TCGA-IB-AAUO-01 | IFN-gamma Dominant (Immune C2) |
| TCGA-ER-A2NF-01 | IFN-gamma Dominant (Immune C2) |
| TCGA-DX-AB3A-01 | Wound Healing (Immune C1) |
| TCGA-N7-A59B-01 | IFN-gamma Dominant (Immune C2) |
| TCGA-AC-A2BM-01 | IFN-gamma Dominant (Immune C2) |
| TCGA-A2-A0EU-01 | Wound Healing (Immune C1) |
| TCGA-C5-A1M6-01 | Wound Healing (Immune C1) |
| TCGA-HT-7601-01 | Immunologically Quiet (Immune C5) |
| TCGA-CR-7382-01 | IFN-gamma Dominant (Immune C2) |
| TCGA-VQ-A8P5-01 | IFN-gamma Dominant (Immune C2) |
| TCGA-N7-A4Y0-01 | IFN-gamma Dominant (Immune C2) |
| TCGA-AG-A02X-01 | Wound Healing (Immune C1) |
| TCGA-BP-4768-01 | Inflammatory (Immune C3) |
| TCGA-DJ-A13L-01 | Inflammatory (Immune C3) |
| TCGA-CJ-5677-01 | TGF-beta Dominant (Immune C6) |
| TCGA-E7-A6ME-01 | IFN-gamma Dominant (Immune C2) |
| TCGA-56-8503-01 | Wound Healing (Immune C1) |
| TCGA-AJ-A3BF-01 | IFN-gamma Dominant (Immune C2) |
| TCGA-BH-A0E0-01 | Wound Healing (Immune C1) |
| TCGA-56-7223-01 | Wound Healing (Immune C1) |
| TCGA-EL-A4KG-01 | Lymphocyte Depleted (Immune C4) |
| TCGA-RU-A8FL-01 | Wound Healing (Immune C1) |
| TCGA-HD-A6HZ-01 | Wound Healing (Immune C1) |
| TCGA-AA-A00D-01 | IFN-gamma Dominant (Immune C2) |
| TCGA-C8-A12U-01 | IFN-gamma Dominant (Immune C2) |
| TCGA-BP-4784-01 | Inflammatory (Immune C3) |
| TCGA-F7-8298-01 | Wound Healing (Immune C1) |
| TCGA-XF-A8HD-01 | IFN-gamma Dominant (Immune C2) |
| TCGA-KD-A5QT-01 | Lymphocyte Depleted (Immune C4) |
| TCGA-DD-AACV-01 | Lymphocyte Depleted (Immune C4) |
| TCGA-DJ-A3UK-01 | Inflammatory (Immune C3) |
| TCGA-FY-A4B3-01 | Inflammatory (Immune C3) |
| TCGA-B6-A0WT-01 | Wound Healing (Immune C1) |
| TCGA-24-1548-01 | IFN-gamma Dominant (Immune C2) |
| TCGA-CV-7245-01 | IFN-gamma Dominant (Immune C2) |
| TCGA-D1-A17K-01 | IFN-gamma Dominant (Immune C2) |
| TCGA-A5-A0VP-01 | Wound Healing (Immune C1) |
| TCGA-DU-6403-01 | Lymphocyte Depleted (Immune C4) |
| TCGA-EL-A4K0-01 | Inflammatory (Immune C3) |
| TCGA-J4-A67T-01 | Inflammatory (Immune C3) |
| TCGA-VS-A9V4-01 | Wound Healing (Immune C1) |
| TCGA-DZ-6133-01 | Inflammatory (Immune C3) |
| TCGA-DY-A1H8-01 | Wound Healing (Immune C1) |
| TCGA-ZM-AA05-01 | IFN-gamma Dominant (Immune C2) |
| TCGA-DJ-A2PZ-01 | Inflammatory (Immune C3) |
| TCGA-CN-6016-01 | IFN-gamma Dominant (Immune C2) |
| TCGA-BG-A0M2-01 | Wound Healing (Immune C1) |
| TCGA-AG-3598-01 | Wound Healing (Immune C1) |
| TCGA-BA-5153-01 | Wound Healing (Immune C1) |
| TCGA-BP-4341-01 | Inflammatory (Immune C3) |
| TCGA-2G-AAHP-01 | IFN-gamma Dominant (Immune C2) |
| TCGA-77-6843-01 | Wound Healing (Immune C1) |
| TCGA-BH-A0C1-01 | Wound Healing (Immune C1) |
| TCGA-WB-A81K-01 | Inflammatory (Immune C3) |
| TCGA-R6-A6DQ-01 | IFN-gamma Dominant (Immune C2) |
| TCGA-VQ-A8DV-01 | Wound Healing (Immune C1) |
| TCGA-AR-A1AY-01 | Wound Healing (Immune C1) |
| TCGA-B9-4117-01 | Inflammatory (Immune C3) |
| TCGA-AK-3461-01 | Inflammatory (Immune C3) |
| TCGA-FR-A2OS-01 | Lymphocyte Depleted (Immune C4) |
| TCGA-CH-5737-01 | Inflammatory (Immune C3) |
| TCGA-FJ-A3ZF-01 | Wound Healing (Immune C1) |
| TCGA-UZ-A9Q0-01 | Inflammatory (Immune C3) |
| TCGA-FE-A234-01 | Inflammatory (Immune C3) |
| TCGA-BS-A0TI-01 | IFN-gamma Dominant (Immune C2) |
| TCGA-AA-3966-01 | Wound Healing (Immune C1) |
| TCGA-CA-6719-01 | Wound Healing (Immune C1) |
| TCGA-BG-A0MI-01 | IFN-gamma Dominant (Immune C2) |
| TCGA-AJ-A2QL-01 | Lymphocyte Depleted (Immune C4) |
| TCGA-CD-5813-01 | Inflammatory (Immune C3) |
| TCGA-VQ-AA6I-01 | Wound Healing (Immune C1) |
| TCGA-D8-A27M-01 | IFN-gamma Dominant (Immune C2) |
| TCGA-UY-A9PD-01 | Wound Healing (Immune C1) |
| TCGA-A8-A081-01 | IFN-gamma Dominant (Immune C2) |
| TCGA-V4-A9ED-01 | Inflammatory (Immune C3) |
| TCGA-A5-A0GN-01 | Inflammatory (Immune C3) |
| TCGA-MH-A856-01 | Inflammatory (Immune C3) |
| TCGA-HU-8610-01 | TGF-beta Dominant (Immune C6) |
| TCGA-69-7760-01 | Wound Healing (Immune C1) |
| TCGA-46-3766-01 | TGF-beta Dominant (Immune C6) |
| TCGA-06-5415-01 | Lymphocyte Depleted (Immune C4) |
| TCGA-DD-AAEK-01 | Inflammatory (Immune C3) |
| TCGA-CQ-7069-01 | IFN-gamma Dominant (Immune C2) |
| TCGA-ZM-AA0D-01 | IFN-gamma Dominant (Immune C2) |
| TCGA-33-4533-01 | Wound Healing (Immune C1) |
| TCGA-OR-A5LC-01 | Lymphocyte Depleted (Immune C4) |
| TCGA-VS-A8Q8-01 | IFN-gamma Dominant (Immune C2) |
| TCGA-60-2711-01 | Wound Healing (Immune C1) |
| TCGA-CV-6939-01 | Wound Healing (Immune C1) |
| TCGA-NH-A6GB-01 | Wound Healing (Immune C1) |
| TCGA-DW-7842-01 | Inflammatory (Immune C3) |
| TCGA-13-1497-01 | IFN-gamma Dominant (Immune C2) |
| TCGA-EO-A3AY-01 | IFN-gamma Dominant (Immune C2) |
| TCGA-CZ-5470-01 | Inflammatory (Immune C3) |
| TCGA-AX-A3G1-01 | IFN-gamma Dominant (Immune C2) |
| TCGA-AA-3712-01 | Wound Healing (Immune C1) |
| TCGA-AG-3594-01 | IFN-gamma Dominant (Immune C2) |
| TCGA-AZ-4614-01 | Wound Healing (Immune C1) |
| TCGA-B0-4824-01 | Inflammatory (Immune C3) |
| TCGA-QR-A70T-01 | Inflammatory (Immune C3) |
| TCGA-AA-A00O-01 | Wound Healing (Immune C1) |
| TCGA-BP-4345-01 | Inflammatory (Immune C3) |
| TCGA-B0-5115-01 | Inflammatory (Immune C3) |
| TCGA-DK-AA6U-01 | IFN-gamma Dominant (Immune C2) |
| TCGA-DD-AACB-01 | IFN-gamma Dominant (Immune C2) |
| TCGA-DS-A0VN-01 | IFN-gamma Dominant (Immune C2) |
| TCGA-DB-A64R-01 | Immunologically Quiet (Immune C5) |
| TCGA-ZF-AA5H-01 | IFN-gamma Dominant (Immune C2) |
| TCGA-ET-A25M-01 | Inflammatory (Immune C3) |
| TCGA-B5-A11N-01 | IFN-gamma Dominant (Immune C2) |
| TCGA-A6-3809-01 | IFN-gamma Dominant (Immune C2) |
| TCGA-VP-A87H-01 | Inflammatory (Immune C3) |
| TCGA-G9-7521-01 | IFN-gamma Dominant (Immune C2) |
| TCGA-YL-A8HO-01 | Inflammatory (Immune C3) |
| TCGA-46-3769-01 | IFN-gamma Dominant (Immune C2) |
| TCGA-CM-5864-01 | Wound Healing (Immune C1) |
| TCGA-DX-A1KY-01 | Wound Healing (Immune C1) |
| TCGA-HC-7079-01 | Inflammatory (Immune C3) |
| TCGA-UF-A71B-01 | IFN-gamma Dominant (Immune C2) |
| TCGA-LL-A5YP-01 | Wound Healing (Immune C1) |
| TCGA-E2-A15H-01 | Wound Healing (Immune C1) |
| TCGA-J4-AATZ-01 | Inflammatory (Immune C3) |
| TCGA-E1-A7YQ-01 | Lymphocyte Depleted (Immune C4) |
| TCGA-CV-6935-01 | Wound Healing (Immune C1) |
| TCGA-D7-A4YX-01 | IFN-gamma Dominant (Immune C2) |
| TCGA-FV-A4ZQ-01 | IFN-gamma Dominant (Immune C2) |
| TCGA-BH-A0B0-01 | IFN-gamma Dominant (Immune C2) |
| TCGA-A8-A093-01 | Wound Healing (Immune C1) |
| TCGA-KN-8434-01 | Inflammatory (Immune C3) |
| TCGA-D9-A4Z5-01 | Lymphocyte Depleted (Immune C4) |
| TCGA-BP-4758-01 | Inflammatory (Immune C3) |
| TCGA-DI-A2QU-01 | Wound Healing (Immune C1) |
| TCGA-MA-AA3Y-01 | IFN-gamma Dominant (Immune C2) |
| TCGA-BP-5200-01 | Inflammatory (Immune C3) |
| TCGA-EB-A5UM-01 | Inflammatory (Immune C3) |
| TCGA-AG-3600-01 | Wound Healing (Immune C1) |
| TCGA-CI-6624-01 | Wound Healing (Immune C1) |
| TCGA-69-7764-01 | Inflammatory (Immune C3) |
| TCGA-FY-A3YR-01 | Inflammatory (Immune C3) |
| TCGA-BH-A18S-01 | Inflammatory (Immune C3) |
| TCGA-UF-A7JH-01 | IFN-gamma Dominant (Immune C2) |
| TCGA-44-2665-01 | TGF-beta Dominant (Immune C6) |
| TCGA-V4-A9F4-01 | Lymphocyte Depleted (Immune C4) |
| TCGA-HU-A4G2-01 | IFN-gamma Dominant (Immune C2) |
| TCGA-AR-A1AO-01 | IFN-gamma Dominant (Immune C2) |
| TCGA-WB-A81D-01 | Inflammatory (Immune C3) |
| TCGA-TN-A7HI-01 | IFN-gamma Dominant (Immune C2) |
| TCGA-ZF-AA54-01 | Lymphocyte Depleted (Immune C4) |
| TCGA-NC-A5HJ-01 | IFN-gamma Dominant (Immune C2) |
| TCGA-55-8505-01 | Wound Healing (Immune C1) |
| TCGA-FI-A2CX-01 | IFN-gamma Dominant (Immune C2) |
| TCGA-67-6217-01 | Inflammatory (Immune C3) |
| TCGA-MU-A8JM-01 | Wound Healing (Immune C1) |
| TCGA-BJ-A4O9-01 | Inflammatory (Immune C3) |
| TCGA-MX-A5UG-01 | Inflammatory (Immune C3) |
| TCGA-CR-6477-01 | IFN-gamma Dominant (Immune C2) |
| TCGA-B6-A0IB-01 | Wound Healing (Immune C1) |
| TCGA-AA-3851-01 | Wound Healing (Immune C1) |
| TCGA-PZ-A5RE-01 | Wound Healing (Immune C1) |
| TCGA-BP-4335-01 | Inflammatory (Immune C3) |
| TCGA-C5-A2LY-01 | IFN-gamma Dominant (Immune C2) |
| TCGA-XF-A9ST-01 | Wound Healing (Immune C1) |
| TCGA-B0-5698-01 | Inflammatory (Immune C3) |
| TCGA-BP-4173-01 | Inflammatory (Immune C3) |
| TCGA-AN-A0FV-01 | Lymphocyte Depleted (Immune C4) |
| TCGA-Q9-A6FU-01 | Wound Healing (Immune C1) |
| TCGA-FX-A2QS-01 | TGF-beta Dominant (Immune C6) |
| TCGA-G3-A6UC-01 | Lymphocyte Depleted (Immune C4) |
| TCGA-DX-A3U7-01 | IFN-gamma Dominant (Immune C2) |
| TCGA-FU-A23K-01 | IFN-gamma Dominant (Immune C2) |
| TCGA-HZ-7925-01 | Wound Healing (Immune C1) |
| TCGA-LN-A49M-01 | IFN-gamma Dominant (Immune C2) |
| TCGA-L9-A7SV-01 | Wound Healing (Immune C1) |
| TCGA-KQ-A41R-01 | Lymphocyte Depleted (Immune C4) |
| TCGA-HV-A5A6-01 | IFN-gamma Dominant (Immune C2) |
| TCGA-EA-A6QX-01 | IFN-gamma Dominant (Immune C2) |
| TCGA-55-8089-01 | IFN-gamma Dominant (Immune C2) |
| TCGA-VQ-A94T-01 | Wound Healing (Immune C1) |
| TCGA-DX-A3UC-01 | Lymphocyte Depleted (Immune C4) |
| TCGA-IQ-7630-01 | IFN-gamma Dominant (Immune C2) |
| TCGA-XF-AAN3-01 | Wound Healing (Immune C1) |
| TCGA-EJ-7328-01 | Inflammatory (Immune C3) |
| TCGA-BP-4787-01 | Inflammatory (Immune C3) |
| TCGA-YZ-A982-01 | Immunologically Quiet (Immune C5) |
| TCGA-BG-A0M6-01 | Wound Healing (Immune C1) |
| TCGA-EM-A3FL-01 | Inflammatory (Immune C3) |
| TCGA-W5-AA2O-01 | Lymphocyte Depleted (Immune C4) |
| TCGA-B2-5636-01 | Inflammatory (Immune C3) |
| TCGA-BF-A1PV-01 | Wound Healing (Immune C1) |
| TCGA-DD-AACU-01 | Lymphocyte Depleted (Immune C4) |
| TCGA-B0-5108-01 | Inflammatory (Immune C3) |
| TCGA-DD-A4NJ-01 | Inflammatory (Immune C3) |
| TCGA-D1-A17L-01 | Inflammatory (Immune C3) |
| TCGA-EO-A3B1-01 | Wound Healing (Immune C1) |
| TCGA-AG-A02G-01 | Inflammatory (Immune C3) |
| TCGA-EJ-7317-01 | Inflammatory (Immune C3) |
| TCGA-BP-5168-01 | Inflammatory (Immune C3) |
| TCGA-AP-A05A-01 | Wound Healing (Immune C1) |
| TCGA-KQ-A41Q-01 | Wound Healing (Immune C1) |
| TCGA-AR-A24Z-01 | IFN-gamma Dominant (Immune C2) |
| TCGA-44-8117-01 | Wound Healing (Immune C1) |
| TCGA-CV-7101-01 | Wound Healing (Immune C1) |
| TCGA-B0-4699-01 | IFN-gamma Dominant (Immune C2) |
| TCGA-GI-A2C9-01 | IFN-gamma Dominant (Immune C2) |
| TCGA-S9-A7J1-01 | Immunologically Quiet (Immune C5) |
| TCGA-CK-5914-01 | Wound Healing (Immune C1) |
| TCGA-D8-A145-01 | Inflammatory (Immune C3) |
| TCGA-CR-6473-01 | IFN-gamma Dominant (Immune C2) |
| TCGA-AA-3844-01 | Wound Healing (Immune C1) |
| TCGA-AA-A01K-01 | Wound Healing (Immune C1) |
| TCGA-D8-A1XB-01 | Wound Healing (Immune C1) |
| TCGA-LC-A66R-01 | IFN-gamma Dominant (Immune C2) |
| TCGA-LK-A4O2-01 | Lymphocyte Depleted (Immune C4) |
| TCGA-18-4086-01 | Wound Healing (Immune C1) |
| TCGA-ET-A3DU-01 | Inflammatory (Immune C3) |
| TCGA-A2-A0YL-01 | Wound Healing (Immune C1) |
| TCGA-A7-A5ZX-01 | Inflammatory (Immune C3) |
| TCGA-B2-5635-01 | Inflammatory (Immune C3) |
| TCGA-AP-A0LF-01 | IFN-gamma Dominant (Immune C2) |
| TCGA-BP-4162-01 | Inflammatory (Immune C3) |
| TCGA-EW-A1P7-01 | Inflammatory (Immune C3) |
| TCGA-AX-A2H4-01 | IFN-gamma Dominant (Immune C2) |
| TCGA-AR-A5QQ-01 | IFN-gamma Dominant (Immune C2) |
| TCGA-SQ-A6I4-01 | Inflammatory (Immune C3) |
| TCGA-C5-A7CH-01 | IFN-gamma Dominant (Immune C2) |
| TCGA-E1-A7YJ-01 | Lymphocyte Depleted (Immune C4) |
| TCGA-W5-AA31-01 | Wound Healing (Immune C1) |
| TCGA-GJ-A9DB-01 | Inflammatory (Immune C3) |
| TCGA-S3-AA15-01 | Wound Healing (Immune C1) |
| TCGA-HZ-8519-01 | Inflammatory (Immune C3) |
| TCGA-HI-7168-01 | Wound Healing (Immune C1) |
| TCGA-BR-8081-01 | IFN-gamma Dominant (Immune C2) |
| TCGA-B4-5838-01 | Inflammatory (Immune C3) |
| TCGA-06-5411-01 | Lymphocyte Depleted (Immune C4) |
| TCGA-CV-A45Z-01 | IFN-gamma Dominant (Immune C2) |
| TCGA-5P-A9KH-01 | Lymphocyte Depleted (Immune C4) |
| TCGA-KC-A4BL-01 | Inflammatory (Immune C3) |
| TCGA-BJ-A45C-01 | Lymphocyte Depleted (Immune C4) |
| TCGA-66-2783-01 | Wound Healing (Immune C1) |
| TCGA-DK-A3WW-01 | IFN-gamma Dominant (Immune C2) |
| TCGA-YA-A8S7-01 | Lymphocyte Depleted (Immune C4) |
| TCGA-62-A46V-01 | Lymphocyte Depleted (Immune C4) |
| TCGA-DD-A1EC-01 | Inflammatory (Immune C3) |
| TCGA-AR-A24M-01 | TGF-beta Dominant (Immune C6) |
| TCGA-SY-A9G5-01 | IFN-gamma Dominant (Immune C2) |
| TCGA-AX-A0IU-01 | Lymphocyte Depleted (Immune C4) |
| TCGA-KN-8422-01 | Immunologically Quiet (Immune C5) |
| TCGA-ZN-A9VV-01 | Lymphocyte Depleted (Immune C4) |
| TCGA-4Z-AA89-01 | Lymphocyte Depleted (Immune C4) |
| TCGA-AA-A00L-01 | Wound Healing (Immune C1) |
| TCGA-26-5139-01 | Lymphocyte Depleted (Immune C4) |
| TCGA-3B-A9HV-01 | Wound Healing (Immune C1) |
| TCGA-EK-A2RN-01 | Lymphocyte Depleted (Immune C4) |
| TCGA-P3-A6T5-01 | IFN-gamma Dominant (Immune C2) |
| TCGA-P6-A5OF-01 | Lymphocyte Depleted (Immune C4) |
| TCGA-EY-A548-01 | IFN-gamma Dominant (Immune C2) |
| TCGA-B0-4828-01 | Inflammatory (Immune C3) |
| TCGA-BG-A222-01 | IFN-gamma Dominant (Immune C2) |
| TCGA-FD-A62S-01 | IFN-gamma Dominant (Immune C2) |
| TCGA-DJ-A3UW-01 | Inflammatory (Immune C3) |
| TCGA-86-8673-01 | Wound Healing (Immune C1) |
| TCGA-DU-7018-01 | Immunologically Quiet (Immune C5) |
| TCGA-BC-A10U-01 | Inflammatory (Immune C3) |
| TCGA-CR-6478-01 | IFN-gamma Dominant (Immune C2) |
| TCGA-66-2795-01 | Wound Healing (Immune C1) |
| TCGA-EJ-A65G-01 | Inflammatory (Immune C3) |
| TCGA-A2-A0EW-01 | Inflammatory (Immune C3) |
| TCGA-BR-8371-01 | Inflammatory (Immune C3) |
| TCGA-A3-3380-01 | Inflammatory (Immune C3) |
| TCGA-HT-7857-01 | Lymphocyte Depleted (Immune C4) |
| TCGA-HT-7690-01 | Lymphocyte Depleted (Immune C4) |
| TCGA-CM-5341-01 | IFN-gamma Dominant (Immune C2) |
| TCGA-97-8171-01 | Lymphocyte Depleted (Immune C4) |
| TCGA-BP-4803-01 | Inflammatory (Immune C3) |
| TCGA-QG-A5YW-01 | Wound Healing (Immune C1) |
| TCGA-TM-A84M-01 | Immunologically Quiet (Immune C5) |
| TCGA-BP-5191-01 | Inflammatory (Immune C3) |
| TCGA-LN-A4MR-01 | IFN-gamma Dominant (Immune C2) |
| TCGA-CN-A499-01 | IFN-gamma Dominant (Immune C2) |
| TCGA-J8-A3YH-01 | Inflammatory (Immune C3) |
| TCGA-ET-A3DQ-01 | Inflammatory (Immune C3) |
| TCGA-CR-6482-01 | IFN-gamma Dominant (Immune C2) |
| TCGA-50-7109-01 | IFN-gamma Dominant (Immune C2) |
| TCGA-DU-A7TJ-01 | Lymphocyte Depleted (Immune C4) |
| TCGA-AA-A01X-01 | Wound Healing (Immune C1) |
| TCGA-FY-A40N-01 | Inflammatory (Immune C3) |
| TCGA-BH-A1FD-01 | Lymphocyte Depleted (Immune C4) |
| TCGA-DX-A8BN-01 | Lymphocyte Depleted (Immune C4) |
| TCGA-EW-A424-01 | Wound Healing (Immune C1) |
| TCGA-E2-A2P5-01 | Wound Healing (Immune C1) |
| TCGA-DD-AACZ-01 | IFN-gamma Dominant (Immune C2) |
| TCGA-44-6145-01 | IFN-gamma Dominant (Immune C2) |
| TCGA-PT-A8TR-01 | Lymphocyte Depleted (Immune C4) |
| TCGA-HT-8105-01 | Immunologically Quiet (Immune C5) |
| TCGA-BR-4256-01 | IFN-gamma Dominant (Immune C2) |
| TCGA-L5-A4OP-01 | Wound Healing (Immune C1) |
| TCGA-CC-A7II-01 | Wound Healing (Immune C1) |
| TCGA-A2-A259-01 | Wound Healing (Immune C1) |
| TCGA-E2-A1B1-01 | IFN-gamma Dominant (Immune C2) |
| TCGA-A7-A3J0-01 | Inflammatory (Immune C3) |
| TCGA-B5-A3S1-01 | IFN-gamma Dominant (Immune C2) |
| TCGA-N5-A4RD-01 | Wound Healing (Immune C1) |
| TCGA-76-4928-01 | Lymphocyte Depleted (Immune C4) |
| TCGA-DX-A7ER-01 | Wound Healing (Immune C1) |
| TCGA-55-8085-01 | IFN-gamma Dominant (Immune C2) |
| TCGA-D5-6538-01 | Wound Healing (Immune C1) |
| TCGA-DD-AADC-01 | Lymphocyte Depleted (Immune C4) |
| TCGA-06-0644-01 | Lymphocyte Depleted (Immune C4) |
| TCGA-BR-8078-01 | IFN-gamma Dominant (Immune C2) |
| TCGA-AA-3560-01 | Wound Healing (Immune C1) |
| TCGA-EK-A2RJ-01 | IFN-gamma Dominant (Immune C2) |
| TCGA-DD-AACW-01 | Lymphocyte Depleted (Immune C4) |
| TCGA-VS-A9V0-01 | Wound Healing (Immune C1) |
| TCGA-EJ-7318-01 | Lymphocyte Depleted (Immune C4) |
| TCGA-A6-6141-01 | Wound Healing (Immune C1) |
| TCGA-C4-A0F0-01 | IFN-gamma Dominant (Immune C2) |
| TCGA-ET-A3BP-01 | Inflammatory (Immune C3) |
| TCGA-E1-A7YY-01 | Immunologically Quiet (Immune C5) |
| TCGA-ZM-AA0H-01 | IFN-gamma Dominant (Immune C2) |
| TCGA-OR-A5KX-01 | Lymphocyte Depleted (Immune C4) |
| TCGA-24-1846-01 | Lymphocyte Depleted (Immune C4) |
| TCGA-SN-A84Y-01 | IFN-gamma Dominant (Immune C2) |
| TCGA-K4-AAQO-01 | Wound Healing (Immune C1) |
| TCGA-BR-6565-01 | IFN-gamma Dominant (Immune C2) |
| TCGA-CR-7385-01 | IFN-gamma Dominant (Immune C2) |
| TCGA-EO-A22U-01 | Wound Healing (Immune C1) |
| TCGA-A2-A0ES-01 | Inflammatory (Immune C3) |
| TCGA-WK-A8XQ-01 | Lymphocyte Depleted (Immune C4) |
| TCGA-NJ-A4YF-01 | Wound Healing (Immune C1) |
| TCGA-CV-5439-01 | IFN-gamma Dominant (Immune C2) |
| TCGA-33-4547-01 | Wound Healing (Immune C1) |
| TCGA-2G-AAHA-01 | IFN-gamma Dominant (Immune C2) |
| TCGA-R8-A6MK-01 | Immunologically Quiet (Immune C5) |
| TCGA-24-2298-01 | IFN-gamma Dominant (Immune C2) |
| TCGA-25-1631-01 | Lymphocyte Depleted (Immune C4) |
| TCGA-QR-A70X-01 | Inflammatory (Immune C3) |
| TCGA-DU-A7TA-01 | Immunologically Quiet (Immune C5) |
| TCGA-C5-A1BM-01 | IFN-gamma Dominant (Immune C2) |
| TCGA-DU-6400-01 | Immunologically Quiet (Immune C5) |
| TCGA-QK-A8Z9-01 | IFN-gamma Dominant (Immune C2) |
| TCGA-AJ-A8CT-01 | Inflammatory (Immune C3) |
| TCGA-NK-A5CT-01 | Wound Healing (Immune C1) |
| TCGA-AR-A0U4-01 | Lymphocyte Depleted (Immune C4) |
| TCGA-CV-7427-01 | IFN-gamma Dominant (Immune C2) |
| TCGA-EL-A4JV-01 | Inflammatory (Immune C3) |
| TCGA-24-1416-01 | Lymphocyte Depleted (Immune C4) |
| TCGA-WB-A80P-01 | Inflammatory (Immune C3) |
| TCGA-P5-A737-01 | Immunologically Quiet (Immune C5) |
| TCGA-B8-4620-01 | Lymphocyte Depleted (Immune C4) |
| TCGA-VD-A8K9-01 | Lymphocyte Depleted (Immune C4) |
| TCGA-AA-3681-01 | Wound Healing (Immune C1) |
| TCGA-CM-6172-01 | Wound Healing (Immune C1) |
| TCGA-3U-A98E-01 | IFN-gamma Dominant (Immune C2) |
| TCGA-B5-A11V-01 | Wound Healing (Immune C1) |
| TCGA-E7-A7DU-01 | TGF-beta Dominant (Immune C6) |
| TCGA-CJ-4904-01 | Inflammatory (Immune C3) |
| TCGA-14-0871-01 | Wound Healing (Immune C1) |
| TCGA-A4-7583-01 | Inflammatory (Immune C3) |
| TCGA-NJ-A7XG-01 | Inflammatory (Immune C3) |
| TCGA-JY-A93C-01 | IFN-gamma Dominant (Immune C2) |
| TCGA-D5-6533-01 | Wound Healing (Immune C1) |
| TCGA-VN-A88I-01 | Inflammatory (Immune C3) |
| TCGA-EP-A3RK-01 | IFN-gamma Dominant (Immune C2) |
| TCGA-RY-A83Y-01 | Immunologically Quiet (Immune C5) |
| TCGA-SJ-A6ZJ-01 | Wound Healing (Immune C1) |
| TCGA-TT-A6YO-01 | Lymphocyte Depleted (Immune C4) |
| TCGA-HC-A9TE-01 | Inflammatory (Immune C3) |
| TCGA-BJ-A2N8-01 | Inflammatory (Immune C3) |
| TCGA-YZ-A985-01 | Lymphocyte Depleted (Immune C4) |
| TCGA-A8-A08C-01 | Inflammatory (Immune C3) |
| TCGA-BP-5175-01 | TGF-beta Dominant (Immune C6) |
| TCGA-HT-8564-01 | Lymphocyte Depleted (Immune C4) |
| TCGA-05-4403-01 | TGF-beta Dominant (Immune C6) |
| TCGA-55-8097-01 | Inflammatory (Immune C3) |
| TCGA-LL-A7T0-01 | IFN-gamma Dominant (Immune C2) |
| TCGA-NI-A4U2-01 | Lymphocyte Depleted (Immune C4) |
| TCGA-DX-AB2Z-01 | TGF-beta Dominant (Immune C6) |
| TCGA-KK-A6E2-01 | Inflammatory (Immune C3) |
| TCGA-BQ-5879-01 | Inflammatory (Immune C3) |
| TCGA-AA-3861-01 | IFN-gamma Dominant (Immune C2) |
| TCGA-VP-A872-01 | Inflammatory (Immune C3) |
| TCGA-DD-AAVQ-01 | IFN-gamma Dominant (Immune C2) |
| TCGA-HT-7686-01 | Immunologically Quiet (Immune C5) |
| TCGA-LN-A7HW-01 | Wound Healing (Immune C1) |
| TCGA-2G-AAGZ-01 | IFN-gamma Dominant (Immune C2) |
| TCGA-B4-5834-01 | Inflammatory (Immune C3) |
| TCGA-BA-6873-01 | IFN-gamma Dominant (Immune C2) |
| TCGA-85-8582-01 | IFN-gamma Dominant (Immune C2) |
| TCGA-AP-A1E4-01 | Wound Healing (Immune C1) |
| TCGA-F7-7848-01 | Wound Healing (Immune C1) |
| TCGA-CN-4725-01 | Wound Healing (Immune C1) |
| TCGA-CD-8531-01 | IFN-gamma Dominant (Immune C2) |
| TCGA-NJ-A4YQ-01 | IFN-gamma Dominant (Immune C2) |
| TCGA-B5-A121-01 | Wound Healing (Immune C1) |
| TCGA-DK-A1A3-01 | Wound Healing (Immune C1) |
| TCGA-DS-A7WH-01 | Wound Healing (Immune C1) |
| TCGA-ZJ-AAX4-01 | IFN-gamma Dominant (Immune C2) |
| TCGA-QK-AA3J-01 | Wound Healing (Immune C1) |
| TCGA-97-7547-01 | Inflammatory (Immune C3) |
| TCGA-C5-A7CK-01 | IFN-gamma Dominant (Immune C2) |
| TCGA-KK-A7AP-01 | Inflammatory (Immune C3) |
| TCGA-E1-A7YM-01 | Immunologically Quiet (Immune C5) |
| TCGA-V1-A9OH-01 | Inflammatory (Immune C3) |
| TCGA-DM-A28H-01 | Wound Healing (Immune C1) |
| TCGA-DV-5567-01 | Inflammatory (Immune C3) |
| TCGA-AG-3883-01 | Wound Healing (Immune C1) |
| TCGA-J8-A42S-01 | Inflammatory (Immune C3) |
| TCGA-UD-AABY-01 | TGF-beta Dominant (Immune C6) |
| TCGA-VS-A8EK-01 | IFN-gamma Dominant (Immune C2) |
| TCGA-KQ-A41N-01 | Wound Healing (Immune C1) |
| TCGA-A7-A13D-01 | IFN-gamma Dominant (Immune C2) |
| TCGA-D6-6824-01 | IFN-gamma Dominant (Immune C2) |
| TCGA-MQ-A6BR-01 | Wound Healing (Immune C1) |
| TCGA-DH-A66F-01 | Immunologically Quiet (Immune C5) |
| TCGA-EW-A1OZ-01 | Wound Healing (Immune C1) |
| TCGA-B9-4617-01 | Inflammatory (Immune C3) |
| TCGA-F4-6460-01 | Wound Healing (Immune C1) |
| TCGA-B0-5116-01 | Inflammatory (Immune C3) |
| TCGA-A2-A1FX-01 | Lymphocyte Depleted (Immune C4) |
| TCGA-60-2716-01 | Wound Healing (Immune C1) |
| TCGA-KN-8421-01 | Lymphocyte Depleted (Immune C4) |
| TCGA-A4-A57E-01 | Lymphocyte Depleted (Immune C4) |
| TCGA-RW-A688-01 | Immunologically Quiet (Immune C5) |
| TCGA-AH-6897-01 | Wound Healing (Immune C1) |
| TCGA-A7-A4SD-01 | IFN-gamma Dominant (Immune C2) |
| TCGA-XF-A9T6-01 | IFN-gamma Dominant (Immune C2) |
| TCGA-2G-AAG6-01 | Inflammatory (Immune C3) |
| TCGA-EM-A2OY-01 | Inflammatory (Immune C3) |
| TCGA-HZ-7926-01 | Inflammatory (Immune C3) |
| TCGA-D5-5540-01 | Wound Healing (Immune C1) |
| TCGA-EA-A1QS-01 | Wound Healing (Immune C1) |
| TCGA-ET-A25N-01 | Inflammatory (Immune C3) |
| TCGA-43-8118-01 | Lymphocyte Depleted (Immune C4) |
| TCGA-DK-A2I4-01 | IFN-gamma Dominant (Immune C2) |
| TCGA-VD-A8KB-01 | Lymphocyte Depleted (Immune C4) |
| TCGA-HU-A4G9-01 | Wound Healing (Immune C1) |
| TCGA-NK-A5CR-01 | Wound Healing (Immune C1) |
| TCGA-EB-A553-01 | Wound Healing (Immune C1) |
| TCGA-A6-2672-01 | IFN-gamma Dominant (Immune C2) |
| TCGA-EM-A4FU-01 | Inflammatory (Immune C3) |
| TCGA-2Z-A9JD-01 | Inflammatory (Immune C3) |
| TCGA-VP-A876-01 | Inflammatory (Immune C3) |
| TCGA-HU-A4GN-01 | IFN-gamma Dominant (Immune C2) |
| TCGA-DD-AAVU-01 | Lymphocyte Depleted (Immune C4) |
| TCGA-SI-A71O-01 | Wound Healing (Immune C1) |
| TCGA-P4-A5EA-01 | IFN-gamma Dominant (Immune C2) |
| TCGA-25-2393-01 | Wound Healing (Immune C1) |
| TCGA-XD-AAUI-01 | Inflammatory (Immune C3) |
| TCGA-D5-5537-01 | Wound Healing (Immune C1) |
| TCGA-F4-6463-01 | Wound Healing (Immune C1) |
| TCGA-FV-A23B-01 | Inflammatory (Immune C3) |
| TCGA-CS-5390-01 | Immunologically Quiet (Immune C5) |
| TCGA-MI-A75H-01 | Lymphocyte Depleted (Immune C4) |
| TCGA-B5-A11E-01 | Wound Healing (Immune C1) |
| TCGA-2H-A9GH-01 | IFN-gamma Dominant (Immune C2) |
| TCGA-GM-A5PX-01 | Inflammatory (Immune C3) |
| TCGA-AY-4071-01 | Wound Healing (Immune C1) |
| TCGA-49-AARN-01 | IFN-gamma Dominant (Immune C2) |
| TCGA-FU-A3WB-01 | IFN-gamma Dominant (Immune C2) |
| TCGA-2G-AAGY-01 | Wound Healing (Immune C1) |
| TCGA-S5-A6DX-01 | IFN-gamma Dominant (Immune C2) |
| TCGA-OY-A56P-01 | IFN-gamma Dominant (Immune C2) |
| TCGA-BH-A0DE-01 | TGF-beta Dominant (Immune C6) |
| TCGA-32-2632-01 | Lymphocyte Depleted (Immune C4) |
| TCGA-AO-A0JM-01 | IFN-gamma Dominant (Immune C2) |
| TCGA-IM-A3U3-01 | Inflammatory (Immune C3) |
| TCGA-E2-A14P-01 | Wound Healing (Immune C1) |
| TCGA-ZG-A9L6-01 | Inflammatory (Immune C3) |
| TCGA-E9-A5UP-01 | Lymphocyte Depleted (Immune C4) |
| TCGA-FG-A87N-01 | Lymphocyte Depleted (Immune C4) |
| TCGA-E9-A227-01 | Wound Healing (Immune C1) |
| TCGA-AP-A059-01 | IFN-gamma Dominant (Immune C2) |
| TCGA-L5-A4OS-01 | Wound Healing (Immune C1) |
| TCGA-OL-A5D7-01 | IFN-gamma Dominant (Immune C2) |
| TCGA-BF-AAP7-01 | IFN-gamma Dominant (Immune C2) |
| TCGA-VV-A829-01 | Immunologically Quiet (Immune C5) |
| TCGA-RZ-AB0B-01 | Inflammatory (Immune C3) |
| TCGA-44-A4SS-01 | Inflammatory (Immune C3) |
| TCGA-DH-A66B-01 | Immunologically Quiet (Immune C5) |
| TCGA-FU-A3TQ-01 | IFN-gamma Dominant (Immune C2) |
| TCGA-2J-AABR-01 | Inflammatory (Immune C3) |
| TCGA-A8-A07O-01 | Wound Healing (Immune C1) |
| TCGA-EJ-7792-01 | Inflammatory (Immune C3) |
| TCGA-B0-5693-01 | Inflammatory (Immune C3) |
| TCGA-XE-AAO6-01 | IFN-gamma Dominant (Immune C2) |
| TCGA-GN-A8LN-01 | Wound Healing (Immune C1) |
| TCGA-CD-A4MI-01 | IFN-gamma Dominant (Immune C2) |
| TCGA-AJ-A3EK-01 | Wound Healing (Immune C1) |
| TCGA-CV-5431-01 | Wound Healing (Immune C1) |
| TCGA-BR-A4J2-01 | Inflammatory (Immune C3) |
| TCGA-85-8350-01 | Wound Healing (Immune C1) |
| TCGA-2G-AAG9-01 | IFN-gamma Dominant (Immune C2) |
| TCGA-DJ-A13U-01 | Inflammatory (Immune C3) |
| TCGA-DD-A4NA-01 | Inflammatory (Immune C3) |
| TCGA-IS-A3K6-01 | IFN-gamma Dominant (Immune C2) |
| TCGA-DX-A7EN-01 | Lymphocyte Depleted (Immune C4) |
| TCGA-BT-A42E-01 | IFN-gamma Dominant (Immune C2) |
| TCGA-CR-5249-01 | TGF-beta Dominant (Immune C6) |
| TCGA-HT-A5R7-01 | Lymphocyte Depleted (Immune C4) |
| TCGA-AA-3815-01 | IFN-gamma Dominant (Immune C2) |
| TCGA-CJ-6027-01 | IFN-gamma Dominant (Immune C2) |
| TCGA-C8-A1HG-01 | IFN-gamma Dominant (Immune C2) |
| TCGA-AO-A0JA-01 | Wound Healing (Immune C1) |
| TCGA-2J-AABV-01 | Wound Healing (Immune C1) |
| TCGA-A2-A4S0-01 | Inflammatory (Immune C3) |
| TCGA-53-7813-01 | IFN-gamma Dominant (Immune C2) |
| TCGA-BJ-A0ZA-01 | Inflammatory (Immune C3) |
| TCGA-G3-A7M8-01 | Lymphocyte Depleted (Immune C4) |
| TCGA-AA-3977-01 | Wound Healing (Immune C1) |
| TCGA-98-A539-01 | Wound Healing (Immune C1) |
| TCGA-O2-A52S-01 | Wound Healing (Immune C1) |
| TCGA-EB-A57M-01 | Inflammatory (Immune C3) |
| TCGA-G9-6329-01 | Inflammatory (Immune C3) |
| TCGA-25-2397-01 | Lymphocyte Depleted (Immune C4) |
| TCGA-FR-A726-01 | Lymphocyte Depleted (Immune C4) |
| TCGA-C5-A8XI-01 | IFN-gamma Dominant (Immune C2) |
| TCGA-05-5715-01 | TGF-beta Dominant (Immune C6) |
| TCGA-86-7713-01 | IFN-gamma Dominant (Immune C2) |
| TCGA-C5-A1BN-01 | IFN-gamma Dominant (Immune C2) |
| TCGA-CS-5394-01 | Immunologically Quiet (Immune C5) |
| TCGA-RT-A6Y9-01 | Lymphocyte Depleted (Immune C4) |
| TCGA-US-A774-01 | TGF-beta Dominant (Immune C6) |
| TCGA-QT-A69Q-01 | Inflammatory (Immune C3) |
| TCGA-D7-A4YV-01 | IFN-gamma Dominant (Immune C2) |
| TCGA-KR-A7K7-01 | IFN-gamma Dominant (Immune C2) |
| TCGA-CV-7247-01 | Wound Healing (Immune C1) |
| TCGA-CD-A48A-01 | IFN-gamma Dominant (Immune C2) |
| TCGA-DD-A4NV-01 | Inflammatory (Immune C3) |
| TCGA-TS-A7P7-01 | IFN-gamma Dominant (Immune C2) |
| TCGA-BQ-5875-01 | Lymphocyte Depleted (Immune C4) |
| TCGA-B6-A0IE-01 | Wound Healing (Immune C1) |
| TCGA-05-4382-01 | TGF-beta Dominant (Immune C6) |
| TCGA-43-A475-01 | IFN-gamma Dominant (Immune C2) |
| TCGA-ZP-A9CV-01 | Lymphocyte Depleted (Immune C4) |
| TCGA-BC-A10Y-01 | Lymphocyte Depleted (Immune C4) |
| TCGA-24-2281-01 | IFN-gamma Dominant (Immune C2) |
| TCGA-VQ-A91S-01 | IFN-gamma Dominant (Immune C2) |
| TCGA-L5-A8NL-01 | Wound Healing (Immune C1) |
| TCGA-N6-A4VG-01 | Wound Healing (Immune C1) |
| TCGA-E9-A1QZ-01 | IFN-gamma Dominant (Immune C2) |
| TCGA-AK-3453-01 | Lymphocyte Depleted (Immune C4) |
| TCGA-KM-8440-01 | Immunologically Quiet (Immune C5) |
| TCGA-FD-A43P-01 | IFN-gamma Dominant (Immune C2) |
| TCGA-56-7823-01 | Wound Healing (Immune C1) |
| TCGA-ZJ-A8QO-01 | IFN-gamma Dominant (Immune C2) |
| TCGA-93-A4JQ-01 | IFN-gamma Dominant (Immune C2) |
| TCGA-BR-8364-01 | Inflammatory (Immune C3) |
| TCGA-AA-3811-01 | Wound Healing (Immune C1) |
| TCGA-HT-7877-01 | Immunologically Quiet (Immune C5) |
| TCGA-F5-6812-01 | Wound Healing (Immune C1) |
| TCGA-CR-7401-01 | IFN-gamma Dominant (Immune C2) |
| TCGA-CV-6940-01 | Wound Healing (Immune C1) |
| TCGA-DG-A2KM-01 | IFN-gamma Dominant (Immune C2) |
| TCGA-BQ-5876-01 | Lymphocyte Depleted (Immune C4) |
| TCGA-IR-A3LA-01 | Wound Healing (Immune C1) |
| TCGA-OR-A5J1-01 | Lymphocyte Depleted (Immune C4) |
| TCGA-OL-A66J-01 | Wound Healing (Immune C1) |
| TCGA-CN-5359-01 | IFN-gamma Dominant (Immune C2) |
| TCGA-QK-A652-01 | IFN-gamma Dominant (Immune C2) |
| TCGA-HT-A618-01 | Immunologically Quiet (Immune C5) |
| TCGA-06-0882-01 | Lymphocyte Depleted (Immune C4) |
| TCGA-DE-A4M9-01 | Inflammatory (Immune C3) |
| TCGA-DU-6542-01 | Immunologically Quiet (Immune C5) |
| TCGA-VQ-A922-01 | Wound Healing (Immune C1) |
| TCGA-05-5428-01 | IFN-gamma Dominant (Immune C2) |
| TCGA-EL-A3ZT-01 | Inflammatory (Immune C3) |
| TCGA-AL-7173-01 | Inflammatory (Immune C3) |
| TCGA-BR-4267-01 | Wound Healing (Immune C1) |
| TCGA-CW-5588-01 | Inflammatory (Immune C3) |
| TCGA-D1-A16O-01 | Inflammatory (Immune C3) |
| TCGA-D8-A1JI-01 | Inflammatory (Immune C3) |
| TCGA-GM-A2D9-01 | Wound Healing (Immune C1) |
| TCGA-S9-A6WQ-01 | Immunologically Quiet (Immune C5) |
| TCGA-OL-A5RX-01 | Inflammatory (Immune C3) |
| TCGA-24-1850-01 | Wound Healing (Immune C1) |
| TCGA-41-4097-01 | Lymphocyte Depleted (Immune C4) |
| TCGA-A3-A8OU-01 | Inflammatory (Immune C3) |
| TCGA-EW-A1OY-01 | IFN-gamma Dominant (Immune C2) |
| TCGA-BD-A3EP-01 | Inflammatory (Immune C3) |
| TCGA-QH-A6CS-01 | Immunologically Quiet (Immune C5) |
| TCGA-E1-A7YU-01 | Immunologically Quiet (Immune C5) |
| TCGA-AR-A5QM-01 | TGF-beta Dominant (Immune C6) |
| TCGA-W9-A837-01 | Immunologically Quiet (Immune C5) |
| TCGA-KC-A4BN-01 | Inflammatory (Immune C3) |
| TCGA-HQ-A2OE-01 | Wound Healing (Immune C1) |
| TCGA-IK-7675-01 | Immunologically Quiet (Immune C5) |
| TCGA-SP-A6QG-01 | Inflammatory (Immune C3) |
| TCGA-D1-A3JQ-01 | IFN-gamma Dominant (Immune C2) |
| TCGA-F7-A624-01 | IFN-gamma Dominant (Immune C2) |
| TCGA-BJ-A45K-01 | Inflammatory (Immune C3) |
| TCGA-BH-A0BA-01 | Inflammatory (Immune C3) |
| TCGA-DJ-A2Q6-01 | Inflammatory (Immune C3) |
| TCGA-BP-5201-01 | Inflammatory (Immune C3) |
| TCGA-GN-A263-01 | IFN-gamma Dominant (Immune C2) |
| TCGA-05-4249-01 | Inflammatory (Immune C3) |
| TCGA-BJ-A3F0-01 | Inflammatory (Immune C3) |
| TCGA-34-5236-01 | Wound Healing (Immune C1) |
| TCGA-DU-7306-01 | Lymphocyte Depleted (Immune C4) |
| TCGA-C8-A26Z-01 | Wound Healing (Immune C1) |
| TCGA-ET-A39I-01 | Inflammatory (Immune C3) |
| TCGA-EM-A2CU-01 | Inflammatory (Immune C3) |
| TCGA-QH-A6X9-01 | Immunologically Quiet (Immune C5) |
| TCGA-G3-A7M7-01 | Lymphocyte Depleted (Immune C4) |
| TCGA-BS-A0U9-01 | IFN-gamma Dominant (Immune C2) |
| TCGA-BK-A13C-01 | Inflammatory (Immune C3) |
| TCGA-2Z-A9JG-01 | Inflammatory (Immune C3) |
| TCGA-L5-A4OW-01 | Wound Healing (Immune C1) |
| TCGA-UY-A78O-01 | Wound Healing (Immune C1) |
| TCGA-SX-A7SU-01 | Inflammatory (Immune C3) |
| TCGA-CW-5591-01 | Inflammatory (Immune C3) |
| TCGA-EK-A3GK-01 | IFN-gamma Dominant (Immune C2) |
| TCGA-2G-AAG5-01 | Wound Healing (Immune C1) |
| TCGA-IB-AAUM-01 | Inflammatory (Immune C3) |
| TCGA-4K-AA1G-01 | IFN-gamma Dominant (Immune C2) |
| TCGA-OL-A66H-01 | IFN-gamma Dominant (Immune C2) |
| TCGA-BT-A20T-01 | Wound Healing (Immune C1) |
| TCGA-PG-A5BC-01 | Wound Healing (Immune C1) |
| TCGA-30-1860-01 | IFN-gamma Dominant (Immune C2) |
| TCGA-90-A4ED-01 | Inflammatory (Immune C3) |
| TCGA-CV-7235-01 | IFN-gamma Dominant (Immune C2) |
| TCGA-ET-A25K-01 | Inflammatory (Immune C3) |
| TCGA-E2-A14S-01 | Lymphocyte Depleted (Immune C4) |
| TCGA-XX-A899-01 | Inflammatory (Immune C3) |
| TCGA-A8-A06O-01 | IFN-gamma Dominant (Immune C2) |
| TCGA-A3-3387-01 | Inflammatory (Immune C3) |
| TCGA-HT-7611-01 | Immunologically Quiet (Immune C5) |
| TCGA-D8-A1JM-01 | Wound Healing (Immune C1) |
| TCGA-22-4594-01 | IFN-gamma Dominant (Immune C2) |
| TCGA-55-6986-01 | Inflammatory (Immune C3) |
| TCGA-AA-3524-01 | Wound Healing (Immune C1) |
| TCGA-S9-A6TY-01 | Immunologically Quiet (Immune C5) |
| TCGA-DU-A7TB-01 | Immunologically Quiet (Immune C5) |
| TCGA-DC-6160-01 | Wound Healing (Immune C1) |
| TCGA-63-5128-01 | Wound Healing (Immune C1) |
| TCGA-5P-A9K8-01 | Immunologically Quiet (Immune C5) |
| TCGA-63-A5MU-01 | Wound Healing (Immune C1) |
| TCGA-CV-A6JM-01 | Wound Healing (Immune C1) |
| TCGA-E9-A228-01 | Lymphocyte Depleted (Immune C4) |
| TCGA-E9-A1RA-01 | Wound Healing (Immune C1) |
| TCGA-BR-8682-01 | Inflammatory (Immune C3) |
| TCGA-OL-A6VR-01 | Lymphocyte Depleted (Immune C4) |
| TCGA-O2-A52W-01 | Wound Healing (Immune C1) |
| TCGA-XE-A8H1-01 | IFN-gamma Dominant (Immune C2) |
| TCGA-BJ-A18Y-01 | Inflammatory (Immune C3) |
| TCGA-FB-A4P5-01 | TGF-beta Dominant (Immune C6) |
| TCGA-19-2625-01 | Lymphocyte Depleted (Immune C4) |
| TCGA-DJ-A2Q2-01 | Inflammatory (Immune C3) |
| TCGA-IB-A5SP-01 | TGF-beta Dominant (Immune C6) |
| TCGA-IK-8125-01 | Immunologically Quiet (Immune C5) |
| TCGA-AR-A1AW-01 | IFN-gamma Dominant (Immune C2) |
| TCGA-WH-A86K-01 | Immunologically Quiet (Immune C5) |
| TCGA-D5-6530-01 | IFN-gamma Dominant (Immune C2) |
| TCGA-YC-A8S6-01 | Wound Healing (Immune C1) |
| TCGA-MP-A4T9-01 | Inflammatory (Immune C3) |
| TCGA-61-1995-01 | Lymphocyte Depleted (Immune C4) |
| TCGA-21-1075-01 | Wound Healing (Immune C1) |
| TCGA-BH-A0H6-01 | Wound Healing (Immune C1) |
| TCGA-BR-4279-01 | IFN-gamma Dominant (Immune C2) |
| TCGA-YL-A8SP-01 | Lymphocyte Depleted (Immune C4) |
| TCGA-TS-A7P3-01 | Wound Healing (Immune C1) |
| TCGA-E7-A5KF-01 | Lymphocyte Depleted (Immune C4) |
| TCGA-W2-A7HH-01 | Inflammatory (Immune C3) |
| TCGA-B0-5106-01 | Inflammatory (Immune C3) |
| TCGA-B0-4843-01 | Inflammatory (Immune C3) |
| TCGA-HV-A7OL-01 | IFN-gamma Dominant (Immune C2) |
| TCGA-CK-5916-01 | IFN-gamma Dominant (Immune C2) |
| TCGA-DX-A3LW-01 | Wound Healing (Immune C1) |
| TCGA-A2-A04U-01 | Wound Healing (Immune C1) |
| TCGA-DH-5141-01 | Immunologically Quiet (Immune C5) |
| TCGA-BL-A13J-01 | Wound Healing (Immune C1) |
| TCGA-CV-5971-01 | Wound Healing (Immune C1) |
| TCGA-D8-A1JF-01 | Inflammatory (Immune C3) |
| TCGA-OL-A6VQ-01 | Inflammatory (Immune C3) |
| TCGA-QT-A5XK-01 | Lymphocyte Depleted (Immune C4) |
| TCGA-56-7582-01 | Wound Healing (Immune C1) |
| TCGA-G9-A9S0-01 | Wound Healing (Immune C1) |
| TCGA-S4-A8RO-01 | IFN-gamma Dominant (Immune C2) |
| TCGA-C8-A273-01 | IFN-gamma Dominant (Immune C2) |
| TCGA-AH-6644-01 | Wound Healing (Immune C1) |
| TCGA-BP-4769-01 | Inflammatory (Immune C3) |
| TCGA-ZG-A9LS-01 | Inflammatory (Immune C3) |
| TCGA-DJ-A2PT-01 | Inflammatory (Immune C3) |
| TCGA-BB-7864-01 | IFN-gamma Dominant (Immune C2) |
| TCGA-C8-A132-01 | Wound Healing (Immune C1) |
| TCGA-A5-A0R7-01 | IFN-gamma Dominant (Immune C2) |
| TCGA-E2-A10F-01 | Wound Healing (Immune C1) |
| TCGA-FG-A60L-01 | Lymphocyte Depleted (Immune C4) |
| TCGA-91-A4BD-01 | Lymphocyte Depleted (Immune C4) |
| TCGA-G2-A2EJ-01 | IFN-gamma Dominant (Immune C2) |
| TCGA-BP-4355-01 | Inflammatory (Immune C3) |
| TCGA-GF-A769-01 | Lymphocyte Depleted (Immune C4) |
| TCGA-DB-5280-01 | Immunologically Quiet (Immune C5) |
| TCGA-BH-A0EE-01 | IFN-gamma Dominant (Immune C2) |
| TCGA-QR-A6GU-01 | Inflammatory (Immune C3) |
| TCGA-BS-A0VI-01 | Wound Healing (Immune C1) |
| TCGA-FD-A3SN-01 | IFN-gamma Dominant (Immune C2) |
| TCGA-B6-A0RQ-01 | Inflammatory (Immune C3) |
| TCGA-BR-7717-01 | IFN-gamma Dominant (Immune C2) |
| TCGA-G4-6303-01 | Wound Healing (Immune C1) |
| TCGA-BR-8686-01 | Inflammatory (Immune C3) |
| TCGA-KL-8341-01 | Immunologically Quiet (Immune C5) |
| TCGA-DJ-A13M-01 | Inflammatory (Immune C3) |
| TCGA-MA-AA42-01 | IFN-gamma Dominant (Immune C2) |
| TCGA-XE-A8H5-01 | IFN-gamma Dominant (Immune C2) |
| TCGA-EM-A2CN-01 | Inflammatory (Immune C3) |
| TCGA-S9-A7IX-01 | Lymphocyte Depleted (Immune C4) |
| TCGA-DD-A3A2-01 | Inflammatory (Immune C3) |
| TCGA-3B-A9HO-01 | IFN-gamma Dominant (Immune C2) |
| TCGA-KS-A4I5-01 | Lymphocyte Depleted (Immune C4) |
| TCGA-CD-8534-01 | IFN-gamma Dominant (Immune C2) |
| TCGA-D8-A27W-01 | IFN-gamma Dominant (Immune C2) |
| TCGA-85-6560-01 | IFN-gamma Dominant (Immune C2) |
| TCGA-98-8022-01 | Inflammatory (Immune C3) |
| TCGA-25-1871-01 | Wound Healing (Immune C1) |
| TCGA-EI-6511-01 | IFN-gamma Dominant (Immune C2) |
| TCGA-C5-A8YQ-01 | Wound Healing (Immune C1) |
| TCGA-A8-A075-01 | Wound Healing (Immune C1) |
| TCGA-WC-A883-01 | Lymphocyte Depleted (Immune C4) |
| TCGA-04-1357-01 | IFN-gamma Dominant (Immune C2) |
| TCGA-CZ-5467-01 | Inflammatory (Immune C3) |
| TCGA-G2-A3VY-01 | Lymphocyte Depleted (Immune C4) |
| TCGA-51-4081-01 | Wound Healing (Immune C1) |
| TCGA-KC-A4BV-01 | Inflammatory (Immune C3) |
| TCGA-59-2350-01 | IFN-gamma Dominant (Immune C2) |
| TCGA-A2-A0CT-01 | Wound Healing (Immune C1) |
| TCGA-B7-A5TJ-01 | Wound Healing (Immune C1) |
| TCGA-32-1980-01 | Lymphocyte Depleted (Immune C4) |
| TCGA-AH-6643-01 | Wound Healing (Immune C1) |
| TCGA-FG-5965-01 | Immunologically Quiet (Immune C5) |
| TCGA-US-A77G-01 | Lymphocyte Depleted (Immune C4) |
| TCGA-VP-A875-01 | Lymphocyte Depleted (Immune C4) |
| TCGA-D6-A4Z9-01 | IFN-gamma Dominant (Immune C2) |
| TCGA-VQ-A8DT-01 | IFN-gamma Dominant (Immune C2) |
| TCGA-AZ-4682-01 | Wound Healing (Immune C1) |
| TCGA-QK-A6IH-01 | IFN-gamma Dominant (Immune C2) |
| TCGA-55-7907-01 | Inflammatory (Immune C3) |
| TCGA-VF-A8AD-01 | IFN-gamma Dominant (Immune C2) |
| TCGA-D1-A16G-01 | Wound Healing (Immune C1) |
| TCGA-HC-8262-01 | Inflammatory (Immune C3) |
| TCGA-QQ-A5VC-01 | Lymphocyte Depleted (Immune C4) |
| TCGA-95-7039-01 | Wound Healing (Immune C1) |
| TCGA-DX-A3M2-01 | IFN-gamma Dominant (Immune C2) |
| TCGA-46-6026-01 | Wound Healing (Immune C1) |
| TCGA-CL-4957-01 | Wound Healing (Immune C1) |
| TCGA-64-1680-01 | Inflammatory (Immune C3) |
| TCGA-BS-A0U5-01 | Wound Healing (Immune C1) |
| TCGA-SC-A6LR-01 | Inflammatory (Immune C3) |
| TCGA-ZF-AA4X-01 | Lymphocyte Depleted (Immune C4) |
| TCGA-CQ-7072-01 | Wound Healing (Immune C1) |
| TCGA-J2-A4AE-01 | Inflammatory (Immune C3) |
| TCGA-A2-A3Y0-01 | IFN-gamma Dominant (Immune C2) |
| TCGA-2G-AALS-01 | IFN-gamma Dominant (Immune C2) |
| TCGA-EJ-5501-01 | Inflammatory (Immune C3) |
| TCGA-22-5473-01 | Wound Healing (Immune C1) |
| TCGA-EW-A6SD-01 | Wound Healing (Immune C1) |
| TCGA-A7-A426-01 | Inflammatory (Immune C3) |
| TCGA-L5-A4OJ-01 | Wound Healing (Immune C1) |
| TCGA-EL-A3T6-01 | Inflammatory (Immune C3) |
| TCGA-06-0744-01 | Lymphocyte Depleted (Immune C4) |
| TCGA-EI-7004-01 | Wound Healing (Immune C1) |
| TCGA-E9-A1N6-01 | Lymphocyte Depleted (Immune C4) |
| TCGA-DQ-7589-01 | Wound Healing (Immune C1) |
| TCGA-X6-A8C7-01 | Inflammatory (Immune C3) |
| TCGA-L5-A88W-01 | Wound Healing (Immune C1) |
| TCGA-E2-A14W-01 | Wound Healing (Immune C1) |
| TCGA-KS-A4I9-01 | Inflammatory (Immune C3) |
| TCGA-SP-A6QK-01 | Inflammatory (Immune C3) |
| TCGA-AD-6890-01 | Wound Healing (Immune C1) |
| TCGA-CV-5440-01 | Wound Healing (Immune C1) |
| TCGA-LQ-A4E4-01 | Wound Healing (Immune C1) |
| TCGA-OR-A5LB-01 | Lymphocyte Depleted (Immune C4) |
| TCGA-HZ-8636-01 | TGF-beta Dominant (Immune C6) |
| TCGA-2G-AAKL-01 | IFN-gamma Dominant (Immune C2) |
| TCGA-XF-AAMY-01 | Wound Healing (Immune C1) |
| TCGA-5X-AA5U-01 | Wound Healing (Immune C1) |
| TCGA-AD-6889-01 | Wound Healing (Immune C1) |
| TCGA-AR-A256-01 | IFN-gamma Dominant (Immune C2) |
| TCGA-ED-A4XI-01 | Lymphocyte Depleted (Immune C4) |
| TCGA-B5-A0JU-01 | IFN-gamma Dominant (Immune C2) |
| TCGA-CR-7391-01 | IFN-gamma Dominant (Immune C2) |
| TCGA-KC-A7FD-01 | Wound Healing (Immune C1) |
| TCGA-FY-A3NN-01 | Inflammatory (Immune C3) |
| TCGA-AG-A025-01 | Wound Healing (Immune C1) |
| TCGA-D1-A176-01 | Inflammatory (Immune C3) |
| TCGA-EY-A1GV-01 | Wound Healing (Immune C1) |
| TCGA-P5-A5F6-01 | Immunologically Quiet (Immune C5) |
| TCGA-A2-A0T3-01 | Wound Healing (Immune C1) |
| TCGA-2Z-A9JT-01 | Inflammatory (Immune C3) |
| TCGA-VD-AA8S-01 | Lymphocyte Depleted (Immune C4) |
| TCGA-FD-A5C0-01 | Wound Healing (Immune C1) |
| TCGA-86-8359-01 | Inflammatory (Immune C3) |
| TCGA-21-1079-01 | IFN-gamma Dominant (Immune C2) |
| TCGA-WZ-A7V3-01 | IFN-gamma Dominant (Immune C2) |
| TCGA-BH-A0HN-01 | Lymphocyte Depleted (Immune C4) |
| TCGA-CN-5369-01 | IFN-gamma Dominant (Immune C2) |
| TCGA-DB-A64W-01 | Lymphocyte Depleted (Immune C4) |
| TCGA-K7-A5RF-01 | Lymphocyte Depleted (Immune C4) |
| TCGA-EY-A215-01 | Wound Healing (Immune C1) |
| TCGA-55-8614-01 | Wound Healing (Immune C1) |
| TCGA-XK-AAJP-01 | Inflammatory (Immune C3) |
| TCGA-AO-A03O-01 | IFN-gamma Dominant (Immune C2) |
| TCGA-AW-A1PO-01 | IFN-gamma Dominant (Immune C2) |
| TCGA-AA-3858-01 | Wound Healing (Immune C1) |
| TCGA-55-7574-01 | Inflammatory (Immune C3) |
| TCGA-D1-A16S-01 | IFN-gamma Dominant (Immune C2) |
| TCGA-CV-A45T-01 | IFN-gamma Dominant (Immune C2) |
| TCGA-27-1834-01 | Lymphocyte Depleted (Immune C4) |
| TCGA-AC-A23G-01 | Wound Healing (Immune C1) |
| TCGA-ED-A8O5-01 | Inflammatory (Immune C3) |
| TCGA-29-2425-01 | Wound Healing (Immune C1) |
| TCGA-AG-A01L-01 | Wound Healing (Immune C1) |
| TCGA-CV-6934-01 | IFN-gamma Dominant (Immune C2) |
| TCGA-IB-A7M4-01 | IFN-gamma Dominant (Immune C2) |
| TCGA-XF-A8HI-01 | Wound Healing (Immune C1) |
| TCGA-DX-A8BS-01 | Lymphocyte Depleted (Immune C4) |
| TCGA-HC-A76W-01 | Inflammatory (Immune C3) |
| TCGA-DU-7007-01 | Lymphocyte Depleted (Immune C4) |
| TCGA-G9-A9S7-01 | Inflammatory (Immune C3) |
| TCGA-HC-8216-01 | Inflammatory (Immune C3) |
| TCGA-MP-A4TI-01 | IFN-gamma Dominant (Immune C2) |
| TCGA-BB-7862-01 | Wound Healing (Immune C1) |
| TCGA-EJ-A8FN-01 | Inflammatory (Immune C3) |
| TCGA-2G-AAFG-01 | IFN-gamma Dominant (Immune C2) |
| TCGA-GV-A40E-01 | IFN-gamma Dominant (Immune C2) |
| TCGA-RS-A6TP-01 | IFN-gamma Dominant (Immune C2) |
| TCGA-73-4677-01 | Inflammatory (Immune C3) |
| TCGA-EW-A1J3-01 | Wound Healing (Immune C1) |
| TCGA-ZJ-AB0H-01 | IFN-gamma Dominant (Immune C2) |
| TCGA-HW-7490-01 | Lymphocyte Depleted (Immune C4) |
| TCGA-D6-A6EM-01 | IFN-gamma Dominant (Immune C2) |
| TCGA-CS-6188-01 | Lymphocyte Depleted (Immune C4) |
| TCGA-TM-A7C3-01 | Lymphocyte Depleted (Immune C4) |
| TCGA-A7-A0CD-01 | Lymphocyte Depleted (Immune C4) |
| TCGA-DX-A3UA-01 | Wound Healing (Immune C1) |
| TCGA-BG-A0RY-01 | Lymphocyte Depleted (Immune C4) |
| TCGA-68-A59I-01 | Wound Healing (Immune C1) |
| TCGA-V4-A9F8-01 | Inflammatory (Immune C3) |
| TCGA-CS-6668-01 | Immunologically Quiet (Immune C5) |
| TCGA-BH-A208-01 | Wound Healing (Immune C1) |
| TCGA-BH-A1F6-01 | IFN-gamma Dominant (Immune C2) |
| TCGA-BA-A6DB-01 | IFN-gamma Dominant (Immune C2) |
| TCGA-C5-A7X5-01 | IFN-gamma Dominant (Immune C2) |
| TCGA-CF-A47W-01 | Lymphocyte Depleted (Immune C4) |
| TCGA-EL-A3ZH-01 | Inflammatory (Immune C3) |
| TCGA-XF-AAN2-01 | IFN-gamma Dominant (Immune C2) |
| TCGA-58-A46M-01 | IFN-gamma Dominant (Immune C2) |
| TCGA-BS-A0TD-01 | Wound Healing (Immune C1) |
| TCGA-VS-A9U5-01 | IFN-gamma Dominant (Immune C2) |
| TCGA-98-8021-01 | Wound Healing (Immune C1) |
| TCGA-DC-6154-01 | Wound Healing (Immune C1) |
| TCGA-E9-A1NE-01 | IFN-gamma Dominant (Immune C2) |
| TCGA-ZP-A9D1-01 | Inflammatory (Immune C3) |
| TCGA-22-5481-01 | IFN-gamma Dominant (Immune C2) |
| TCGA-DX-A3UE-01 | Lymphocyte Depleted (Immune C4) |
| TCGA-DD-AADU-01 | Inflammatory (Immune C3) |
| TCGA-EB-A6QY-01 | Wound Healing (Immune C1) |
| TCGA-AR-A24X-01 | Wound Healing (Immune C1) |
| TCGA-BG-A0MT-01 | Lymphocyte Depleted (Immune C4) |
| TCGA-29-1701-01 | IFN-gamma Dominant (Immune C2) |
| TCGA-B6-A0IO-01 | Wound Healing (Immune C1) |
| TCGA-FP-8210-01 | Inflammatory (Immune C3) |
| TCGA-QG-A5Z2-01 | IFN-gamma Dominant (Immune C2) |
| TCGA-BJ-A191-01 | Inflammatory (Immune C3) |
| TCGA-HB-A5W3-01 | Inflammatory (Immune C3) |
| TCGA-D6-8569-01 | IFN-gamma Dominant (Immune C2) |
| TCGA-A5-A0GP-01 | Wound Healing (Immune C1) |
| TCGA-2A-AAYO-01 | IFN-gamma Dominant (Immune C2) |
| TCGA-E2-A1IE-01 | Wound Healing (Immune C1) |
| TCGA-G9-6364-01 | Inflammatory (Immune C3) |
| TCGA-L5-A8NK-01 | IFN-gamma Dominant (Immune C2) |
| TCGA-G4-6294-01 | Wound Healing (Immune C1) |
| TCGA-AP-A0LD-01 | IFN-gamma Dominant (Immune C2) |
| TCGA-2G-AAFL-01 | IFN-gamma Dominant (Immune C2) |
| TCGA-NA-A4R1-01 | Wound Healing (Immune C1) |
| TCGA-66-2769-01 | Wound Healing (Immune C1) |
| TCGA-UD-AAC6-01 | Lymphocyte Depleted (Immune C4) |
| TCGA-FD-A3B5-01 | IFN-gamma Dominant (Immune C2) |
| TCGA-4Z-AA80-01 | IFN-gamma Dominant (Immune C2) |
| TCGA-78-7167-01 | Inflammatory (Immune C3) |
| TCGA-78-8662-01 | Wound Healing (Immune C1) |
| TCGA-CF-A3MF-01 | Inflammatory (Immune C3) |
| TCGA-B8-5165-01 | Inflammatory (Immune C3) |
| TCGA-C5-A1M7-01 | IFN-gamma Dominant (Immune C2) |
| TCGA-DU-8165-01 | Lymphocyte Depleted (Immune C4) |
| TCGA-86-8585-01 | IFN-gamma Dominant (Immune C2) |
| TCGA-BP-4985-01 | Wound Healing (Immune C1) |
| TCGA-AN-A0AJ-01 | IFN-gamma Dominant (Immune C2) |
| TCGA-WK-A8XX-01 | Lymphocyte Depleted (Immune C4) |
| TCGA-BQ-7053-01 | Inflammatory (Immune C3) |
| TCGA-AR-A24T-01 | TGF-beta Dominant (Immune C6) |
| TCGA-24-1428-01 | IFN-gamma Dominant (Immune C2) |
| TCGA-ET-A4KQ-01 | Inflammatory (Immune C3) |
| TCGA-QG-A5Z1-01 | Wound Healing (Immune C1) |
| TCGA-23-1120-01 | IFN-gamma Dominant (Immune C2) |
| TCGA-D5-6923-01 | Wound Healing (Immune C1) |
| TCGA-BR-A4PD-01 | IFN-gamma Dominant (Immune C2) |
| TCGA-BH-A1F2-01 | Lymphocyte Depleted (Immune C4) |
| TCGA-24-2038-01 | Lymphocyte Depleted (Immune C4) |
| TCGA-G9-6371-01 | Lymphocyte Depleted (Immune C4) |
| TCGA-DX-A7EF-01 | Wound Healing (Immune C1) |
| TCGA-EM-A4FH-01 | Inflammatory (Immune C3) |
| TCGA-BR-A44U-01 | IFN-gamma Dominant (Immune C2) |
| TCGA-KU-A66S-01 | IFN-gamma Dominant (Immune C2) |
| TCGA-D8-A1J9-01 | Lymphocyte Depleted (Immune C4) |
| TCGA-AP-A0LH-01 | IFN-gamma Dominant (Immune C2) |
| TCGA-Q1-A5R2-01 | IFN-gamma Dominant (Immune C2) |
| TCGA-IB-AAUR-01 | TGF-beta Dominant (Immune C6) |
| TCGA-HU-A4GH-01 | Wound Healing (Immune C1) |
| TCGA-2X-A9D5-01 | IFN-gamma Dominant (Immune C2) |
| TCGA-DD-AADY-01 | Inflammatory (Immune C3) |
| TCGA-HU-A4GT-01 | IFN-gamma Dominant (Immune C2) |
| TCGA-HT-7692-01 | Immunologically Quiet (Immune C5) |
| TCGA-CF-A3MI-01 | Inflammatory (Immune C3) |
| TCGA-DJ-A2Q1-01 | Inflammatory (Immune C3) |
| TCGA-B0-5099-01 | Inflammatory (Immune C3) |
| TCGA-G4-6628-01 | IFN-gamma Dominant (Immune C2) |
| TCGA-G2-A3IE-01 | Inflammatory (Immune C3) |
| TCGA-61-1914-01 | IFN-gamma Dominant (Immune C2) |
| TCGA-CZ-5988-01 | Inflammatory (Immune C3) |
| TCGA-29-1705-01 | Lymphocyte Depleted (Immune C4) |
| TCGA-BG-A0VT-01 | Wound Healing (Immune C1) |
| TCGA-77-7338-01 | Wound Healing (Immune C1) |
| TCGA-EM-A22K-01 | Inflammatory (Immune C3) |
| TCGA-E2-A150-01 | IFN-gamma Dominant (Immune C2) |
| TCGA-AR-A251-01 | Wound Healing (Immune C1) |
| TCGA-EL-A3GR-01 | Inflammatory (Immune C3) |
| TCGA-51-6867-01 | IFN-gamma Dominant (Immune C2) |
| TCGA-09-1673-01 | Lymphocyte Depleted (Immune C4) |
| TCGA-SR-A6MX-01 | Inflammatory (Immune C3) |
| TCGA-TQ-A7RM-01 | Lymphocyte Depleted (Immune C4) |
| TCGA-MU-A51Y-01 | IFN-gamma Dominant (Immune C2) |
| TCGA-AO-A128-01 | IFN-gamma Dominant (Immune C2) |
| TCGA-AA-A01Q-01 | Wound Healing (Immune C1) |
| TCGA-VS-A8EB-01 | Wound Healing (Immune C1) |
| TCGA-EY-A1GM-01 | IFN-gamma Dominant (Immune C2) |
| TCGA-V4-A9F1-01 | Lymphocyte Depleted (Immune C4) |
| TCGA-FY-A40L-01 | Inflammatory (Immune C3) |
| TCGA-AF-5654-01 | Wound Healing (Immune C1) |
| TCGA-4Z-AA84-01 | IFN-gamma Dominant (Immune C2) |
| TCGA-78-8655-01 | Inflammatory (Immune C3) |
| TCGA-78-7163-01 | Inflammatory (Immune C3) |
| TCGA-2Y-A9GS-01 | Lymphocyte Depleted (Immune C4) |
| TCGA-KL-8336-01 | Inflammatory (Immune C3) |
| TCGA-DU-8161-01 | Lymphocyte Depleted (Immune C4) |
| TCGA-F1-6874-01 | Wound Healing (Immune C1) |
| TCGA-D8-A1JE-01 | Lymphocyte Depleted (Immune C4) |
| TCGA-ZF-A9R2-01 | Wound Healing (Immune C1) |
| TCGA-CG-5717-01 | IFN-gamma Dominant (Immune C2) |
| TCGA-LN-A9FP-01 | Inflammatory (Immune C3) |
| TCGA-JY-A6FB-01 | Lymphocyte Depleted (Immune C4) |
| TCGA-LP-A4AX-01 | IFN-gamma Dominant (Immune C2) |
| TCGA-2G-AALG-01 | Wound Healing (Immune C1) |
| TCGA-05-4417-01 | Inflammatory (Immune C3) |
| TCGA-A2-A0CP-01 | Wound Healing (Immune C1) |
| TCGA-2J-AABO-01 | Wound Healing (Immune C1) |
| TCGA-97-7554-01 | IFN-gamma Dominant (Immune C2) |
| TCGA-DD-AADN-01 | IFN-gamma Dominant (Immune C2) |
| TCGA-BB-8601-01 | Wound Healing (Immune C1) |
| TCGA-KN-8431-01 | Lymphocyte Depleted (Immune C4) |
| TCGA-P5-A736-01 | Immunologically Quiet (Immune C5) |
| TCGA-2Y-A9H9-01 | Lymphocyte Depleted (Immune C4) |
| TCGA-EL-A4K9-01 | Inflammatory (Immune C3) |
| TCGA-BQ-7045-01 | Inflammatory (Immune C3) |
| TCGA-23-2077-01 | IFN-gamma Dominant (Immune C2) |
| TCGA-ZU-A8S4-01 | Wound Healing (Immune C1) |
| TCGA-IN-A7NU-01 | IFN-gamma Dominant (Immune C2) |
| TCGA-RC-A7SK-01 | Lymphocyte Depleted (Immune C4) |
| TCGA-EO-A3AS-01 | Wound Healing (Immune C1) |
| TCGA-AL-3472-01 | Inflammatory (Immune C3) |
| TCGA-AX-A1C4-01 | Wound Healing (Immune C1) |
| TCGA-J8-A3YE-01 | Inflammatory (Immune C3) |
| TCGA-43-A56U-01 | IFN-gamma Dominant (Immune C2) |
| TCGA-EM-A3FP-01 | Inflammatory (Immune C3) |
| TCGA-BH-A5IZ-01 | Wound Healing (Immune C1) |
| TCGA-49-4505-01 | IFN-gamma Dominant (Immune C2) |
| TCGA-AK-3431-01 | Inflammatory (Immune C3) |
| TCGA-LB-A8F3-01 | Wound Healing (Immune C1) |
| TCGA-AA-3875-01 | IFN-gamma Dominant (Immune C2) |
| TCGA-06-0141-01 | Lymphocyte Depleted (Immune C4) |
| TCGA-EJ-5518-01 | Inflammatory (Immune C3) |
| TCGA-B8-A54I-01 | Inflammatory (Immune C3) |
| TCGA-SR-A6MT-01 | Inflammatory (Immune C3) |
| TCGA-GV-A3QG-01 | IFN-gamma Dominant (Immune C2) |
| TCGA-WB-A80Y-01 | Inflammatory (Immune C3) |
| TCGA-DX-A8BT-01 | Wound Healing (Immune C1) |
| TCGA-W5-AA2Z-01 | TGF-beta Dominant (Immune C6) |
| TCGA-A8-A076-01 | IFN-gamma Dominant (Immune C2) |
| TCGA-VS-A9UM-01 | IFN-gamma Dominant (Immune C2) |
| TCGA-D6-A6EN-01 | IFN-gamma Dominant (Immune C2) |
| TCGA-LL-A442-01 | Lymphocyte Depleted (Immune C4) |
| TCGA-DU-6401-01 | Immunologically Quiet (Immune C5) |
| TCGA-46-3768-01 | Lymphocyte Depleted (Immune C4) |
| TCGA-2G-AAGO-01 | IFN-gamma Dominant (Immune C2) |
| TCGA-22-5489-01 | IFN-gamma Dominant (Immune C2) |
| TCGA-DK-A6AV-01 | Wound Healing (Immune C1) |
| TCGA-LN-A7HV-01 | Wound Healing (Immune C1) |
| TCGA-A3-3311-01 | Inflammatory (Immune C3) |
| TCGA-G7-A8LC-01 | Inflammatory (Immune C3) |
| TCGA-CC-A8HT-01 | Lymphocyte Depleted (Immune C4) |
| TCGA-DX-A6BH-01 | Wound Healing (Immune C1) |
| TCGA-VQ-A91K-01 | IFN-gamma Dominant (Immune C2) |
| TCGA-B6-A40B-01 | Wound Healing (Immune C1) |
| TCGA-41-2571-01 | Lymphocyte Depleted (Immune C4) |
| TCGA-33-AASJ-01 | IFN-gamma Dominant (Immune C2) |
| TCGA-BR-6453-01 | IFN-gamma Dominant (Immune C2) |
| TCGA-ED-A459-01 | Lymphocyte Depleted (Immune C4) |
| TCGA-DM-A1DB-01 | Wound Healing (Immune C1) |
| TCGA-HF-7136-01 | Wound Healing (Immune C1) |
| TCGA-AG-3893-01 | Wound Healing (Immune C1) |
| TCGA-2Z-A9J1-01 | Inflammatory (Immune C3) |
| TCGA-TT-A6YN-01 | Immunologically Quiet (Immune C5) |
| TCGA-DK-AA6W-01 | Wound Healing (Immune C1) |
| TCGA-QR-A6H6-01 | Inflammatory (Immune C3) |
| TCGA-G7-6793-01 | Inflammatory (Immune C3) |
| TCGA-AX-A2HG-01 | Wound Healing (Immune C1) |
| TCGA-IB-7644-01 | Wound Healing (Immune C1) |
| TCGA-P4-A5E7-01 | Inflammatory (Immune C3) |
| TCGA-CV-A465-01 | IFN-gamma Dominant (Immune C2) |
| TCGA-R3-A69X-01 | Wound Healing (Immune C1) |
| TCGA-FE-A232-01 | Inflammatory (Immune C3) |
| TCGA-3A-A9I7-01 | TGF-beta Dominant (Immune C6) |
| TCGA-BR-4253-01 | IFN-gamma Dominant (Immune C2) |
| TCGA-TQ-A7RV-01 | Immunologically Quiet (Immune C5) |
| TCGA-BQ-7058-01 | Inflammatory (Immune C3) |
| TCGA-66-2765-01 | IFN-gamma Dominant (Immune C2) |
| TCGA-2G-AAH8-01 | IFN-gamma Dominant (Immune C2) |
| TCGA-B6-A0IH-01 | Inflammatory (Immune C3) |
| TCGA-A6-5659-01 | Wound Healing (Immune C1) |
| TCGA-BR-6563-01 | Inflammatory (Immune C3) |
| TCGA-D8-A27K-01 | Inflammatory (Immune C3) |
| TCGA-31-1944-01 | Wound Healing (Immune C1) |
| TCGA-BP-4761-01 | Inflammatory (Immune C3) |
| TCGA-AP-A05D-01 | IFN-gamma Dominant (Immune C2) |
| TCGA-S7-A7WT-01 | Inflammatory (Immune C3) |
| TCGA-MJ-A68H-01 | Wound Healing (Immune C1) |
| TCGA-BR-A4J4-01 | Wound Healing (Immune C1) |
| TCGA-V4-A9EY-01 | Lymphocyte Depleted (Immune C4) |
| TCGA-DU-5871-01 | Immunologically Quiet (Immune C5) |
| TCGA-22-0944-01 | IFN-gamma Dominant (Immune C2) |
| TCGA-BH-A0E9-01 | Inflammatory (Immune C3) |
| TCGA-86-8669-01 | Inflammatory (Immune C3) |
| TCGA-59-2351-01 | IFN-gamma Dominant (Immune C2) |
| TCGA-BR-6803-01 | Inflammatory (Immune C3) |
| TCGA-14-0781-01 | Lymphocyte Depleted (Immune C4) |
| TCGA-HT-7483-01 | Immunologically Quiet (Immune C5) |
| TCGA-HT-7882-01 | TGF-beta Dominant (Immune C6) |
| TCGA-E2-A574-01 | Wound Healing (Immune C1) |
| TCGA-FD-A3B6-01 | IFN-gamma Dominant (Immune C2) |
| TCGA-ZF-AA5P-01 | Wound Healing (Immune C1) |
| TCGA-SG-A849-01 | IFN-gamma Dominant (Immune C2) |
| TCGA-E9-A1NH-01 | Inflammatory (Immune C3) |
| TCGA-A5-A0G9-01 | Wound Healing (Immune C1) |
| TCGA-VS-A94Z-01 | IFN-gamma Dominant (Immune C2) |
| TCGA-D5-6927-01 | Wound Healing (Immune C1) |
| TCGA-EW-A2FW-01 | Wound Healing (Immune C1) |
| TCGA-31-1951-01 | IFN-gamma Dominant (Immune C2) |
| TCGA-CD-8530-01 | Wound Healing (Immune C1) |
| TCGA-CJ-4902-01 | Inflammatory (Immune C3) |
| TCGA-66-2766-01 | Wound Healing (Immune C1) |
| TCGA-55-6975-01 | IFN-gamma Dominant (Immune C2) |
| TCGA-2G-AAGC-01 | IFN-gamma Dominant (Immune C2) |
| TCGA-DW-7840-01 | Inflammatory (Immune C3) |
| TCGA-09-1659-01 | IFN-gamma Dominant (Immune C2) |
| TCGA-A7-A3IY-01 | Lymphocyte Depleted (Immune C4) |
| TCGA-CC-A1HT-01 | IFN-gamma Dominant (Immune C2) |
| TCGA-77-8156-01 | Wound Healing (Immune C1) |
| TCGA-DJ-A2PP-01 | Inflammatory (Immune C3) |
| TCGA-G3-AAUZ-01 | IFN-gamma Dominant (Immune C2) |
| TCGA-CX-7085-01 | Wound Healing (Immune C1) |
| TCGA-BR-6457-01 | Inflammatory (Immune C3) |
| TCGA-AO-A0J8-01 | IFN-gamma Dominant (Immune C2) |
| TCGA-61-1918-01 | Lymphocyte Depleted (Immune C4) |
| TCGA-HF-7132-01 | IFN-gamma Dominant (Immune C2) |
| TCGA-AG-3882-01 | Inflammatory (Immune C3) |
| TCGA-A6-5660-01 | Wound Healing (Immune C1) |
| TCGA-DJ-A1QG-01 | Inflammatory (Immune C3) |
| TCGA-IF-A3RQ-01 | Lymphocyte Depleted (Immune C4) |
| TCGA-V4-A9EU-01 | Inflammatory (Immune C3) |
| TCGA-D6-6825-01 | IFN-gamma Dominant (Immune C2) |
| TCGA-HT-7620-01 | Immunologically Quiet (Immune C5) |
| TCGA-2G-AAH3-01 | IFN-gamma Dominant (Immune C2) |
| TCGA-DO-A2HM-01 | Inflammatory (Immune C3) |
| TCGA-ET-A25O-01 | Inflammatory (Immune C3) |
| TCGA-AX-A2HK-01 | IFN-gamma Dominant (Immune C2) |
| TCGA-B5-A11L-01 | Wound Healing (Immune C1) |
| TCGA-06-0157-01 | Lymphocyte Depleted (Immune C4) |
| TCGA-B0-4694-01 | Inflammatory (Immune C3) |
| TCGA-BR-8592-01 | Inflammatory (Immune C3) |
| TCGA-RC-A7SB-01 | Lymphocyte Depleted (Immune C4) |
| TCGA-CM-5862-01 | Wound Healing (Immune C1) |
| TCGA-D7-A6F0-01 | Wound Healing (Immune C1) |
| TCGA-EK-A3GM-01 | Lymphocyte Depleted (Immune C4) |
| TCGA-2G-AAG7-01 | Wound Healing (Immune C1) |
| TCGA-S9-A7J0-01 | Lymphocyte Depleted (Immune C4) |
| TCGA-AA-3561-01 | Wound Healing (Immune C1) |
| TCGA-B1-5398-01 | Inflammatory (Immune C3) |
| TCGA-B7-5816-01 | IFN-gamma Dominant (Immune C2) |
| TCGA-B8-A54F-01 | Inflammatory (Immune C3) |
| TCGA-A7-A3IZ-01 | Inflammatory (Immune C3) |
| TCGA-G3-AAV6-01 | Wound Healing (Immune C1) |
| TCGA-BJ-A3PU-01 | Inflammatory (Immune C3) |
| TCGA-A2-A04W-01 | Wound Healing (Immune C1) |
| TCGA-55-8208-01 | IFN-gamma Dominant (Immune C2) |
| TCGA-EY-A5W2-01 | Wound Healing (Immune C1) |
| TCGA-PG-A6IB-01 | IFN-gamma Dominant (Immune C2) |
| TCGA-D1-A17T-01 | IFN-gamma Dominant (Immune C2) |
| TCGA-XK-AAJA-01 | Inflammatory (Immune C3) |
| TCGA-F4-6805-01 | Wound Healing (Immune C1) |
| TCGA-12-3653-01 | Lymphocyte Depleted (Immune C4) |
| TCGA-DX-AB2H-01 | Wound Healing (Immune C1) |
| TCGA-B5-A1MU-01 | IFN-gamma Dominant (Immune C2) |
| TCGA-A2-A0D2-01 | Wound Healing (Immune C1) |
| TCGA-A2-A1G0-01 | Inflammatory (Immune C3) |
| TCGA-WX-AA46-01 | Inflammatory (Immune C3) |
| TCGA-2G-AAGT-01 | Wound Healing (Immune C1) |
| TCGA-E8-A415-01 | Inflammatory (Immune C3) |
| TCGA-D1-A1NS-01 | Wound Healing (Immune C1) |
| TCGA-CQ-A4CA-01 | IFN-gamma Dominant (Immune C2) |
| TCGA-BK-A6W4-01 | Wound Healing (Immune C1) |
| TCGA-TM-A84Q-01 | Immunologically Quiet (Immune C5) |
| TCGA-XF-AAMW-01 | IFN-gamma Dominant (Immune C2) |
| TCGA-DD-A73F-01 | IFN-gamma Dominant (Immune C2) |
| TCGA-WC-A87Y-01 | Inflammatory (Immune C3) |
| TCGA-A3-3365-01 | Inflammatory (Immune C3) |
| TCGA-76-4926-01 | Lymphocyte Depleted (Immune C4) |
| TCGA-UZ-A9PN-01 | Inflammatory (Immune C3) |
| TCGA-75-6206-01 | Inflammatory (Immune C3) |
| TCGA-36-1571-01 | IFN-gamma Dominant (Immune C2) |
| TCGA-D8-A1XZ-01 | IFN-gamma Dominant (Immune C2) |
| TCGA-CV-7407-01 | IFN-gamma Dominant (Immune C2) |
| TCGA-FV-A2QQ-01 | Inflammatory (Immune C3) |
| TCGA-55-6971-01 | Inflammatory (Immune C3) |
| TCGA-05-4410-01 | Inflammatory (Immune C3) |
| TCGA-BR-8487-01 | Wound Healing (Immune C1) |
| TCGA-AP-A05O-01 | IFN-gamma Dominant (Immune C2) |
| TCGA-AR-A0TY-01 | Wound Healing (Immune C1) |
| TCGA-26-5133-01 | Lymphocyte Depleted (Immune C4) |
| TCGA-VD-AA8O-01 | Inflammatory (Immune C3) |
| TCGA-A2-A0SW-01 | IFN-gamma Dominant (Immune C2) |
| TCGA-AA-3543-01 | IFN-gamma Dominant (Immune C2) |
| TCGA-IM-A3U2-01 | Inflammatory (Immune C3) |
| TCGA-36-1568-01 | IFN-gamma Dominant (Immune C2) |
| TCGA-EU-5904-01 | Inflammatory (Immune C3) |
| TCGA-55-8207-01 | Inflammatory (Immune C3) |
| TCGA-EJ-5514-01 | Inflammatory (Immune C3) |
| TCGA-OL-A5DA-01 | Inflammatory (Immune C3) |
| TCGA-R6-A6L6-01 | Wound Healing (Immune C1) |
| TCGA-A7-A5ZV-01 | Lymphocyte Depleted (Immune C4) |
| TCGA-B5-A11H-01 | IFN-gamma Dominant (Immune C2) |
| TCGA-LL-A50Y-01 | Wound Healing (Immune C1) |
| TCGA-06-2562-01 | Lymphocyte Depleted (Immune C4) |
| TCGA-UN-AAZ9-01 | Lymphocyte Depleted (Immune C4) |
| TCGA-BR-7707-01 | IFN-gamma Dominant (Immune C2) |
| TCGA-KM-8639-01 | Inflammatory (Immune C3) |
| TCGA-AX-A3FT-01 | IFN-gamma Dominant (Immune C2) |
| TCGA-BP-5007-01 | Inflammatory (Immune C3) |
| TCGA-EJ-7794-01 | Inflammatory (Immune C3) |
| TCGA-KS-A41J-01 | Inflammatory (Immune C3) |
| TCGA-A3-3374-01 | Lymphocyte Depleted (Immune C4) |
| TCGA-CK-4951-01 | IFN-gamma Dominant (Immune C2) |
| TCGA-BP-5180-01 | Inflammatory (Immune C3) |
| TCGA-EY-A2OQ-01 | Wound Healing (Immune C1) |
| TCGA-FV-A3R3-01 | Inflammatory (Immune C3) |
| TCGA-D7-6524-01 | Wound Healing (Immune C1) |
| TCGA-F4-6704-01 | Wound Healing (Immune C1) |
| TCGA-A5-A7WK-01 | IFN-gamma Dominant (Immune C2) |
| TCGA-CZ-5984-01 | Inflammatory (Immune C3) |
| TCGA-EB-A4IQ-01 | Wound Healing (Immune C1) |
| TCGA-F1-A72C-01 | IFN-gamma Dominant (Immune C2) |
| TCGA-BH-A0HW-01 | Lymphocyte Depleted (Immune C4) |
| TCGA-C5-A1MQ-01 | IFN-gamma Dominant (Immune C2) |
| TCGA-CN-A49A-01 | IFN-gamma Dominant (Immune C2) |
| TCGA-BH-A0BP-01 | Wound Healing (Immune C1) |
| TCGA-2F-A9KR-01 | Wound Healing (Immune C1) |
| TCGA-ET-A2N3-01 | Inflammatory (Immune C3) |
| TCGA-B3-4103-01 | Inflammatory (Immune C3) |
| TCGA-BJ-A190-01 | Inflammatory (Immune C3) |
| TCGA-RY-A847-01 | Immunologically Quiet (Immune C5) |
| TCGA-A2-A0EO-01 | Inflammatory (Immune C3) |
| TCGA-25-1634-01 | Wound Healing (Immune C1) |
| TCGA-B0-5083-01 | Inflammatory (Immune C3) |
| TCGA-BR-A44T-01 | IFN-gamma Dominant (Immune C2) |
| TCGA-EJ-5517-01 | Inflammatory (Immune C3) |
| TCGA-BK-A0CA-01 | Wound Healing (Immune C1) |
| TCGA-WK-A8XS-01 | Wound Healing (Immune C1) |
| TCGA-CW-5581-01 | Inflammatory (Immune C3) |
| TCGA-66-2727-01 | IFN-gamma Dominant (Immune C2) |
| TCGA-BR-8284-01 | IFN-gamma Dominant (Immune C2) |
| TCGA-C8-A274-01 | Lymphocyte Depleted (Immune C4) |
| TCGA-HC-A8D0-01 | Inflammatory (Immune C3) |
| TCGA-AR-A1AK-01 | IFN-gamma Dominant (Immune C2) |
| TCGA-HU-A4GX-01 | IFN-gamma Dominant (Immune C2) |
| TCGA-WZ-A8D5-01 | IFN-gamma Dominant (Immune C2) |
| TCGA-5R-AA1D-01 | Inflammatory (Immune C3) |
| TCGA-BJ-A28T-01 | Inflammatory (Immune C3) |
| TCGA-BT-A20R-01 | IFN-gamma Dominant (Immune C2) |
| TCGA-98-A53C-01 | Inflammatory (Immune C3) |
| TCGA-A6-6138-01 | IFN-gamma Dominant (Immune C2) |
| TCGA-22-0940-01 | IFN-gamma Dominant (Immune C2) |
| TCGA-DD-AAE7-01 | Inflammatory (Immune C3) |
| TCGA-HS-A5N7-01 | Wound Healing (Immune C1) |
| TCGA-DS-A0VK-01 | Wound Healing (Immune C1) |
| TCGA-TQ-A7RQ-01 | Immunologically Quiet (Immune C5) |
| TCGA-AC-A2QH-01 | Lymphocyte Depleted (Immune C4) |
| TCGA-BG-A2AE-01 | Wound Healing (Immune C1) |
| TCGA-90-7964-01 | Wound Healing (Immune C1) |
| TCGA-L4-A4E5-01 | Wound Healing (Immune C1) |
| TCGA-L5-A4OE-01 | IFN-gamma Dominant (Immune C2) |
| TCGA-CQ-A4C9-01 | IFN-gamma Dominant (Immune C2) |
| TCGA-SN-A84X-01 | IFN-gamma Dominant (Immune C2) |
| TCGA-DZ-6135-01 | Inflammatory (Immune C3) |
| TCGA-L5-A4OH-01 | IFN-gamma Dominant (Immune C2) |
| TCGA-EX-A1H6-01 | IFN-gamma Dominant (Immune C2) |
| TCGA-LB-A7SX-01 | Wound Healing (Immune C1) |
| TCGA-58-8393-01 | IFN-gamma Dominant (Immune C2) |
| TCGA-63-A5MS-01 | TGF-beta Dominant (Immune C6) |
| TCGA-AD-A5EJ-01 | Wound Healing (Immune C1) |
| TCGA-A3-3378-01 | Inflammatory (Immune C3) |
| TCGA-BR-A4J7-01 | Inflammatory (Immune C3) |
| TCGA-WC-AA9A-01 | Lymphocyte Depleted (Immune C4) |
| TCGA-EM-A3FQ-01 | Inflammatory (Immune C3) |
| TCGA-D7-6520-01 | IFN-gamma Dominant (Immune C2) |
| TCGA-CJ-5683-01 | Inflammatory (Immune C3) |
| TCGA-30-1855-01 | IFN-gamma Dominant (Immune C2) |
| TCGA-CA-6716-01 | Wound Healing (Immune C1) |
| TCGA-DX-A48L-01 | IFN-gamma Dominant (Immune C2) |
| TCGA-XF-A9T3-01 | IFN-gamma Dominant (Immune C2) |
| TCGA-AA-3548-01 | Wound Healing (Immune C1) |
| TCGA-ZJ-AAXF-01 | IFN-gamma Dominant (Immune C2) |
| TCGA-VF-A8A9-01 | IFN-gamma Dominant (Immune C2) |
| TCGA-B6-A0IP-01 | Inflammatory (Immune C3) |
| TCGA-HU-8604-01 | IFN-gamma Dominant (Immune C2) |
| TCGA-N5-A4RJ-01 | Wound Healing (Immune C1) |
| TCGA-LL-A441-01 | IFN-gamma Dominant (Immune C2) |
| TCGA-77-A5GF-01 | IFN-gamma Dominant (Immune C2) |
| TCGA-A8-A0A2-01 | Inflammatory (Immune C3) |
| TCGA-EJ-5507-01 | Inflammatory (Immune C3) |
| TCGA-C4-A0F1-01 | IFN-gamma Dominant (Immune C2) |
| TCGA-DD-AAE4-01 | IFN-gamma Dominant (Immune C2) |
| TCGA-24-2035-01 | Wound Healing (Immune C1) |
| TCGA-3A-A9IC-01 | Wound Healing (Immune C1) |
| TCGA-21-1080-01 | IFN-gamma Dominant (Immune C2) |
| TCGA-93-7347-01 | Inflammatory (Immune C3) |
| TCGA-EJ-5506-01 | Inflammatory (Immune C3) |
| TCGA-FG-8191-01 | Immunologically Quiet (Immune C5) |
| TCGA-CW-5585-01 | Inflammatory (Immune C3) |
| TCGA-E1-A7YW-01 | Immunologically Quiet (Immune C5) |
| TCGA-62-A471-01 | IFN-gamma Dominant (Immune C2) |
| TCGA-97-8177-01 | IFN-gamma Dominant (Immune C2) |
| TCGA-B6-A1KC-01 | Lymphocyte Depleted (Immune C4) |
| TCGA-78-7160-01 | Inflammatory (Immune C3) |
| TCGA-A2-A1FV-01 | Inflammatory (Immune C3) |
| TCGA-B8-4619-01 | Inflammatory (Immune C3) |
| TCGA-VP-A878-01 | Inflammatory (Immune C3) |
| TCGA-LG-A9QC-01 | Lymphocyte Depleted (Immune C4) |
| TCGA-KU-A6H7-01 | IFN-gamma Dominant (Immune C2) |
| TCGA-43-7658-01 | IFN-gamma Dominant (Immune C2) |
| TCGA-DB-5278-01 | Immunologically Quiet (Immune C5) |
| TCGA-AP-A1E1-01 | IFN-gamma Dominant (Immune C2) |
| TCGA-AA-A01Z-01 | Wound Healing (Immune C1) |
| TCGA-56-8305-01 | Wound Healing (Immune C1) |
| TCGA-B3-3925-01 | Inflammatory (Immune C3) |
| TCGA-GV-A3QK-01 | Wound Healing (Immune C1) |
| TCGA-E1-A7YS-01 | Immunologically Quiet (Immune C5) |
| TCGA-EM-A3FJ-01 | Inflammatory (Immune C3) |
| TCGA-97-7938-01 | Inflammatory (Immune C3) |
| TCGA-ZN-A9VP-01 | TGF-beta Dominant (Immune C6) |
| TCGA-SP-A6QF-01 | Lymphocyte Depleted (Immune C4) |
| TCGA-5P-A9KA-01 | Inflammatory (Immune C3) |
| TCGA-DD-AAD2-01 | Inflammatory (Immune C3) |
| TCGA-DD-A39W-01 | Inflammatory (Immune C3) |
| TCGA-BR-8384-01 | Inflammatory (Immune C3) |
| TCGA-D8-A1JT-01 | Lymphocyte Depleted (Immune C4) |
| TCGA-G6-A8L7-01 | Wound Healing (Immune C1) |
| TCGA-A2-A04P-01 | IFN-gamma Dominant (Immune C2) |
| TCGA-66-2755-01 | IFN-gamma Dominant (Immune C2) |
| TCGA-MA-AA3X-01 | IFN-gamma Dominant (Immune C2) |
| TCGA-61-2113-01 | IFN-gamma Dominant (Immune C2) |
| TCGA-DX-AB2O-01 | IFN-gamma Dominant (Immune C2) |
| TCGA-DX-A7EQ-01 | Inflammatory (Immune C3) |
| TCGA-02-2486-01 | Lymphocyte Depleted (Immune C4) |
| TCGA-AX-A0J1-01 | Wound Healing (Immune C1) |
| TCGA-BH-A0GY-01 | IFN-gamma Dominant (Immune C2) |
| TCGA-IN-8663-01 | IFN-gamma Dominant (Immune C2) |
| TCGA-E2-A56Z-01 | IFN-gamma Dominant (Immune C2) |
| TCGA-2A-AAYF-01 | Inflammatory (Immune C3) |
| TCGA-G9-6333-01 | Inflammatory (Immune C3) |
| TCGA-HT-7695-01 | Immunologically Quiet (Immune C5) |
| TCGA-A7-A13E-01 | Wound Healing (Immune C1) |
| TCGA-DU-7012-01 | Inflammatory (Immune C3) |
| TCGA-RX-A8JQ-01 | Inflammatory (Immune C3) |
| TCGA-BB-A6UM-01 | IFN-gamma Dominant (Immune C2) |
| TCGA-EB-A3Y7-01 | Inflammatory (Immune C3) |
| TCGA-PC-A5DN-01 | Inflammatory (Immune C3) |
| TCGA-CG-4444-01 | IFN-gamma Dominant (Immune C2) |
| TCGA-ZN-A9VS-01 | Lymphocyte Depleted (Immune C4) |
| TCGA-SL-A6J9-01 | IFN-gamma Dominant (Immune C2) |
| TCGA-CW-5590-01 | Inflammatory (Immune C3) |
| TCGA-AG-A002-01 | Wound Healing (Immune C1) |
| TCGA-HT-7879-01 | Immunologically Quiet (Immune C5) |
| TCGA-BK-A139-01 | IFN-gamma Dominant (Immune C2) |
| TCGA-FD-A6TG-01 | Wound Healing (Immune C1) |
| TCGA-O2-A52N-01 | Wound Healing (Immune C1) |
| TCGA-E2-A1LS-01 | Inflammatory (Immune C3) |
| TCGA-DU-A7TC-01 | Immunologically Quiet (Immune C5) |
| TCGA-DD-A39X-01 | Inflammatory (Immune C3) |
| TCGA-CH-5789-01 | Inflammatory (Immune C3) |
| TCGA-B6-A0X7-01 | IFN-gamma Dominant (Immune C2) |
| TCGA-02-2483-01 | Lymphocyte Depleted (Immune C4) |
| TCGA-69-8253-01 | Inflammatory (Immune C3) |
| TCGA-DX-A6YQ-01 | IFN-gamma Dominant (Immune C2) |
| TCGA-CV-7411-01 | IFN-gamma Dominant (Immune C2) |
| TCGA-B8-4622-01 | Inflammatory (Immune C3) |
| TCGA-DX-A1L4-01 | TGF-beta Dominant (Immune C6) |
| TCGA-A2-A0CX-01 | IFN-gamma Dominant (Immune C2) |
| TCGA-A8-A07S-01 | Lymphocyte Depleted (Immune C4) |
| TCGA-DJ-A13P-01 | Inflammatory (Immune C3) |
| TCGA-AT-A5NU-01 | Inflammatory (Immune C3) |
| TCGA-FY-A3W9-01 | Inflammatory (Immune C3) |
| TCGA-BH-A1FM-01 | Lymphocyte Depleted (Immune C4) |
| TCGA-EJ-A46D-01 | Inflammatory (Immune C3) |
| TCGA-JY-A93E-01 | Inflammatory (Immune C3) |
| TCGA-YL-A8SI-01 | Inflammatory (Immune C3) |
| TCGA-D5-6531-01 | IFN-gamma Dominant (Immune C2) |
| TCGA-VN-A88K-01 | Wound Healing (Immune C1) |
| TCGA-VS-A8EH-01 | Lymphocyte Depleted (Immune C4) |
| TCGA-AG-4015-01 | Wound Healing (Immune C1) |
| TCGA-GU-A766-01 | IFN-gamma Dominant (Immune C2) |
| TCGA-PR-A5PG-01 | Lymphocyte Depleted (Immune C4) |
| TCGA-2J-AABA-01 | Inflammatory (Immune C3) |
| TCGA-AZ-6598-01 | Wound Healing (Immune C1) |
| TCGA-78-7159-01 | Wound Healing (Immune C1) |
| TCGA-K1-A3PO-01 | Inflammatory (Immune C3) |
| TCGA-ET-A40S-01 | Inflammatory (Immune C3) |
| TCGA-DM-A1D9-01 | Wound Healing (Immune C1) |
| TCGA-W2-A7HE-01 | Inflammatory (Immune C3) |
| TCGA-27-2521-01 | Lymphocyte Depleted (Immune C4) |
| TCGA-A3-3351-01 | Inflammatory (Immune C3) |
| TCGA-HT-A74H-01 | Lymphocyte Depleted (Immune C4) |
| TCGA-HD-8224-01 | Inflammatory (Immune C3) |
| TCGA-DM-A0XF-01 | Wound Healing (Immune C1) |
| TCGA-A1-A0SM-01 | Wound Healing (Immune C1) |
| TCGA-XF-A9SH-01 | Inflammatory (Immune C3) |
| TCGA-KK-A8I8-01 | Wound Healing (Immune C1) |
| TCGA-DX-A23T-01 | TGF-beta Dominant (Immune C6) |
| TCGA-HT-7680-01 | Immunologically Quiet (Immune C5) |
| TCGA-DB-A4X9-01 | Immunologically Quiet (Immune C5) |
| TCGA-V1-A8MF-01 | Inflammatory (Immune C3) |
| TCGA-EL-A3CN-01 | IFN-gamma Dominant (Immune C2) |
| TCGA-SR-A6N0-01 | Inflammatory (Immune C3) |
| TCGA-CN-4730-01 | Wound Healing (Immune C1) |
| TCGA-OR-A5JB-01 | IFN-gamma Dominant (Immune C2) |
| TCGA-ET-A3DW-01 | Inflammatory (Immune C3) |
| TCGA-2G-AAHL-01 | IFN-gamma Dominant (Immune C2) |
| TCGA-AX-A062-01 | Wound Healing (Immune C1) |
| TCGA-43-2578-01 | Wound Healing (Immune C1) |
| TCGA-33-6738-01 | Wound Healing (Immune C1) |
| TCGA-AQ-A04H-01 | Wound Healing (Immune C1) |
| TCGA-HZ-A49I-01 | Wound Healing (Immune C1) |
| TCGA-BT-A20Q-01 | Wound Healing (Immune C1) |
| TCGA-B6-A0RS-01 | Wound Healing (Immune C1) |
| TCGA-PJ-A5Z8-01 | Inflammatory (Immune C3) |
| TCGA-ZG-A9KY-01 | Inflammatory (Immune C3) |
| TCGA-CQ-6224-01 | IFN-gamma Dominant (Immune C2) |
| TCGA-HZ-8001-01 | Wound Healing (Immune C1) |
| TCGA-97-7937-01 | Wound Healing (Immune C1) |
| TCGA-CJ-4901-01 | Inflammatory (Immune C3) |
| TCGA-BQ-5891-01 | Lymphocyte Depleted (Immune C4) |
| TCGA-EL-A3GO-01 | Inflammatory (Immune C3) |
| TCGA-AZ-6603-01 | Wound Healing (Immune C1) |
| TCGA-LL-A6FP-01 | Lymphocyte Depleted (Immune C4) |
| TCGA-A2-A0T5-01 | Wound Healing (Immune C1) |
| TCGA-VQ-A8E7-01 | Wound Healing (Immune C1) |
| TCGA-RD-A8N6-01 | Wound Healing (Immune C1) |
| TCGA-63-A5MP-01 | IFN-gamma Dominant (Immune C2) |
| TCGA-DD-AAE0-01 | Wound Healing (Immune C1) |
| TCGA-A6-A5ZU-01 | Inflammatory (Immune C3) |
| TCGA-BR-6454-01 | IFN-gamma Dominant (Immune C2) |
| TCGA-EB-A6R0-01 | IFN-gamma Dominant (Immune C2) |
| TCGA-CC-A5UE-01 | Lymphocyte Depleted (Immune C4) |
| TCGA-DF-A2KS-01 | IFN-gamma Dominant (Immune C2) |
| TCGA-VN-A88O-01 | Lymphocyte Depleted (Immune C4) |
| TCGA-E2-A573-01 | IFN-gamma Dominant (Immune C2) |
| TCGA-TQ-A7RI-01 | Immunologically Quiet (Immune C5) |
| TCGA-DR-A0ZM-01 | IFN-gamma Dominant (Immune C2) |
| TCGA-NH-A5IV-01 | Wound Healing (Immune C1) |
| TCGA-A7-A4SF-01 | Wound Healing (Immune C1) |
| TCGA-EJ-8469-01 | Inflammatory (Immune C3) |
| TCGA-33-4589-01 | IFN-gamma Dominant (Immune C2) |
| TCGA-XF-A9T4-01 | IFN-gamma Dominant (Immune C2) |
| TCGA-MA-AA43-01 | Wound Healing (Immune C1) |
| TCGA-2G-AAH0-01 | IFN-gamma Dominant (Immune C2) |
| TCGA-B6-A402-01 | Wound Healing (Immune C1) |
| TCGA-J2-A4AD-01 | Wound Healing (Immune C1) |
| TCGA-A2-A25B-01 | IFN-gamma Dominant (Immune C2) |
| TCGA-39-5037-01 | Wound Healing (Immune C1) |
| TCGA-97-A4LX-01 | Inflammatory (Immune C3) |
| TCGA-EM-A4FV-01 | Inflammatory (Immune C3) |
| TCGA-A2-A3XW-01 | IFN-gamma Dominant (Immune C2) |
| TCGA-BC-A110-01 | Inflammatory (Immune C3) |
| TCGA-GD-A6C6-01 | IFN-gamma Dominant (Immune C2) |
| TCGA-KL-8328-01 | Inflammatory (Immune C3) |
| TCGA-2A-AAYU-01 | Lymphocyte Depleted (Immune C4) |
| TCGA-TM-A7CA-01 | Immunologically Quiet (Immune C5) |
| TCGA-CU-A0YR-01 | Wound Healing (Immune C1) |
| TCGA-CA-6715-01 | Wound Healing (Immune C1) |
| TCGA-C5-A7UC-01 | IFN-gamma Dominant (Immune C2) |
| TCGA-A1-A0SI-01 | IFN-gamma Dominant (Immune C2) |
| TCGA-YU-A90Q-01 | IFN-gamma Dominant (Immune C2) |
| TCGA-XE-AAOB-01 | IFN-gamma Dominant (Immune C2) |
| TCGA-62-A46P-01 | Inflammatory (Immune C3) |
| TCGA-QH-A86X-01 | Immunologically Quiet (Immune C5) |
| TCGA-64-1678-01 | Wound Healing (Immune C1) |
| TCGA-B8-A54E-01 | Lymphocyte Depleted (Immune C4) |
| TCGA-HT-8013-01 | Immunologically Quiet (Immune C5) |
| TCGA-C5-A7XC-01 | IFN-gamma Dominant (Immune C2) |
| TCGA-XS-A8TJ-01 | IFN-gamma Dominant (Immune C2) |
| TCGA-CL-5917-01 | Wound Healing (Immune C1) |
| TCGA-DU-A76R-01 | Immunologically Quiet (Immune C5) |
| TCGA-DH-5144-01 | Immunologically Quiet (Immune C5) |
| TCGA-06-1804-01 | Lymphocyte Depleted (Immune C4) |
| TCGA-AQ-A04L-01 | Wound Healing (Immune C1) |
| TCGA-DV-A4VX-01 | Lymphocyte Depleted (Immune C4) |
| TCGA-KM-8442-01 | Inflammatory (Immune C3) |
| TCGA-K1-A42X-01 | Inflammatory (Immune C3) |
| TCGA-BB-7861-01 | IFN-gamma Dominant (Immune C2) |
| TCGA-BJ-A45J-01 | Inflammatory (Immune C3) |
| TCGA-GC-A3RC-01 | IFN-gamma Dominant (Immune C2) |
| TCGA-LG-A9QD-01 | Lymphocyte Depleted (Immune C4) |
| TCGA-2H-A9GK-01 | Wound Healing (Immune C1) |
| TCGA-25-2399-01 | IFN-gamma Dominant (Immune C2) |
| TCGA-CC-A7IK-01 | Lymphocyte Depleted (Immune C4) |
| TCGA-B5-A0JT-01 | Wound Healing (Immune C1) |
| TCGA-P4-A5EB-01 | Lymphocyte Depleted (Immune C4) |
| TCGA-DX-A3LY-01 | Lymphocyte Depleted (Immune C4) |
| TCGA-CV-7097-01 | IFN-gamma Dominant (Immune C2) |
| TCGA-34-2600-01 | Wound Healing (Immune C1) |
| TCGA-E2-A14N-01 | IFN-gamma Dominant (Immune C2) |
| TCGA-B9-5156-01 | Lymphocyte Depleted (Immune C4) |
| TCGA-85-7844-01 | Wound Healing (Immune C1) |
| TCGA-EA-A3HS-01 | IFN-gamma Dominant (Immune C2) |
| TCGA-X6-A8C2-01 | Lymphocyte Depleted (Immune C4) |
| TCGA-A8-A06Z-01 | Lymphocyte Depleted (Immune C4) |
| TCGA-85-A4JC-01 | Wound Healing (Immune C1) |
| TCGA-D7-6822-01 | IFN-gamma Dominant (Immune C2) |
| TCGA-D5-6932-01 | Wound Healing (Immune C1) |
| TCGA-RD-A8N2-01 | Inflammatory (Immune C3) |
| TCGA-2G-AAHG-01 | IFN-gamma Dominant (Immune C2) |
| TCGA-LK-A4O7-01 | Wound Healing (Immune C1) |
| TCGA-UZ-A9PU-01 | Inflammatory (Immune C3) |
| TCGA-CD-5799-01 | Wound Healing (Immune C1) |
| TCGA-AJ-A5DW-01 | Wound Healing (Immune C1) |
| TCGA-KS-A4IC-01 | Inflammatory (Immune C3) |
| TCGA-55-7728-01 | Inflammatory (Immune C3) |
| TCGA-33-4586-01 | Wound Healing (Immune C1) |
| TCGA-A1-A0SB-01 | Wound Healing (Immune C1) |
| TCGA-EM-A1YD-01 | Inflammatory (Immune C3) |
| TCGA-LN-A49S-01 | IFN-gamma Dominant (Immune C2) |
| TCGA-BH-A0GZ-01 | Wound Healing (Immune C1) |
| TCGA-BP-5196-01 | Inflammatory (Immune C3) |
| TCGA-49-4510-01 | Inflammatory (Immune C3) |
| TCGA-09-0366-01 | Lymphocyte Depleted (Immune C4) |
| TCGA-2Z-A9JI-01 | IFN-gamma Dominant (Immune C2) |
| TCGA-59-2348-01 | IFN-gamma Dominant (Immune C2) |
| TCGA-OR-A5JY-01 | Lymphocyte Depleted (Immune C4) |
| TCGA-2G-AAHC-01 | IFN-gamma Dominant (Immune C2) |
| TCGA-23-1027-01 | IFN-gamma Dominant (Immune C2) |
| TCGA-VS-A8QH-01 | Wound Healing (Immune C1) |
| TCGA-A3-3328-01 | Lymphocyte Depleted (Immune C4) |
| TCGA-BR-6852-01 | IFN-gamma Dominant (Immune C2) |
| TCGA-CV-7089-01 | IFN-gamma Dominant (Immune C2) |
| TCGA-A8-A06Y-01 | Lymphocyte Depleted (Immune C4) |
| TCGA-24-2019-01 | Wound Healing (Immune C1) |
| TCGA-13-0893-01 | IFN-gamma Dominant (Immune C2) |
| TCGA-BS-A0T9-01 | Wound Healing (Immune C1) |
| TCGA-A6-6137-01 | Wound Healing (Immune C1) |
| TCGA-B6-A409-01 | IFN-gamma Dominant (Immune C2) |
| TCGA-B8-5552-01 | Inflammatory (Immune C3) |
| TCGA-UZ-A9PQ-01 | Inflammatory (Immune C3) |
| TCGA-LP-A5U3-01 | IFN-gamma Dominant (Immune C2) |
| TCGA-55-A493-01 | Wound Healing (Immune C1) |
| TCGA-AK-3443-01 | Immunologically Quiet (Immune C5) |
| TCGA-MP-A4TE-01 | Wound Healing (Immune C1) |
| TCGA-55-6979-01 | IFN-gamma Dominant (Immune C2) |
| TCGA-18-4721-01 | IFN-gamma Dominant (Immune C2) |
| TCGA-WN-A9G9-01 | Inflammatory (Immune C3) |
| TCGA-IN-A6RJ-01 | Lymphocyte Depleted (Immune C4) |
| TCGA-41-5651-01 | Lymphocyte Depleted (Immune C4) |
| TCGA-RD-A8N9-01 | Wound Healing (Immune C1) |
| TCGA-CC-5264-01 | Wound Healing (Immune C1) |
| TCGA-E1-A7Z2-01 | Immunologically Quiet (Immune C5) |
| TCGA-DU-A5TP-01 | Lymphocyte Depleted (Immune C4) |
| TCGA-LN-A4A6-01 | IFN-gamma Dominant (Immune C2) |
| TCGA-YL-A8SJ-01 | Inflammatory (Immune C3) |
| TCGA-DS-A1O9-01 | IFN-gamma Dominant (Immune C2) |
| TCGA-HS-A5NA-01 | Lymphocyte Depleted (Immune C4) |
| TCGA-DM-A28M-01 | Wound Healing (Immune C1) |
| TCGA-06-0210-01 | Lymphocyte Depleted (Immune C4) |
| TCGA-EY-A1GO-01 | Wound Healing (Immune C1) |
| TCGA-A6-2681-01 | Wound Healing (Immune C1) |
| TCGA-44-2659-01 | Inflammatory (Immune C3) |
| TCGA-D6-A6ES-01 | Lymphocyte Depleted (Immune C4) |
| TCGA-DD-AAC9-01 | Inflammatory (Immune C3) |
| TCGA-YL-A8SF-01 | Inflammatory (Immune C3) |
| TCGA-AC-A2QI-01 | Inflammatory (Immune C3) |
| TCGA-55-7227-01 | Inflammatory (Immune C3) |
| TCGA-KK-A8IJ-01 | Inflammatory (Immune C3) |
| TCGA-GM-A3XL-01 | IFN-gamma Dominant (Immune C2) |
| TCGA-B5-A5OD-01 | Wound Healing (Immune C1) |
| TCGA-FG-8187-01 | Immunologically Quiet (Immune C5) |
| TCGA-56-7730-01 | Wound Healing (Immune C1) |
| TCGA-FX-A76Y-01 | IFN-gamma Dominant (Immune C2) |
| TCGA-61-1741-01 | IFN-gamma Dominant (Immune C2) |
| TCGA-BT-A20U-01 | Wound Healing (Immune C1) |
| TCGA-2G-AAG3-01 | Wound Healing (Immune C1) |
| TCGA-LN-A49W-01 | IFN-gamma Dominant (Immune C2) |
| TCGA-EA-A3HT-01 | Wound Healing (Immune C1) |
| TCGA-BT-A42C-01 | Wound Healing (Immune C1) |
| TCGA-2Z-A9JM-01 | Inflammatory (Immune C3) |
| TCGA-HZ-A4BH-01 | IFN-gamma Dominant (Immune C2) |
| TCGA-A8-A06N-01 | Lymphocyte Depleted (Immune C4) |
| TCGA-V5-A7RC-01 | IFN-gamma Dominant (Immune C2) |
| TCGA-CL-5918-01 | Wound Healing (Immune C1) |
| TCGA-AA-3955-01 | Wound Healing (Immune C1) |
| TCGA-B5-A3FH-01 | Wound Healing (Immune C1) |
| TCGA-VR-AA4G-01 | IFN-gamma Dominant (Immune C2) |
| TCGA-61-2012-01 | IFN-gamma Dominant (Immune C2) |
| TCGA-A3-3359-01 | Inflammatory (Immune C3) |
| TCGA-55-1592-01 | TGF-beta Dominant (Immune C6) |
| TCGA-L5-A8NR-01 | IFN-gamma Dominant (Immune C2) |
| TCGA-AA-A017-01 | Wound Healing (Immune C1) |
| TCGA-MP-A4TF-01 | Wound Healing (Immune C1) |
| TCGA-IR-A3LC-01 | IFN-gamma Dominant (Immune C2) |
| TCGA-5R-AAAM-01 | Inflammatory (Immune C3) |
| TCGA-E1-5304-01 | Lymphocyte Depleted (Immune C4) |
| TCGA-BC-A3KG-01 | Lymphocyte Depleted (Immune C4) |
| TCGA-BH-A8G0-01 | Inflammatory (Immune C3) |
| TCGA-63-A5MT-01 | IFN-gamma Dominant (Immune C2) |
| TCGA-AO-A03R-01 | Wound Healing (Immune C1) |
| TCGA-QH-A6CZ-01 | Immunologically Quiet (Immune C5) |
| TCGA-WZ-A7V5-01 | IFN-gamma Dominant (Immune C2) |
| TCGA-XF-AAML-01 | IFN-gamma Dominant (Immune C2) |
| TCGA-AX-A3GI-01 | IFN-gamma Dominant (Immune C2) |
| TCGA-75-5146-01 | IFN-gamma Dominant (Immune C2) |
| TCGA-EX-A69M-01 | IFN-gamma Dominant (Immune C2) |
| TCGA-Z4-AAPF-01 | Wound Healing (Immune C1) |
| TCGA-CN-4738-01 | IFN-gamma Dominant (Immune C2) |
| TCGA-EL-A3ZQ-01 | Inflammatory (Immune C3) |
| TCGA-CZ-5451-01 | Inflammatory (Immune C3) |
| TCGA-A2-A0T6-01 | Inflammatory (Immune C3) |
| TCGA-C9-A47Z-01 | IFN-gamma Dominant (Immune C2) |
| TCGA-67-4679-01 | Inflammatory (Immune C3) |
| TCGA-DX-A8BO-01 | IFN-gamma Dominant (Immune C2) |
| TCGA-CS-6665-01 | Immunologically Quiet (Immune C5) |
| TCGA-G9-7519-01 | Inflammatory (Immune C3) |
| TCGA-VQ-A8PP-01 | Wound Healing (Immune C1) |
| TCGA-CH-5748-01 | Inflammatory (Immune C3) |
| TCGA-19-5960-01 | Lymphocyte Depleted (Immune C4) |
| TCGA-YL-A8SQ-01 | Inflammatory (Immune C3) |
| TCGA-66-2737-01 | Wound Healing (Immune C1) |
| TCGA-BH-A1EW-01 | IFN-gamma Dominant (Immune C2) |
| TCGA-AA-A00J-01 | Wound Healing (Immune C1) |
| TCGA-EY-A1GX-01 | Lymphocyte Depleted (Immune C4) |
| TCGA-JY-A6F8-01 | Wound Healing (Immune C1) |
| TCGA-L5-A8NE-01 | Wound Healing (Immune C1) |
| TCGA-CG-4436-01 | IFN-gamma Dominant (Immune C2) |
| TCGA-A3-3323-01 | Inflammatory (Immune C3) |
| TCGA-AA-A01T-01 | Wound Healing (Immune C1) |
| TCGA-DH-5140-01 | Lymphocyte Depleted (Immune C4) |
| TCGA-FU-A770-01 | IFN-gamma Dominant (Immune C2) |
| TCGA-LL-A5YL-01 | Wound Healing (Immune C1) |
| TCGA-TM-A7CF-01 | Immunologically Quiet (Immune C5) |
| TCGA-3B-A9I0-01 | IFN-gamma Dominant (Immune C2) |
| TCGA-FD-A6TK-01 | IFN-gamma Dominant (Immune C2) |
| TCGA-BP-4776-01 | Inflammatory (Immune C3) |
| TCGA-C8-A26X-01 | IFN-gamma Dominant (Immune C2) |
| TCGA-BP-4972-01 | Inflammatory (Immune C3) |
| TCGA-XP-A8T8-01 | IFN-gamma Dominant (Immune C2) |
| TCGA-25-2391-01 | IFN-gamma Dominant (Immune C2) |
| TCGA-22-5492-01 | IFN-gamma Dominant (Immune C2) |
| TCGA-CM-6674-01 | IFN-gamma Dominant (Immune C2) |
| TCGA-IB-A5SQ-01 | TGF-beta Dominant (Immune C6) |
| TCGA-G3-A7M5-01 | Lymphocyte Depleted (Immune C4) |
| TCGA-B0-4813-01 | Inflammatory (Immune C3) |
| TCGA-IM-A3ED-01 | Inflammatory (Immune C3) |
| TCGA-E2-A1B4-01 | Inflammatory (Immune C3) |
| TCGA-V5-AASV-01 | IFN-gamma Dominant (Immune C2) |
| TCGA-D1-A16B-01 | Wound Healing (Immune C1) |
| TCGA-K4-A3WV-01 | Wound Healing (Immune C1) |
| TCGA-SX-A7SP-01 | Lymphocyte Depleted (Immune C4) |
| TCGA-NC-A5HD-01 | IFN-gamma Dominant (Immune C2) |
| TCGA-DH-A7US-01 | Immunologically Quiet (Immune C5) |
| TCGA-CJ-4641-01 | Inflammatory (Immune C3) |
| TCGA-AN-A0FD-01 | IFN-gamma Dominant (Immune C2) |
| TCGA-AN-A0AK-01 | Wound Healing (Immune C1) |
| TCGA-85-8048-01 | IFN-gamma Dominant (Immune C2) |
| TCGA-HT-A61B-01 | Lymphocyte Depleted (Immune C4) |
| TCGA-D1-A3DA-01 | Wound Healing (Immune C1) |
| TCGA-D8-A1JS-01 | Lymphocyte Depleted (Immune C4) |
| TCGA-BP-5184-01 | Inflammatory (Immune C3) |
| TCGA-B8-5158-01 | Inflammatory (Immune C3) |
| TCGA-QQ-A5VB-01 | IFN-gamma Dominant (Immune C2) |
| TCGA-CS-6670-01 | Immunologically Quiet (Immune C5) |
| TCGA-MB-A8JK-01 | Wound Healing (Immune C1) |
| TCGA-AO-A1KS-01 | Lymphocyte Depleted (Immune C4) |
| TCGA-WB-A81V-01 | Inflammatory (Immune C3) |
| TCGA-AR-A24U-01 | IFN-gamma Dominant (Immune C2) |
| TCGA-B0-4810-01 | Inflammatory (Immune C3) |
| TCGA-AG-3728-01 | Inflammatory (Immune C3) |
| TCGA-LL-A73Z-01 | IFN-gamma Dominant (Immune C2) |
| TCGA-E2-A15E-01 | Wound Healing (Immune C1) |
| TCGA-QR-A703-01 | Inflammatory (Immune C3) |
| TCGA-DK-A3X2-01 | IFN-gamma Dominant (Immune C2) |
| TCGA-96-7544-01 | Wound Healing (Immune C1) |
| TCGA-DD-AAEA-01 | Lymphocyte Depleted (Immune C4) |
| TCGA-F7-A50G-01 | IFN-gamma Dominant (Immune C2) |
| TCGA-L5-A8NI-01 | Wound Healing (Immune C1) |
| TCGA-BP-5195-01 | Inflammatory (Immune C3) |
| TCGA-2A-A8W1-01 | Lymphocyte Depleted (Immune C4) |
| TCGA-CV-6961-01 | Wound Healing (Immune C1) |
| TCGA-DJ-A2Q0-01 | Inflammatory (Immune C3) |
| TCGA-44-7662-01 | IFN-gamma Dominant (Immune C2) |
[truncated: 59,470 more chars]
