# Supplementary material for: Administration of statins is correlated with favourable prognosis in lung cancer patients receiving immune checkpoint inhibitors
Source: Front Immunol. 2025 Oct 6;16:1638677. doi: 10.3389/fimmu.2025.1638677 (PMC12535986; doi:10.3389/fimmu.2025.1638677)
Supplement: Supplementary Figure 1 — Comparison of therapy response between statin users and non-statin users in the first evaluation. [file DataSheet1.zip › Supplementary Table2.docx]

Supplementary Table 2. Statin target genes identified from online databases.

| Gene | | | | | |
| --- | --- | --- | --- | --- | --- |
| HMGCR | DPP4 | AHR | HDAC2 | TNF | CASP3 |
| CYP3A4 | NOS3 | SLCO1B1 | IL1B | ABCC2 | ITGB1 |
| MAPK1 | MAPK3 | NFE2L2 | PARP1 | AKT1 | APOB |
| ABCB1 | BCL2 | CASP9 | CCL2 | CRP | ABCB11 |
| ICAM1 | RAC1 | RELA | VCAM1 | ABCA1 | CASP8 |
| MAPK14 | MMP9 | NOS2 | PPARA | DGAT1 | EDNRA |
| GCK | MTTP | PTGS2 | ABCC3 | ACACB | APP |
| BAX | CAT | KCNH2 | MB | MMP2 | PRKCQ |
| SIRT1 | SLC10A2 | TGFB1 | ABCG2 | ACE | ADAM17 |
| CASP1 | CDC42 | CYBA | CYP17A1 | CYP19A1 | EGFR |
| F2 | HNF4A | IKBKB | ITGAL | MCL1 | MDM2 |
| MSTN | MTOR | PDPK1 | PIK3CA | PLAU | PPARG |
| PRKCA | SELE | SERPINE1 | SLC29A1 | SLC6A4 | SOD1 |
| HDAC6 | HDAC1 | PTGER4 | NR3C1 | FDFT1 | ESR2 |
| HSD11B1 | PTGER1 | PTGFR | PTGER3 | AKR1C3 | EGLN1 |
| NR1H4 | LTB4R | PDE5A | GRB2 | PTGIR | PDE4A |
| PDE4B | PDE4D | MMP1 | RARG | RARB | THRA |
| THRB | RARA | ELANE | TYRO3 | EDNRB | MMP13 |
| MMP12 | PPARD | FKBP1A | PTPN1 | RXRA | DUSP3 |
| SLC6A1 | PIK3CB | LTA4H | SRD5A2 | HRH1 | SSTR1 |
| CCR1 | CASP7 | NR3C2 | NR1H3 | MAPK8 | ITGA2B |
| PYGL | ITGA4 | PTPN22 | CMA1 | IMPDH2 | CDK4 |
| PLA2G2A | CNR1 | ITGAV | BACE2 | BACE1 | ITGB3 |
| S1PR3 | PTPRC | NR2E3 | GALR3 | ECE1 | HTR1E |
| GPR35 | AXL | GRIA2 | TMPRSS15 | PTGS1 | GCGR |
| CACNA1H | CRHR1 | HDAC8 | ESR1 | MMEL1 | SCN5A |
| ADRA1D | HCRTR2 | PTK2 | TRPV1 | CYP1A2 | CCR3 |
| CTSB | PTGDR2 | P2RY12 | WEE2 | AGTR1 | PTAFR |
| TACR1 | PDGFRB | GRM2 | STS | AKR1B1 | ACACA |
| AR | ADA | TNK2 | HTR1A | SCD | MLNR |
| PDE2A | ALK | TUBB2B | MC1R | ITGB5 | HSD11B2 |
| NPY1R | ADRA2B | ADRA1B | ALOX5 | TACR2 | S1PR1 |
| CHRFAM7A | PRKCE | HCAR2 | CHRNA7 | FNTA | AGTR2 |
| CYP11B2 | MTNR1B | BMP1 | HTR3A | PGR | ADAM10 |
| PIM2 | ACHE | GHSR | METAP2 | ADORA1 | ADORA2A |
| SIGMAR1 | SLC5A2 | CTSS | DRD4 | CNR2 | AVPR1A |
| JAK1 | CXCR2 | CXCR1 | MC3R | HRH2 | PLAT |
| MAP2K1 | JAK3 | CLK1 | ERBB2 | NTRK1 | EPHB4 |
| PDE7A | PTPN2 | CCR4 | CDK5 | CACNA1G | FGFR1 |
| PNOC | NPY5R | OPRD1 | SIRT2 | MC5R | DRD5 |
| CCNA1 | OPRM1 | PREP | NPBWR1 | TGM2 | SOAT1 |
| AKT3 | AVPR2 | CHRM2 | IGF1R | GRM1 | CA5A |
| ICMT | KCNA5 | LACTBL1 | PIK3CG | DHODH | DHFR2 |
| PDGFRA | RXRB | SYK | ADRB1 | ZAP70 | GRIN2B |
| MET | XDH | CTSL | MAPKAPK2 | MPL | EPHX2 |
| BTK | DRD2 | HTR4 | TGFBR1 | MAOB | MCHR1 |
| PTGER2 | CTSG | CA4 | PTGDR | TACR3 | F2R |
| HTR1F | CHRM5 | ADRA2A | HCRTR1 | PSEN2 | ADORA2B |
| CASR | SSTR4 | P2RY6 | CA12 | ROCK1 | TEK |
| CDK5R1 | CDK2 | FNTB | CA14 | PRSS2 | NOS1 |
| PRSS1 | MAOA | HTR1D | HTR1B | HTR2A | HCK |
| MAPK11 | TYK2 | SLC9A1 | ITK | CSF1R | CTSD |
| DRD1 | F7 | ADORA3 | P2RY1 | KDR | APOBEC3G |
| KIF11 | JAK2 | CA9 | OXTR | BDKRB2 | CCKBR |
| CCKAR | XIAP | MC4R | MIF | PLK1 | MAPK9 |
| PGGT1B | PLA2G1B | FOLH1 | RPS6KB1 | PLG | ALOX15 |
| F10 | CTDSP1 | SRC | MAP3K8 | S1PR5 | AVPR1B |
| PRCP | KCNC3 | PLIN5 | CXCR3 | GSK3B | GSK3A |
| FAAH | HRH3 | CES1 | PLD2 | MTNR1A | APOBEC3A |
| GPR119 | HDAC3 | LIPE | CYSLTR1 | FLT4 | PIM3 |
| CALCRL | CHRM4 | MAP3K10 | BRS3 | PNP | SSTR5 |
| REN | SLC5A1 | PTGES | CACNA1B | SSTR3 | CYP2C19 |
| MMP3 | P2RX7 | DRD3 | HTR5A | DYRK1A | HTR7 |
| SLC6A3 | ALOX5AP | CHRM1 | CHRM3 | CCNE2 | SLC6A2 |
| CHEK2 | P2RY2 | PAK4 | ATP4A | HDAC4 | SRD5A1 |
| DPP8 | S1PR2 | CASP6 | CCR5 | CCR8 | HTR2C |
| TAAR1 | GRM5 | HTR6 | ANPEP | PRKCG | CHRNA4 |
| LYN | KISS1R | CES2 | BCL2A1 | TOP1 | CXCR4 |
| PDE3A | CDK1 | HSP90AB1 | CLK4 | FLT3 | ADRB3 |
| CA2 | MLYCD | SLC6A9 | CA1 | PDE10A | TBXA2R |
| RORA | GPBAR1 | CA7 | CYP2D6 | NPY2R | NR2F2 |
| BDKRB1 | PTPN7 | SSTR2 | CYP11B1 | FDPS | TSPO |
| LCK | CDK9 | CSNK2A1 | ADRA2C | AKT2 | CYP2S1 |
| F3 | GRIN1 | HSD17B1 | APH1B | TYMS | ADK |
| HSP90AA1 | CDC7 | MGLL | TUBA1A | PLIN1 | PRKCD |
| FFAR1 | TPSAB1 | RPS6KA3 | ROCK2 | SCN9A | PSENEN |
| PNMT | CYP2C9 | CA13 | DPP9 | HRH4 | FLT1 |
| IRAK4 | APH1A | NCSTN | MMP8 | L3MBTL1 | NTSR1 |
| BCHE | GRM4 | TERT | CTSK | CDK7 | DPP7 |
| RXRG | PRKCB | CDC25B | PIM1 | CA5B | PLA2G7 |
| DNMT1 | TPSB2 | AURKA | CCR2 | CAPN1 | PIK3CD |
| GNRHR | CETP | CA6 | BCL2L1 | MMP7 | ABL1 |
| ALPL | PKIA | S1PR4 | SMO | PSEN1 | TLR9 |
| FURIN | BRAF | UTS2R | FYN | HSD17B2 | HSD17B3 |
