# Supplementary material for: Administration of statins is correlated with favourable prognosis in lung cancer patients receiving immune checkpoint inhibitors
Source: Front Immunol. 2025 Oct 6;16:1638677. doi: 10.3389/fimmu.2025.1638677 (PMC12535986; doi:10.3389/fimmu.2025.1638677)
Supplement: Supplementary Figure 1 — Comparison of therapy response between statin users and non-statin users in the first evaluation. [file DataSheet1.zip › Supplementary Table3.docx]

Supplementary Table 3. Univariate survival cox regression in the TCGA cohort.

| Gene | HR | z | p-value | lower | upper |
| --- | --- | --- | --- | --- | --- |
| CCL14 | 0.680142995 | -2.037577343 | 0.04159222 | 0.469436532 | 0.985424999 |
| ARRDC5 | 0.82898084 | -1.982446279 | 0.047429314 | 0.688672509 | 0.997875222 |
| ZDHHC11 | 0.96577597 | -2.539696539 | 0.011094869 | 0.940167063 | 0.99208243 |
| PNMA8C | 0.474241633 | -2.074787838 | 0.038006202 | 0.234385277 | 0.95955313 |
| SCN1A | 0.808776854 | -2.220619699 | 0.02637673 | 0.670619947 | 0.975395978 |
| AL441992.2 | 0.041772926 | -2.032802078 | 0.042072523 | 0.001955262 | 0.892451795 |
| ATXN7 | 0.971167456 | -1.972213694 | 0.048585217 | 0.943337631 | 0.999818301 |
| IRX5 | 0.991022787 | -2.461874371 | 0.013821307 | 0.983933443 | 0.998163211 |
| CERKL | 0.933719374 | -2.117152212 | 0.034246932 | 0.876282281 | 0.994921259 |
| IL2 | 0.550765371 | -2.243628129 | 0.024856338 | 0.327101925 | 0.927363828 |
| ZNF77 | 0.972953031 | -2.120117452 | 0.033996142 | 0.948600369 | 0.997930879 |
| GATA1 | 0.707695886 | -2.024156536 | 0.042954048 | 0.506355101 | 0.989095332 |
| AMIGO1 | 0.97510828 | -2.053138097 | 0.040059186 | 0.951924456 | 0.998856738 |
| ZNF556 | 0.928794365 | -1.977673543 | 0.047965547 | 0.863229783 | 0.999338751 |
| ZNF709 | 0.209369889 | -2.514900791 | 0.011906588 | 0.061897797 | 0.708195654 |
| NDUFA7 | 0.776793085 | -2.232662002 | 0.025571242 | 0.622312951 | 0.969620663 |
| ZNF540 | 0.832693294 | -2.965463819 | 0.003022269 | 0.737787449 | 0.939807424 |
| OGT | 0.996131639 | -2.254575712 | 0.024159973 | 0.992780928 | 0.999493658 |
| CBX7 | 0.978185754 | -2.299963259 | 0.021450302 | 0.959972216 | 0.996744857 |
| CD1E | 0.98154833 | -2.080116907 | 0.037514811 | 0.96447412 | 0.998924807 |
| FNIP2 | 0.991462013 | -2.10984917 | 0.03487135 | 0.983595897 | 0.999391037 |
| ZNF441 | 0.929814211 | -2.464436347 | 0.013722894 | 0.877529429 | 0.985214215 |
| ANKRD44 | 0.95522053 | -3.0499172 | 0.002289045 | 0.92750804 | 0.983761025 |
| CCDC190 | 0.991585226 | -1.992092193 | 0.046360943 | 0.983375272 | 0.999863723 |
| COX14 | 0.99673487 | -2.088607829 | 0.03674304 | 0.993680546 | 0.999798582 |
| COL4A3 | 0.976948119 | -2.613081044 | 0.008973002 | 0.960007295 | 0.994187891 |
| BORCS8 | 0.958770277 | -2.274784029 | 0.022918889 | 0.924612472 | 0.994189967 |
| KLRB1 | 0.980674597 | -2.177777832 | 0.029422581 | 0.963601571 | 0.998050122 |
| DHRS4L2 | 0.982500741 | -2.003621375 | 0.045110634 | 0.965679104 | 0.999615403 |
| CCDC177 | 0.916825076 | -2.173035539 | 0.029777643 | 0.847755998 | 0.991521408 |
| SLC38A4 | 0.978940353 | -2.139704439 | 0.03237866 | 0.960039189 | 0.998213641 |
| CD302 | 0.96511804 | -2.668469501 | 0.00761977 | 0.94027506 | 0.990617395 |
| DNASE2B | 0.928251188 | -2.163939132 | 0.030469021 | 0.867718606 | 0.993006561 |
| MTUS1 | 0.994201461 | -2.51170914 | 0.012014807 | 0.989700048 | 0.998723348 |
| ZNF248 | 0.962494305 | -2.035691064 | 0.041781387 | 0.927713593 | 0.998578973 |
| HLA-DMB | 0.995068658 | -2.712610021 | 0.006675562 | 0.991520716 | 0.998629296 |
| NECAB3 | 0.995236835 | -2.054278176 | 0.039948776 | 0.990713503 | 0.999780819 |
| FGFBP2 | 0.999465259 | -2.034045431 | 0.041947015 | 0.998950264 | 0.999980519 |
| STK33 | 0.954612487 | -2.378286478 | 0.017393307 | 0.918760847 | 0.99186312 |
| MUSK | 0.645792888 | -2.481991604 | 0.013065035 | 0.457223833 | 0.912131922 |
| GALNT11 | 0.991965384 | -1.962742749 | 0.049676073 | 0.984006562 | 0.999988579 |
| ATP13A4 | 0.993489339 | -2.559414369 | 0.010484868 | 0.988532239 | 0.998471297 |
| LIFR | 0.991784032 | -2.338614905 | 0.019355373 | 0.984950347 | 0.998665129 |
| NRXN3 | 0.957102773 | -2.113300032 | 0.034575096 | 0.918964532 | 0.996823802 |
| DNAJC28 | 0.905955887 | -2.039356174 | 0.041414493 | 0.823917877 | 0.996162471 |
| BTBD9 | 0.988018345 | -2.682461297 | 0.007308261 | 0.979354709 | 0.996758622 |
| GLB1L3 | 0.985166545 | -2.596868978 | 0.00940778 | 0.974117005 | 0.996341421 |
| ROM1 | 0.961168158 | -2.322471331 | 0.02020757 | 0.929573088 | 0.993837106 |
| GPR31 | 0.501833433 | -2.069023359 | 0.038543895 | 0.261157711 | 0.964309242 |
| ITIH4 | 0.559479687 | -1.980726855 | 0.047621913 | 0.314928877 | 0.99393083 |
| CYP17A1 | 0.483881261 | -2.143092769 | 0.03210565 | 0.249124763 | 0.939854681 |
| MOK | 0.953102422 | -2.498945798 | 0.012456336 | 0.917864134 | 0.989693565 |
| ZNF747 | 0.969943398 | -2.253100344 | 0.024252823 | 0.944532976 | 0.996037429 |
| SMAD9 | 0.972359224 | -2.237192269 | 0.025273776 | 0.948772224 | 0.996532609 |
| AC010547.4 | 0.00013174 | -2.029877367 | 0.042369006 | 2.36E-08 | 0.735113978 |
| BTK | 0.981095223 | -2.201557576 | 0.027696575 | 0.964565933 | 0.997907767 |
| ABHD14B | 0.995837517 | -2.028644055 | 0.042494558 | 0.991832412 | 0.999858794 |
| ZNF20 | 0.373998383 | -2.837813191 | 0.004542376 | 0.189612925 | 0.737685946 |
| SCART1 | 0.904912887 | -2.288080457 | 0.022132836 | 0.830684761 | 0.985773871 |
| ACOXL | 0.930471656 | -2.74424556 | 0.006065015 | 0.88379322 | 0.979615462 |
| AQP6 | 0.869315504 | -1.986503265 | 0.046977471 | 0.757124723 | 0.998130721 |
| ENPP5 | 0.991930726 | -2.32971685 | 0.019821121 | 0.985192592 | 0.998714945 |
| SEPTIN1 | 0.975082428 | -2.008132492 | 0.044629214 | 0.95136139 | 0.99939492 |
| AC012651.1 | 0.143005204 | -2.312217479 | 0.020765702 | 0.027503068 | 0.743571171 |
| WDR83 | 0.932713199 | -2.123725041 | 0.033693142 | 0.874639277 | 0.994643088 |
| ZNF44 | 0.934624903 | -3.312889069 | 0.000923376 | 0.897978384 | 0.972766967 |
| CFAP91 | 0.917092734 | -1.991322119 | 0.046445485 | 0.84220613 | 0.998638044 |
| VMAC | 0.972042484 | -2.004120942 | 0.045057107 | 0.945457095 | 0.99937543 |
| CCDC181 | 0.937459176 | -2.138820209 | 0.032450232 | 0.88358875 | 0.994613961 |
| RPS6KA5 | 0.898399779 | -2.236039707 | 0.02534917 | 0.817869868 | 0.986858905 |
| GGA2 | 0.994205304 | -2.031502694 | 0.042204027 | 0.988646494 | 0.999795369 |
| SETDB2 | 0.960665451 | -2.145413425 | 0.031919807 | 0.926084904 | 0.996537255 |
| LDHD | 0.99101166 | -2.322344079 | 0.020214416 | 0.983488759 | 0.998592104 |
| PZP | 0.927189491 | -2.018231412 | 0.043567166 | 0.861558685 | 0.997819845 |
| ZSCAN31 | 0.990949442 | -2.115243411 | 0.034409207 | 0.982636412 | 0.999332799 |
| NDNF | 0.998207863 | -2.086594109 | 0.03692484 | 0.996527413 | 0.999891148 |
| CARMIL3 | 0.902933725 | -2.931648013 | 0.003371687 | 0.843353104 | 0.966723557 |
| CES4A | 0.969651882 | -2.085683192 | 0.03700733 | 0.941972988 | 0.998144091 |
| CA5B | 0.932771927 | -2.260561246 | 0.02378644 | 0.878152677 | 0.99078838 |
| SENP8 | 0.884563215 | -2.022837531 | 0.043089901 | 0.785440905 | 0.996194718 |
| GMPR | 0.989194544 | -2.225728758 | 0.026032355 | 0.979776042 | 0.998703586 |
| HPGDS | 0.971885087 | -1.996476414 | 0.045882091 | 0.94505338 | 0.999478592 |
| CABLES1 | 0.987855291 | -2.168371986 | 0.030130395 | 0.977004804 | 0.998826283 |
| TREML1 | 0.882393539 | -2.385619456 | 0.017050384 | 0.796195778 | 0.977923245 |
| EPHX1 | 0.999749952 | -2.247986607 | 0.024577044 | 0.999531992 | 0.999967959 |
| HLA-DMA | 0.998724208 | -2.59868865 | 0.009358061 | 0.997763067 | 0.999686276 |
| SETD4 | 0.974605218 | -2.097298237 | 0.035967183 | 0.951456584 | 0.99831705 |
| NRTN | 0.981670961 | -1.960068715 | 0.049987759 | 0.963678829 | 0.999999012 |
| TSLP | 0.951926511 | -2.67750622 | 0.007417248 | 0.918207618 | 0.986883646 |
| DMRTC1 | 7.10E-05 | -2.629952713 | 0.008539675 | 5.75E-08 | 0.087724242 |
| TRIP6 | 0.998553034 | -2.016607211 | 0.04373652 | 0.997148717 | 0.999959328 |
| HLF | 0.984648761 | -2.703848954 | 0.006854144 | 0.973668502 | 0.995752847 |
| CYP2U1 | 0.955740094 | -2.526017529 | 0.011536373 | 0.922752543 | 0.98990692 |
| FDX2 | 0.174549476 | -2.355239358 | 0.018510778 | 0.040838001 | 0.746058052 |
| ADAMTS8 | 0.973613362 | -2.059963884 | 0.039401994 | 0.949154304 | 0.998702713 |
| ACSBG1 | 0.683930662 | -2.284703473 | 0.022330221 | 0.493713424 | 0.947434539 |
| UPRT | 0.975890665 | -2.02649589 | 0.042713993 | 0.953125959 | 0.999199089 |
| GGTLC1 | 0.996679762 | -2.447108986 | 0.014400729 | 0.994028434 | 0.999338161 |
| JAML | 0.981638681 | -2.103806213 | 0.035395355 | 0.964836246 | 0.998733727 |
| SORCS2 | 0.989725578 | -2.308838453 | 0.020952547 | 0.981086546 | 0.998440681 |
| KLHDC8B | 0.991516369 | -3.630229915 | 0.000283169 | 0.986966008 | 0.99608771 |
| HHATL | 0.808159463 | -2.032456576 | 0.042107456 | 0.658102395 | 0.992431759 |
| BEST4 | 0.922322868 | -2.357615898 | 0.018392713 | 0.862360855 | 0.986454182 |
| DEXI | 0.919400361 | -2.493567941 | 0.012646638 | 0.860635099 | 0.98217819 |
| PAPLN | 0.982376438 | -2.300488917 | 0.021420536 | 0.96760681 | 0.99737151 |
| MPP7 | 0.992030615 | -2.345663109 | 0.018993266 | 0.985420369 | 0.998685204 |
| RNFT1 | 0.980271116 | -2.179574694 | 0.029289002 | 0.962862683 | 0.99799429 |
| ZNF589 | 0.972227793 | -3.167664028 | 0.00153669 | 0.955431621 | 0.989319236 |
| CFTR | 0.981703676 | -2.357125825 | 0.018417005 | 0.966745339 | 0.996893461 |
| SCN4A | 0.891268441 | -2.171237694 | 0.029913209 | 0.803306925 | 0.988861679 |
| SLC4A8 | 0.925842197 | -2.429217165 | 0.015131466 | 0.870037582 | 0.985226146 |
| INAFM2 | 0.985482777 | -2.919607111 | 0.003504729 | 0.975855624 | 0.995204906 |
| STIMATE-MUSTN1 | 0.295772216 | -2.441678557 | 0.014619157 | 0.111247091 | 0.786368464 |
| SLC47A1 | 0.982928106 | -2.618612346 | 0.008828821 | 0.970341215 | 0.995678269 |
| TRDMT1 | 0.861405201 | -2.299220048 | 0.021492447 | 0.758534411 | 0.978227104 |
| TMEM150A | 0.99382647 | -2.178150103 | 0.029394863 | 0.988303929 | 0.999379871 |
| RFTN2 | 0.927453618 | -1.966502104 | 0.049240639 | 0.860385623 | 0.999749636 |
| BCDIN3D | 0.929980409 | -1.980615204 | 0.047634443 | 0.865518416 | 0.999243396 |
| SCN4B | 0.975894977 | -2.008214072 | 0.044620548 | 0.952929499 | 0.999413921 |
| CX3CR1 | 0.969695439 | -2.268854302 | 0.023277187 | 0.944257012 | 0.99581918 |
| ABCA13 | 0.988995582 | -2.000639754 | 0.045431226 | 0.978332337 | 0.99977505 |
| DMD | 0.967493762 | -2.02902415 | 0.042455831 | 0.937097605 | 0.998875864 |
| RNF180 | 0.95884205 | -2.151856575 | 0.031408652 | 0.922830319 | 0.996259071 |
| TSPOAP1 | 0.974929345 | -2.101589098 | 0.035589286 | 0.952114941 | 0.998290424 |
| NUDT14 | 0.995407597 | -1.970674356 | 0.048761136 | 0.990861071 | 0.999974984 |
| SIX1 | 0.989967643 | -2.809987864 | 0.004954337 | 0.983029727 | 0.996954524 |
| AMPH | 0.954831393 | -2.002739808 | 0.045205224 | 0.912603474 | 0.999013279 |
| CPED1 | 0.96179965 | -2.620280503 | 0.008785747 | 0.934182966 | 0.990232749 |
| GPD1L | 0.993625749 | -3.183774338 | 0.001453683 | 0.989721917 | 0.997544979 |
| XCR1 | 0.913034377 | -2.361094956 | 0.018221064 | 0.846617375 | 0.984661783 |
| SMARCE1 | 0.894209779 | -2.093144312 | 0.036336274 | 0.805320195 | 0.992910811 |
| CXCL17 | 0.999785846 | -2.401080219 | 0.016346753 | 0.999611069 | 0.999960653 |
| CLHC1 | 0.932751828 | -2.001853911 | 0.045300446 | 0.871294312 | 0.998544304 |
| UQCR10 | 0.996990698 | -2.06337995 | 0.039076543 | 0.994140608 | 0.999848959 |
| DRAM1 | 0.998633671 | -2.031031192 | 0.042251831 | 0.997316921 | 0.99995216 |
| RAB44 | 0.583866861 | -3.136392093 | 0.001710404 | 0.417139109 | 0.817234594 |
| ERC2 | 0.885694348 | -2.101331723 | 0.035611857 | 0.790886642 | 0.991867149 |
| SLC26A5 | 0.882127659 | -2.303012492 | 0.02127814 | 0.792823147 | 0.981491534 |
| CD74 | 0.999924836 | -2.375199517 | 0.017539467 | 0.999862817 | 0.999986859 |
| RNF175 | 0.906344523 | -2.709411702 | 0.006740264 | 0.844111242 | 0.973166041 |
| KCNJ11 | 0.983307695 | -1.985193487 | 0.047122949 | 0.967100893 | 0.999786093 |
| ZNF254 | 0.980105445 | -2.291163598 | 0.021953953 | 0.963401162 | 0.997099361 |
| ANKRD45 | 0.914407963 | -2.169817018 | 0.030020711 | 0.84340919 | 0.991383462 |
| ZNF396 | 0.918353297 | -1.983991026 | 0.04725684 | 0.844243154 | 0.998969046 |
| STIMATE | 0.548435784 | -2.073849341 | 0.038093305 | 0.310869035 | 0.967551527 |
| KCNJ15 | 0.992559814 | -2.085972316 | 0.036981131 | 0.985619519 | 0.999548979 |
| STING1 | 0.997375233 | -2.07989046 | 0.037535581 | 0.994908116 | 0.999848468 |
| TESPA1 | 0.932865615 | -2.336674374 | 0.019456122 | 0.880042876 | 0.988858929 |
| IL33 | 0.99559408 | -2.331188296 | 0.019743433 | 0.991904795 | 0.999297087 |
| GLIPR1L2 | 0.831921767 | -2.003924816 | 0.045078115 | 0.694893349 | 0.995971292 |
| RORA | 0.969221224 | -2.103664104 | 0.035407758 | 0.941398006 | 0.997866762 |
| TTLL2 | 0.718981451 | -2.13691004 | 0.032605309 | 0.531251063 | 0.973050904 |
| ZNF563 | 0.917199977 | -2.333610954 | 0.019616102 | 0.852978638 | 0.986256584 |
| NIBAN1 | 0.995430295 | -1.984723411 | 0.047175253 | 0.99093809 | 0.999942864 |
| C1QL2 | 0.988970531 | -2.308347607 | 0.020979811 | 0.979701215 | 0.998327547 |
| GRIA1 | 0.881302207 | -2.322214484 | 0.020221389 | 0.792154482 | 0.980482465 |
| TCTA | 0.991121922 | -3.232528764 | 0.001226998 | 0.985777347 | 0.996495473 |
| ZNF502 | 0.960143723 | -2.914000178 | 0.003568297 | 0.93423377 | 0.986772261 |
| ZNF831 | 0.845963387 | -2.198607653 | 0.027905832 | 0.728766867 | 0.982006844 |
| FAM214A | 0.98275527 | -2.0114482 | 0.044278137 | 0.966238032 | 0.99955486 |
| FYB2 | 0.943968527 | -2.205336743 | 0.027430473 | 0.896811844 | 0.993604829 |
| NKIRAS1 | 0.94813932 | -2.294930221 | 0.021737124 | 0.905982976 | 0.992257244 |
| GDF15 | 0.999328055 | -2.01430535 | 0.043977485 | 0.998674671 | 0.999981867 |
| ZNF626 | 0.972349022 | -1.988377774 | 0.046769925 | 0.94584154 | 0.999599384 |
| DAPK2 | 0.969938193 | -1.979877096 | 0.047717341 | 0.941068955 | 0.999693055 |
| CRYGN | 0.914158033 | -2.242726483 | 0.024914458 | 0.845195135 | 0.988747893 |
| CLECL1 | 0.898767034 | -2.756557238 | 0.005841339 | 0.833085118 | 0.96962743 |
| TMA7 | 0.989321646 | -2.007078569 | 0.044741298 | 0.97900401 | 0.999748018 |
| SNAI3 | 0.938925637 | -2.369668054 | 0.017804062 | 0.891239297 | 0.989163465 |
| POU2AF1 | 0.992720695 | -2.125308962 | 0.033560841 | 0.986054679 | 0.999431774 |
| HLA-DPB1 | 0.999066132 | -2.335281383 | 0.019528726 | 0.998283025 | 0.999849854 |
| ACSM5 | 0.860189576 | -1.994726778 | 0.046072687 | 0.741870669 | 0.99737884 |
| SPTLC2 | 0.993833758 | -2.032158501 | 0.042137613 | 0.987922601 | 0.999780284 |
| C6 | 0.962928101 | -2.132264253 | 0.032985129 | 0.930065298 | 0.996952075 |
| CD200R1 | 0.879787029 | -3.468922458 | 0.00052255 | 0.818371521 | 0.945811525 |
| NPRL2 | 0.977408818 | -2.679238304 | 0.007378986 | 0.961206408 | 0.993884342 |
| BDH2 | 0.977362463 | -2.029420186 | 0.042415511 | 0.955986264 | 0.999216641 |
| ZNF10 | 0.960436161 | -1.966196456 | 0.049275922 | 0.922555661 | 0.99987205 |
| MS4A2 | 0.95536086 | -2.331482398 | 0.019727937 | 0.919380269 | 0.992749576 |
| CACNA2D2 | 0.995932328 | -2.049994024 | 0.040365014 | 0.992058769 | 0.999821011 |
| ZNF57 | 0.689726352 | -2.174949296 | 0.029633916 | 0.493514378 | 0.963948493 |
| PGM5 | 0.964253822 | -2.385637731 | 0.017049537 | 0.935844088 | 0.993526 |
| CSAD | 0.985101806 | -2.182082871 | 0.029103417 | 0.971909442 | 0.998473237 |
| FRS3 | 0.979422708 | -1.991985109 | 0.046372692 | 0.959589512 | 0.999665826 |
| KCNK16 | 0.73252251 | -2.046846828 | 0.040673126 | 0.543725769 | 0.986874742 |
| GPLD1 | 0.903548643 | -2.715456015 | 0.006618457 | 0.839765839 | 0.972175947 |
| C12orf42 | 0.765102116 | -1.991628898 | 0.04641179 | 0.587878466 | 0.995752154 |
| TTC23L | 0.74588939 | -2.989321076 | 0.002795981 | 0.615449969 | 0.903974345 |
| MAGEH1 | 0.996211806 | -2.061127404 | 0.039290887 | 0.992622854 | 0.999813734 |
| OPHN1 | 0.971251844 | -2.117133734 | 0.034248499 | 0.945375098 | 0.997836887 |
| PLPPR1 | 0.979507828 | -2.80780878 | 0.004987984 | 0.965452852 | 0.993767415 |
| STK32A | 0.9819232 | -2.607016603 | 0.009133493 | 0.968548505 | 0.995482587 |
| EFHC2 | 0.969995667 | -2.246065216 | 0.024699831 | 0.94454975 | 0.996127091 |
| TMEM163 | 0.993528825 | -2.038533276 | 0.04149663 | 0.987346553 | 0.999749808 |
| TLR2 | 0.994450335 | -2.061118027 | 0.039291781 | 0.989201604 | 0.999726916 |
| IVD | 0.992533267 | -2.476151715 | 0.013280713 | 0.986662628 | 0.998438837 |
| PDIK1L | 0.984159573 | -2.21380417 | 0.026842253 | 0.970344987 | 0.998170834 |
| CLEC2D | 0.963551499 | -2.436274738 | 0.014839409 | 0.935195567 | 0.992767207 |
| MADCAM1 | 0.823474187 | -2.131192252 | 0.033073307 | 0.688774386 | 0.984516485 |
| TMEM243 | 0.991604419 | -2.106290041 | 0.035179166 | 0.98385541 | 0.99941446 |
| CRY2 | 0.99111363 | -2.121507568 | 0.033879112 | 0.982974111 | 0.999320548 |
| SLC38A6 | 0.966719174 | -2.161734499 | 0.030638647 | 0.937503056 | 0.996845775 |
| HAPLN4 | 8.81E-25 | -1.981166585 | 0.047572595 | 1.41E-48 | 0.552793945 |
| NPC2 | 0.999678399 | -2.03529959 | 0.041820737 | 0.9993688 | 0.999988094 |
| C12orf76 | 0.967888493 | -2.607275397 | 0.009126592 | 0.944430093 | 0.991929569 |
| FUCA1 | 0.99890866 | -2.023931097 | 0.042977242 | 0.997852948 | 0.99996549 |
| BCL6 | 0.995592307 | -2.033043455 | 0.042048133 | 0.991361445 | 0.999841224 |
| AC055839.2 | 0.975589449 | -2.123565697 | 0.033706477 | 0.953588629 | 0.998097863 |
| C3orf62 | 0.942345935 | -2.290765587 | 0.021976975 | 0.895663582 | 0.991461391 |
| SH2B1 | 0.989110826 | -2.408966331 | 0.015997774 | 0.980338805 | 0.997961339 |
| RTL5 | 0.975649514 | -2.562267314 | 0.010399124 | 0.957424038 | 0.99422193 |
| HLA-DPA1 | 0.999442456 | -2.079810459 | 0.037542921 | 0.998917323 | 0.999967864 |
| ADHFE1 | 0.903355551 | -2.349835187 | 0.018781725 | 0.829929313 | 0.98327802 |
| PHKG2 | 0.974214475 | -2.814442087 | 0.004886198 | 0.956651332 | 0.992100059 |
| AZIN2 | 0.940971064 | -2.187562682 | 0.028701473 | 0.89104927 | 0.993689769 |
| ATAD3C | 0.98041954 | -2.778465178 | 0.005461637 | 0.966838302 | 0.994191556 |
| CXorf21 | 0.974715859 | -2.179670903 | 0.029281865 | 0.952526658 | 0.99742196 |
| PDZD9 | 0.547895778 | -2.143196744 | 0.032097304 | 0.316035555 | 0.949860795 |
| CYP4X1 | 0.996217558 | -2.227905663 | 0.025886806 | 0.992901847 | 0.999544342 |
| C5orf38 | 0.992818733 | -2.15265321 | 0.031345943 | 0.986325142 | 0.999355076 |
| AL669918.1 | 6.17E-14 | -2.211083402 | 0.027030062 | 1.20E-25 | 0.031603801 |
| PARP15 | 0.972285443 | -2.087205828 | 0.036869533 | 0.946960125 | 0.998288057 |
| NR3C2 | 0.97945293 | -2.220902021 | 0.026357598 | 0.961670949 | 0.997563711 |
| CYP4B1 | 0.998914527 | -2.322725423 | 0.020193908 | 0.997999499 | 0.999830394 |
| RSBN1L | 0.986201125 | -2.090248306 | 0.0365955 | 0.973435354 | 0.999134307 |
| HMGN3 | 0.998629419 | -2.774849712 | 0.005522722 | 0.997662467 | 0.999597309 |
| LRP2BP | 0.921667412 | -2.107311638 | 0.035090575 | 0.854329708 | 0.99431263 |
| LRRC3C | 0.573475678 | -1.999522939 | 0.045551802 | 0.332512207 | 0.989059488 |
| FITM1 | 0.735030275 | -2.364035873 | 0.018077062 | 0.569458633 | 0.948742322 |
| MYLIP | 0.993285219 | -3.402169096 | 0.000668533 | 0.989437373 | 0.997148029 |
| ZNF555 | 0.878280898 | -2.151992582 | 0.031397939 | 0.780362926 | 0.988485372 |
| RNF130 | 0.984358141 | -2.156312115 | 0.031059302 | 0.970352954 | 0.998565466 |
| AKR1A1 | 0.997680725 | -2.245584059 | 0.024730662 | 0.99566084 | 0.999704708 |
| RORB | 0.906359767 | -2.189235638 | 0.028579718 | 0.829990283 | 0.989756199 |
| TP53INP1 | 0.995453124 | -1.961357825 | 0.049837296 | 0.99093013 | 0.999996761 |
| CD5 | 0.984934532 | -2.412558115 | 0.015841012 | 0.972862587 | 0.997156275 |
| SLC6A18 | 0.475759051 | -2.197698745 | 0.027970581 | 0.245285899 | 0.922787146 |
| GGT6 | 0.993576239 | -2.099292191 | 0.035791151 | 0.98761607 | 0.999572377 |
| KHDRBS2 | 0.952151267 | -2.457098143 | 0.014006442 | 0.915630463 | 0.990128738 |
| CHADL | 0.959882419 | -2.107289824 | 0.035092465 | 0.924015497 | 0.997141563 |
| SLC34A2 | 0.999847828 | -2.78025429 | 0.005431635 | 0.999740568 | 0.999955101 |
| GRM6 | 0.535401829 | -1.96171309 | 0.049795897 | 0.286814838 | 0.999443126 |
| GCSAML | 0.848885164 | -1.967252265 | 0.049154135 | 0.721043536 | 0.999393221 |
| ANKRD29 | 0.986046492 | -2.270740068 | 0.023162718 | 0.97415933 | 0.998078706 |
| SPRYD4 | 0.950414206 | -2.379278835 | 0.017346549 | 0.911419633 | 0.99107714 |
| EPHX2 | 0.986625374 | -2.056685869 | 0.039716452 | 0.974046226 | 0.999366974 |
| AC068896.1 | 0.498061359 | -2.318364179 | 0.020429537 | 0.276288813 | 0.897847127 |
| ADCY9 | 0.982615416 | -2.593036612 | 0.009513264 | 0.969676001 | 0.995727497 |
| C1QTNF7 | 0.938571354 | -2.262029034 | 0.02369561 | 0.888405484 | 0.991569956 |
| ABCC8 | 0.801549868 | -2.299654802 | 0.021467785 | 0.663822366 | 0.967852582 |
| LRIG1 | 0.991391289 | -3.002305909 | 0.002679428 | 0.985811376 | 0.997002785 |
| LUZP2 | 0.840532847 | -3.122763755 | 0.001791615 | 0.7537067 | 0.937361267 |
| C11orf16 | 0.968745831 | -2.402030499 | 0.016304349 | 0.943968745 | 0.994173261 |
| CPXM2 | 0.997691483 | -1.963601304 | 0.049576346 | 0.995392556 | 0.999995719 |
| FGD3 | 0.978448905 | -2.031237901 | 0.042230867 | 0.958094415 | 0.99923582 |
| PRDM16 | 0.972508229 | -2.390816436 | 0.016810956 | 0.950535494 | 0.99498889 |
| SEC14L6 | 0.982740289 | -2.053640961 | 0.040010455 | 0.966545779 | 0.999206139 |
| LIPT1 | 0.941602391 | -2.533284004 | 0.011299936 | 0.89877145 | 0.986474439 |
| SLC5A11 | 0.928723368 | -2.009091455 | 0.044527436 | 0.864088066 | 0.998193503 |
| PRICKLE4 | 0.34207715 | -2.25024166 | 0.024433609 | 0.134383331 | 0.870768537 |
| FHAD1 | 0.934791948 | -1.978141736 | 0.047912719 | 0.874377624 | 0.999380545 |
| CLIC6 | 0.998488383 | -2.705737037 | 0.006815299 | 0.997394836 | 0.99958313 |
| TCEA3 | 0.997095605 | -2.003248035 | 0.045150672 | 0.99426213 | 0.999937156 |
| DNASE2 | 0.997412208 | -1.982475237 | 0.047426076 | 0.994860385 | 0.999970578 |
| CLEC9A | 0.802776467 | -2.067818228 | 0.038657119 | 0.651876708 | 0.988607274 |
| SLC16A11 | 0.971679963 | -2.157076568 | 0.030999699 | 0.946643842 | 0.997378221 |
| USP4 | 0.98734307 | -2.178318388 | 0.029382341 | 0.976091844 | 0.998723988 |
| FAM189A2 | 0.967588336 | -2.149493213 | 0.031595324 | 0.938951073 | 0.997099013 |
| SNX30 | 0.98756777 | -2.834863201 | 0.004584531 | 0.979062877 | 0.996146543 |
| POU6F1 | 0.952595766 | -2.089586665 | 0.036654945 | 0.910176561 | 0.996991938 |
| MS4A4E | 0.844896556 | -2.236477404 | 0.025320516 | 0.728881518 | 0.979377544 |
| KLRG1 | 0.900473456 | -2.510000701 | 0.012073092 | 0.829695982 | 0.977288626 |
| GAPT | 0.941481949 | -2.333642346 | 0.019614457 | 0.894988373 | 0.990390812 |
| C19orf18 | 0.936474809 | -2.152553525 | 0.031353784 | 0.882150024 | 0.994145036 |
| NAPSA | 0.999799713 | -2.18963096 | 0.028551012 | 0.999620469 | 0.99997899 |
| CCDC88C | 0.989040207 | -2.117792707 | 0.034192627 | 0.979004246 | 0.999179049 |
| DENND1C | 0.984927343 | -2.580288703 | 0.009871775 | 0.973630298 | 0.996355468 |
| ELAPOR1 | 0.996668945 | -2.699679978 | 0.00694062 | 0.994257557 | 0.999086181 |
| PXMP4 | 0.990542728 | -2.396630555 | 0.016546598 | 0.982875092 | 0.998270181 |
| ZNF506 | 0.962768867 | -2.401742925 | 0.016317171 | 0.933415586 | 0.993045225 |
| SGF29 | 0.981831099 | -2.42379338 | 0.015359342 | 0.967380769 | 0.996497282 |
| HLA-DRB1 | 0.999922592 | -2.1009944 | 0.035641458 | 0.999850385 | 0.999994804 |
| ZNF655 | 0.989318037 | -2.542668338 | 0.01100096 | 0.981162004 | 0.997541868 |
| TLR5 | 0.966373879 | -2.389692391 | 0.016862489 | 0.939640322 | 0.993868029 |
| HLA-DQA1 | 0.99875874 | -2.26312454 | 0.023628013 | 0.997684999 | 0.999833635 |
| TMEM50B | 0.994028275 | -2.300266447 | 0.021433129 | 0.988968157 | 0.999114284 |
| ZDHHC11B | 0.97356783 | -2.995213431 | 0.002742529 | 0.956650866 | 0.990783947 |
| NDUFA11 | 0.950788783 | -2.214189197 | 0.026815767 | 0.909252303 | 0.994222734 |
| IKZF4 | 0.968797757 | -2.066430434 | 0.038787855 | 0.94010323 | 0.998368119 |
| ZFP3 | 0.967238578 | -2.068338839 | 0.038608172 | 0.937184757 | 0.998256172 |
| TTC39B | 0.977458505 | -2.135852277 | 0.032691457 | 0.957220666 | 0.998124219 |
| PELI1 | 0.996966751 | -2.240245989 | 0.025074958 | 0.994320546 | 0.999619999 |
| SH3BP5 | 0.971715595 | -2.053914282 | 0.039983989 | 0.945471254 | 0.998688424 |
| PPP1R13B | 0.98515627 | -2.891287779 | 0.003836666 | 0.975219419 | 0.995194372 |
| RAP1GAP | 0.997806301 | -2.094932453 | 0.036176997 | 0.995758292 | 0.999858523 |
| CIITA | 0.981773459 | -2.170371204 | 0.029978736 | 0.965599523 | 0.998218311 |
| ESYT3 | 0.968489403 | -2.718164064 | 0.006564528 | 0.946386243 | 0.991108789 |
| CDKL2 | 0.9883453 | -2.056456213 | 0.039738563 | 0.977363902 | 0.999450082 |
| NUPR1 | 0.941850049 | -2.955789219 | 0.003118701 | 0.905168116 | 0.980018518 |
| SUSD3 | 0.986057076 | -2.377760447 | 0.017418137 | 0.974710351 | 0.997535889 |
| P2RY13 | 0.979879241 | -2.240496472 | 0.02505871 | 0.962610065 | 0.997458226 |
| PET100 | 0.894432157 | -2.10035528 | 0.0356976 | 0.80599707 | 0.992570462 |
| FMC1 | 0.76633645 | -2.949127568 | 0.003186724 | 0.642104477 | 0.914604361 |
| TTC28 | 0.96767066 | -2.384102678 | 0.017120825 | 0.941877131 | 0.994170551 |
| TFAP2E | 0.96799915 | -2.072377396 | 0.03823026 | 0.938676932 | 0.99823733 |
| ZC3H12D | 0.901195054 | -2.628110925 | 0.008586051 | 0.833919358 | 0.973898157 |
| ATG16L2 | 0.978151858 | -2.500464983 | 0.012403039 | 0.961360663 | 0.995236329 |
| SCUBE3 | 0.991290524 | -2.036700939 | 0.04168002 | 0.982980825 | 0.999670469 |
| SELENOK | 0.994979278 | -2.061962125 | 0.039211342 | 0.990230284 | 0.999751048 |
| CERS4 | 0.994695636 | -2.795041833 | 0.0051893 | 0.990992855 | 0.998412253 |
| PLEKHB1 | 0.99382922 | -2.228427136 | 0.025852045 | 0.988433328 | 0.999254568 |
| ENTPD3 | 0.994518436 | -2.005250842 | 0.044936237 | 0.989189706 | 0.999875871 |
| ZNF25 | 0.962930847 | -2.545954789 | 0.01089793 | 0.935332533 | 0.99134349 |
| GDAP1 | 0.939094419 | -2.27291732 | 0.023031163 | 0.889561801 | 0.991385115 |
| HLA-DOB | 0.977892547 | -2.41410207 | 0.015774043 | 0.960303899 | 0.995803343 |
| PPM1M | 0.983204423 | -2.974533927 | 0.00293434 | 0.972292043 | 0.994239278 |
| SLC9A9 | 0.987844119 | -2.532369363 | 0.011329458 | 0.978537432 | 0.99723932 |
| PRKCE | 0.962829194 | -2.01899936 | 0.043487286 | 0.928067403 | 0.998893027 |
| GSTA3 | 0.93681156 | -2.331828941 | 0.019709692 | 0.88679901 | 0.989644655 |
| HLA-DRA | 0.999955024 | -2.15164746 | 0.031425131 | 0.999914056 | 0.999995993 |
| HLA-DQB1 | 0.999159714 | -1.969019828 | 0.048950814 | 0.998323994 | 0.999996134 |
| CPEB3 | 0.916397955 | -2.158606701 | 0.030880692 | 0.846559287 | 0.991998109 |
| ECHDC2 | 0.988245742 | -2.162876804 | 0.030550656 | 0.977713596 | 0.998891343 |
| MICU3 | 0.895091215 | -3.047368243 | 0.002308547 | 0.833508378 | 0.96122403 |
| SLC25A42 | 0.967623143 | -2.790500361 | 0.005262664 | 0.945511341 | 0.990252053 |
| PXK | 0.972498911 | -2.206095844 | 0.02737729 | 0.94870119 | 0.996893586 |
| SULT1A3 | 0.139251638 | -2.183735579 | 0.028981684 | 0.023732148 | 0.817078094 |
| CPA3 | 0.997448698 | -1.976198346 | 0.048132317 | 0.994924783 | 0.999979015 |
| CCL17 | 0.995187668 | -1.965084332 | 0.049404478 | 0.990410944 | 0.99998743 |
| RPS27AP5 | 0.927570356 | -1.997204622 | 0.045802959 | 0.861593837 | 0.998599024 |
| EXPH5 | 0.97399312 | -2.698646814 | 0.006962202 | 0.955529939 | 0.992813054 |
| COL21A1 | 0.986378048 | -2.049553312 | 0.04040804 | 0.973525137 | 0.999400649 |
| ALK | 0.885408345 | -2.456866714 | 0.014015468 | 0.803484416 | 0.975685306 |
| JPH1 | 0.983969309 | -3.098299287 | 0.001946348 | 0.973961362 | 0.994080093 |
| CATSPERE | 0.764891676 | -2.850786149 | 0.004361129 | 0.636169596 | 0.919659286 |
| ZNF490 | 0.851868713 | -2.443394964 | 0.014549804 | 0.749068016 | 0.968777585 |
| SUOX | 0.98886409 | -1.99520113 | 0.046020948 | 0.978045601 | 0.999802246 |
| TPPP | 0.985873109 | -2.808089546 | 0.004983637 | 0.976131382 | 0.995712059 |
| TMEM168 | 0.984038512 | -2.265886888 | 0.02345831 | 0.970437667 | 0.997829975 |
| PTCHD4 | 0.93463348 | -2.2342269 | 0.025468147 | 0.880818843 | 0.991735985 |
| NRL | 0.799768075 | -2.146580304 | 0.031826709 | 0.652174928 | 0.9807629 |
| MOAP1 | 0.994019139 | -2.074248568 | 0.038056232 | 0.988400677 | 0.999669539 |
| PGPEP1 | 0.98539868 | -2.156857919 | 0.031016737 | 0.972315251 | 0.998658158 |
| CIRBP | 0.996623615 | -2.664410675 | 0.007712335 | 0.994147197 | 0.999106203 |
| NEIL1 | 0.973185554 | -2.30763398 | 0.021019503 | 0.950976444 | 0.995913335 |
| MAPK13 | 0.996576292 | -2.048217976 | 0.040538646 | 0.99331108 | 0.999852236 |
| FAM13B | 0.978633437 | -2.000344881 | 0.045463036 | 0.958141064 | 0.999564094 |
| C10orf143 | 0.860964036 | -2.107966016 | 0.035033929 | 0.749091342 | 0.989544306 |
| FAIM2 | 0.956667721 | -2.66250237 | 0.007756202 | 0.925973776 | 0.9883791 |
| CLEC18B | 0.782235333 | -2.012139129 | 0.044205273 | 0.615801352 | 0.993651791 |
| GAB3 | 0.955856807 | -2.222643481 | 0.02623985 | 0.91855024 | 0.994678566 |
| SCGB3A1 | 0.999912682 | -2.466176246 | 0.013656412 | 0.999843292 | 0.999982076 |
| AQP5 | 0.99923591 | -2.166575546 | 0.030267234 | 0.99854519 | 0.999927109 |
| PLAAT1 | 0.991378352 | -1.999291136 | 0.045576863 | 0.982998454 | 0.999829687 |
| ZNF512 | 0.985380076 | -2.195546783 | 0.028124399 | 0.972509539 | 0.998420946 |
| GLS2 | 0.474748396 | -2.431209354 | 0.015048517 | 0.260398592 | 0.865542467 |
| MCTP2 | 0.978730087 | -2.10556437 | 0.03524221 | 0.95933776 | 0.998514416 |
| CCDC66 | 0.935778039 | -2.196974275 | 0.028022284 | 0.881973589 | 0.992864809 |
| SUSD4 | 0.995545784 | -1.976356433 | 0.048114422 | 0.991148107 | 0.999962974 |
| ZFP2 | 0.878913847 | -2.297630746 | 0.021582814 | 0.787282203 | 0.981210484 |
| TRAF3IP3 | 0.952530883 | -2.339756215 | 0.019296331 | 0.914505893 | 0.992136947 |
| PDE6C | 0.653063025 | -2.180153482 | 0.029246086 | 0.44524517 | 0.957879711 |
| SLAMF1 | 0.955953527 | -2.326670388 | 0.019982815 | 0.920358267 | 0.992925449 |
| DAAM2 | 0.978657874 | -2.132164227 | 0.032993348 | 0.95944143 | 0.998259201 |
| ZNF493 | 0.9433298 | -2.239846965 | 0.02510086 | 0.896381855 | 0.992736641 |
| SPATA6L | 0.920612741 | -2.11135684 | 0.034741652 | 0.852569492 | 0.994086495 |
| SLC25A23 | 0.994940663 | -2.176719277 | 0.029501519 | 0.990407032 | 0.999495046 |
| MCOLN2 | 0.958426488 | -2.920656096 | 0.003492952 | 0.931501335 | 0.986129916 |
| FCRL6 | 0.941681084 | -2.190358683 | 0.028498234 | 0.892385775 | 0.993699461 |
| ITPKB | 0.995339898 | -1.966428064 | 0.049249184 | 0.990716724 | 0.999984646 |
| GDPD1 | 0.975112729 | -2.02834847 | 0.042524695 | 0.951653087 | 0.999150685 |
| STX10 | 0.99648642 | -2.103649166 | 0.035409062 | 0.993223938 | 0.999759619 |
| SFTA3 | 0.996733214 | -2.209126347 | 0.027165854 | 0.993843816 | 0.999631012 |
| INPP5J | 0.960014621 | -2.843054453 | 0.004468344 | 0.93338417 | 0.987404868 |
| NWD1 | 0.965521666 | -2.093811968 | 0.036276734 | 0.934325373 | 0.997759576 |
| MTURN | 0.991200934 | -1.963485749 | 0.049589759 | 0.982494865 | 0.999984148 |
| ATP8A2 | 0.93283723 | -2.4857265 | 0.012928728 | 0.8830762 | 0.985402275 |
| CCR2 | 0.970032615 | -2.416754667 | 0.015659568 | 0.946390097 | 0.994265766 |
| FAM184A | 0.961090742 | -2.750746838 | 0.005945958 | 0.934294233 | 0.988655802 |
| LRRC4 | 0.996357881 | -2.324333748 | 0.020107613 | 0.993297023 | 0.999428171 |
| GNG7 | 0.950917799 | -3.435409285 | 0.00059166 | 0.924002495 | 0.97861712 |
| GSTA4 | 0.998083623 | -2.275796322 | 0.022858203 | 0.996436143 | 0.999733828 |
| ZNF483 | 0.922704343 | -2.013069265 | 0.044107343 | 0.853192026 | 0.997880053 |
| SCN7A | 0.973409171 | -2.285205436 | 0.022300785 | 0.951166876 | 0.996171585 |
| ABAT | 0.974494347 | -2.821350636 | 0.00478219 | 0.957159762 | 0.992142869 |
| WFIKKN2 | 0.615344273 | -2.431604249 | 0.015032122 | 0.41604424 | 0.910116131 |
| BTLA | 0.920412457 | -2.046970719 | 0.04066096 | 0.850150676 | 0.996481112 |
| RMDN2 | 0.952971708 | -2.808325956 | 0.004979979 | 0.921466808 | 0.985553758 |
| GUCY1A1 | 0.984238903 | -1.968169321 | 0.049048559 | 0.96879038 | 0.999933771 |
| GCGR | 0.936659885 | -2.00682789 | 0.044767992 | 0.878673376 | 0.998473113 |
| ATP5MG | 0.987693935 | -2.011869929 | 0.04423365 | 0.97585101 | 0.999680587 |
| DNAH12 | 0.926031883 | -2.157957155 | 0.030931163 | 0.863602634 | 0.9929741 |
| PCP2 | 0.932558626 | -2.856308665 | 0.004285982 | 0.888931478 | 0.978326916 |
| NGF | 0.98883847 | -2.163704339 | 0.030487048 | 0.978835514 | 0.998943649 |
| ANKDD1B | 0.954898315 | -2.029674915 | 0.042389594 | 0.91327726 | 0.99841618 |
| THSD7B | 0.955185366 | -2.310333668 | 0.020869688 | 0.91874522 | 0.993070837 |
| ETV1 | 0.990349267 | -2.267810023 | 0.023340788 | 0.982083646 | 0.998684456 |
| PIGR | 0.999795397 | -2.417780153 | 0.015615508 | 0.999629566 | 0.999961254 |
| MGP | 0.998968613 | -2.333272741 | 0.019633835 | 0.998103063 | 0.999834913 |
| ICAM5 | 0.984491682 | -2.217017503 | 0.026621896 | 0.970981902 | 0.99818943 |
| AC008770.2 | 0.038450777 | -2.33574255 | 0.019504663 | 0.002497315 | 0.592020748 |
| CD40LG | 0.942938641 | -2.674529202 | 0.007483426 | 0.903200637 | 0.984424994 |
| ABCC4 | 0.990376678 | -2.100212633 | 0.035710141 | 0.981479541 | 0.999354467 |
| ZNF791 | 0.971617393 | -2.62280106 | 0.008721018 | 0.950934858 | 0.992749767 |
| ANKHD1-EIF4EBP3 | 0.557671507 | -2.091819443 | 0.03645467 | 0.322658906 | 0.96385844 |
| AC006254.1 | 0.638115317 | -2.266563011 | 0.023416934 | 0.432702812 | 0.941041162 |
| CCDC153 | 0.955946863 | -2.315094629 | 0.020607754 | 0.920171814 | 0.993112799 |
| ADGRF5 | 0.998674768 | -2.410532494 | 0.015929252 | 0.997598539 | 0.999752159 |
| CAPN3 | 0.914039679 | -2.078404156 | 0.03767215 | 0.83975877 | 0.994891109 |
| IRX6 | 0.988003241 | -2.438398501 | 0.014752501 | 0.978464764 | 0.997634702 |
| PRRT3 | 0.970287422 | -2.050244821 | 0.040340546 | 0.942708957 | 0.998672682 |
| RALGPS1 | 0.961316328 | -2.143712475 | 0.032055932 | 0.927259418 | 0.996624101 |
| FBXO9 | 0.985931009 | -2.351678136 | 0.018688939 | 0.974356807 | 0.997642699 |
| SUSD2 | 0.998933384 | -2.348932857 | 0.018827301 | 0.998044265 | 0.999823296 |
| ZNF682 | 0.953950867 | -2.866622404 | 0.004148777 | 0.923692784 | 0.985200137 |
| SEC14L3 | 0.845486792 | -2.130219441 | 0.033153501 | 0.724501955 | 0.986674929 |
| FYCO1 | 0.989384585 | -2.202012138 | 0.027664451 | 0.980030856 | 0.99882759 |
| PRKCD | 0.996169201 | -2.148483313 | 0.031675381 | 0.992687338 | 0.999663277 |
| FAM200A | 0.981042195 | -2.281869005 | 0.022497075 | 0.965045963 | 0.997303574 |
| LYRM9 | 0.93017539 | -1.975336523 | 0.048229971 | 0.865713771 | 0.999436864 |
| NFIX | 0.996793645 | -2.082253722 | 0.037319301 | 0.993784991 | 0.999811408 |
| MAGI3 | 0.989091742 | -2.102853998 | 0.035478534 | 0.979031869 | 0.999254984 |
| ANOS1 | 0.987683181 | -3.032051121 | 0.002428981 | 0.979802247 | 0.995627505 |
| DMTF1 | 0.986682587 | -2.101730149 | 0.035576921 | 0.974423322 | 0.999096086 |
| TMEM215 | 0.728697084 | -2.035829385 | 0.041767491 | 0.537299285 | 0.98827498 |
| AL365205.1 | 0.833712654 | -1.973042696 | 0.048490698 | 0.695915237 | 0.998795188 |
| CLEC18A | 0.258180149 | -2.087137733 | 0.036875686 | 0.072389985 | 0.920804019 |
| CRYM | 0.994886928 | -2.608158644 | 0.009103075 | 0.991061798 | 0.998726821 |
| AC144573.1 | 0.000338318 | -1.983220574 | 0.047342797 | 1.26E-07 | 0.910543104 |
| CISH | 0.992667945 | -2.467649262 | 0.013600351 | 0.986882687 | 0.998487118 |
| GPR174 | 0.946765711 | -2.238091888 | 0.025215064 | 0.902479584 | 0.99322503 |
| ITGA8 | 0.983574019 | -1.965078009 | 0.049405209 | 0.96745955 | 0.999956898 |
| NOTCH2NLR | 0.302663702 | -2.626877556 | 0.008617234 | 0.124078017 | 0.738288045 |
| ABCC6 | 0.984890498 | -2.053993668 | 0.039976305 | 0.970685603 | 0.999303267 |
| CENPC | 0.968280479 | -1.987549707 | 0.046861513 | 0.937986626 | 0.999552723 |
| CLNK | 0.818396202 | -2.260087892 | 0.023815796 | 0.687836213 | 0.973738124 |
| MERTK | 0.993451162 | -2.004748401 | 0.044989951 | 0.987090083 | 0.999853234 |
| CAV3 | 0.517396489 | -2.14416371 | 0.032019772 | 0.283290272 | 0.944964064 |
| IRX2 | 0.996450707 | -2.060059286 | 0.039392874 | 0.993085564 | 0.999827253 |
| NEUROD1 | 0.91860464 | -1.972551472 | 0.048546687 | 0.844291773 | 0.999458376 |
| GPRIN2 | 0.989239229 | -2.414396557 | 0.015761298 | 0.980589038 | 0.997965726 |
| LCA5 | 0.954986444 | -2.784403224 | 0.005362632 | 0.924521635 | 0.986455128 |
| KCNS3 | 0.996988714 | -2.163401737 | 0.030510294 | 0.994268427 | 0.999716444 |
| CLEC3B | 0.988719879 | -2.524042869 | 0.011601378 | 0.980048504 | 0.997467978 |
| AMPD1 | 0.902609774 | -2.147587141 | 0.031746568 | 0.822030227 | 0.991088135 |
| CACNB4 | 0.620695871 | -2.711549228 | 0.00669696 | 0.439711071 | 0.8761739 |
| CTF1 | 0.991465314 | -2.239954909 | 0.02509385 | 0.984057225 | 0.998929173 |
| SMARCD3 | 0.988087679 | -2.213619204 | 0.026854985 | 0.97765887 | 0.998627732 |
| AL139353.1 | 0.498814657 | -2.059846777 | 0.039413191 | 0.257350763 | 0.966836308 |
| SCML4 | 0.852971658 | -2.146001004 | 0.031872899 | 0.737660429 | 0.98630836 |
| HLA-DRB5 | 0.999851388 | -2.044590184 | 0.040895278 | 0.999708947 | 0.999993848 |
| ANKRD65 | 0.99472875 | -2.980729299 | 0.002875629 | 0.991277817 | 0.998191697 |
| SNX20 | 0.976059624 | -2.019176462 | 0.043468882 | 0.953369607 | 0.999289659 |
| ERO1B | 0.990852813 | -2.570238569 | 0.01016285 | 0.983933794 | 0.997820486 |
| ZNF394 | 0.955060021 | -2.508417419 | 0.012127331 | 0.921356132 | 0.989996823 |
| KMT2E | 0.989494468 | -2.280845522 | 0.02255759 | 0.980555122 | 0.998515311 |
