# Supplementary material for: Administration of statins is correlated with favourable prognosis in lung cancer patients receiving immune checkpoint inhibitors
Source: Front Immunol. 2025 Oct 6;16:1638677. doi: 10.3389/fimmu.2025.1638677 (PMC12535986; doi:10.3389/fimmu.2025.1638677)
Supplement: Supplementary Figure 1 — Comparison of therapy response between statin users and non-statin users in the first evaluation. [file DataSheet1.zip › Supplementary Table4.docx]

Supplementary Table 4. Baseline characteristics of the validation cohort.

| Clinical features | Non-statin  (n=29) | Statin  (n=13) | p-value |
| --- | --- | --- | --- |
| Age |  |  | 0.391 |
| ＜70 years | 16 (55.2) | 9 (69.2) |  |
| ≥70 years | 13 (44.8) | 4 (30.8) |  |
| Gender |  |  | 0.686 |
| Female | 6 (20.7) | 2 (15.4) |  |
| Male | 23 (79.3) | 11 (84.6) |  |
| Smoking |  |  | 0.555 |
| Never | 11 (37.9) | 3 (23.1) |  |
| Current/former | 18 (62.1) | 10 (76.9) |  |
| Stage |  |  | 0.899 |
| Ⅲ | 15 (51.7) | 7 (53.8) |  |
| Ⅳ | 14 (48.3) | 6 (46.2) |  |
| ECOG |  |  | 0.892 |
| 0-1 | 25 (86.2) | 11 (84.6) |  |
| ≥2 | 4 (13.8) | 2 (15.4) |  |
| Treatment |  |  | 0.766 |
| Monotherapy | 2 (6.9) | 2 (15.4) |  |
| Combination | 27 (93.1) | 11 (84.6) |  |
| Surgery History |  |  | 0.686 |
| No | 23 (79.3) | 11 (84.6) |  |
| Yes | 6 (20.7) | 2 (15.4) |  |
| CD8^+^ T cell |  |  | 0.032 |
| Richness | 6(20.7) | 7(53.8) |  |
| Deficiency | 23(79.3) | 6(46.2) |  |
| RORA expression |  |  | 0.036 |
| High | 8(27.6) | 8(61.5) |  |
| Low | 21(72.4) | 5(38.5) |  |

Abbreviations: ECOG, Eastern Cooperative Oncology Group.
