# Supplementary figures and images for: Administration of statins is correlated with favourable prognosis in lung cancer patients receiving immune checkpoint inhibitors
Source: Front Immunol. 2025 Oct 6;16:1638677. doi: 10.3389/fimmu.2025.1638677 (PMC12535986; doi:10.3389/fimmu.2025.1638677)

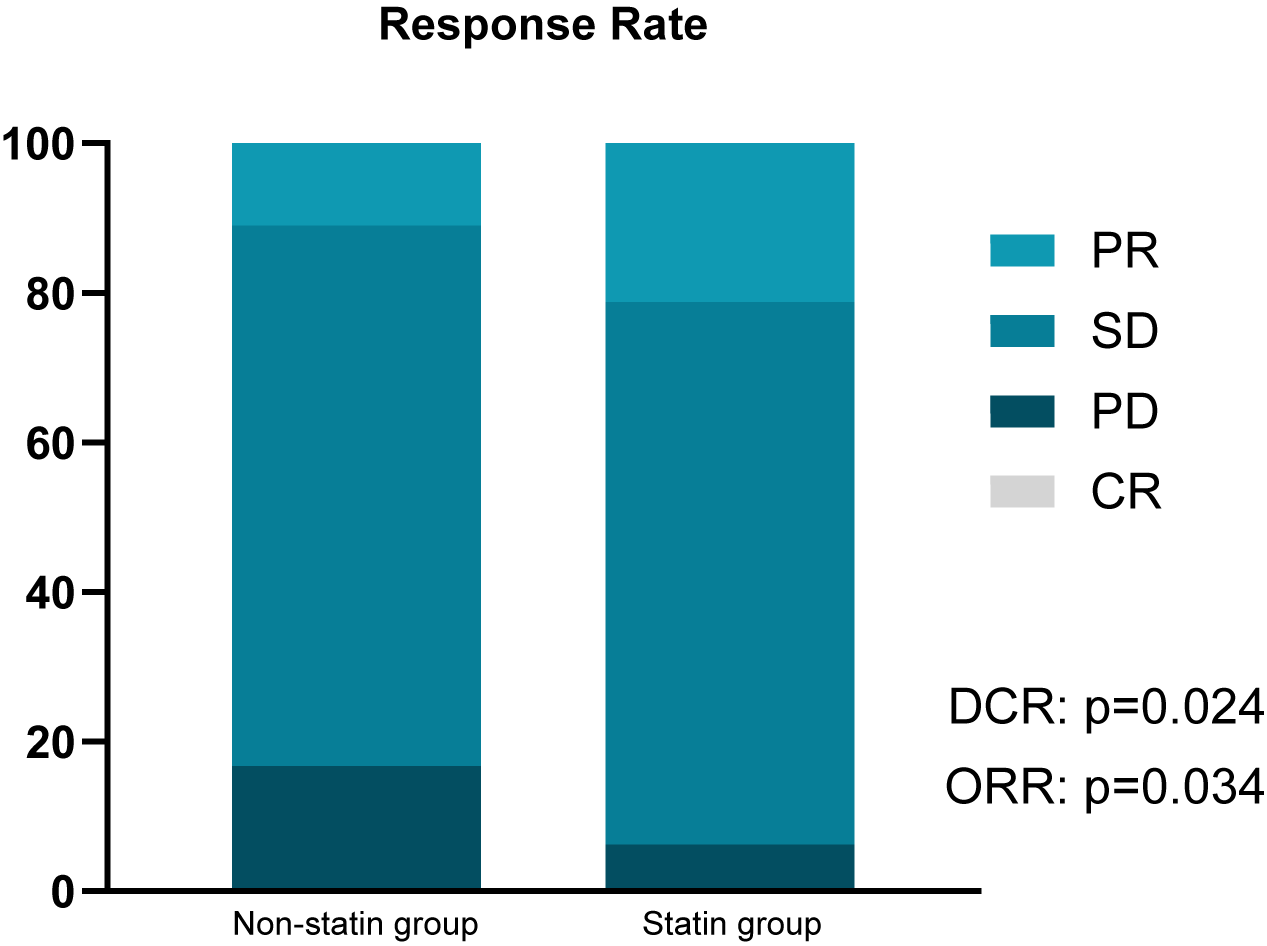

Supplement: Supplementary Figure 1 — Comparison of therapy response between statin users and non-statin users in the first evaluation. [file DataSheet1.zip › FigureS1.tif]

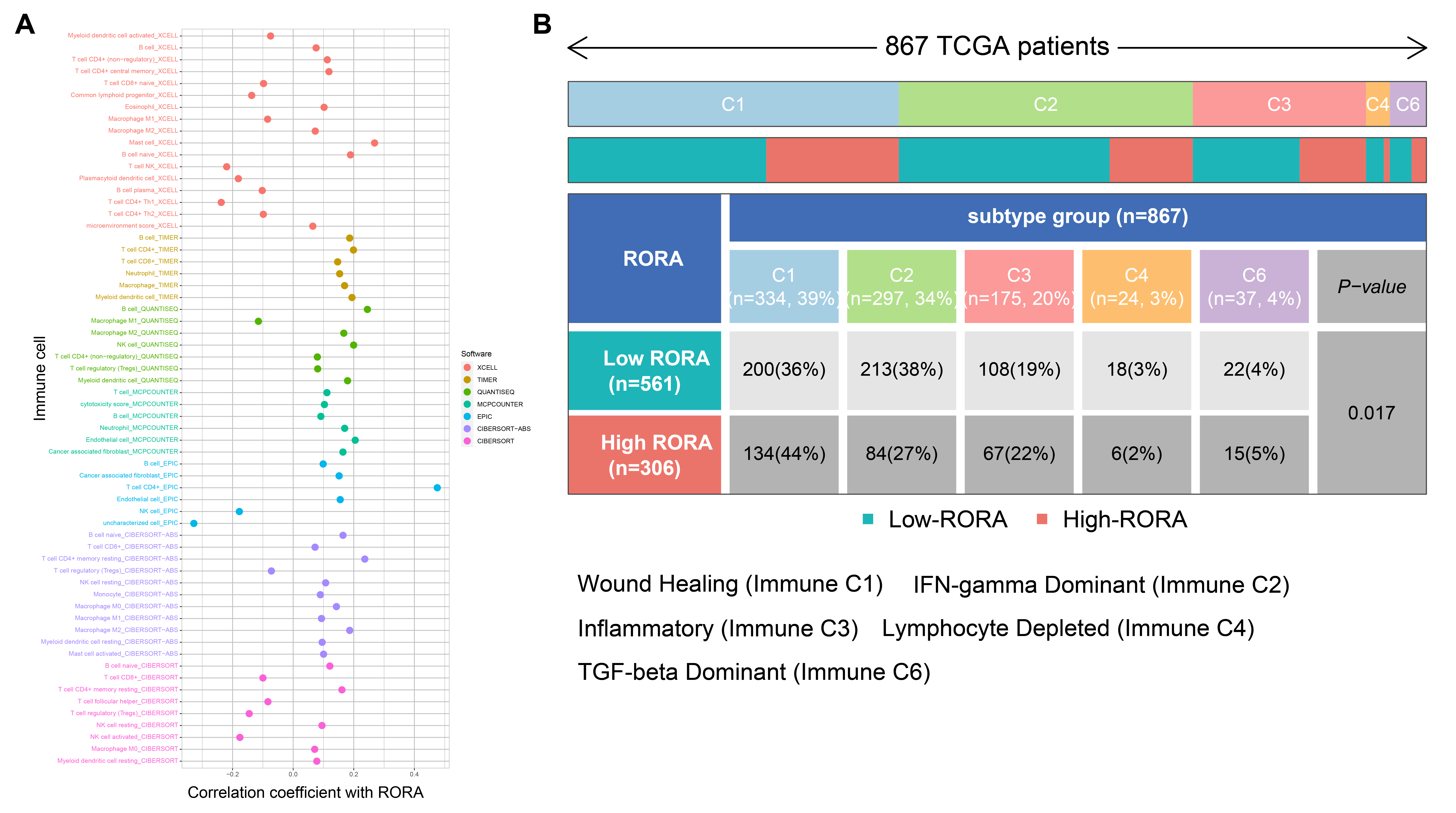

Supplement: Supplementary Figure 1 — Comparison of therapy response between statin users and non-statin users in the first evaluation. [file DataSheet1.zip › FigureS10.tif]

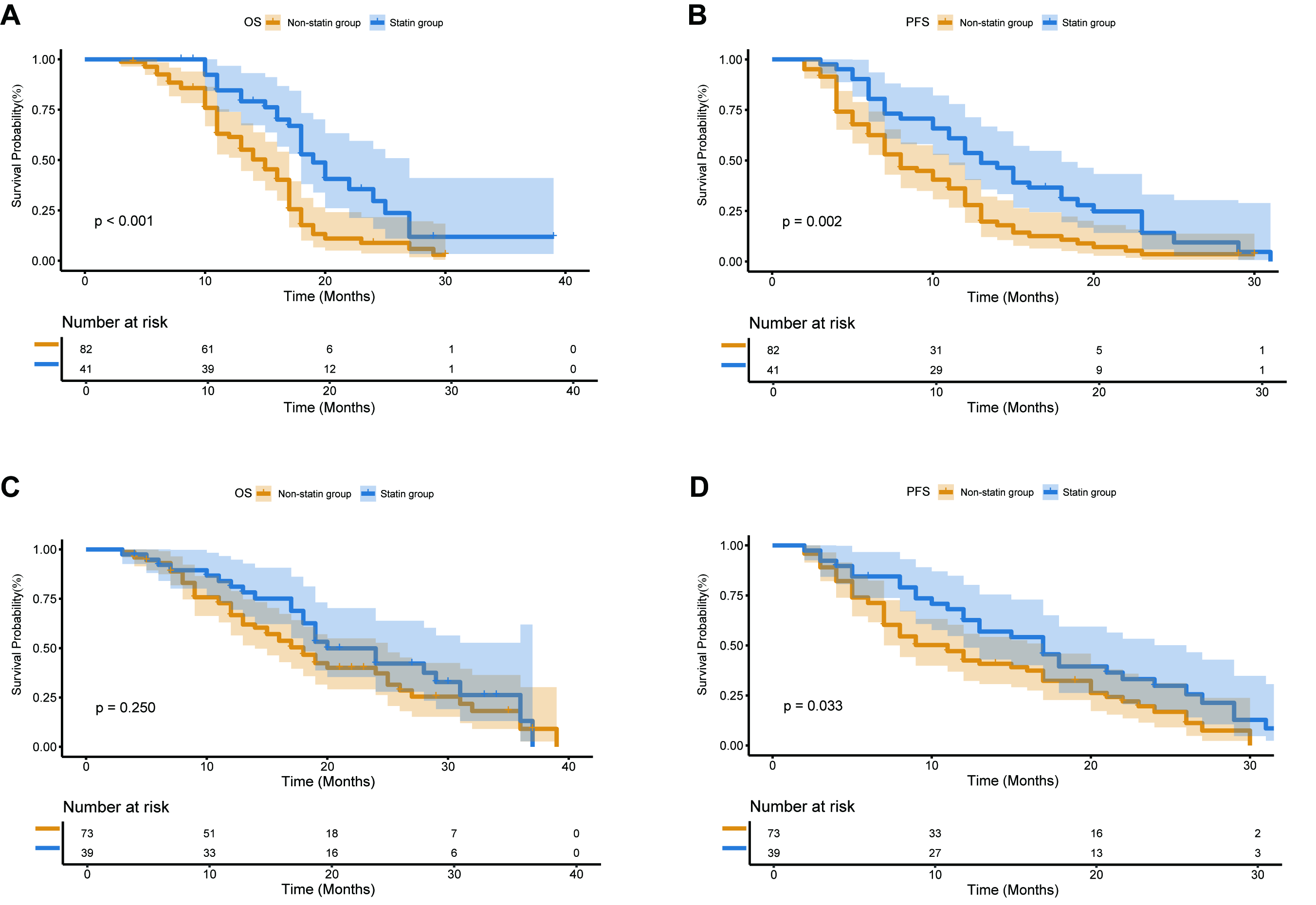

Supplement: Supplementary Figure 1 — Comparison of therapy response between statin users and non-statin users in the first evaluation. [file DataSheet1.zip › FigureS3.tif]

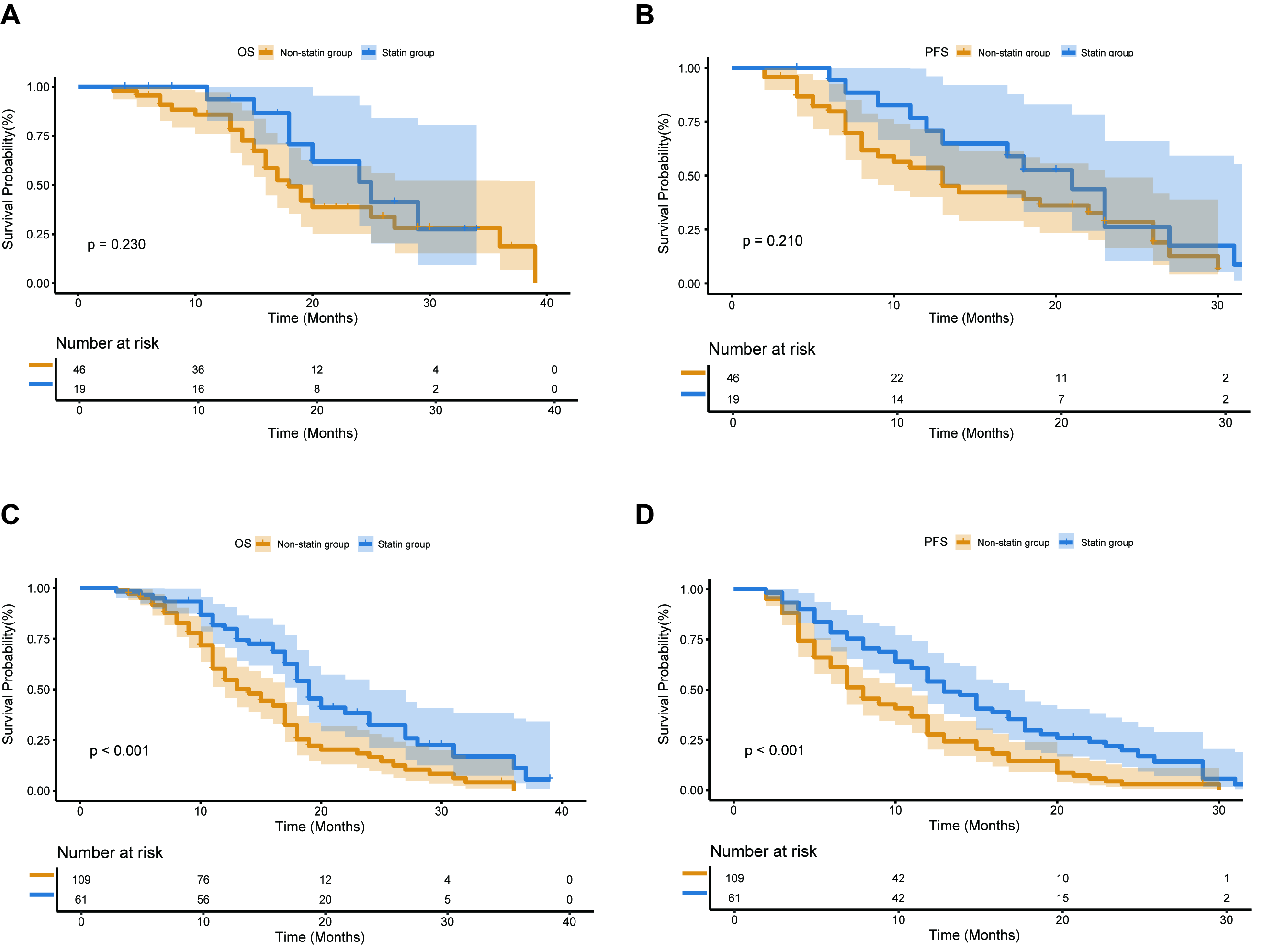

Supplement: Supplementary Figure 1 — Comparison of therapy response between statin users and non-statin users in the first evaluation. [file DataSheet1.zip › FigureS5.tif]

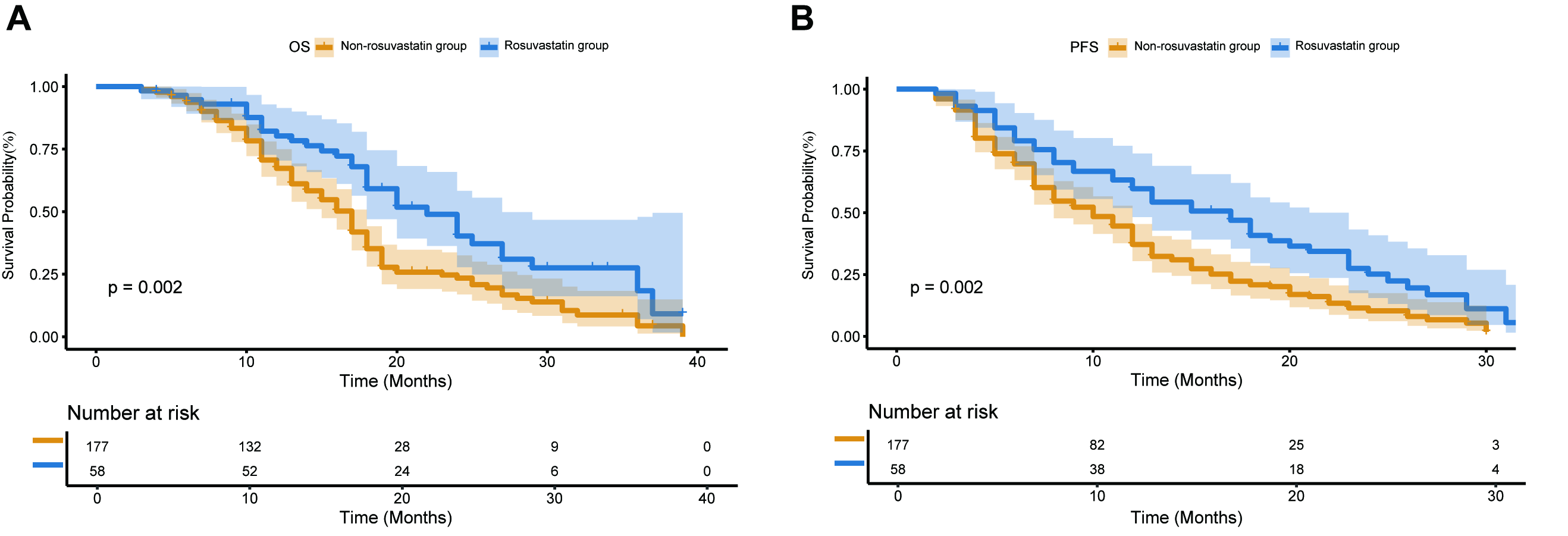

Supplement: Supplementary Figure 1 — Comparison of therapy response between statin users and non-statin users in the first evaluation. [file DataSheet1.zip › FigureS6.tif]

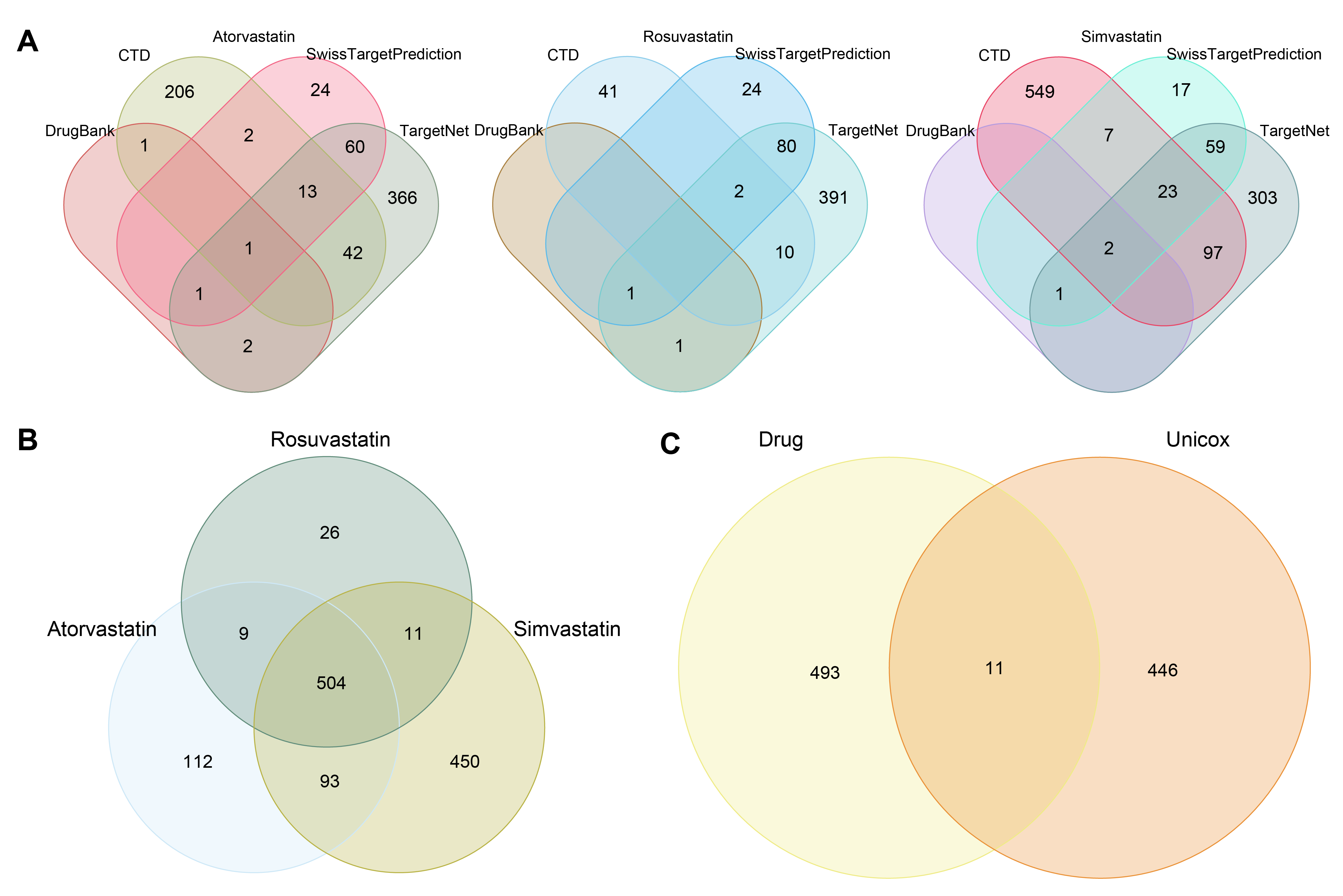

Supplement: Supplementary Figure 1 — Comparison of therapy response between statin users and non-statin users in the first evaluation. [file DataSheet1.zip › FigureS7.tif]

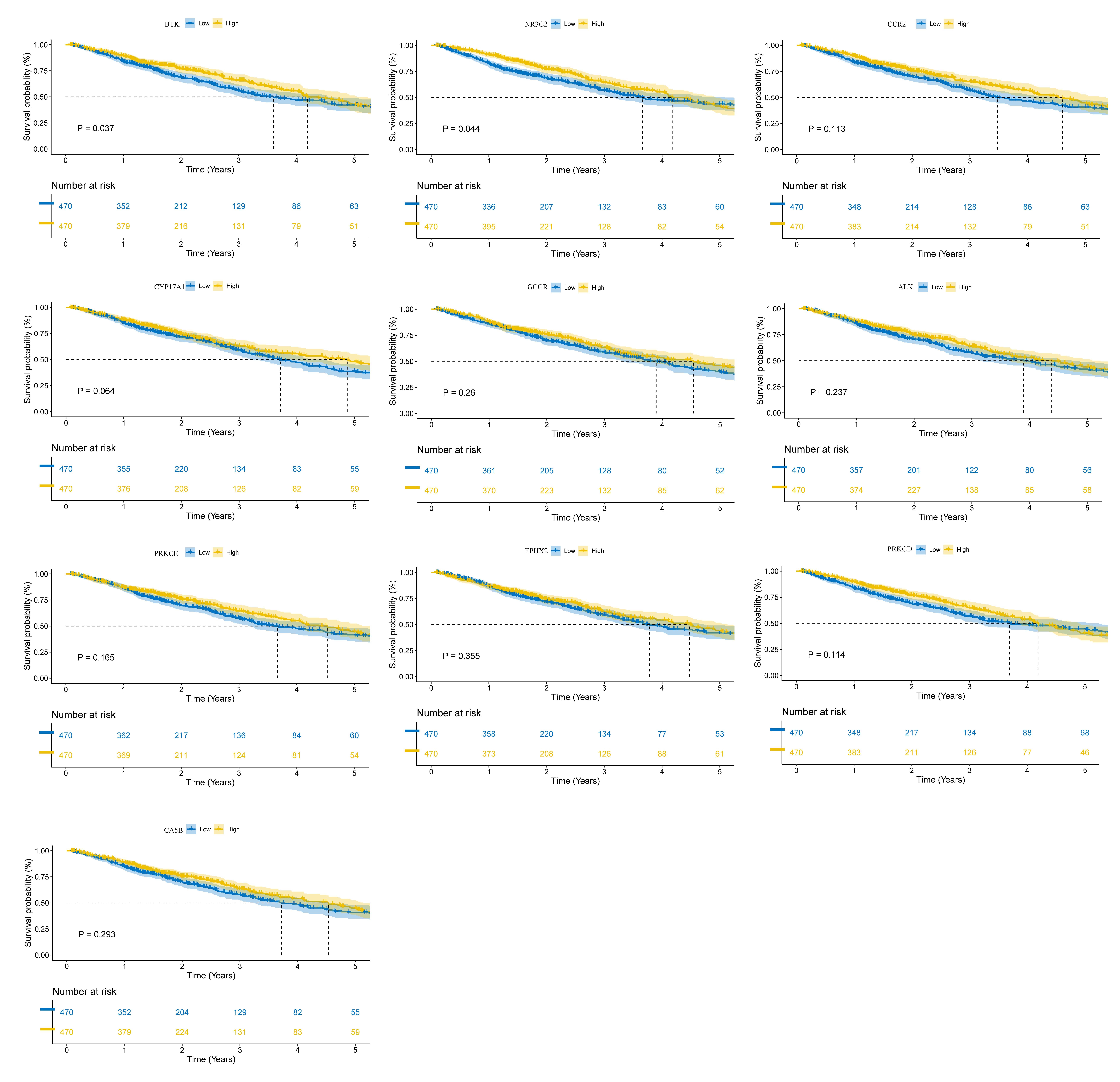

Supplement: Supplementary Figure 1 — Comparison of therapy response between statin users and non-statin users in the first evaluation. [file DataSheet1.zip › FigureS8.tif]

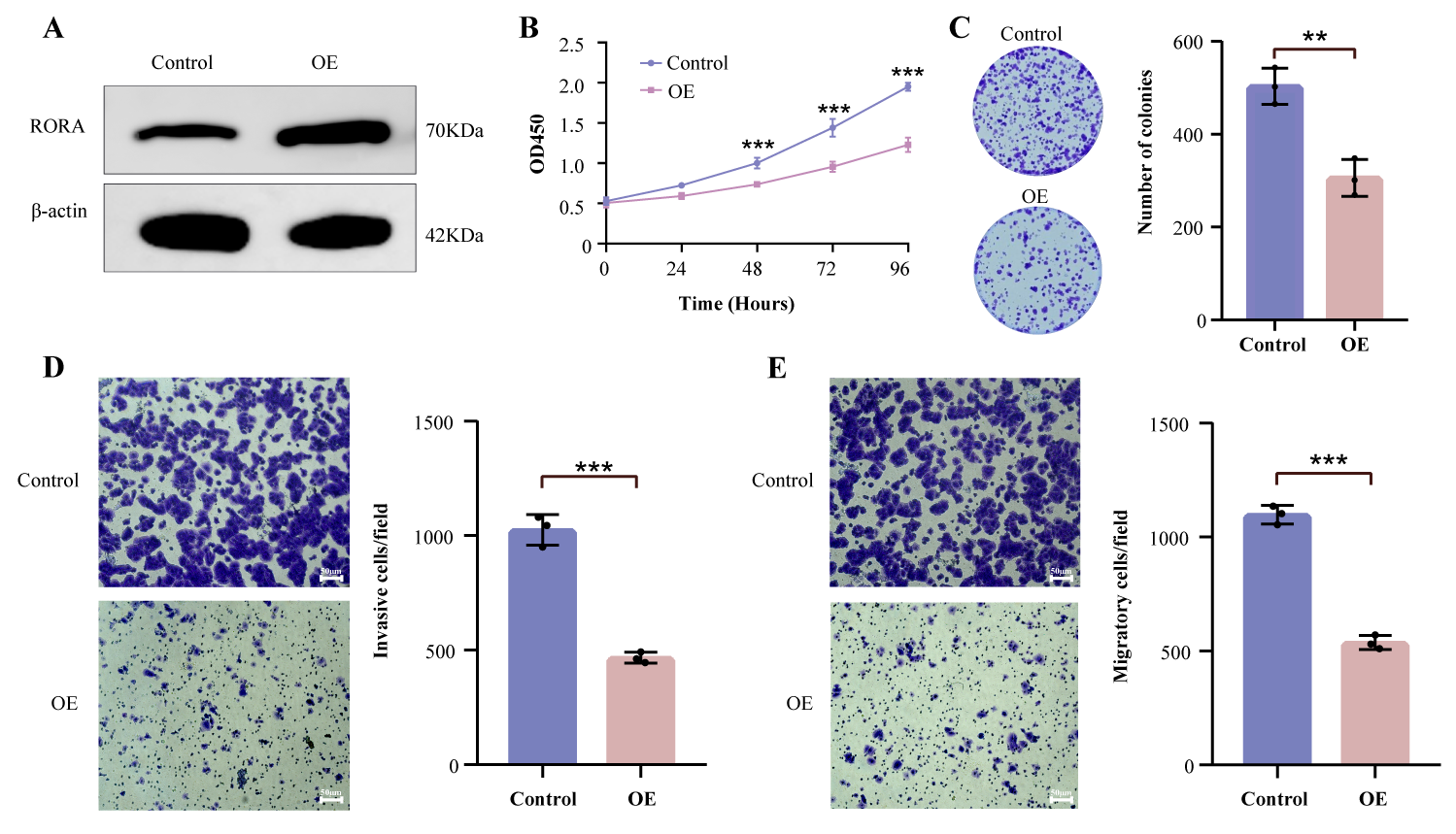

Supplement: Supplementary Figure 1 — Comparison of therapy response between statin users and non-statin users in the first evaluation. [file DataSheet1.zip › FigureS9.tif]

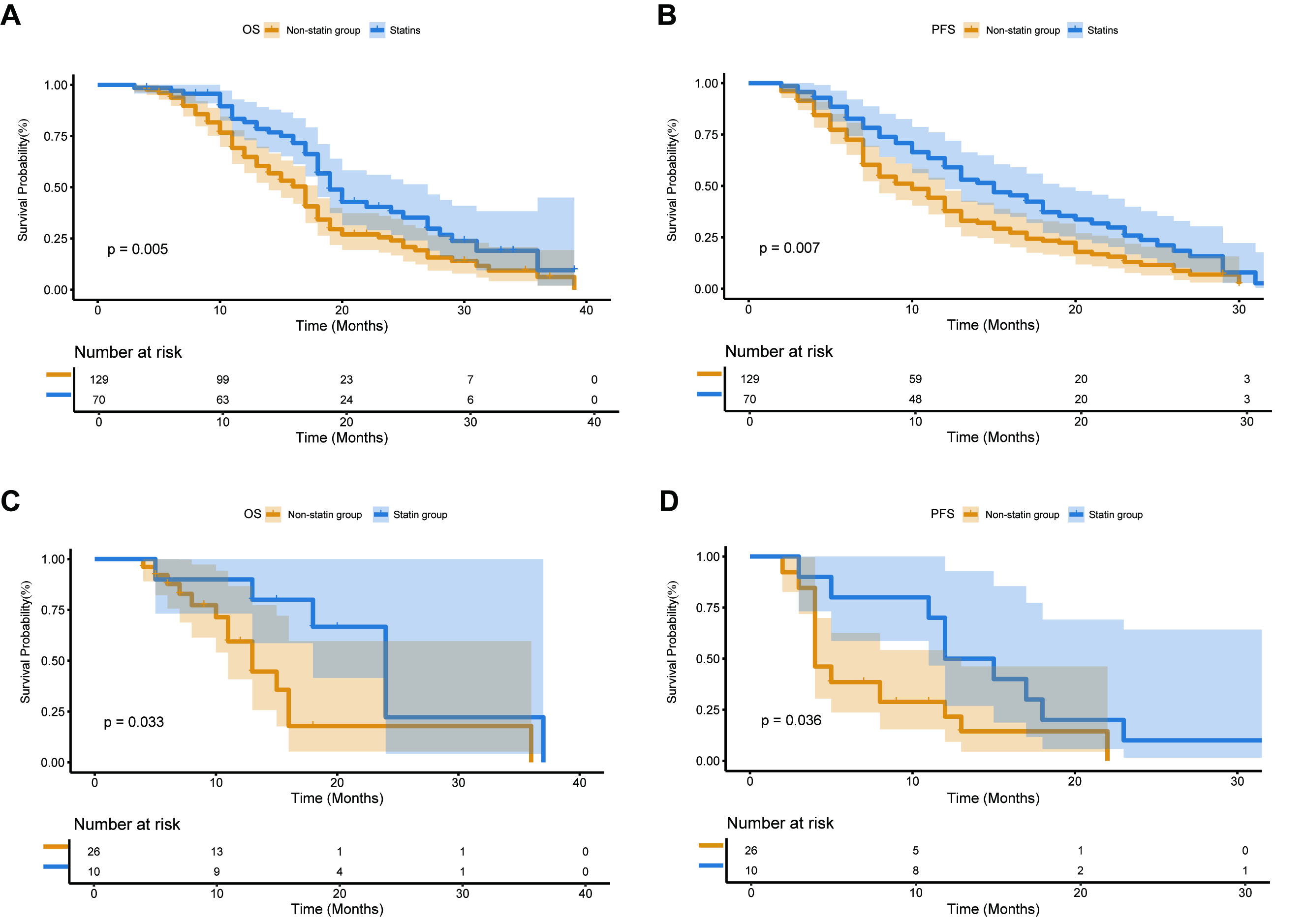

Supplement: Supplementary Figure 1 — Comparison of therapy response between statin users and non-statin users in the first evaluation. [file DataSheet1.zip › FigureS4.tif]

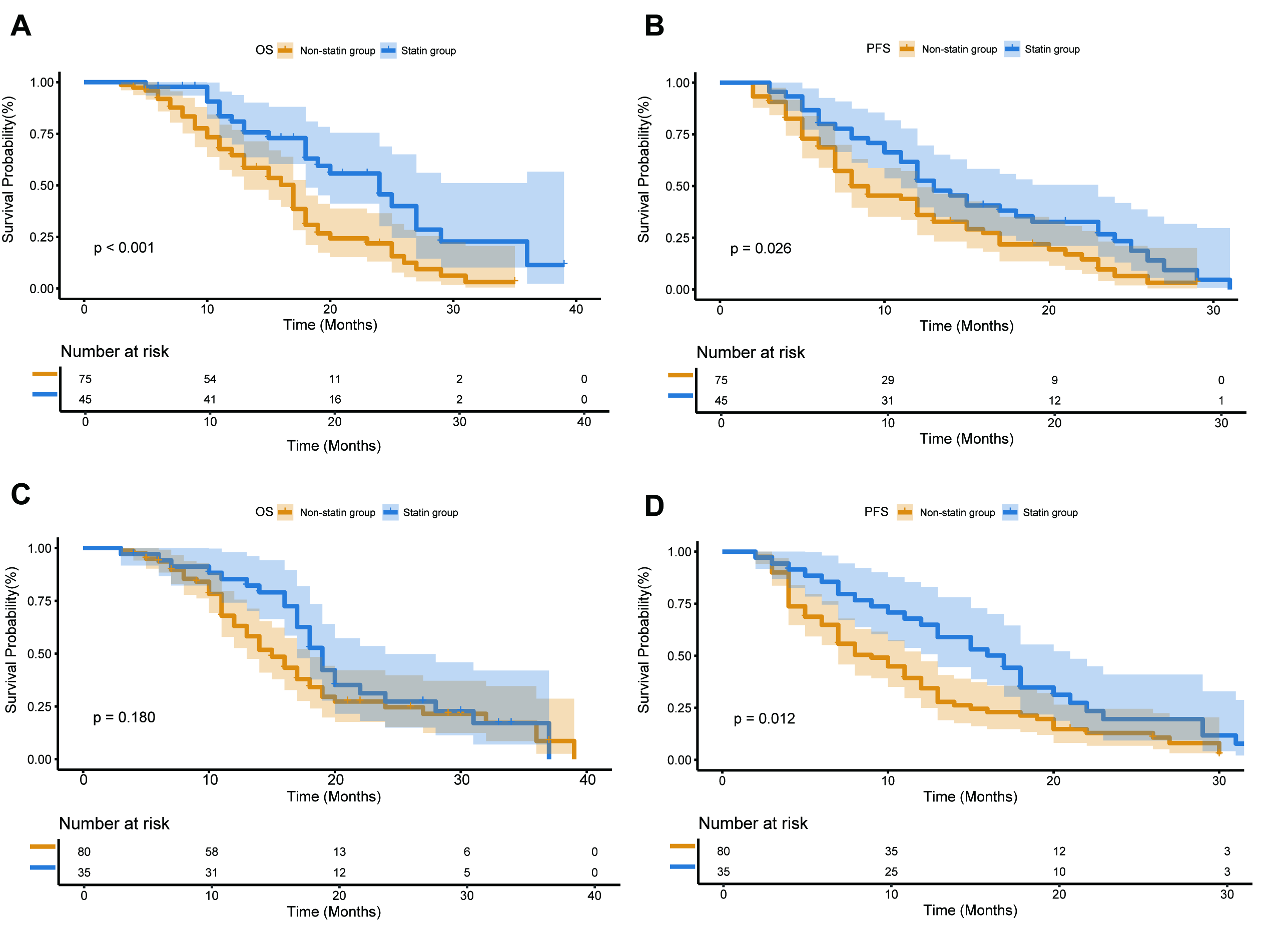

Supplement: Supplementary Figure 1 — Comparison of therapy response between statin users and non-statin users in the first evaluation. [file DataSheet1.zip › FigureS2.tif]
